# Unintended pregnancy among women living with HIV and its predictors in East Africa, 2024. A systematic review and meta-analysis

**DOI:** 10.1371/journal.pone.0310212

**Published:** 2024-12-27

**Authors:** Eyob Shitie Lake, Mulat Ayele, Abebaw Alamrew, Befikad Derese Tilahun, Besfat Berihun Erega, Alemu Birara Zemariam, Getinet Kumie, Gizachew Yilak

**Affiliations:** 1 Department of Midwifery, College of Medicine and Health Science, Woldia University, Woldia, Ethiopia; 2 Department of Nursing, College of Medicine and Health Science, Woldia University, Woldia, Ethiopia; 3 Department of Midwifery, College of Medicine and Health Science, Debre Tabor University, Debre Tabor, Ethiopia; 4 Department of Medical Laboratory, College of Medicine and Health Science, Woldia University, Woldia, Ethiopia; Hospital Femina, BRAZIL

## Abstract

**Introduction:**

An unintended pregnancy refers to a situation where a pregnancy occurs either when there is no desire for a child (unwanted) or when it takes place at a time that was not anticipated (mistimed). Pregnant women infected with HIV face a two to tenfold increased risk of mortality during both pregnancy and the postpartum period compared to those who are not infected. A national level cohort study has identified that about 70 babies born HIV positive, 60% of them were from unplanned pregnancy. In pregnant women living with HIV and on antiretroviral therapy, preterm birth and low birth weight have been reported. A systematic review and meta-analysis were conducted on the rate of vertical transmission of HIV in East Africa and revealed the pooled prevalence of 7.68% (ranges from 1.58–32.1%), which is far from the desired target of WHO, which is below 5%.

**Methods:**

Appropriate and comprehensive searches of PubMed, MEDLINE, EMBASE, Google Scholar, HINARI, and Scopus have been performed. The electronic literature search was last performed on December 28/2023. All observational study designs were eligible in this SRMA (systematic review and meta-analysis). Primary studies lacking the outcome of interest, were excluded from the SRMA. The extracted Microsoft Excel spreadsheet data were imported into the STATA software version 17 (STATA Corporation, Texas, USA) for analysis. A random-effects model was used to estimate the pooled prevalence of unintended pregnancy among women living with HIV in East Africa. The Cochrane Q-test and I^2^ statistics were computed to assess the heterogeneity among the studies included in the SRMA.

**Result:**

A total of 2140 articles were found by using our search strategies and finally ten studies were included in the SRMA, comprised of 4319 participants. The pooled prevalence of unintended pregnancy among women living with HIV in East Africa was 40.98% (95% CI: 28.75, 53.20%). The finding of this subgroup analysis by study country showed that the pooled prevalence of unintended pregnancy among women living with HIV was lower in Ethiopia (28.38%; 95% CI: 15.54, 41.21%) and higher in Rwanda (62.7%; 95% CI: 58.71, 66.69%). Unemployment (AOR = 2.75, 95% CI: 1.82, 4.16), high parity (AOR = 3.16, 95% CI: 2.34, 4.36) and no formal education (AOR = 2.04, 95% CI: 1.23, 3.38) were significantly associated with unintended pregnancy among women living with HIV in East Africa.

**Conclusion:**

The findings of this SRMA suggest a substantial need for concerted efforts to reduce unintended pregnancies among women living with HIV. It underscores the importance of continuous and rigorous initiatives to enhance women’s empowerment, focusing on improving both employment and educational status. Additionally, all stakeholders are urged to diligently implement the WHO recommendations, particularly emphasizing a four-pronged approach to a comprehensive PMTCT strategy and the prevention of unintended pregnancies.

## Introduction

Unintended pregnancy remains an enduring and worldwide concern, carrying significant implications for the health and overall welfare of mothers, infants, and families including Prenatal depression, postpartum depression, maternal experience of interpersonal violence, preterm birth, and infant low birth weight [1, 2]. An unintended pregnancy refers to a situation where a pregnancy occurs either when there is no desire for a child (unwanted) or when it takes place at a time that was not anticipated (mistimed) [3]. Unintended pregnancies exert a detrimental influence on the personal lives of women, their families, and society at large. On a global scale, 74 million women experienced unintended pregnancies in low and middle-income countries, leading to approximately 25 million unsafe abortions and 47 thousand maternal deaths annually. The significant prevalence of unintended pregnancies contributes to adverse outcomes such as preterm births and other unfavorable pregnancy results and the problem peaks in eastern Africa [4].

A global comparative study has assessed the differences in the rates of unintended pregnancies across various countries worldwide. For instance, the estimated unintended pregnancy rates range from 11 per 1000 women of reproductive age in Southern Europe to 145 per 1000 in Uganda [5]. On a global scale, HIV-related factors played a role in 19,000–56,000 maternal deaths in 2011, accounting for 6%–20% of all maternal deaths. Pregnant women infected with HIV face a two to ten-fold increased risk of mortality during both pregnancy and the postpartum period compared to those who are not infected [6]. The prevalence of unplanned pregnancy in South Africa was reported around 71% among women living with HIV and women who reported unplanned pregnancies were more likely to book their appointments late and have lower CD4 counts [7]. In pregnant women living with HIV and on antiretroviral therapy, preterm birth and low birth weight have been reported [8].

Eastern and Southern Africa is a region where 20.6 million people were living with HIV by 2021. Within this region, 670,000 people acquired the virus, and 280,000 individuals succumbed to HIV-related conditions by the conclusion of 2021. Additionally, there were 160,000 new infections reported among children aged 0–14 years old [9]. A systematic review and meta-analysis (SRMA) were conducted in 2019 on the rate of vertical transmission of HIV in East Africa and revealed the pooled prevalence of 7.68% [10] (ranges from 1.58–32.1%), which is far from the desired target of WHO, which is below 5%.

The impact of unintended pregnancy accounts for the majority of new HIV infections globally. For instance, according to the UNICEFs’ reports, about 130,000 new HIV infections worldwide among children under five occurred in 2022 and most of the cases are due to vertical transmission from the mother to the child [11]. A lower rate of viral load suppression at the time of delivery was linked to unintended pregnancies, illustrated by the fact that 95.3% of women with planned pregnancies achieved viral suppression, whereas only 76.6% of women with unplanned pregnancies experienced the same outcome [12]. This discrepancy is a critical factor influencing the likelihood of vertical transmission of the virus from the mother to the child. Women living with HIV who were on anti-retroviral therapy were followed after facing unintended pregnancy and higher rate of elevated viral load after birth was observed when compared with women who had planned pregnancy [13].

WHO recommends a four-pronged approach to a comprehensive PMTCT (prevention of mother-to-child transmission) strategy and prevention of unintended pregnancy is the second prong mentioned by providing effective and appropriate contraceptives [14]. Despite reducing MTCT of HIV since the introduction of the ‘Global Plan towards the Elimination of New HIV Infections among Children and Keeping their Mothers Alive’ in 2011, the progress is not fast enough to reach the 2025 targets set by UNAIDS [11]. There exists a significant variation in the occurrence of unintended pregnancies among women who are HIV-positive, ranging from 19.3% [15] to 74% [16] in various regions of East Africa. Therefore, this SRMA aimed to identify the pooled prevalence of unintended pregnancy among women living with HIV and its predictors in East Africa, 2024.

## Objective

To estimate the pooled prevalence of unintended pregnancy among women living with HIV and its predictors in East Africa, 2024.

## Justification of study

Avoiding unintended pregnancy among women living with HIV is essential in preventing plenty of maternal morbidity and mortality during antenatal and postnatal periods. Reducing the rate of vertical transmission of HIV from the infected mother to her offspring should be primarily achieved by avoiding unplanned and mistimed pregnancies from women living with HIV. Specially, in the areas like eastern Africa, where the knowledge and practices towards the prevention of new HIV infection is low, the problem worsens. There exists a significant variation in the occurrence of unintended pregnancies among women who are HIV-positive, ranging from 19.3% to 74% in various regions of East Africa. Thus, the pooled prevalence of unintended pregnancies and its predictors among women living with HIV should be determined.

## Methods

### Study design and setting

A SRMA were conducted on unintended pregnancy among women living with HIV and its predictors in East Africa. Preferred Reporting Items for Systematic Review and Meta-Analysis (PRISMA) guidelines were followed (S1 File). PRISMA is a protocol consisting of checklists that guide the conduct and reporting of systematic reviews and meta-analyses, which increase the transparency and accuracy of reviews in medicine and other fields [17]. The population of Eastern Africa constitutes 6.03% of the global population, making it the leading sub-region in Africa in terms of population. As of January 22, 2024, the estimated population of Eastern Africa is 491,899,592, according to the latest United Nations data [18]. After a thorough searching of literatures has been made, four studies has been selected from Ethiopia [19–22], three from Kenya [15, 23, 24], two from Uganda [25, 26] and one study has been selected from Rwanda [27].

### Search strategies

A SRMA was conducted on unintended pregnancy among women living HIV and its predictors in the eastern Africa. Both published and unpublished literatures on the prevalence and/or factors associated with unintended pregnancy among women living with HIV in eastern Africa were searched via two authors (ESL, and MA). Literatures written in English were eligible in the review. Appropriate and comprehensive searches of PubMed, MEDLINE, EMBASE, Google Scholar, HINARI, and Scopus have been performed. Furthermore, relevant articles found in the gray literature available on local shelves and institutional repositories were systematically reviewed. Medical Subject Headings (MeSH) and key terms had been developed using different Boolean operators, ‘AND’ and ‘OR’. The following search terms were used: (Prevalence) OR (proportion) OR (Magnitude) AND (unintended pregnancy) AND (women living with HIV) AND (predictors) OR (determinants) OR (associated factors) AND (East Africa)

The electronic literature search was last performed on December 28/2023. Mendeley reference manager software was used to collect and manage the literatures as well as to avoid possible duplications.

### Eligibility criteria

#### Inclusion

This SRMA included articles conducted in all countries of East Africa, reporting the prevalence/proportion/ of unintended pregnancy among women living with HIV and its predictors. Both published articles and grey literatures published in English were included.

#### Exclusion

Primary studies lacking the outcome of interest (unintended pregnancy among women living with HIV and its predictors), were excluded from the SRMA. In contrast, studies with poor quality according to the criteria of reviewing articles were excluded.

### Outcome measurement

This SRMA has comprised of two main outcomes. The primary outcome of this SRMA was the proportion of unintended pregnancy among women living with HIV, a pregnancy that occurred when no more children were desired or one that occurred earlier than it was desired or occurred when the woman did not desire to become pregnant [3]. The secondary outcome was the predictors of unintended pregnancy among women living with HIV.

### Data extraction

All the datasets were exported to the Mendeley reference manager and transferred to a Microsoft Excel spreadsheet to remove duplicate data in the review. Two authors (ESL and MA) independently extracted all important data using a standard data extraction format developed according to the Joanna Briggs Institute (JBI) Reviewers’ manual 2014 [28]. Any disagreement between reviewers was resolved by a third author (GY). A consensus was reached through critical discussion and evaluation of the articles by all independent reviewers. The name of the author, sample size, publication year, study country, study design, prevalence/proportion of unintended pregnancy, and adjusted odds ratio with its 95% confidence interval (CI) of the factors associated with unintended pregnancy. Articles that fulfilled the predetermined criteria were used as data sources for the final analysis.

### Quality assessment

Once the database results were exported to the Mendeley reference manager and duplicate results were removed, we used the Newcastle–Ottawa Quality Assessment Scale (NOS) adapted for observational studies to assess the quality of each study included in the SRMA (S2 File). The quality assessment scale evaluates the literatures in three categories.

- Selection (4 points)
- Comparability (2points) and
- Outcome (3 points)

Two Authors (ESL and MA) assessed the quality of each study (methodological quality, sample selection, sample size, comparability, outcome, and statistical analysis). In case of disagreement between the two authors, a third author (GY) was involved and discussed and resolved the disagreement.

### Data processing and analysis

The extracted Microsoft Excel spreadsheet data were imported into the STATA software version 17 (STATA Corporation, Texas, USA) for analysis. A random-effects model was used to estimate the pooled prevalence of unintended pregnancy among women living with HIV in eastern Africa. The Cochrane Q-test and I^2^ statistics were computed to assess the heterogeneity among the studies included in the SRMA. Accordingly, if the result of I ^2^ is 0–40% it is mild heterogeneity, 40 to 70% would be moderate heterogeneity, and 70 to 100% would be considerable heterogeneity [29]. Funnel plots and Egger’s test were used to assess publication bias. A p-value>0.05 indicated that there was no publication bias. A forest plot format was used to present the pooled prevalence of unintended pregnancy among women living with HIV in eastern Africa with 95% CI. Subgroup analyses were performed according to the country where the primary studies conducted. To identify determinant factors, we used the pooled AOR in forest plot format with its respective 95%CI. A robust statistical method, random effect model, and lastly sensitivity analysis was conducted to assess different assumptions about if the missing data affects the result.

## Result

**Fig 1:**
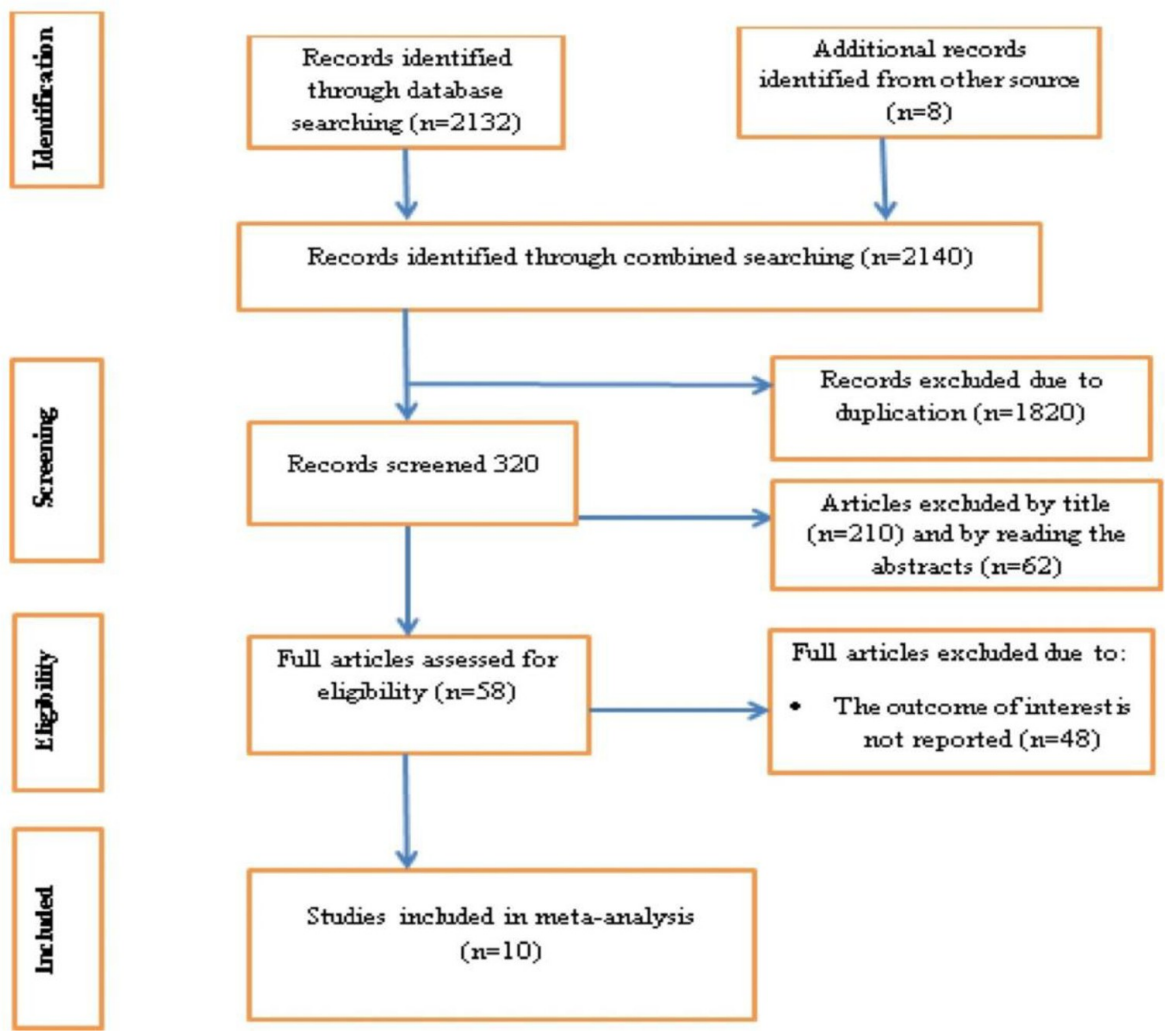
PRISMA flow chart for the selection of systematic review of unintended pregnancy among women living with HIV in East Africa.

A total of 2140 articles were found by using our search strategies: Google scholar, pub-med, Hinari, EMBASE, Scopus and Medline. After 1820 articles were removed for the reason of duplication, 320 articles left. Then by reviewing their titles and abstracts, 210 and 62 articles were removed respectively. Finally 58 full text papers were accessed and evaluated for the predefined inclusion criteria. Thus 48 more articles were excluded for the afore-mentioned reasons. Eventually, 10 articles were found eligible for inclusion in the final systematic review and meta-analysis (Fig 1).

Among the studies included in this SRMA, four studies were in Ethiopia, three in Kenya, two from Uganda and one Rwanda. Seven studies are comprised of cross sectional, two cohort and one case control study designs. A total of 4319 participants with the smallest 110 to the largest 849 participants included and found the prevalence which ranges from 19.3 [15] to 74% [16] of unintended pregnancy among women living with HIV in East Africa. On the other hand, the quality of each study was assessed by using NOS, the score of all included studies lies between 7 to 9 which indicates good quality (Table 1).

**Table 1:** Characteristics of included studies included in the SRMA of unintended pregnancy among women living with HIV and its predictors in East Africa: 2024.

Seri no	Author	Year	Country	Study design	Sample size	Prevalence of unintended pregnancy(%)	Quality
1	Samuel K et al [19]	2022	Ethiopia	Cross sectional	408	22.4	Good
2	Tigist T et al [20]	2021	Ethiopia	Cross sectional	353	41.7	Good
3	Dereje B and Rose M [21]	2021	Ethiopia	Cross sectional	670	21.4	Good
4	Yosef L et al [22]	2022	Ethiopia	Case control	304		Good
5	Agnes N et al [25]	2020	Uganda	Cross sectional	547	41.1	Good
6	Kimiyo K et al [27]	2011	Rwanda	Cross sectional	565	62.7	Good
7	Fredrick O et al [15]	2015	Kenya	Cross sectional	119	19.3	Good
8	Jana J et al [26]	2018	Uganda	Cohort	110	45	Good
9	Francis O et al [16]	2012	Kenya	Cross sectional	394	74	Good
10	Donatien B et al [24]	2014	Kenya	Cohort	849	41	Good

### Magnitude of unintended pregnancy among women living with HIV

**Fig 2:**
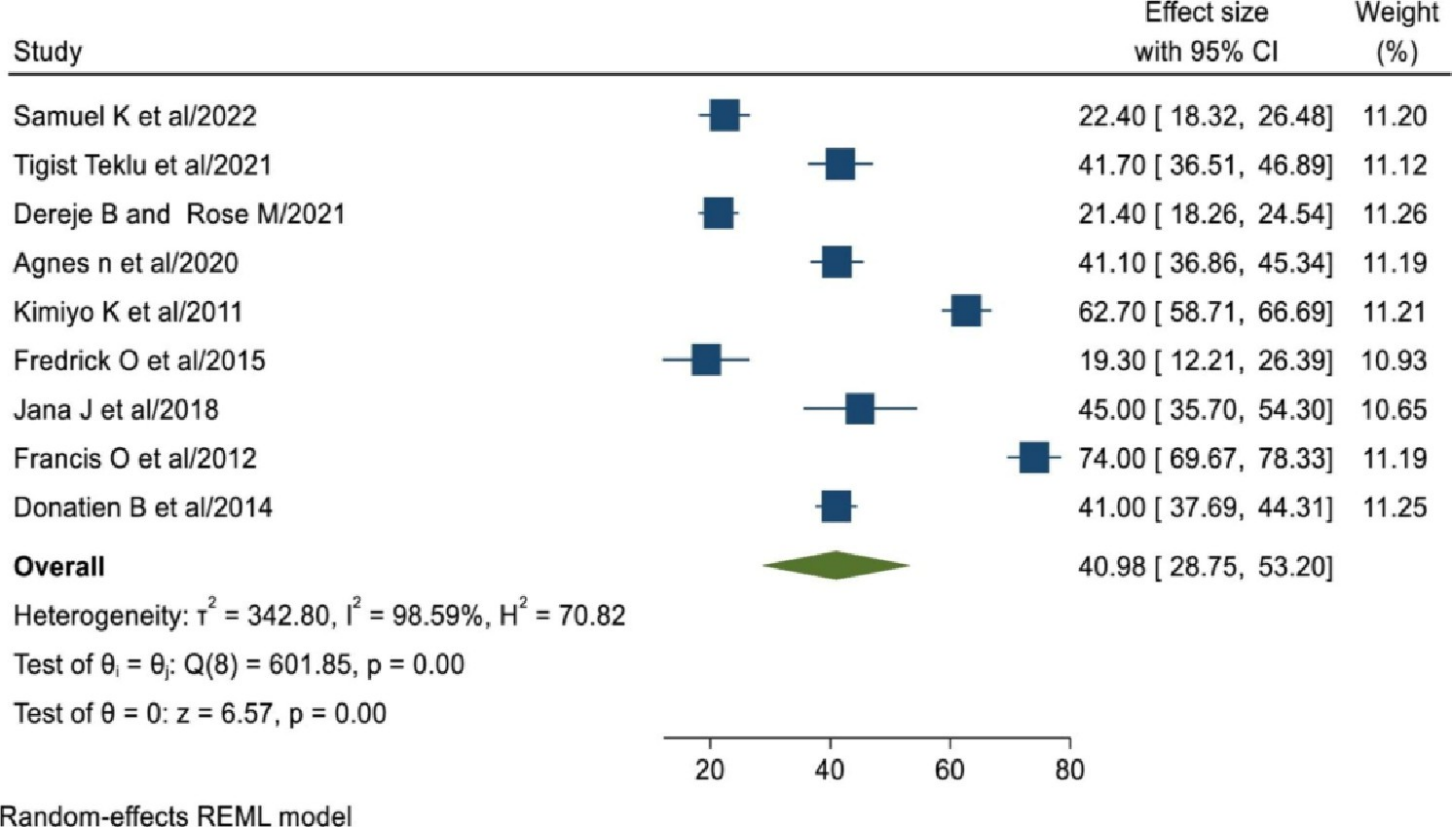
Forest plot showing the pooled prevalence of unintended pregnancy among women living with HIV in East Africa, 2024.

The pooled prevalence of unintended pregnancy among women living with HIV in East Africa was 40.98% (95% CI: 28.75,53.20), with the Cochrane heterogeneity index (I^2^ = 98.59%), P = 0.00, showing the presence of significant heterogeneity among the primary studies included. Therefore we have used the random effect model to resolve the issue of heterogeneity among included studies. Moreover, we have considered subgroup analysis as a potential way of addressing heterogeneity. The finding was presented using a forest plot (Fig 2).

### Publication bias

**Fig 3:**
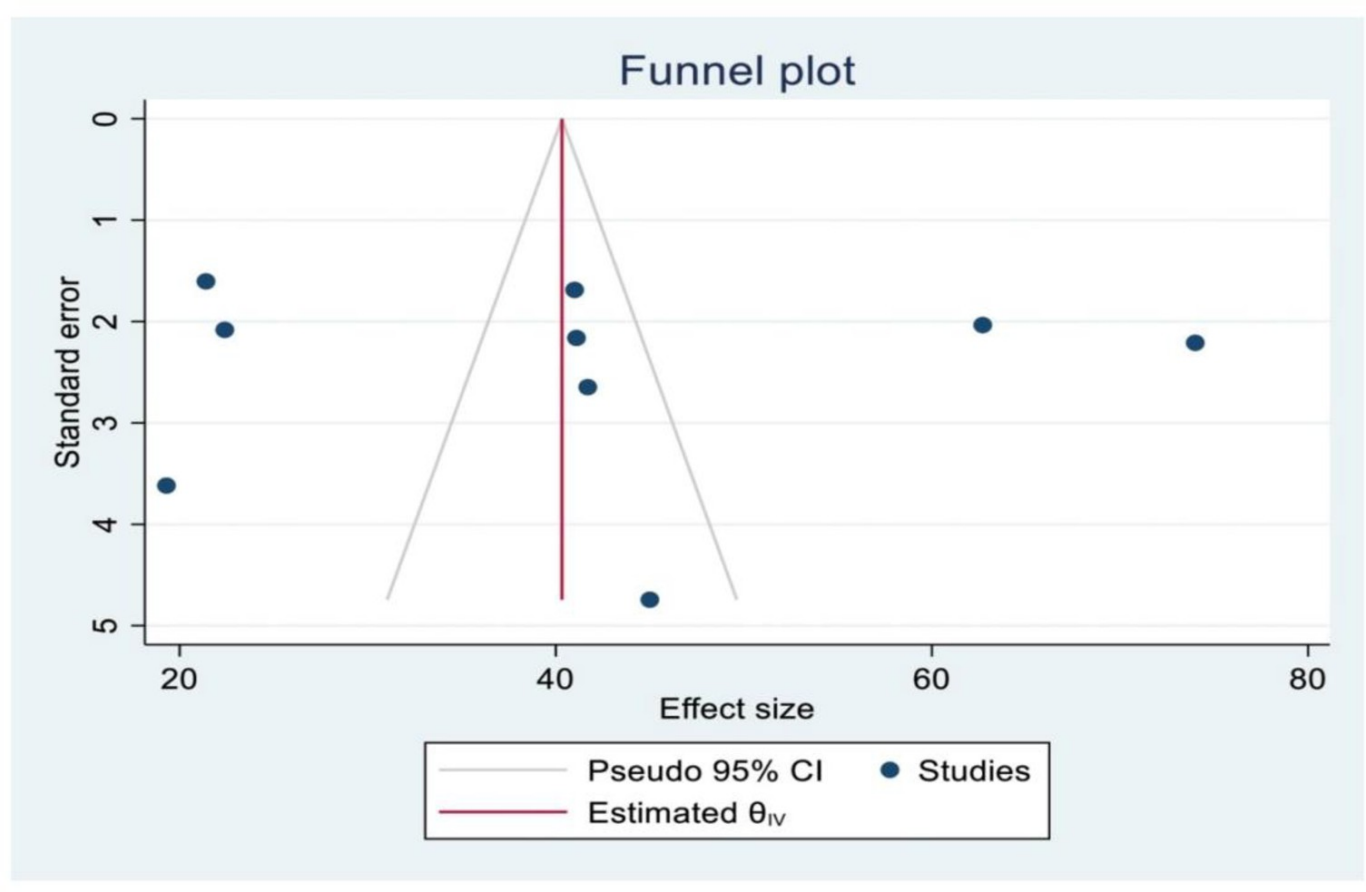
Funnel plot showing the symmetric distribution of the primary studies on unintended pregnancy among women living with HIV in Eastern Africa, 2024.

The presence or absence of publication bias was verified by using statistical analysis (funnel plot and egger’s test (P = 0.87 (P >0.05)) result showed no small study effect (Fig 3). Nevertheless, the egger’s test used to show the publication bias might be affected by the significant heterogeneity between the included studies and the small number of studies.

### Sub group analysis

**Fig 4:**
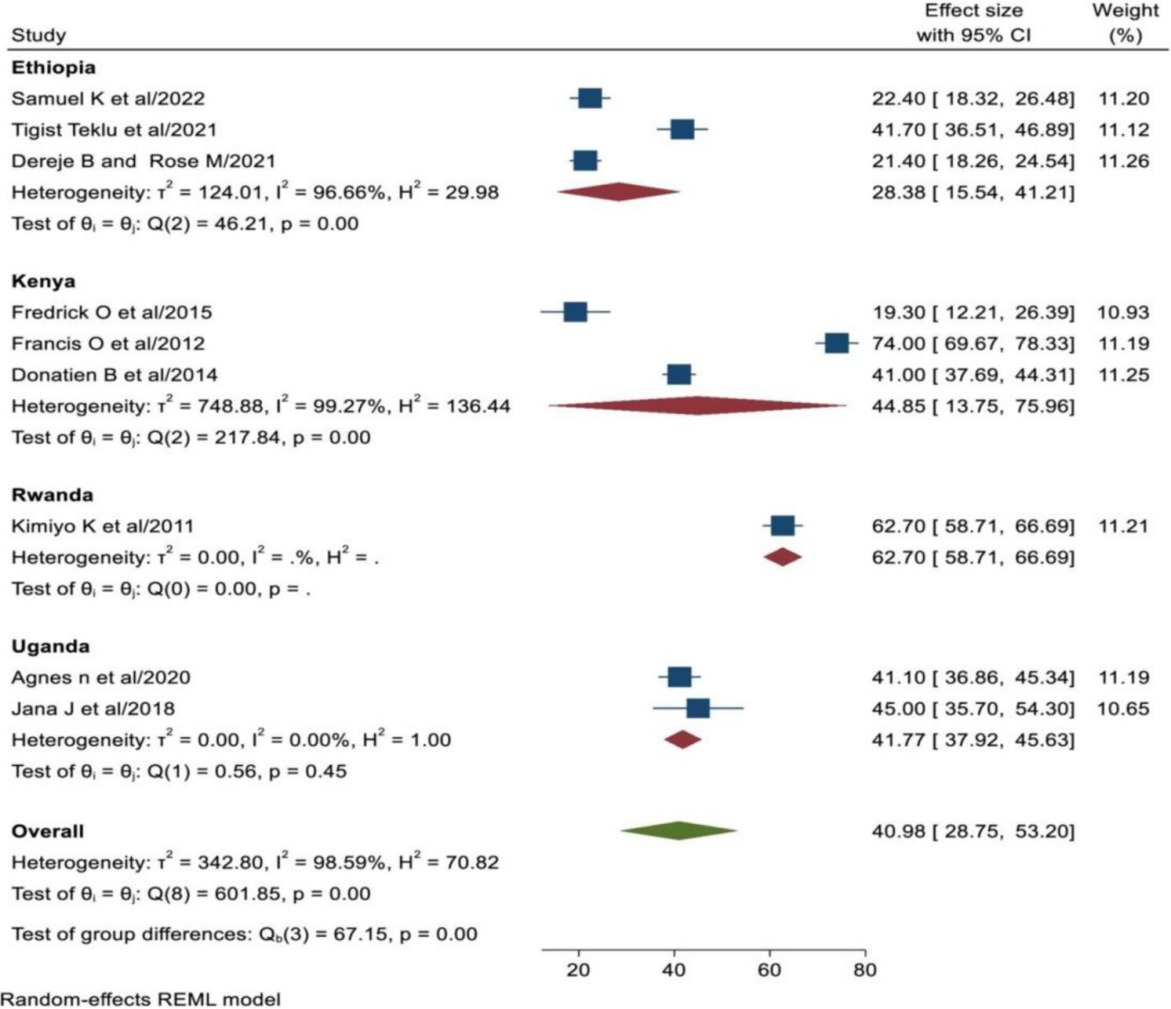
Sub group analysis of unintended pregnancy among women living with HIV by the studied country in Eastern Africa, 2024.

Subgroup analysis was done based on the studied country. The finding of this subgroup analysis by studied country showed that the pooled prevalence of unintended pregnancy in Ethiopia was lower (28.38%; (95% CI: 15.54, 41.21%), with the Cochrane heterogeneity index (I^2^ = 96.66%, P = 0.00). However, the pooled prevalence of unintended pregnancy among women living with HIV in Rwanda was higher (62.70%; (95% CI: 58.71, 66.69%), I^2^ = . P = 0.00) (Fig 4).

### Sensitivity analysis

**Fig 5:**
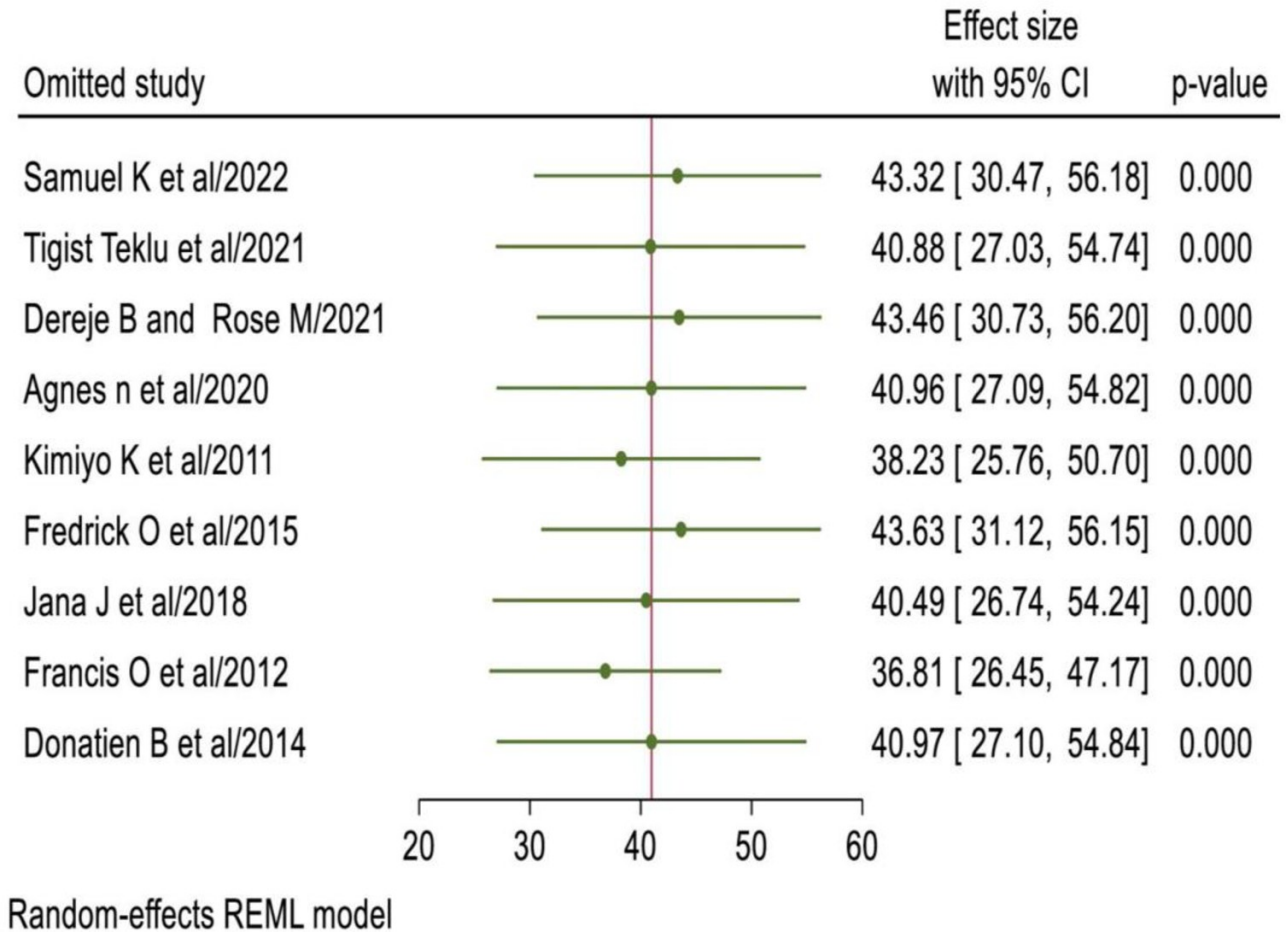
Sensitivity analysis showing the absence of single study effect on the pooled prevalence of unintended pregnancy among women living with HIV in East Africa, 2024.

The result of a random effect model revealed that, the pooled prevalence of unintended pregnancy among women living with HIV was not influenced by a single study (Fig 5).

### Determinants of unintended pregnancy among women living with HIV

In this SRMA, variables used by the primary studies were included to identify the predictors of unintended pregnancy among women living with HIV in Eastern Africa. Therefore, women with higher parity, unemployment and having no formal education were found to be the predictors of unintended pregnancy among women living with HIV in Eastern Africa, 2024. Women who have high parity were 3.16 (95% CI: 2.34, 4.36) more likely of conceiving the unintended pregnancy when compared to their counterparts. The employment status of women also determines the unintended pregnancy. The likelihood of unintended pregnancy was seen 2.75 (95% CI: 1.82, 4.16) times higher among women who are unemployed than employed women. On the other hand, women living with HIV and who have no formal education were around two times (2.04, 95% CI: 1.23, 3.38) more likely of having unintended pregnancy than those who have secondary and above education level (Table 2).

**Table 2:** Predictors of unintended pregnancy among women living with HIV in Eastern Africa, 2024.

Variable	Author name/ publication year	AOR	95% CI	Pooled AOR	95% CI
High parity	Samuel K et al/2022	4.24	2.31–7.77	3.16	2.34–4.36
	Agnes n et al/2020	2.79	1.85–4.22		
	Kimiyo K et al/2011	3.83	1.3–11.3		
	Fredrick O et al/2015	2.8	1.12–6.98		
Unemployment	Samuel K et al/2022	2.27	0.64–7.69	2.75	1.82–4.16
	Tigist Teklu et al/2021	2.42	1.34–4.36		
	Yosef L et al/2022	5.55	1.92–16.6		
	Fredrick O et al/2015	2.52	1.05–6.06		
No formal education	Yosef L et al/2022	4.62	1.6–12.67	2.04	1.23–3.38
	Kimiyo K et al/2011	1.41	0.56–3.55		
	Fredrick O et al/2015	1.37	0.5–3.7		
	Francis O et al/2012	1.43	0.42–5		
	Donatien B et al/2014	3.13	0.98–10		

## Discussion

The prevalence of unintended pregnancy among women living with HIV in Eastern Africa was 40.98% with a 95% CI (27.85–53.20). This finding is in line with previous studies conducted in South Africa [30], Nigeria [31] and Botswana [32]. The possible explanation for this higher rate of unintended pregnancy among HIV positive women is the high discontinuation and/or irregular use of contraceptives [33], unmet need of family planning services [34, 35], underutilization of emergency contraceptives and abortion services for one or another reasons. But, our study finding was higher than a study conducted in America [36], Mumbi, India [37] and South Asian countries [38]. This is because of the difference in study area, in which American women have access of optimal family planning services and abortion is legal. Furthermore, the other reason of this discrepancy is due to the fact that the study conducted in south Asian countries was conducted on the general population, not specifically done on HIV positive women. On the contrary, our study finding revealed that the prevalence of unintended pregnancy among women living with HIV much lower when compared with a study conducted in Chicago [12] and Canada [39]. This might because they have employed retrospective cohort study designs and took women only at tertiary health facilities unlike our study.

This SRMA has also identified the independent risk factors determining unintended pregnancy among women living with HIV. Unemployed women, high order parity and having no formal education were identified as the independent risk factors associated with unintended pregnancy in Eastern Africa. Women who were unemployed were 2.75 (95% CI: 1.82, 4.16) times more likely of having unintended pregnancy. This study finding is consistent with previous studies conducted in Botswana [40]. This finding could potentially be explained by the observation that, in many cases, employed women tend to have higher educational qualifications. Women with better education often have increased opportunities to access information through various media channels, contributing to their awareness and utilization of reproductive health services and contraceptive methods. Women who are employed and actively involved in paid work have a higher likelihood of accessing reproductive health services, including the adoption and updating of modern contraceptive methods. This ultimately aids in preventing unintended pregnancies [41]. Women without employment face financial insecurity, preventing them from accessing essential sexual and reproductive healthcare services, including contraceptive [42].

The likelihood of conceiving the unintended pregnancy among women with no formal education was 2.04 times (95% CI: 1.23, 3.38) higher than their counterparts. This is consistent with studies conducted in Botswana [40], Sub-Saharan Africa [43] and Philadelphia [44]. This is due to having primary and secondary education holds the capability to increase awareness among women regarding the consequences of unintended pregnancies and the potential contraceptive options that educated women may be utilizing.

High order parity is also among the determining factors of unintended pregnancy. Women with high order parity were 3.16 times (95% CI: 2.34, 4.36) more likely to have unintended pregnancy than their counterparts. This study finding is consistent with studies conducted in cape town, South Africa [45] and Senegal [46]. This can be justified as multiparous women experiences pregnancies of various intentions, including both intended and unintended, leading to an overall increase in their parity over time. on the contrary, a study conducted in Canada [47] revealed that women who have never given birth were more likely of conceiving unintended pregnancy than their counterparts. This discrepancy may be due to the difference in education level and socio-economic characteristics of the women living in Canada and Eastern Africa. Thus, multi-parous women living in Canada are more likely to have good experience and knowledge about the contraceptive utilization to avoid unintended pregnancy than women with no history of pregnancy.

The findings of this SRMA suggest a substantial need for concerted efforts to reduce unintended pregnancies among women living with HIV. It underscores the importance of continuous and rigorous initiatives to enhance women’s empowerment, focusing on improving both employment and educational status. Additionally, all stakeholders are urged to diligently implement the WHO recommendations, particularly emphasizing a four-pronged approach to a comprehensive PMTCT strategy and the prevention of unintended pregnancies. Furthermore, it is anticipated that researchers will explore potential facilitators and challenges related to unintended pregnancies among women living with HIV using a qualitative approach. This methodology is expected to yield valuable insights that can contribute to the development of effective strategies for preventing unintended pregnancies in this specific population.

Despite the efforts made to mitigate potential limitations in this SRMA, it is crucial to interpret the results in consideration of certain constraints. Firstly, our inclusion was restricted to only ten studies identified through our search strategy. Additionally, the absence of similar reviews compelled us to rely on comparisons and discussions with primary studies. Furthermore, it’s important to note that the power of Egger’s test for publication bias may be diminished when the number of included primary studies is low, especially in the presence of significant heterogeneity among them.

## Supporting information

S1 File PRISMA checklist for unintended pregnancy among women living with HIV. (DOCX)

S2 File NOS for unintended pregnancy of included studies. (DOCX)

S3 File (ZIP)

S1 Data (XLSX)
