# Supplementary material for: Unintended pregnancy among women living with HIV and its predictors in East Africa, 2024. A systematic review and meta-analysis
Source: PLoS One. 2024 Dec 27;19(12):e0310212. doi: 10.1371/journal.pone.0310212 (PMC11676498; doi:10.1371/journal.pone.0310212)
Supplement: S1 File — (DOCX) [file pone.0310212.s001.docx]

Newcastle-Ottawa Quality Assessment Scale for cross sectional studies used in the systematic review and meta-analysis, unintended pregnancy among women living with HIV in East Africa, 2024

| \|  \| **Selection (4)** \| \| \| \| **Comparability(2)** \| **Outcome (3)** \| \| **Total score** \| \| --- \| --- \| --- \| --- \| --- \| --- \| --- \| --- \| --- \| \| Author name \| Representativeness(1) \| Sample size(1) \| Non respondents (1) \| Ascertainment of the exposure risk factor (1) \| The subjects in different outcome groups are comparable, based on the study design or analysis. confounding factors are controlled (2) \| Assessment of the outcome (2) \| Statistical test (1) \|  \| \| Samuel K et al(18) \| 1 \| 1 \| 1 \| 1 \| 1 \| 2 \| 1 \| **8** \| \| Tigist T et al(19) \| 1 \| 1 \| 1 \| 1 \| 1 \| 2 \| 1 \| **8** \| \| Dereje B and Rose M(20) \| 1 \| 1 \| 1 \| 1 \| 2 \| 2 \| 1 \| **9** \| \| Yosef L et al(21) \| 1 \| 1 \| 1 \| 1 \| 1 \| 2 \| 1 \| **8** \| \| Agnes n et al(24) \| 1 \| 1 \| 1 \| 1 \| 1 \| 2 \| 1 \| **8** \| \| Kimiyo K et al(26) \| 1 \| 1 \| 1 \| 1 \| 2 \| 1 \| 1 \| **8** \| \| Fredrick O et al(14) \| 1 \| 1 \| 1 \| 1 \| 2 \| 1 \| 1 \| **8** \| \| Jana J et al(25) \| 1 \| 1 \| 1 \| 1 \| 1 \| 2 \| 1 \| **8** \| \| Francis O et al(15) \| 1 \| 1 \| 1 \| 1 \| 2 \| 1 \| 1 \| **8** \| \| Donatien B et al(28) \| 1 \| 1 \| 1 \| 1 \| 1 \| 2 \| 1 \| **8** \| |
| --- | --- | --- | --- | --- | --- | --- | --- | --- | --- | --- | --- | --- | --- | --- | --- | --- | --- | --- | --- | --- | --- | --- | --- | --- | --- | --- | --- | --- | --- | --- | --- | --- | --- | --- | --- | --- | --- | --- | --- | --- | --- | --- | --- | --- | --- | --- | --- | --- | --- | --- | --- | --- | --- | --- | --- | --- | --- | --- | --- | --- | --- | --- | --- | --- | --- | --- | --- | --- | --- | --- | --- | --- | --- | --- | --- | --- | --- | --- | --- | --- | --- | --- | --- | --- | --- | --- | --- | --- | --- | --- | --- | --- | --- | --- | --- | --- | --- | --- | --- | --- | --- | --- | --- | --- | --- | --- | --- | --- |
