# Supplementary material for: Unintended pregnancy among women living with HIV and its predictors in East Africa, 2024. A systematic review and meta-analysis
Source: PLoS One. 2024 Dec 27;19(12):e0310212. doi: 10.1371/journal.pone.0310212 (PMC11676498; doi:10.1371/journal.pone.0310212)
Supplement: S3 File — (ZIP) [file pone.0310212.s003.zip › Excluded studies after reading the abstract.docx]

***Excluded studies after reading the abstract***

1. Abay F, Yeshita HY, Mekonnen FA, Sisay M. Dual contraception method utilization and associated factors among sexually active women on antiretroviral therapy in Gondar City, northwest, Ethiopia: a cross sectional study. BMC Womens Health. 2020;20:1–9.
2. Gelagay AA, Koye DN, Yeshita HY. Demand for long acting contraceptive methods among married HIV positive women attending care at public health facilities at Bahir Dar City, Northwest Ethiopia. Reprod Health. 2015;12:1–9.
3. Araya BM, Solomon AA, Gebreslasie KZ, Gudayu TW, Anteneh KT. The role of counseling on modern contraceptive utilization among HIV positive women: the case of Northwest Ethiopia. BMC Womens Health. 2018;18:1–9.
4. Mbongueh MC, Nicholas T, Ndum AC, Gisèle EL, Nguedia A, Clement J. Unintended pregnancy and sexually transmissible infections amongst adolescents and young adults in Douala IV municipality, Cameroon: Prevalence, knowledge, and associated factors. 2023;
5. Magadi MA. HIV and Unintended Fertility in Sub-Saharan Africa: Multilevel Predictors of Mistimed and Unwanted Fertility Among HIV-Positive Women. Popul Res Policy Rev. 2021;40(5):987–1024.
6. Feyissa TR, Melka AS. Demand for modern family planning among married women living with HIV in western Ethiopia. PLoS One. 2014;9(11):e113008.
7. Dibaba Y, Fantahun M, Hindin MJ. The association of unwanted pregnancy and social support with depressive symptoms in pregnancy: evidence from rural Southwestern Ethiopia. BMC Pregnancy Childbirth. 2013;13:1–8.
8. Meseret M, Shimeka A, Bekele A. Research Article Incidence and Predictors of Tumusiigirwe K. Factors associated with unwanted pregnancies among girls aged 15 to 19 years in Kakoba Division in Mbarara District. 2017;
9. Obiyan MO, Olaleye AO, Oyinlola FF, Folayan MO. Factors associated with pregnancy and induced abortion among street-involved female adolescents in two Nigeria urban cities: a mixed-method study. BMC Health Serv Res. 2023;23(1):25.
10. Moradi F, Balaghi Z, Joulaei H, Zare N, Mohammadi S, Moghadami M. Unmet Need for Prevention of Unwanted Pregnancy in Shiraz. 2014;
11. Pregnancy among Women on ART in Debre Markos Referral Hospital, Northwest Ethiopia: A Five-Year Retrospective Cohort Study. 2017;
12. Yam EA, Kidanu A, Burnett‐Zieman B, Pilgrim N, Okal J, Bekele A, et al. Pregnancy experiences of female sex workers in Adama City, Ethiopia: Complexity of partner relationships and pregnancy intentions. Stud Fam Plann. 2017;48(2):107–19.
13. Bafana TNS. Factors influencing contraceptive use and unplanned pregnancy in a South African population. 2010.
14. Mbongueh MC, Nicholas T, Ndum AC, Gisèle EL, Nguedia A, Clement J. Unintended pregnancy and sexually transmissible infections amongst adolescents and young adults in Douala IV municipality, Cameroon: Prevalence, knowledge, and associated factors. 2023;
15. Dibaba Y, Fantahun M, Hindin MJ. The association of unwanted pregnancy and social support with depressive symptoms in pregnancy: evidence from rural Southwestern Ethiopia. BMC Pregnancy Childbirth. 2013;13:1–8.
16. Raifman J, Chetty T, Tanser F, Mutevedzi T, Matthews P, Herbst K, et al. Preventing unintended pregnancy and HIV transmission: effects of the HIV treatment cascade on contraceptive use and choice in rural KwaZulu-Natal. JAIDS J Acquir Immune Defic Syndr. 2014;67:S218–27.
17. Akinyi MB, Momanyi LB, Murangiri GJ, Mutei MJ. Incidences of Pregnancy During Concurrent Use of Hormonal Contraceptives and Antiretroviral Therapy at Nakuru Khan MN, Harris ML, Shifti DM, Laar AS, Loxton D. Effects of unintended pregnancy on maternal healthcare services utilization in low-and lower-middle-income countries: systematic review and meta-analysis. Int J Public Health. 2019;64:743–54.
18. County Referral and Teaching Hospital in Kenya. African J Pharm Altern Med. 2023;2(1):32–46.
19. Erena MG, Kerbo AA. Unwanted pregnancy and associated factors among female students of Madawalabu University Bale Zone, Oromia Region south east, Ethiopia. Sci J Public Heal. 2015;3(1):50–5.
20. Bankole A, Singh S, Hussain R, Oestreicher G. Condom use for preventing STI/HIV and unintended pregnancy among young men in Sub-Saharan Africa. Am J Mens Health. 2009;3(1):60–78.
21. Singh S, Bankole A, Woog V. Evaluating the need for sex education in developing countries: sexual behaviour, knowledge of preventing sexually transmitted infections/HIV and unplanned pregnancy. Sex Educ. 2005;5(4):307–31.
22. Magadi MA, Agwanda AO. Determinants of transitions to first sexual intercourse, marriage and pregnancy among female adolescents: evidence from South Nyanza, Kenya. J Biosoc Sci. 2009;41(3):409–27.
23. Ahinkorah BO, Seidu A-A, Appiah F, Oduro JK, Sambah F, Baatiema L, et al. Effect of sexual violence on planned, mistimed and unwanted pregnancies among women of reproductive age in sub-Saharan Africa: A multi-country analysis of Demographic and Health Surveys. SSM-population Heal. 2020;11:100601.
24. Ma Q, Ono-Kihara M, Cong L, Xu G, Pan X, Zamani S, et al. Unintended pregnancy and its risk factors among university students in eastern China. Contraception. 2008;77(2):108–13.
25. Tumusiigirwe K. Factors associated with unwanted pregnancies among girls aged 15 to 19 years in Kakoba Division in Mbarara District. 2017;
26. Arega T. HIV and Unintended Pregnancy Risk Perception and Contraceptive Use among Youth in Debre Birhan District, Ethiopia. Addis Ababa University; 2010.
27. Alene KA, Atalell KA. Contraceptive use and method preference among HIV-positive women in Amhara region, Ethiopia. BMC Womens Health. 2018;18:1–9.
28. Doherty K, Arena K, Wynn A, Offorjebe OA, Moshashane N, Sickboy O, et al. Unintended pregnancy in Gaborone, Botswana: A cross sectional study. Afr J Reprod Health. 2018;22(2):76–82. Yam EA, Kidanu A, Burnett‐Zieman B, Pilgrim N, Okal J, Bekele A, et al. Pregnancy experiences of female sex workers in Adama City, Ethiopia: Complexity of partner relationships and pregnancy intentions. Stud Fam Plann. 2017;48(2):107–19.
29. Ferede TA, Muluneh AG, Wagnew A, Walle AD. Prevalence and associated factors of early sexual initiation among youth female in sub-Saharan Africa: a multilevel analysis of recent demographic and health surveys. BMC Womens Health. 2023;23(1):147.
30. Bafana TNS. Factors influencing contraceptive use and unplanned pregnancy in a South African population. 2010.
31. Mbongueh MC, Nicholas T, Ndum AC, Gisèle EL, Nguedia A, Clement J. Unintended pregnancy and sexually transmissible infections amongst adolescents and young adults in Douala IV municipality, Cameroon: Prevalence, knowledge, and associated factors. 2023;
32. Bakibinga P, Matanda DJ, Ayiko R, Rujumba J, Muiruri C, Amendah D, et al. Pregnancy history and current use of contraception among women of reproductive age in Burundi, Kenya, Rwanda, Tanzania and Uganda: analysis of demographic and health survey data. BMJ Open. 2016;6(3):e009991.
33. ADENUGA FA. CONTRACEPTIVE USE AMONG WOMEN LIVING WITH HIV AND AIDS RECEIVING CARE AT SECONDARY AND TERTIARY HEALTH FACILITIES IN IBADAN NIGERIA. 2016.
34. Peel J, de Gersigny JB, Teague R, Howard J, Bradshaw C, Chen M, et al. Reproductive health among women living with HIV attending Melbourne Sexual Health Centre for HIV care from February 2019 to February 2020. Sex Health. 2024;21(1):NULL-NULL.
35. Newmann SJ, Zakaras JM, Tao AR, Onono M, Bukusi EA, Cohen CR, et al. Integrating family planning into HIV care in western Kenya: HIV care providers’ perspectives and experiences one year following integration. AIDS Care. 2016;28(2):209–13.
36. Abebe EC, Ayele TM, Dejenie TA, Muche ZT. Assessment of Modern Contraceptive Utilization and Associated Factors Among Women Living With HIV/AIDS In DTRH In Debre Tabor Town, South Gondar Zone, Ethiopia. 2021;
37. MacCarthy S, Rasanathan JJK, Ferguson L, Gruskin S. The pregnancy decisions of HIV-positive women: the state of knowledge and way forward. Reprod Health Matters. 2012;20(sup39):119–40.
38. Khu NH, Vwalika B, Karita E, Kilembe W, Bayingana RA, Sitrin D, et al. Fertility goal-based counseling increases contraceptive implant and IUD use in HIV-discordant couples in Rwanda and Zambia. Contraception. 2013;88(1):74–82.
39. Haile D, Lagebo B. Magnitude of dual contraceptive method utilization and the associated factors among women on antiretroviral treatment in Wolaita zone, Southern Ethiopia. Heliyon. 2022;8(6).
40. Maharaj P. The dual risks of unwanted pregnancy and HIV/AIDS: the case of KwaZulu-Natal, South Africa. London School of Hygiene & Tropical Medicine; 2003.
41. Ma Q, Ono-Kihara M, Cong L, Xu G, Pan X, Zamani S, et al. Unintended pregnancy and its risk factors among university students in eastern China. Contraception. 2008;77(2):108–13.
42. Bakari HM, Alo O, Mbwana MS, Salim SM, Ludeman E, Lascko T, et al. Prevalence of unmet need for family planning and unintended pregnancies among women of reproductive age living with HIV in sub-Saharan Africa: a systematic review and meta-analysis. Afr Health Sci. 2024;24(2):41–53.
43. Greene S, Ion A, Kwaramba G, Smith S, Loutfy MR. “Why are you pregnant? What were you thinking?”: How women navigate experiences of HIV-related stigma in medical settings during pregnancy and birth. Soc Work Health Care. 2016;55(2):161–79.
44. Okereke CI. Unmet reproductive health needs and health-seeking behaviour of adolescents in Owerri, Nigeria. Afr J Reprod Health. 2010;14(1).
45. Mshweshwe-Pakela NT, Matlakala MC, Mbengo F. Attitudes to, and knowledge and use of contraception among female learners attending a high school in Mdantsane. Afr J Nurs Midwifery. 2017;19(1):170–89.
46. Omoro T, Gray SC, Otieno G, Mbeda C, Phillips-Howard PA, Hayes T, et al. Teen pregnancy in rural western Kenya: a public health issue. Int J Adolesc Youth. 2018;23(4):399–408.
47. Hale F, Vazquez M. Violence against women living with HIV/AIDS: A background paper. Washingt DC Dev Connect. 2011;
48. Moradi F, Balaghi Z, Joulaei H, Zare N, Mohammadi S, Moghadami M. Unmet Need for Prevention of Unwanted Pregnancy in Shiraz. 2014;
49. Tessema ZT, Teshale AB, Tesema GA, Tamirat KS. Determinants of completing recommended antenatal care utilization in sub-Saharan from 2006 to 2018: evidence from 36 countries using Demographic and Health Surveys. BMC Pregnancy Childbirth. 2021;21:1–12.
50. Obiyan MO, Olaleye AO, Oyinlola FF, Folayan MO. Factors associated with pregnancy and induced abortion among street-involved female adolescents in two Nigeria urban cities: a mixed-method study. BMC Health Serv Res. 2023;23(1):25.
51. Musyimi CW, Mutiso VN, Nyamai DN, Ebuenyi I, Ndetei DM. Suicidal behavior risks during adolescent pregnancy in a low-resource setting: A qualitative study. PLoS One. 2020;15(7):e0236269.
52. Tadesse G, Yakob B. Risky sexual behaviors among female youth in Tiss Abay, a semi-urban area of the Amhara Region, Ethiopia. PLoS One. 2015;10(3):e0119050.
53. Adilo TM, Wordofa HM. Prevalence of fertility desire and its associated factors among 15-to 49-year-old people living with HIV/AIDS in Addis Ababa, Ethiopia: a cross-sectional study design. HIV/AIDS-Research Palliat Care. 2017;167–76.
54. Ashimi AO, Amole TG, Abubakar MY, Ugwa EA. Fertility desire and utilization of family planning methods among HIV‑positive women attending a tertiary hospital in a suburban setting in Northern Nigeria. Trop J Obstet Gynaecol. 2017;34(1):54–60.
55. Selke HM, Kimaiyo S, Sidle JE, Vedanthan R, Tierney WM, Shen C, et al. Task-shifting of antiretroviral delivery from health care workers to persons living with HIV/AIDS: clinical outcomes of a community-based program in Kenya. JAIDS J Acquir Immune Defic Syndr. 2010;55(4):483–90.
56. Druce N, Nolan A. Seizing the big missed opportunity: linking HIV and maternity care services in sub-Saharan Africa. Reprod Health Matters. 2007;15(30):190–201.
57. Nyanja TAN, Tulinius C. Relationships matter: contraceptive choices among HIV-positive women in Tanzania. African J AIDS Res. 2017;16(2):109–17.
58. Johnson LF, Mutemaringa T, Heekes A, Boulle A. Effect of HIV infection and antiretroviral treatment on pregnancy rates in the Western Cape province of South Africa. J Infect Dis. 2020;221(12):1953–62.
59. Nakku‐Joloba E, Pisarski EE, Wyatt MA, Muwonge TR, Asiimwe S, Celum CL, et al. Beyond HIV prevention: everyday life priorities and demand for PrEP among Ugandan HIV serodiscordant couples. African J Reprod Gynaecol Endosc. 2019;22(1).
60. Maharaj P. The dual risks of unwanted pregnancy and HIV/AIDS: the case of KwaZulu-Natal, South Africa. London School of Hygiene & Tropical Medicine; 2003.
61. Ajayi AI, Ezegbe HC. Association between sexual violence and unintended pregnancy among adolescent girls and young women in South Africa. BMC Public Health. 2020;20(1):1370.
62. Chukwunyere AP, Stella KA. Unintended pregnancy among undergraduate students at a select university, Eastern Cape, South Africa: effects, influences, outcomes and solutions. Gend Behav. 2019;17(4):14272–86.
63. King R, Khana K, Nakayiwa S, Katuntu D, Homsy J, Lindkvist P, et al. “Pregnancy comes accidentally-like it did with me”: reproductive decisions among women on ART and their partners in rural Uganda. BMC Public Health. 2011;11:1–11.
64. Bradley H, Tsui A, Kidanu A, Gillespie D. HIV infection and contraceptive need among female Ethiopian voluntary HIV counseling and testing clients. AIDS Care. 2010;22(10):1295–304.
65. Abubeker FA, Fanta MB, Dalton VK. Unmet Need for Contraception among HIV‐Positive Women Attending HIV Care and Treatment Service at Saint Paul’s Hospital Millennium Medical College, Addis Ababa, Ethiopia. Int J Reprod Med. 2019;2019(1):3276780.
66. Chanda P, JO EK, Ochieng LA. FACTORS AFFECTING UPTAKE OF CONTRACEPTIVES AMONG WOMEN AGED 15-25 IN THE CONTEXT OF EARLY PREGNANCY AND HIV/AIDS PREVENTION IN UGANDA.
67. Suryavanshi N, Erande A, Pisal H, Shankar A V, Bhosale RA, Bollinger RC, et al. Repeated pregnancy among women with known HIV status in Pune, India. AIDS Care. 2008;20(9):1111–8.
68. Levandowski BA, Kalilani‐Phiri L, Kachale F, Awah P, Kangaude G, Mhango C. Investigating social consequences of unwanted pregnancy and unsafe abortion in Malawi: the role of stigma. Int J Gynecol Obstet. 2012;118:S167–71.
69. MONEM AA. UNINTENDED PREGNANCIES IN THE MIDDLE EAST AND NORTH AFRICA. 2010;
70. Willard Cates JR, Steiner MJ. Dual protection against unintended pregnancy and sexually transmitted infections: what is the best contraceptive approach? Sex Transm Dis. 2002;29(3):168–74.
71. Kyaw KWY, Mon AA, Phyo KH, Kyaw NTT, Kumar AM V, Lwin TT, et al. Initiation of antiretroviral therapy or antiretroviral prophylaxis in pregnant women living with HIV registered in five townships of Mandalay, Myanmar: A cross sectional study. BMC Pregnancy Childbirth. 2019;19:1–9.
72. Nzioka C. Perspectives of adolescent boys on the risks of unwanted pregnancy and sexually transmitted infections: Kenya. Reprod Health Matters. 2001;9(17):108–17.
73. Eyakuze C, Jones DA, Starrs AM, Sorkin N. From PMTCT to a more comprehensive AIDS response for women: a much‐needed shift. Dev World Bioeth. 2008;8(1):33–42.
74. Onyeka IN, Miettola J, Vaskilampi T, Ilika AL. Unintended pregnancy and termination of studies among students in Anambra state, Nigeria: Are secondary schools playing their part? Afr J Reprod Health. 2011;15(2):109–15.
75. Mutiso SM, Kinuthia J, Qureshi Z. Contraceptive use among HIV infected women attending Comprehensive Care Centre. East Afr Med J. 2008;85(4):171–7.
76. Pallitto CC, O’Campo P. The relationship between intimate partner violence and unintended pregnancy: analysis of a national sample from Colombia. Int Fam Plan Perspect. 2004;165–73.
77. Tibebu NS, Kassie BA, Anteneh TA, Rade BK. Depression, anxiety and stress among HIV-positive pregnant women in Ethiopia during the COVID-19 pandemic. Trans R Soc Trop Med Hyg. 2023;117(5):317–25.
78. Bouris A, Guilamo-Ramos V, Jaccard J, McCoy W, Aranda D, Pickard A, et al. The feasibility of a clinic-based parent intervention to prevent HIV, sexually transmitted infections, and unintended pregnancies among Latino and African American adolescents. AIDS Patient Care STDS. 2010;24(6):381–7.
79. Bakari HM, Alo O, Mbwana MS, Salim SM, Ludeman E, Lascko T, et al. Prevalence of unmet need for family planning and unintended pregnancies among women of reproductive age living with HIV in sub-Saharan Africa: a systematic review and meta-analysis. Afr Health Sci. 2024;24(2):41–53.
80. Atukunda EC, Mugyenyi GR, Atuhumuza EB, Kaida A, Boatin A, Agaba AG, et al. Factors associated with pregnancy intentions amongst postpartum women living with HIV in rural Southwestern Uganda. AIDS Behav. 2019;23:1552–60.
81. Wall KM, Haddad L, Vwalika B, Htee Khu N, Brill I, Kilembe W, et al. Unintended pregnancy among HIV positive couples receiving integrated HIV counseling, testing, and family planning services in Zambia. PLoS One. 2013;8(9):e75353.
82. Namukisa M, Kamacooko O, Lunkuse JF, Ruzagira E, Price MA, Mayanja Y. Incidence of unintended pregnancy and associated factors among adolescent girls and young women at risk of HIV infection in Kampala, Uganda. Front Reprod Heal. 2023;5:1089104.
83. Amongi PR. Factors Associated With Unintended Pregnancy Among Hiv Positive Women On Anti Retroviral Therapy In Gulu District. CIU; 2018.
84. Mwalye PJ. Impact of Unintended pregnancy on HIV viral load outcomes among postpartum women living with HIV in Cape Town, South Africa: clues from postpartum adherence clubs for antiretroviral therapy trial. 2022;
85. Bain LE, Zweekhorst MBM, de Cock Buning T. Prevalence and determinants of unintended pregnancy in sub–saharan Africa: a systematic review. Afr J Reprod Health. 2020;24(2):187–205.
86. Fotso JC, Izugbara C, Saliku T, Ochako R. Unintended pregnancy and subsequent use of modern contraceptive among slum and non-slum women in Nairobi, Kenya. BMC Pregnancy Childbirth. 2014;14:1–10.
87. Duff P, Muzaaya G, Muldoon K, Dobrer S, Akello M, Birungi J, et al. High rates of unintended pregnancies among young women sex Workers in Conflict-affected Northern Uganda: the social contexts of brothels/lodges and substance use. Afr J Reprod Health. 2017;21(2):64–72.
88. Bankole A, Singh S, Hussain R, Oestreicher G. Condom use for preventing STI/HIV and unintended pregnancy among young men in Sub-Saharan Africa. Am J Mens Health. 2009;3(1):60–78.
89. Dhakal S, Song JS, Shin DE, Lee TH, So AY, Nam EW. Unintended pregnancy and its correlates among currently pregnant women in the Kwango District, Democratic Republic of the Congo. Reprod Health. 2016;13:1–7.
90. Izugbara C, Egesa C. The management of unwanted pregnancy among women in Nairobi, Kenya. Int J Sex Heal. 2014;26(2):100–12.
91. Wall KM, Kilembe W, Vwalika B, Haddad LB, Khu NH, Brill I, et al. Optimizing prevention of HIV and unplanned pregnancy in discordant African couples. J women’s Heal. 2017;26(8):900–10.
92. Aragaw FM, Amare T, Teklu RE, Tegegne BA, Alem AZ. Magnitude of unintended pregnancy and its determinants among childbearing age women in low and middle-income countries: evidence from 61 low and middle income countries. Front Reprod Heal. 2023;5:1113926.
93. Tusiime S, Musinguzi G, Tinkitina B, Mwebaza N, Kisa R, Anguzu R, et al. Prevalence of sexual coercion and its association with unwanted pregnancies among young pregnant females in Kampala, Uganda: a facility based cross-sectional study. BMC Womens Health. 2015;15:1–12.
94. Teklu T, Davey G. Which factors influence North Ethiopian adults’ use of dual protection from unintended pregnancy and HIV/AIDS? Ethiop J Heal Dev. 2008;22(3).
95. Chukwunyere AP, Stella KA. Unintended pregnancy among undergraduate students at a select university, Eastern Cape, South Africa: effects, influences, outcomes and solutions. Gend Behav. 2019;17(4):14272–86. 1.
96. Ahinkorah BO, Seidu A-A, Appiah F, Oduro JK, Sambah F, Baatiema L, et al. Effect of sexual violence on planned, mistimed and unwanted pregnancies among women of reproductive age in sub-Saharan Africa: A multi-country analysis of Demographic and Health Surveys. SSM-population Heal. 2020;11:100601.
97. Kaida A, Matthews LT, Kanters S, Kabakyenga J, Muzoora C, Mocello AR, et al. Incidence and predictors of pregnancy among a cohort of HIV-positive women initiating antiretroviral therapy in Mbarara, Uganda. PLoS One. 2013;8(5):e63411.
98. Weldegebreal R, Melaku YA, Alemayehu M, Gebrehiwot TG. Unintended pregnancy among female sex workers in Mekelle city, northern Ethiopia: a cross-sectional study. BMC Public Health. 2015;15:1–9.
99. Erena MG, Kerbo AA. Unwanted pregnancy and associated factors among female students of Madawalabu University Bale Zone, Oromia Region south east, Ethiopia. Sci J Public Heal. 2015;3(1):50–5.
100. Wall KM, Kilembe W, Vwalika B, Haddad LB, Khu NH, Brill I, et al. Optimizing prevention of HIV and unplanned pregnancy in discordant African couples. J women’s Heal. 2017;26(8):900–10.
101. Doherty K, Arena K, Wynn A, Offorjebe OA, Moshashane N, Sickboy O, et al. Unintended pregnancy in Gaborone, Botswana: A cross sectional study. Afr J Reprod Health. 2018;22(2):76–82.
102. Amare T, Tessema F, Shaweno T. Trend of Unintended Pregnancy, Induced Abortion and Associated Factors among Adolescents in Ethiopia: Evidence from the 2000, 2005, 2011 and 2016 EDHS Data. 2022;
103. Abdullahi IS, Chukwudike CO, Sangari JS, Chikwendu JI, Fulani GJ. The causes of unwanted pregnancy and abortion among female students and its impact on their academic performance in FCE Pankshin, Plateau state, Nigeria.
104. Fotso JC, Izugbara C, Saliku T, Ochako R. Unintended pregnancy and subsequent use of modern contraceptive among slum and non-slum women in Nairobi, Kenya. BMC Pregnancy Childbirth. 2014;14:1–10.
105. Izugbara C, Egesa C. The management of unwanted pregnancy among women in Nairobi, Kenya. Int J Sex Heal. 2014;26(2):100–12.
106. Nzioka C. Unwanted pregnancy and sexually transmitted infection among young women in rural Kenya. Cult Health Sex. 2004;6(1):31–44.
107. Mamboleo N. Unwanted pregnancy and induced abortion among female youths: a case study of Temeke district. Muhimbili University of Health and Allied Sciences; 2012.
108. Ingabire R, Parker R, Nyombayire J, Ko JE, Mukamuyango J, Bizimana J, et al. Female sex workers in Kigali, Rwanda: a key population at risk of HIV, sexually transmitted infections, and unplanned pregnancy. Int J STD AIDS. 2019;30(6):557–68.
109. Maharaj P. The dual risks of unwanted pregnancy and HIV/AIDS: the case of KwaZulu-Natal, South Africa. London School of Hygiene & Tropical Medicine; 2003.
110. Moradi F, Balaghi Z, Joulaei H, Zare N, Mohammadi S, Moghadami M. Unmet Need for Prevention of Unwanted Pregnancy in Shiraz. 2014;
