# Supplementary material for: Unintended pregnancy among women living with HIV and its predictors in East Africa, 2024. A systematic review and meta-analysis
Source: PLoS One. 2024 Dec 27;19(12):e0310212. doi: 10.1371/journal.pone.0310212 (PMC11676498; doi:10.1371/journal.pone.0310212)
Supplement: S3 File — (ZIP) [file pone.0310212.s003.zip › Excluded studies because the outcome of interest not reported.docx]

***Excluded studies because the outcome of interest not reported***

1. King R, Khana K, Nakayiwa S, Katuntu D, Homsy J, Lindkvist P, et al. “Pregnancy comes accidentally-like it did with me”: reproductive decisions among women on ART and their partners in rural Uganda. BMC Public Health. 2011;11:1–11.
2. Bradley H, Tsui A, Kidanu A, Gillespie D. HIV infection and contraceptive need among female Ethiopian voluntary HIV counseling and testing clients. AIDS Care. 2010;22(10):1295–304.
3. Abubeker FA, Fanta MB, Dalton VK. Unmet Need for Contraception among HIV‐Positive Women Attending HIV Care and Treatment Service at Saint Paul’s Hospital Millennium Medical College, Addis Ababa, Ethiopia. Int J Reprod Med. 2019;2019(1):3276780.
4. Chanda P, JO EK, Ochieng LA. FACTORS AFFECTING UPTAKE OF CONTRACEPTIVES AMONG WOMEN AGED 15-25 IN THE CONTEXT OF EARLY PREGNANCY AND HIV/AIDS PREVENTION IN UGANDA.
5. Suryavanshi N, Erande A, Pisal H, Shankar A V, Bhosale RA, Bollinger RC, et al. Repeated pregnancy among women with known HIV status in Pune, India. AIDS Care. 2008;20(9):1111–8.
6. Levandowski BA, Kalilani‐Phiri L, Kachale F, Awah P, Kangaude G, Mhango C. Investigating social consequences of unwanted pregnancy and unsafe abortion in Malawi: the role of stigma. Int J Gynecol Obstet. 2012;118:S167–71.
7. MONEM AA. UNINTENDED PREGNANCIES IN THE MIDDLE EAST AND NORTH AFRICA. 2010;
8. Willard Cates JR, Steiner MJ. Dual protection against unintended pregnancy and sexually transmitted infections: what is the best contraceptive approach? Sex Transm Dis. 2002;29(3):168–74.
9. Kyaw KWY, Mon AA, Phyo KH, Kyaw NTT, Kumar AM V, Lwin TT, et al. Initiation of antiretroviral therapy or antiretroviral prophylaxis in pregnant women living with HIV registered in five townships of Mandalay, Myanmar: A cross sectional study. BMC Pregnancy Childbirth. 2019;19:1–9.
10. Nzioka C. Perspectives of adolescent boys on the risks of unwanted pregnancy and sexually transmitted infections: Kenya. Reprod Health Matters. 2001;9(17):108–17.
11. Eyakuze C, Jones DA, Starrs AM, Sorkin N. From PMTCT to a more comprehensive AIDS response for women: a much‐needed shift. Dev World Bioeth. 2008;8(1):33–42.
12. Onyeka IN, Miettola J, Vaskilampi T, Ilika AL. Unintended pregnancy and termination of studies among students in Anambra state, Nigeria: Are secondary schools playing their part? Afr J Reprod Health. 2011;15(2):109–15.
13. Mutiso SM, Kinuthia J, Qureshi Z. Contraceptive use among HIV infected women attending Comprehensive Care Centre. East Afr Med J. 2008;85(4):171–7.
14. Pallitto CC, O’Campo P. The relationship between intimate partner violence and unintended pregnancy: analysis of a national sample from Colombia. Int Fam Plan Perspect. 2004;165–73.
15. Tibebu NS, Kassie BA, Anteneh TA, Rade BK. Depression, anxiety and stress among HIV-positive pregnant women in Ethiopia during the COVID-19 pandemic. Trans R Soc Trop Med Hyg. 2023;117(5):317–25.
16. Bouris A, Guilamo-Ramos V, Jaccard J, McCoy W, Aranda D, Pickard A, et al. The feasibility of a clinic-based parent intervention to prevent HIV, sexually transmitted infections, and unintended pregnancies among Latino and African American adolescents. AIDS Patient Care STDS. 2010;24(6):381–7.
17. Bakari HM, Alo O, Mbwana MS, Salim SM, Ludeman E, Lascko T, et al. Prevalence of unmet need for family planning and unintended pregnancies among women of reproductive age living with HIV in sub-Saharan Africa: a systematic review and meta-analysis. Afr Health Sci. 2024;24(2):41–53.
18. Atukunda EC, Mugyenyi GR, Atuhumuza EB, Kaida A, Boatin A, Agaba AG, et al. Factors associated with pregnancy intentions amongst postpartum women living with HIV in rural Southwestern Uganda. AIDS Behav. 2019;23:1552–60.
19. Wall KM, Haddad L, Vwalika B, Htee Khu N, Brill I, Kilembe W, et al. Unintended pregnancy among HIV positive couples receiving integrated HIV counseling, testing, and family planning services in Zambia. PLoS One. 2013;8(9):e75353.
20. Namukisa M, Kamacooko O, Lunkuse JF, Ruzagira E, Price MA, Mayanja Y. Incidence of unintended pregnancy and associated factors among adolescent girls and young women at risk of HIV infection in Kampala, Uganda. Front Reprod Heal. 2023;5:1089104.
21. Amongi PR. Factors Associated With Unintended Pregnancy Among Hiv Positive Women On Anti Retroviral Therapy In Gulu District. CIU; 2018.
22. Mwalye PJ. Impact of Unintended pregnancy on HIV viral load outcomes among postpartum women living with HIV in Cape Town, South Africa: clues from postpartum adherence clubs for antiretroviral therapy trial. 2022;
23. Bain LE, Zweekhorst MBM, de Cock Buning T. Prevalence and determinants of unintended pregnancy in sub–saharan Africa: a systematic review. Afr J Reprod Health. 2020;24(2):187–205.
24. Fotso JC, Izugbara C, Saliku T, Ochako R. Unintended pregnancy and subsequent use of modern contraceptive among slum and non-slum women in Nairobi, Kenya. BMC Pregnancy Childbirth. 2014;14:1–10.
25. Duff P, Muzaaya G, Muldoon K, Dobrer S, Akello M, Birungi J, et al. High rates of unintended pregnancies among young women sex Workers in Conflict-affected Northern Uganda: the social contexts of brothels/lodges and substance use. Afr J Reprod Health. 2017;21(2):64–72.
26. Bankole A, Singh S, Hussain R, Oestreicher G. Condom use for preventing STI/HIV and unintended pregnancy among young men in Sub-Saharan Africa. Am J Mens Health. 2009;3(1):60–78.
27. Dhakal S, Song JS, Shin DE, Lee TH, So AY, Nam EW. Unintended pregnancy and its correlates among currently pregnant women in the Kwango District, Democratic Republic of the Congo. Reprod Health. 2016;13:1–7.
28. Izugbara C, Egesa C. The management of unwanted pregnancy among women in Nairobi, Kenya. Int J Sex Heal. 2014;26(2):100–12.
29. Wall KM, Kilembe W, Vwalika B, Haddad LB, Khu NH, Brill I, et al. Optimizing prevention of HIV and unplanned pregnancy in discordant African couples. J women’s Heal. 2017;26(8):900–10.
30. Aragaw FM, Amare T, Teklu RE, Tegegne BA, Alem AZ. Magnitude of unintended pregnancy and its determinants among childbearing age women in low and middle-income countries: evidence from 61 low and middle income countries. Front Reprod Heal. 2023;5:1113926.
31. Tusiime S, Musinguzi G, Tinkitina B, Mwebaza N, Kisa R, Anguzu R, et al. Prevalence of sexual coercion and its association with unwanted pregnancies among young pregnant females in Kampala, Uganda: a facility based cross-sectional study. BMC Womens Health. 2015;15:1–12.
32. Teklu T, Davey G. Which factors influence North Ethiopian adults’ use of dual protection from unintended pregnancy and HIV/AIDS? Ethiop J Heal Dev. 2008;22(3).
33. Chukwunyere AP, Stella KA. Unintended pregnancy among undergraduate students at a select university, Eastern Cape, South Africa: effects, influences, outcomes and solutions. Gend Behav. 2019;17(4):14272–86. 1.
34. Ahinkorah BO, Seidu A-A, Appiah F, Oduro JK, Sambah F, Baatiema L, et al. Effect of sexual violence on planned, mistimed and unwanted pregnancies among women of reproductive age in sub-Saharan Africa: A multi-country analysis of Demographic and Health Surveys. SSM-population Heal. 2020;11:100601.
35. Kaida A, Matthews LT, Kanters S, Kabakyenga J, Muzoora C, Mocello AR, et al. Incidence and predictors of pregnancy among a cohort of HIV-positive women initiating antiretroviral therapy in Mbarara, Uganda. PLoS One. 2013;8(5):e63411.
36. Weldegebreal R, Melaku YA, Alemayehu M, Gebrehiwot TG. Unintended pregnancy among female sex workers in Mekelle city, northern Ethiopia: a cross-sectional study. BMC Public Health. 2015;15:1–9.
37. Erena MG, Kerbo AA. Unwanted pregnancy and associated factors among female students of Madawalabu University Bale Zone, Oromia Region south east, Ethiopia. Sci J Public Heal. 2015;3(1):50–5.
38. Wall KM, Kilembe W, Vwalika B, Haddad LB, Khu NH, Brill I, et al. Optimizing prevention of HIV and unplanned pregnancy in discordant African couples. J women’s Heal. 2017;26(8):900–10.
39. Doherty K, Arena K, Wynn A, Offorjebe OA, Moshashane N, Sickboy O, et al. Unintended pregnancy in Gaborone, Botswana: A cross sectional study. Afr J Reprod Health. 2018;22(2):76–82.
40. Amare T, Tessema F, Shaweno T. Trend of Unintended Pregnancy, Induced Abortion and Associated Factors among Adolescents in Ethiopia: Evidence from the 2000, 2005, 2011 and 2016 EDHS Data. 2022;
41. Abdullahi IS, Chukwudike CO, Sangari JS, Chikwendu JI, Fulani GJ. The causes of unwanted pregnancy and abortion among female students and its impact on their academic performance in FCE Pankshin, Plateau state, Nigeria.
42. Fotso JC, Izugbara C, Saliku T, Ochako R. Unintended pregnancy and subsequent use of modern contraceptive among slum and non-slum women in Nairobi, Kenya. BMC Pregnancy Childbirth. 2014;14:1–10.
43. Izugbara C, Egesa C. The management of unwanted pregnancy among women in Nairobi, Kenya. Int J Sex Heal. 2014;26(2):100–12.
44. Nzioka C. Unwanted pregnancy and sexually transmitted infection among young women in rural Kenya. Cult Health Sex. 2004;6(1):31–44.
45. Mamboleo N. Unwanted pregnancy and induced abortion among female youths: a case study of Temeke district. Muhimbili University of Health and Allied Sciences; 2012.
46. Ingabire R, Parker R, Nyombayire J, Ko JE, Mukamuyango J, Bizimana J, et al. Female sex workers in Kigali, Rwanda: a key population at risk of HIV, sexually transmitted infections, and unplanned pregnancy. Int J STD AIDS. 2019;30(6):557–68.
47. Maharaj P. The dual risks of unwanted pregnancy and HIV/AIDS: the case of KwaZulu-Natal, South Africa. London School of Hygiene & Tropical Medicine; 2003.
48. Moradi F, Balaghi Z, Joulaei H, Zare N, Mohammadi S, Moghadami M. Unmet Need for Prevention of Unwanted Pregnancy in Shiraz. 2014;
