# Supplementary material for: Unintended pregnancy among women living with HIV and its predictors in East Africa, 2024. A systematic review and meta-analysis
Source: PLoS One. 2024 Dec 27;19(12):e0310212. doi: 10.1371/journal.pone.0310212 (PMC11676498; doi:10.1371/journal.pone.0310212)
Supplement: S3 File — (ZIP) [file pone.0310212.s003.zip › Excluded studies by reading the titles.docx]

***Excluded studies by titles***

1. Lunani LL, Abaasa A, Omosa-Manyonyi G. Prevalence and factors associated with contraceptive use among Kenyan women aged 15–49 years. AIDS Behav. 2018;22:125–30.
2. Wilcher R, Cates W. Reaching the underserved: family planning for women with HIV. Stud Fam Plann. 2010;41(2):125–8.
3. Colombini M, Mayhew SH, Mutemwa R, Kivunaga J, Ndwiga C, Team I. Perceptions and experiences of integrated service delivery among women living with HIV attending reproductive health services in Kenya: a mixed methods study. AIDS Behav. 2016;20:2130–40.
4. Skerritt L, Kaida A, O’Brien N, Burchell AN, Bartlett G, Savoie É, et al. Patterns of Magadi MA, Agwanda AO. Determinants of transitions to first sexual intercourse, marriage and pregnancy among female adolescents: evidence from South Nyanza, Kenya. J Biosoc Sci. 2009;41(3):409–27.
5. Omollo C. Determinants of contraceptives preference and use among people living with hiv and aids in rural areas: a study of Nyamarambe division, Kisii county, Kenya. 2021.
6. Kebede YB, Geremew TT, Mehretie Y, Abejie AN, Bewket L, Dellie E. Associated factors of modern contraceptive use among women infected with human immunodeficiency virus in Enemay District, Northwest Ethiopia: a facility-based cross-sectional study. BMC Public Health. 2019;19:1–11.
7. Tumusiigirwe K. Factors associated with unwanted pregnancies among girls aged 15 to 19 years in Kakoba Division in Mbarara District. 2017;
8. Kamangu AA, Myeya HE. Exploring Young Peoples’ Sexual Behaviours and the Underlying Factors in East Africa: A Review of Literature from Four Countries. J Anthropol Surv India. 2023;72(1):149–62.
9. De Bruyn M. Women, reproductive rights, and HIV/AIDS: Issues on which research and interventions are still needed. J Health Popul Nutr. 2006;24(4):413.
10. Abubeker FA, Fanta MB, Dalton VK. Unmet Need for Contraception among HIV‐Positive Women Attending HIV Care and Treatment Service at Saint Paul’s Hospital Millennium Medical College, Addis Ababa, Ethiopia. Int J Reprod Med. 2019;2019(1):3276780.
11. Ma Q, Ono-Kihara M, Cong L, Xu G, Pan X, Zamani S, et al. Early initiation of sexual activity: a risk factor for sexually transmitted diseases, HIV infection, and unwanted pregnancy among university students in China. BMC Public Health. 2009;9:1–8.
12. Shikhansari S, Khalesi ZB, Rad EH. Factors associated with the reproductive health of women living with HIV in Iran. Eur J Obstet Gynecol Reprod Biol X. 2022;13:100136.
13. Nyanja TAN, Tulinius C. Relationships matter: contraceptive choices among HIV-positive women in Tanzania. African J AIDS Res. 2017;16(2):109–17.
14. Johnson LF, Mutemaringa T, Heekes A, Boulle A. Effect of HIV infection and antiretroviral treatment on pregnancy rates in the Western Cape province of South Africa. J Infect Dis. 2020;221(12):1953–62.
15. Nakku‐Joloba E, Pisarski EE, Wyatt MA, Muwonge TR, Asiimwe S, Celum CL, et al. Beyond HIV prevention: everyday life priorities and demand for PrEP among Ugandan HIV serodiscordant couples. African J Reprod Gynaecol Endosc. 2019;22(1).
16. Maharaj P. The dual risks of unwanted pregnancy and HIV/AIDS: the case of KwaZulu-Natal, South Africa. London School of Hygiene & Tropical Medicine; 2003.
17. Adilo TM, Wordofa HM. Prevalence of fertility desire and its associated factors among 15-to 49-year-old people living with HIV/AIDS in Addis Ababa, Ethiopia: a cross-sectional study design. HIV/AIDS-Research Palliat Care. 2017;167–76.
18. Ashimi AO, Amole TG, Abubakar MY, Ugwa EA. Fertility desire and utilization of family planning methods among HIV‑positive women attending a tertiary hospital in a suburban setting in Northern Nigeria. Trop J Obstet Gynaecol. 2017;34(1):54–60.
19. Selke HM, Kimaiyo S, Sidle JE, Vedanthan R, Tierney WM, Shen C, et al. Task-shifting of antiretroviral delivery from health care workers to persons living with HIV/AIDS: clinical outcomes of a community-based program in Kenya. JAIDS J Acquir Immune Defic Syndr. 2010;55(4):483–90.
20. Druce N, Nolan A. Seizing the big missed opportunity: linking HIV and maternity care services in sub-Saharan Africa. Reprod Health Matters. 2007;15(30):190–201.
21. Singh S, Bankole A, Woog V. Evaluating the need for sex education in developing countries: sexual behaviour, knowledge of preventing sexually transmitted infections/HIV and unplanned pregnancy. Sex Educ. 2005;5(4):307–31.
22. Nakanwagi M, Bulage L, Kwesiga B, Ario AR, Birungi DA, Lukabwe I, et al. Low proportion of women who came knowing their HIV status at first antenatal care visit, Uganda, 2012–2016: a descriptive analysis of surveillance data. BMC Pregnancy Childbirth. 2020;20:1–8.
23. Kanyangarara M, Sakyi K, Laar A. Availability of integrated family planning services in HIV care and support sites in sub-Saharan Africa: a secondary analysis of national health facility surveys. Reprod Health. 2019;16:1–9.
24. Bowring AL, Schwartz S, Lyons C, Rao A, Olawore O, Njindam IM, et al. Unmet need for family planning and experience of unintended pregnancy among female sex workers in urban Cameroon: results from a national cross-sectional study. Glob Heal Sci Pract. 2020;8(1):82–99.
25. Ofurum IC. Sexual Behaviour, Needs and Concerns Regarding Sexual and Reproductive Health among Adults Living with HIV in Sub-Saharan Africa-A Systematic Review. J Adv Med Med Res. 2021;33(11):113–32.
26. Juliastuti D, Dean J, Fitzgerald L. Sexual and reproductive health of women living with HIV in Muslim-majority countries: a systematic mixed studies review. BMC Int Health Hum Rights. 2020;20:1–12.
27. Manzini N. Sexual initiation and childbearing among adolescent girls in KwaZulu Natal, South Africa. Reprod Health Matters. 2001;9(17):44–52.
28. Khan MN, Harris ML, Shifti DM, Laar AS, Loxton D. Effects of unintended pregnancy on maternal healthcare services utilization in low-and lower-middle-income countries: systematic review and meta-analysis. Int J Public Health. 2019;64:743–54.
29. Ochako R, Temmerman M, Mbondo M, Askew I. Determinants of modern contraceptive use among sexually active men in Kenya. Reprod Health. 2017;14:1–15.
30. Ohnishi M, Leshabari S, Tanaka J, Nishihara M. Factors associated with the awareness of contraceptive methods, understanding the prevention of HIV/AIDS and the perception of HIV/AIDS risk among secondary school students in Dar es Salaam, Tanzania. J Rural Med. 2020;15(4):155–63.
31. Yam EA, Kidanu A, Burnett‐Zieman B, Pilgrim N, Okal J, Bekele A, et al. Pregnancy experiences of female sex workers in Adama City, Ethiopia: Complexity of partner relationships and pregnancy intentions. Stud Fam Plann. 2017;48(2):107–19.
32. Ferede TA, Muluneh AG, Wagnew A, Walle AD. Prevalence and associated factors of early sexual initiation among youth female in sub-Saharan Africa: a multilevel analysis of recent demographic and health surveys. BMC Womens Health. 2023;23(1):147.
33. Bafana TNS. Factors influencing contraceptive use and unplanned pregnancy in a South African population. 2010.
34. Khu NH, Vwalika B, Karita E, Kilembe W, Bayingana RA, Sitrin D, et al. Fertility goal-based counseling increases contraceptive implant and IUD use in HIV-discordant couples in Rwanda and Zambia. Contraception. 2013;88(1):74–82.
35. Haile D, Lagebo B. Magnitude of dual contraceptive method utilization and the associated factors among women on antiretroviral treatment in Wolaita zone, Southern Ethiopia. Heliyon. 2022;8(6).
36. Credé S, Hoke T, Constant D, Green MS, Moodley J, Harries J. Factors impacting knowledge and use of long acting and permanent contraceptive methods by postpartum HIV positive and negative women in Cape Town, South Africa: a cross-sectional study. BMC Public Health. 2012;12:1–9.
37. Wasie B, Belyhun Y, Moges B, Amare B. Effect of emergency oral contraceptive use on condom utilization and sexual risk taking behaviours among university students, Northwest Ethiopia: a cross-sectional study. BMC Res Notes. 2012;5:1–9.
38. Young IC, Benhabbour SR. Multipurpose prevention technologies: oral, parenteral, and vaginal dosage forms for prevention of HIV/STIs and unplanned pregnancy. Polymers (Basel). 2021;13(15):2450.
39. Tirado V, Orsini N, Strömdahl S, Hanson C, Ekström AM. Knowledge gaps related to HIV and condom use for preventing pregnancy: a cross-sectional study among migrants in Sweden. BMC Public Health. 2024;24(1):2334.
40. Amuyunzu-Nyamongo M, Tendo-Wambua L, Babishangire B, Nyagero J, Yitbarek N, Matasha M, et al. Barriers to behaviour change as a response to STD including HIV/AIDS: the East African experience. In Citeseer; 1999.
41. Chanda P, JO EK, Ochieng LA. FACTORS AFFECTING UPTAKE OF CONTRACEPTIVES AMONG WOMEN AGED 15-25 IN THE CONTEXT OF EARLY PREGNANCY AND HIV/AIDS PREVENTION IN UGANDA.
42. Türmen T. Gender and HIV/aids. Int J Gynecol Obstet. 2003;82(3):411–8.
43. Shrikhande L. HIV in women in South-East Asia and India. Population (Paris). 2008;423–586.
44. Schelar E, Polis CB, Essam T, Looker KJ, Bruni L, Chrisman CJ, et al. Multipurpose prevention technologies for sexual and reproductive health: mapping global needs for introduction of new preventive products. Contraception. 2016;93(1):32–43.
45. Harrington BJ, Pence BW, John M, Melhado CG, Phulusa J, Mthiko B, et al. Prevalence and factors associated with antenatal depressive symptoms among women enrolled in Option B+ antenatal HIV care in Malawi: a cross-sectional analysis. J Ment Heal. 2019;28(2):198–205.
46. Tesfa A, Bizuneh AD, Tesfaye T, Gebru AA, Ayene YY, Tamene BA. Assessment of knowledge, attitude and practice towards emergency contraceptive methods among female students in Seto Semero high school, Jimma town, south west Ethiopia. Sci J Public Heal. 2015;3(4):478–86.
47. Hoque ME. Reported risky sexual practices amongst female undergraduate students in KwaZulu-Natal, South Africa. African J Prim Heal care Fam Med. 2011;3(1):1–6.
48. Maharaj P. Male attitudes to family planning in the era of HIV/AIDS: evidence from KwaZulu-Natal, South Africa. J South Afr Stud. 2001;27(2):245–57.
49. Greene S, Ion A, Kwaramba G, Smith S, Loutfy MR. “Why are you pregnant? What were you thinking?”: How women navigate experiences of HIV-related stigma in medical settings during pregnancy and birth. Soc Work Health Care. 2016;55(2):161–79.
50. Tenkorang EY. Intimate partner violence and the sexual and reproductive health outcomes of women in Ghana. Heal Educ Behav. 2019;46(6):969–80.
51. DeJong J, Jawad R, Mortagy I, Shepard B. The sexual and reproductive health of young people in the Arab countries and Iran. Reprod Health Matters. 2005;13(25):49–59.
52. Tepper NK, Curtis KM, Jamieson DJ, Marchbanks PA. Update to CDC’s US Medical Eligibility Criteria for Contraceptive Use, 2010: revised recommendations for the use of hormonal contraception among women at high risk for HIV infection or infected with HIV. MMWR Morb Mortal Wkly Rep. 2012;61(24).
53. Bobrova N, Sergeev O, Grechukhina T, Kapiga S. Social‐cognitive predictors of consistent condom use among young people in Moscow. Perspect Sex Reprod Health. 2005;37(4):174–8.
54. Organization WH. Making the case for interventions linking sexual and reproductive health and HIV in proposals to the Global Fund to Fight AIDS, Tuberculosis and Malaria. World Health Organization; 2010.
55. Liamputtong P. Women, motherhood, and living with HIV/AIDS: an introduction. In: Women, Motherhood and Living with HIV/AIDS: A Cross-Cultural Perspective. Springer; 2013. p. 1–24.
56. Okereke CI. Unmet reproductive health needs and health-seeking behaviour of adolescents in Owerri, Nigeria. Afr J Reprod Health. 2010;14(1).
57. Mshweshwe-Pakela NT, Matlakala MC, Mbengo F. Attitudes to, and knowledge and use of contraception among female learners attending a high school in Mdantsane. Afr J Nurs Midwifery. 2017;19(1):170–89.
58. Mullu G, Gizachew A, Amare D, Alebel A, Wagnew F, Tiruneh C, et al. Prevalence of gender based violence and associated factors among female students of Menkorer high school in Debre Markos town, Northwest Ethiopia. Science (80- ). 2015;3(1):67–74.
59. Ewunetie AA, Alemayehu M, Endalew B, Abiye H, Gedif G, Simieneh MM. Sexual and reproductive health problems and needs of street youths in East Gojjam Zone Administrative Towns, Ethiopia: Exploratory qualitative study. Adolesc Health Med Ther. 2022;55–66.
60. K Shetty A. Epidemiology of HIV infection in women and children: a global perspective. Curr HIV Res. 2013;11(2):81–92.
61. Bharat S, Mahendra VS. Meeting the sexual and reproductive health needs of people living with HIV: challenges for health care providers. Reprod Health Matters. 2007;15(sup29):93–112.
62. Oyieke JBO, Obore S, Kigondu CS. Millennium development goal 5: a review of maternal mortality at the Kenyatta National Hospital, Nairobi. East Afr Med J. 2006;83(1):4–9.
63. Omoro T, Gray SC, Otieno G, Mbeda C, Phillips-Howard PA, Hayes T, et al. Teen pregnancy in rural western Kenya: a public health issue. Int J Adolesc Youth. 2018;23(4):399–408.
64. Hale F, Vazquez M. Violence against women living with HIV/AIDS: A background paper. Washingt DC Dev Connect. 2011;
65. Allen RH. The role of family planning in poverty reduction. Obstet Gynecol. 2007;110(5):999–1002.
66. Nedjat S, Moazen B, Rezaei F, Hajizadeh S, Majdzadeh R, Setayesh HR, et al. Sexual and reproductive health needs of HIV-positive people in Tehran, Iran: a mixed-method descriptive study. Int J Heal Policy Manag. 2015;4(9):591.
67. Oindo ML. Contraception and sexuality among the youth in Kisumu, Kenya. Afr Health Sci. 2002;2(1):33–9.
68. Pellowski JA, Price DM, Harrison AD, Tuthill EL, Myer L, Operario D, et al. A systematic review and meta-analysis of antiretroviral therapy (ART) adherence interventions for women living with HIV. AIDS Behav. 2019;23:1998–2013.
69. Bankole A, Biddlecom A, Singh S, Guiella G, Zulu E. Sexual behavior, knowledge and information sources of very young adolescents in four sub-Saharan African countries. Afr J Reprod Health. 2007;11(3):28–43.
70. Matseke G, Rodriguez VJ, Peltzer K, Jones D. Intimate partner violence among HIV positive pregnant women in South Africa. J Psychol Africa. 2016;26(3):259–66.
71. Chersich MF, Rees H V. Vulnerability of women in southern Africa to infection with HIV: biological determinants and priority health sector interventions. Aids. 2008;22:S27–40.
72. Juma M, Alaii J, Bartholomew LK, Askew I, Van den Born B. Understanding orphan and non-orphan adolescents’ sexual risks in the context of poverty: a qualitative study in Nyanza Province, Kenya. BMC Int Health Hum Rights. 2013;13:1–8.
73. Kriel Y, Milford C, Cordero J, Suleman F, Beksinska M, Steyn P, et al. Male partner influence on family planning and contraceptive use: perspectives from community members and healthcare providers in KwaZulu-Natal, South Africa. Reprod Health. 2019;16:1–15.
74. Moradi F, Balaghi Z, Joulaei H, Zare N, Mohammadi S, Moghadami M. Unmet Need for Prevention of Unwanted Pregnancy in Shiraz. 2014;
75. Tessema ZT, Teshale AB, Tesema GA, Tamirat KS. Determinants of completing recommended antenatal care utilization in sub-Saharan from 2006 to 2018: evidence from 36 countries using Demographic and Health Surveys. BMC Pregnancy Childbirth. 2021;21:1–12.
76. Bazant ES, Koenig MA. Women’s satisfaction with delivery care in Nairobi’s informal settlements. Int J Qual Heal Care. 2009;21(2):79–86.
77. Greene S, Ion A, Elston D, Kwaramba G, Smith S, Carvalhal A, et al. “why aren’t you breastfeeding?”: how mothers living with HIV talk about infant feeding in a “breast is best” world. Health Care Women Int. 2015;36(8):883–901.
78. Nkosi M. Male circumcision as an HIV prevention strategy and implications for woMen’s sexual and reproductive health rights. Agenda. 2008;22(75):141–54.
79. Weinrib R, Minnis A, Agot K, Ahmed K, Owino F, Manenzhe K, et al. End-users’ poduct preference across three multipurpose prevention technology delivery forms: baseline results from young women in Kenya and South Africa. AIDS Behav. 2018;22:133–45.
80. Osinde MO, Kaye DK, Kakaire O. Intimate partner violence among women with HIV infection in rural Uganda: critical implications for policy and practice. BMC Womens Health. 2011;11:1–7.
81. Adhikari R. Factors affecting awareness of emergency contraception among college students in Kathmandu, Nepal. BMC Womens Health. 2009;9:1–5.
82. Ward MC. A different disease: HIV/AIDS and health care for women in poverty. Cult Med Psychiatry. 1993;17:413–30.
83. Maina WK, Kim AA, Rutherford GW, Harper M, K’Oyugi BO, Sharif S, et al. Kenya AIDS Indicator Surveys 2007 and 2012: implications for public health policies for HIV prevention and treatment. JAIDS J Acquir Immune Defic Syndr. 2014;66:S130–7.
84. Birungi H, Obare F, Mugisha JF, Evelia H, Nyombi J. Preventive service needs of young people perinatally infected with HIV in Uganda. AIDS Care. 2009;21(6):725–31.
85. Renzaho AMN, Kamara JK, Georgeou N, Kamanga G. Sexual, reproductive health needs, and rights of young people in slum areas of Kampala, Uganda: a cross sectional study. PLoS One. 2017;12(1):e0169721.
86. Obiyan MO, Olaleye AO, Oyinlola FF, Folayan MO. Factors associated with pregnancy and induced abortion among street-involved female adolescents in two Nigeria urban cities: a mixed-method study. BMC Health Serv Res. 2023;23(1):25.
87. Joyce C, Keraka ÂM, Njagi J. Assessment of the knowledge on pre conception care among women of reproductive age in Ruiru sub-county, Kiambu county, Kenya. Glob J Heal Sci. 2018;3(1):82–100.
88. Mutsindikwa T, Ashipala DO, Tomas N, Endjala T. Knowledge, Attitudes and Practices of Contraception among tertiary students at the University Campus in Namibia. Glob J Heal Sci. 2019;11:180.
89. Musyimi CW, Mutiso VN, Nyamai DN, Ebuenyi I, Ndetei DM. Suicidal behavior risks during adolescent pregnancy in a low-resource setting: A qualitative study. PLoS One. 2020;15(7):e0236269.
90. Tadesse G, Yakob B. Risky sexual behaviors among female youth in Tiss Abay, a semi-urban area of the Amhara Region, Ethiopia. PLoS One. 2015;10(3):e0119050.
91. Roudi-Fahimi F. Women’s reproductive health in the Middle East and North Africa. Population Reference Bureau Washington, DC; 2003.
92. Harrington EK, Dworkin S, Withers M, Onono M, Kwena Z, Newmann SJ. Gendered power dynamics and women’s negotiation of family planning in a high HIV prevalence setting: a qualitative study of couples in western Kenya. Cult Health Sex. 2016;18(4):453–69.
93. Iran S, Moradi F. Unmet Need for Prevention of Unwanted Pregnancy in Shiraz.
94. Ezegbe C, Stephenson N. The reach and limits of the US president’s emergency plan for aids relief (PEPFAR) funding of prevention of mother-to-child transmission (PMTCT) of HIV in Nigeria. Afr J Reprod Health. 2012;16(1).
95. Mulugeta Y, Berhane Y. Factors associated with pre-marital sexual debut among unmarried high school female students in bahir Dar town, Ethiopia: cross-sectional study. Reprod Health. 2014;11:1–6.
96. Usman I, Adesina A, Usman S, Tshuma N, Olubayo G, Fatunmbi O, et al. Factors Associated with Inconsisitent Female Condom Use among Sexually Active Young Persons in Western Nigeria. Asian J Res Med Pharm Sci. 2017;1(3):1–6.
97. Folayan MO, Harrison A, Odetoyinbo M, Brown B. Tackling the sexual and reproductive health and rights of adolescents living with HIV/AIDS: a priority need in Nigeria. Afr J Reprod Health. 2014;18(1):102–8.
98. Raniga T, Mathe S. Private lives, public issues: childbearing experiences of adolescent mothers in the era of HIV and AIDS in Bhambayi, KwaZulu-Natal, South Africa. Soc Work W. 2011;47(3).
99. Ahenda D. Effectiveness Of Koch Fm community Radio Programmes In the Prevention Of Unintended Pregnancies Among Young Women In Korogocho Informal Settlement. University of Nairobi; 2018.
100. Chukwu LC, Onyeonoro UU, Ikechebelu JI. Knowledge and practice of prevention of maternal to child transmission among HIV positive women of reproductive age in a tertiary hospital, south east Nigeria. J Community Med Prim Heal Care. 2010;22(1–2).
101. Speizer IS, Fotso JC, Davis JT, Saad A, Otai J. Timing and circumstances of first sex among female and male youth from select urban areas of Nigeria, Kenya, and Senegal. J Adolesc Heal. 2013;53(5):609–16.
102. Sofolahan YA, Airhihenbuwa CO. Childbearing decision making: A qualitative study of women living with HIV/AIDS in Southwest Nigeria. AIDS Res Treat. 2012;2012(1):478065.
103. Loutfy M, Johnson M, Walmsley S, Samarina A, Vasquez P, Hao-Lan H, et al. The association between HIV disclosure status and perceived barriers to care faced by women living with HIV in Latin America, China, Central/Eastern Europe, and Western Europe/Canada. AIDS Patient Care STDS. 2016;30(9):435–44.
104. Haram L. 10.‘Prostitutes’ or Modern Women? Negotiating Respectability in Northern Tanzania. Re-thinking Sex Africa. 2004;211.
105. McIntyre P, Organization WH. Pregnant adolescents: delivering on global promise of hope. 2006;
106. Izugbara CO, Egesa C, Okelo R. ‘High profile health facilities can add to your trouble’: women, stigma and un/safe abortion in Kenya. Soc Sci Med. 2015;141:9–18.
107. Deering KN, Logie C, Krüsi A, Ranville F, Braschel M, Duff P, et al. Prevalence and correlates of HIV stigma among women living with HIV in metro Vancouver, Canada. AIDS Behav. 2021;25(6):1688–98.
108. Saul J, Bachman G, Allen S, Toiv NF, Cooney C, Beamon T. The DREAMS core package of interventions: a comprehensive approach to preventing HIV among adolescent girls and young women. PLoS One. 2018;13(12):e0208167.
109. Mbonile L, Kayombo EJ. Assessing acceptability of parents/guardians of adolescents towards introduction of sex and reproductive health education in schools at Kinondoni Municipal in Dar es Salaam city. 2008;
110. Singh S, Prada E, Mirembe F, Kiggundu C. The incidence of induced abortion in Uganda. Int Fam Plan Perspect. 2005;183–91.
111. Becquet V, Nouaman M, Plazy M, Masumbuko J-M, Anoma C, Kouame S, et al. Sexual health needs of female sex workers in Côte d’Ivoire: a mixed-methods study to prepare the future implementation of pre-exposure prophylaxis (PrEP) for HIV prevention. BMJ Open. 2020;10(1):e028508.
112. Salih NA, Metaferia H, Reda AA, Biadgilign S. Premarital sexual activity among unmarried adolescents in northern Ethiopia: a cross-sectional study. Sex Reprod Healthc. 2015;6(1):9–13.
113. Todd CS, Nasir A, Raza Stanekzai M, Scott PT, Strathdee SA, Botros BA, et al. Contraceptive utilization and pregnancy termination among female sex workers in Afghanistan. J Women’s Heal. 2010;19(11):2057–62.
114. Vaina A, Perdikaris P. School-based sex education among adolescents worldwide: Interventions for the prevention of STIs and unintended pregnancies. Br J Child Heal. 2022;3(5):229–42.
115. Rodney P, Ndjakani Y, Ceesay FK, Wilson NO. Addressing the impact of HIV/AIDS on women and children in sub-Saharan Africa: PEPFAR, the US strategy. Afr Today. 2010;57(1):64–76.
116. Tessema M, Bayu H. Knowledge, attitude and practice on emergency contraception and associated factors among female students of Debre-Markos University, Debre-Markos Town, East Gojam Zone, North West Ethiopia, 2013. Glob J Med Res. 2015;15(1):1–8.
117. Miralles C, Mardarescu M, Sherr L. What do we know about the situation of women living with HIV in Europe? Antivir Ther. 2013;18(2_suppl):11–7.
118. Endriyas M, Eshete A, Mekonnen E, Misganaw T, Shiferaw M, Ayele S. Contraceptive utilization and associated factors among women of reproductive age group in Southern Nations Nationalities and Peoples’ Region, Ethiopia: cross-sectional survey, mixed-methods. Contracept Reprod Med. 2017;2:1–9.
119. Ezeh AC, Kodzi I, Emina J. Reaching the urban poor with family planning services. Stud Fam Plann. 2010;41(2):109–16.
120. Sinyange N, Sitali L, Jacobs C, Musonda P, Michelo C. Factors associated with late antenatal care booking: population based observations from the 2007 Zambia demographic and health survey. Pan Afr Med J. 2016;25.
121. Barker GK, Rich S. Influences on adolescent sexuality in Nigeria and Kenya: Findings from recent focus-group discussions. Stud Fam Plann. 1992;23(3):199–210.
122. KUMAR R, PITTROF R. Human Immunodeficiency Virus and Contraception. Mind Gaps Cases Gynaecol Sex Reprod Heal Mind Gaps Cases Gynaecol Sex Reprod Heal E-b. 2021;165.
123. Turi E, Merga BT, Fekadu G, Abajobir AA. Why too soon? Early initiation of sexual intercourse among adolescent females in Ethiopia: evidence from 2016 Ethiopian Demographic and Health Survey. Int J Womens Health. 2020;269–75.
124. Elmore-Meegan M, Conroy RM, Agala CB. Sex workers in Kenya, numbers of clients and associated risks: an exploratory survey. Reprod Health Matters. 2004;12(23):50–7.
125. Lüllmann H, Mohr K. Color atlas of pharmacology. Thieme Stuttgart; 1999.
126. Gribble J, Haffey J. Reproductive health in sub-Saharan Africa. Popul Ref Bur. 2008;8.
127. Haberland N, Rogow D. Sexuality education: emerging trends in evidence and practice. J Adolesc Heal. 2015;56(1):S15–21.
128. Agbemenu KA. A CRITICAL EXAMINATION OF COMPREHENSIVE SEX EDUCATION PROGRAMMES TARGETING GIRLS BETWEEN THE AGES OF 14-18, IN KENYA, EAST AFRICA. University of Pittsburgh; 2009.
129. Muhwava LS, Morojele N, London L. Psychosocial factors associated with early initiation and frequency of antenatal care (ANC) visits in a rural and urban setting in South Africa: a cross-sectional survey. BMC Pregnancy Childbirth. 2016;16:1–9.
130. Upadhyay UD, Gipson JD, Withers M, Lewis S, Ciaraldi EJ, Fraser A, et al. Womens empowerment and fertility: a review of the literature. Soc Sci Med. 2014;115:111–20.
131. Demissie TW, Nigatu AM, Beyene GM. Assessment of emergency contraceptives utilization and associated factors among female college students at Debre Tabor town. Contracept Reprod Med. 2020;5:1–9.
132. Mbugua N. Factors inhibiting educated mothers in Kenya from giving meaningful sex-education to their daughters. Soc Sci Med. 2007;64(5):1079–89.
133. Abdissa B, Addisie M, Seifu W. Premarital Sexual practices, consequences and associated factors among regular undergraduate female students in Ambo University, Oromia Regional State, Central Ethiopia, 2015. Heal Sci J. 2017;11(1):1.
134. Popoola BI. Sex-negotiation strategies and safer-sex practices among married women in South-western Nigeria. Sex Relatsh Ther. 2009;24(3–4):261–70.
135. Colombini M, James C, Ndwiga C, Mayhew SH. The risks of partner violence following HIV status disclosure, and health service responses: narratives of women attending reproductive health services in Kenya. African J Reprod Gynaecol Endosc. 2016;19(1).
136. Maternal N. Rapid Assessment of Maternal, Newborn and Child Health, Family Planning and HIV/AIDS Integration in Malawi. 2014;
137. Solomon O. Factors Associated with the Utilisation of Family Planning Services among Women of Reproductive Age (15-49 Years) Attending Hoima Regional Referral Hospital.
138. Osok J, Kigamwa P, Stoep A Vander, Huang K-Y, Kumar M. Depression and its psychosocial risk factors in pregnant Kenyan adolescents: a cross-sectional study in a community health Centre of Nairobi. BMC Psychiatry. 2018;18:1–10.
139. Bereda G. Knowledge, Attitude, and Practice towards Emergency Contraceptives use Among Negelle Health Sciience College Female Students, South Eastern, Ethiopia: a Descriptive Cross-Sectional Study, 2021. J Women Heal Care Issues. 2022;5(1).
140. Hoque ME, Ghuman S. Knowledge, practices, and attitudes of emergency contraception among female university students in KwaZulu-Natal, South Africa. 2012;
141. Najmabadi KM, Sharifi F. Sexual education and women empowerment in health: a review of the literature. Int J women’s Heal Reprod Sci. 2019;7(2):150–5.
142. Nanyonga B. Voices of Female Youths Living with HIV/AIDS on their Experiences regarding access and Utilisation of Contraceptives: A case of Kawempe Division Kampala City, Uganda. 2018;
143. Jijini A. HIV/AIDS TECHNICAL BRIEF.
144. Desai M, Phillips-Howard PA, Odhiambo FO, Katana A, Ouma P, Hamel MJ, et al. An analysis of pregnancy-related mortality in the KEMRI/CDC health and demographic surveillance system in western Kenya. PLoS One. 2013;8(7):e68733.
145. Durojaye E. Realizing access to sexual health information and services for adolescents through the protocol to the African Charter on the rights of women. Wash Lee J Civ Rts Soc Just. 2009;16:135.
146. Gelagay AA, Koye DN, Yeshita HY. Demand for long acting contraceptive methods among married HIV positive women attending care at public health facilities at Bahir Dar City, Northwest Ethiopia. Reprod Health. 2015;12:1–9.
147. Juliastuti D, Dean J, Fitzgerald L. Sexual and reproductive health of women living with HIV in Muslim-majority countries: a systematic mixed studies review. BMC Int Health Hum Rights. 2020;20:1–12.
148. Manzini N. Sexual initiation and childbearing among adolescent girls in KwaZulu Natal, South Africa. Reprod Health Matters. 2001;9(17):44–52.
149. Khan MN, Harris ML, Shifti DM, Laar AS, Loxton D. Effects of unintended pregnancy on maternal healthcare services utilization in low-and lower-middle-income countries: systematic review and meta-analysis. Int J Public Health. 2019;64:743–54.
150. Ochako R, Temmerman M, Mbondo M, Askew I. Determinants of modern contraceptive use among sexually active men in Kenya. Reprod Health. 2017;14:1–15.
151. Ohnishi M, Leshabari S, Tanaka J, Nishihara M. Factors associated with the awareness of contraceptive methods, understanding the prevention of HIV/AIDS and the perception of HIV/AIDS risk among secondary school students in Dar es Salaam, Tanzania. J Rural Med. 2020;15(4):155–63.
152. Araya BM, Solomon AA, Gebreslasie KZ, Gudayu TW, Anteneh KT. The role of counseling on modern contraceptive utilization among HIV positive women: the case of Northwest Ethiopia. BMC Womens Health. 2018;18:1–9.
153. Ferede TA, Muluneh AG, Wagnew A, Walle AD. Prevalence and associated factors of early sexual initiation among youth female in sub-Saharan Africa: a multilevel analysis of recent demographic and health surveys. BMC Womens Health. 2023;23(1):147.
154. Khu NH, Vwalika B, Karita E, Kilembe W, Bayingana RA, Sitrin D, et al. Fertility goal-based counseling increases contraceptive implant and IUD use in HIV-discordant couples in Rwanda and Zambia. Contraception. 2013;88(1):74–82.
155. Haile D, Lagebo B. Magnitude of dual contraceptive method utilization and the associated factors among women on antiretroviral treatment in Wolaita zone, Southern Ethiopia. Heliyon. 2022;8(6).
156. Credé S, Hoke T, Constant D, Green MS, Moodley J, Harries J. Factors impacting knowledge and use of long acting and permanent contraceptive methods by postpartum HIV positive and negative women in Cape Town, South Africa: a cross-sectional study. BMC Public Health. 2012;12:1–9.
157. Wasie B, Belyhun Y, Moges B, Amare B. Effect of emergency oral contraceptive use on condom utilization and sexual risk taking behaviours among university students, Northwest Ethiopia: a cross-sectional study. BMC Res Notes. 2012;5:1–9.
158. Feyissa TR, Melka AS. Demand for modern family planning among married women living with HIV in western Ethiopia. PLoS One. 2014;9(11):e113008.
159. Young IC, Benhabbour SR. Multipurpose prevention technologies: oral, parenteral, and vaginal dosage forms for prevention of HIV/STIs and unplanned pregnancy. Polymers (Basel). 2021;13(15):2450.
160. Tirado V, Orsini N, Strömdahl S, Hanson C, Ekström AM. Knowledge gaps related to HIV and condom use for preventing pregnancy: a cross-sectional study among migrants in Sweden. BMC Public Health. 2024;24(1):2334.
161. Amuyunzu-Nyamongo M, Tendo-Wambua L, Babishangire B, Nyagero J, Yitbarek N, Matasha M, et al. Barriers to behaviour change as a response to STD including HIV/AIDS: the East African experience. In Citeseer; 1999.
162. Chanda P, JO EK, Ochieng LA. FACTORS AFFECTING UPTAKE OF CONTRACEPTIVES AMONG WOMEN AGED 15-25 IN THE CONTEXT OF EARLY PREGNANCY AND HIV/AIDS PREVENTION IN UGANDA.
163. Türmen T. Gender and HIV/aids. Int J Gynecol Obstet. 2003;82(3):411–8.
164. Shrikhande L. HIV in women in South-East Asia and India. Population (Paris). 2008;423–586.
165. Schelar E, Polis CB, Essam T, Looker KJ, Bruni L, Chrisman CJ, et al. Multipurpose prevention technologies for sexual and reproductive health: mapping global needs for introduction of new preventive products. Contraception. 2016;93(1):32–43.
166. Harrington BJ, Pence BW, John M, Melhado CG, Phulusa J, Mthiko B, et al. Prevalence and factors associated with antenatal depressive symptoms among women enrolled in Option B+ antenatal HIV care in Malawi: a cross-sectional analysis. J Ment Heal. 2019;28(2):198–205.
167. Tesfa A, Bizuneh AD, Tesfaye T, Gebru AA, Ayene YY, Tamene BA. Assessment of knowledge, attitude and practice towards emergency contraceptive methods among female students in Seto Semero high school, Jimma town, south west Ethiopia. Sci J Public Heal. 2015;3(4):478–86.
168. Hoque ME. Reported risky sexual practices amongst female undergraduate students in KwaZulu-Natal, South Africa. African J Prim Heal care Fam Med. 2011;3(1):1–6.
169. Maharaj P. Male attitudes to family planning in the era of HIV/AIDS: evidence from KwaZulu-Natal, South Africa. J South Afr Stud. 2001;27(2):245–57.
170. Greene S, Ion A, Kwaramba G, Smith S, Loutfy MR. “Why are you pregnant? What were you thinking?”: How women navigate experiences of HIV-related stigma in medical settings during pregnancy and birth. Soc Work Health Care. 2016;55(2):161–79.
171. Tenkorang EY. Intimate partner violence and the sexual and reproductive health outcomes of women in Ghana. Heal Educ Behav. 2019;46(6):969–80.
172. DeJong J, Jawad R, Mortagy I, Shepard B. The sexual and reproductive health of young people in the Arab countries and Iran. Reprod Health Matters. 2005;13(25):49–59.
173. Tepper NK, Curtis KM, Jamieson DJ, Marchbanks PA. Update to CDC’s US Medical Eligibility Criteria for Contraceptive Use, 2010: revised recommendations for the use of hormonal contraception among women at high risk for HIV infection or infected with HIV. MMWR Morb Mortal Wkly Rep. 2012;61(24).
174. Bobrova N, Sergeev O, Grechukhina T, Kapiga S. Social‐cognitive predictors of consistent condom use among young people in Moscow. Perspect Sex Reprod Health. 2005;37(4):174–8.
175. Organization WH. Making the case for interventions linking sexual and reproductive health and HIV in proposals to the Global Fund to Fight AIDS, Tuberculosis and Malaria. World Health Organization; 2010.
176. Liamputtong P. Women, motherhood, and living with HIV/AIDS: an introduction. In: Women, Motherhood and Living with HIV/AIDS: A Cross-Cultural Perspective. Springer; 2013. p. 1–24.
177. Osinde MO, Kaye DK, Kakaire O. Intimate partner violence among women with HIV infection in rural Uganda: critical implications for policy and practice. BMC Womens Health. 2011;11:1–7.
178. Adhikari R. Factors affecting awareness of emergency contraception among college students in Kathmandu, Nepal. BMC Womens Health. 2009;9:1–5.
179. Ward MC. A different disease: HIV/AIDS and health care for women in poverty. Cult Med Psychiatry. 1993;17:413–30.
180. Maina WK, Kim AA, Rutherford GW, Harper M, K’Oyugi BO, Sharif S, et al. Kenya AIDS Indicator Surveys 2007 and 2012: implications for public health policies for HIV prevention and treatment. JAIDS J Acquir Immune Defic Syndr. 2014;66:S130–7.
181. Birungi H, Obare F, Mugisha JF, Evelia H, Nyombi J. Preventive service needs of young people perinatally infected with HIV in Uganda. AIDS Care. 2009;21(6):725–31.
182. Renzaho AMN, Kamara JK, Georgeou N, Kamanga G. Sexual, reproductive health needs, and rights of young people in slum areas of Kampala, Uganda: a cross sectional study. PLoS One. 2017;12(1):e0169721.
183. Obiyan MO, Olaleye AO, Oyinlola FF, Folayan MO. Factors associated with pregnancy and induced abortion among street-involved female adolescents in two Nigeria urban cities: a mixed-method study. BMC Health Serv Res. 2023;23(1):25.
184. Joyce C, Keraka ÂM, Njagi J. Assessment of the knowledge on pre conception care among women of reproductive age in Ruiru sub-county, Kiambu county, Kenya. Glob J Heal Sci. 2018;3(1):82–100.
185. Mutsindikwa T, Ashipala DO, Tomas N, Endjala T. Knowledge, Attitudes and Practices of Contraception among tertiary students at the University Campus in Namibia. Glob J Heal Sci. 2019;11:180.
186. Musyimi CW, Mutiso VN, Nyamai DN, Ebuenyi I, Ndetei DM. Suicidal behavior risks during adolescent pregnancy in a low-resource setting: A qualitative study. PLoS One. 2020;15(7):e0236269.
187. Tadesse G, Yakob B. Risky sexual behaviors among female youth in Tiss Abay, a semi-urban area of the Amhara Region, Ethiopia. PLoS One. 2015;10(3):e0119050.
188. Roudi-Fahimi F. Women’s reproductive health in the Middle East and North Africa. Population Reference Bureau Washington, DC; 2003.
189. Harrington EK, Dworkin S, Withers M, Onono M, Kwena Z, Newmann SJ. Gendered power dynamics and women’s negotiation of family planning in a high HIV prevalence setting: a qualitative study of couples in western Kenya. Cult Health Sex. 2016;18(4):453–69.
190. Iran S, Moradi F. Unmet Need for Prevention of Unwanted Pregnancy in Shiraz.
191. Ezegbe C, Stephenson N. The reach and limits of the US president’s emergency plan for aids relief (PEPFAR) funding of prevention of mother-to-child transmission (PMTCT) of HIV in Nigeria. Afr J Reprod Health. 2012;16(1).
192. Mulugeta Y, Berhane Y. Factors associated with pre-marital sexual debut among unmarried high school female students in bahir Dar town, Ethiopia: cross-sectional study. Reprod Health. 2014;11:1–6.
193. Usman I, Adesina A, Usman S, Tshuma N, Olubayo G, Fatunmbi O, et al. Factors Associated with Inconsisitent Female Condom Use among Sexually Active Young Persons in Western Nigeria. Asian J Res Med Pharm Sci. 2017;1(3):1–6.
194. Folayan MO, Harrison A, Odetoyinbo M, Brown B. Tackling the sexual and reproductive health and rights of adolescents living with HIV/AIDS: a priority need in Nigeria. Afr J Reprod Health. 2014;18(1):102–8.
195. Raniga T, Mathe S. Private lives, public issues: childbearing experiences of adolescent mothers in the era of HIV and AIDS in Bhambayi, KwaZulu-Natal, South Africa. Soc Work W. 2011;47(3).
