# Supplementary material for: Unintended pregnancy among women living with HIV and its predictors in East Africa, 2024. A systematic review and meta-analysis
Source: PLoS One. 2024 Dec 27;19(12):e0310212. doi: 10.1371/journal.pone.0310212 (PMC11676498; doi:10.1371/journal.pone.0310212)
Supplement: S3 File — (ZIP) [file pone.0310212.s003.zip › Excluded studies due to duplication.docx]

***Excluded studies due to duplication***

1. Manzini N. Sexual initiation and childbearing among adolescent girls in KwaZulu Natal, South Africa. Reprod Health Matters. 2001;9(17):44–52.
2. Khan MN, Harris ML, Shifti DM, Laar AS, Loxton D. Effects of unintended pregnancy on maternal healthcare services utilization in low-and lower-middle-income countries: systematic review and meta-analysis. Int J Public Health. 2019;64:743–54.
3. Ochako R, Temmerman M, Mbondo M, Askew I. Determinants of modern contraceptive use among sexually active men in Kenya. Reprod Health. 2017;14:1–15.
4. Ohnishi M, Leshabari S, Tanaka J, Nishihara M. Factors associated with the awareness of contraceptive methods, understanding the prevention of HIV/AIDS and the perception of HIV/AIDS risk among secondary school students in Dar es Salaam, Tanzania. J Rural Med. 2020;15(4):155–63.
5. Araya BM, Solomon AA, Gebreslasie KZ, Gudayu TW, Anteneh KT. The role of counseling on modern contraceptive utilization among HIV positive women: the case of Northwest Ethiopia. BMC Womens Health. 2018;18:1–9.
6. Yam EA, Kidanu A, Burnett‐Zieman B, Pilgrim N, Okal J, Bekele A, et al. Pregnancy experiences of female sex workers in Adama City, Ethiopia: Complexity of partner relationships and pregnancy intentions. Stud Fam Plann. 2017;48(2):107–19.
7. Ferede TA, Muluneh AG, Wagnew A, Walle AD. Prevalence and associated factors of early sexual initiation among youth female in sub-Saharan Africa: a multilevel analysis of recent demographic and health surveys. BMC Womens Health. 2023;23(1):147.
8. Bafana TNS. Factors influencing contraceptive use and unplanned pregnancy in a South African population. 2010.
9. Mbongueh MC, Nicholas T, Ndum AC, Gisèle EL, Nguedia A, Clement J. Unintended pregnancy and sexually transmissible infections amongst adolescents and young adults in Douala IV municipality, Cameroon: Prevalence, knowledge, and associated factors. 2023;
10. Khu NH, Vwalika B, Karita E, Kilembe W, Bayingana RA, Sitrin D, et al. Fertility goal-based counseling increases contraceptive implant and IUD use in HIV-discordant couples in Rwanda and Zambia. Contraception. 2013;88(1):74–82.
11. Haile D, Lagebo B. Magnitude of dual contraceptive method utilization and the associated factors among women on antiretroviral treatment in Wolaita zone, Southern Ethiopia. Heliyon. 2022;8(6).
12. Credé S, Hoke T, Constant D, Green MS, Moodley J, Harries J. Factors impacting knowledge and use of long acting and permanent contraceptive methods by postpartum HIV positive and negative women in Cape Town, South Africa: a cross-sectional study. BMC Public Health. 2012;12:1–9.
13. Wasie B, Belyhun Y, Moges B, Amare B. Effect of emergency oral contraceptive use on condom utilization and sexual risk taking behaviours among university students, Northwest Ethiopia: a cross-sectional study. BMC Res Notes. 2012;5:1–9.
14. Feyissa TR, Melka AS. Demand for modern family planning among married women living with HIV in western Ethiopia. PLoS One. 2014;9(11):e113008.
15. Bowring AL, Schwartz S, Lyons C, Rao A, Olawore O, Njindam IM, et al. Unmet need for family planning and experience of unintended pregnancy among female sex workers in urban Cameroon: results from a national cross-sectional study. Glob Heal Sci Pract. 2020;8(1):82–99.
16. Tibebu NS, Kassie BA, Anteneh TA, Rade BK. Depression, anxiety and stress among HIV-positive pregnant women in Ethiopia during the COVID-19 pandemic. Trans R Soc Trop Med Hyg. 2023;117(5):317–25.
17. Fotso JC, Izugbara C, Saliku T, Ochako R. Unintended pregnancy and subsequent use of modern contraceptive among slum and non-slum women in Nairobi, Kenya. BMC Pregnancy Childbirth. 2014;14:1–10.
18. Bakari HM, Alo O, Mbwana MS, Salim SM, Ludeman E, Lascko T, et al. Prevalence of unmet need for family planning and unintended pregnancies among women of reproductive age living with HIV in sub-Saharan Africa: a systematic review and meta-analysis. Afr Health Sci. 2024;24(2):41–53.
19. Mayhew SH, Colombini M, Kimani JK, Tomlin K, Warren CE, Initiative I, et al. Fertility intentions and contraceptive practices among clinic-users living with HIV in Kenya: a mixed methods study. BMC Public Health. 2017;17:1–15.
20. Atukunda EC, Mugyenyi GR, Atuhumuza EB, Kaida A, Boatin A, Agaba AG, et al. Factors associated with pregnancy intentions amongst postpartum women living with HIV in rural Southwestern Uganda. AIDS Behav. 2019;23:1552–60.
21. Wall KM, Haddad L, Vwalika B, Htee Khu N, Brill I, Kilembe W, et al. Unintended pregnancy among HIV positive couples receiving integrated HIV counseling, testing, and family planning services in Zambia. PLoS One. 2013;8(9):e75353.
22. Kimani J, Warren C, Abuya T, Mutemwa R, Initiative I, Mayhew S, et al. Family planning use and fertility desires among women living with HIV in Kenya. BMC Public Health. 2015;15:1–10.
23. Namukisa M, Kamacooko O, Lunkuse JF, Ruzagira E, Price MA, Mayanja Y. Incidence of unintended pregnancy and associated factors among adolescent girls and young women at risk of HIV infection in Kampala, Uganda. Front Reprod Heal. 2023;5:1089104.
24. Ikamari L, Izugbara C, Ochako R. Prevalence and determinants of unintended pregnancy among women in Nairobi, Kenya. BMC Pregnancy Childbirth. 2013;13:1–9.
25. Amongi PR. Factors Associated With Unintended Pregnancy Among Hiv Positive Women On Anti Retroviral Therapy In Gulu District. CIU; 2018.
26. Feyissa TR, Harris ML, Melka AS, Loxton D. Unintended pregnancy in women living with HIV in Sub-Saharan Africa: a systematic review and meta-analysis. AIDS Behav. 2019;23:1431–51.
27. Ingabire R, Parker R, Nyombayire J, Ko JE, Mukamuyango J, Bizimana J, et al. Female sex workers in Kigali, Rwanda: a key population at risk of HIV, sexually transmitted infections, and unplanned pregnancy. Int J STD AIDS. 2019;30(6):557–68.
28. Mumah JN, Mulupi S, Wado YD, Ushie BA, Nai D, Kabiru CW, et al. Adolescents’ narratives of coping with unintended pregnancy in Nairobi’s informal settlements. PLoS One. 2020;15(10):e0240797.
29. Mumah J, Kabiru CW, Mukiira C, Brinton J, Mutua M, Izugbara CO, et al. Unintended pregnancies in Kenya: a country profile. 2014;
30. Warren CE, Abuya T, Askew I. Family planning practices and pregnancy intentions among HIV-positive and HIV-negative postpartum women in Swaziland: a cross sectional survey. BMC Pregnancy Childbirth. 2013;13:1–10.
31. Solanke BL. Do community characteristics influence unintended pregnancies in Kenya? Malawi Med J. 2019;31(1):56–64.
32. Luchters S, Bosire W, Feng A, Richter ML, King’ola N, Ampt F, et al. “A baby was an added burden”: predictors and consequences of unintended pregnancies for female sex Workers in Mombasa, Kenya: a mixed-methods study. PLoS One. 2016;11(9):e0162871.
33. 7. Teklu T, Davey G. Which factors influence North Ethiopian adults’ use of dual protection from unintended pregnancy and HIV/AIDS? Ethiop J Heal Dev. 2008;22(3).
34. 8. Raifman J, Chetty T, Tanser F, Mutevedzi T, Matthews P, Herbst K, et al. Preventing unintended pregnancy and HIV transmission: effects of the HIV treatment cascade on contraceptive use and choice in rural KwaZulu-Natal. JAIDS J Acquir Immune Defic Syndr. 2014;67:S218–27.
35. 9. Magadi MA. HIV and Unintended Fertility in Sub-Saharan Africa: Multilevel Predictors of Mistimed and Unwanted Fertility Among HIV-Positive Women. Popul Res Policy Rev. 2021;40(5):987–1024.
36. Mwalye PJ. Impact of Unintended pregnancy on HIV viral load outcomes among postpartum women living with HIV in Cape Town, South Africa: clues from postpartum adherence clubs for antiretroviral therapy trial. 2022;
37. Harrington EK, Newmann SJ, Onono M, Schwartz KD, Bukusi EA, Cohen CR, et al. Fertility intentions and interest in integrated family planning services among women living with HIV in Nyanza Province, Kenya: a qualitative study. Infect Dis Obstet Gynecol. 2012;2012(1):809682.
38. Antelman G, Medley A, Mbatia R, Pals S, Arthur G, Haberlen S, et al. Pregnancy desire and dual method contraceptive use among people living with HIV attending clinical care in Kenya, Namibia and Tanzania. J Fam Plan Reprod Heal Care. 2015;41(1):e1–e1.
39. Heffron R, Thomson K, Celum C, Haberer J, Ngure K, Mugo N, et al. Fertility intentions, pregnancy, and use of PrEP and ART for safer conception among East African HIV serodiscordant couples. AIDS Behav. 2018;22:1758–65.
40. Adeniyi OV, Ajayi AI, Somefun OD, Lambert JS. Provision of immediate postpartum contraception to women living with HIV in the Eastern Cape, South Africa; a cross-sectional analysis. Reprod Health. 2020;17:1–12.
41. Patel RC, Amorim G, Jakait B, Shepherd BE, Mocello AR, Musick B, et al. Pregnancies among women living with HIV using contraceptives and antiretroviral therapy in western Kenya: a retrospective, cohort study. BMC Med. 2021;19:1–11.
42. Bain LE, Zweekhorst MBM, de Cock Buning T. Prevalence and determinants of unintended pregnancy in sub–saharan Africa: a systematic review. Afr J Reprod Health. 2020;24(2):187–205.
43. Fotso JC, Izugbara C, Saliku T, Ochako R. Unintended pregnancy and subsequent use of modern contraceptive among slum and non-slum women in Nairobi, Kenya. BMC Pregnancy Childbirth. 2014;14:1–10.
44. Bakari HM, Alo O, Mbwana MS, Salim SM, Ludeman E, Lascko T, et al. Prevalence of unmet need for family planning and unintended pregnancies among women of reproductive age living with HIV in sub-Saharan Africa: a systematic review and meta-analysis. Afr Health Sci. 2024;24(2):41–53.
45. Njuguna E, Ilovi S, Muiruri P, Mutai K, Kinuthia J, Njoroge P. Factors influencing the utilization of family planning services among HIV infected women in a Kenyan health facility. Int J Reprod Contracept Obs Gynecol. 2017;6(5):1746–52.
46. Lawani LO, Onyebuchi AK, Iyoke CA. Dual method use for protection of pregnancy and disease prevention among HIV-infected women in South East Nigeria. BMC Womens Health. 2014;14:1–6.
47. Habte D, Namasasu J. Family planning use among women living with HIV: knowing HIV positive status helps-results from a national survey. Reprod Health. 2015;12:1–11.
48. Patel RC, Onono M, Gandhi M, Blat C, Hagey J, Shade SB, et al. Pregnancy rates in HIV-positive women using contraceptives and efavirenz-based or nevirapine-based antiretroviral therapy in Kenya: a retrospective cohort study. lancet HIV. 2015;2(11):e474–82.
49. Lewinsohn R, Crankshaw T, Tomlinson M, Gibbs A, Butler L, Smit J. “This baby came up and then he said,“I give up!”: The interplay between unintended pregnancy, sexual partnership dynamics and social support and the impact on women’s well-being in KwaZulu-Natal, South Africa. Midwifery. 2018;62:29–35.
50. Ahinkorah BO, Seidu A-A, Appiah F, Oduro JK, Sambah F, Baatiema L, et al. Effect of sexual violence on planned, mistimed and unwanted pregnancies among women of reproductive age in sub-Saharan Africa: A multi-country analysis of Demographic and Health Surveys. SSM-population Heal. 2020;11:100601.
51. Ochieng Arunda M, Agardh A, Larsson M, Asamoah BO. Survival patterns of neonates born to adolescent mothers and the effect of pregnancy intentions and marital status on newborn survival in Kenya, Uganda, and Tanzania, 2014–2016. Glob Health Action. 2022;15(1):2101731.
52. Ma Q, Ono-Kihara M, Cong L, Xu G, Pan X, Zamani S, et al. Unintended pregnancy and its risk factors among university students in eastern China. Contraception. 2008;77(2):108–13.
53. Mokwena K, Bogale YR. Fertility intention and use of contraception among women living with the human immunodeficiency virus in Oromia Region, Ethiopia. South African Fam Pract. 2017;59(1):46–51.
54. Bakibinga P, Matanda DJ, Ayiko R, Rujumba J, Muiruri C, Amendah D, et al. Pregnancy history and current use of contraception among women of reproductive age in Burundi, Kenya, Rwanda, Tanzania and Uganda: analysis of demographic and health survey data. BMJ Open. 2016;6(3):e009991.
55. Okigbo CC, Speizer IS. Determinants of sexual activity and pregnancy among unmarried young women in urban Kenya: a cross-sectional study. PLoS One. 2015;10(6):e0129286.
56. Ampt FH, Willenberg L, Agius PA, Chersich M, Luchters S, Lim MSC. Incidence of unintended pregnancy among female sex workers in low-income and middle-income countries: a systematic review and meta-analysis. BMJ Open. 2018;8(9):e021779.
57. Duff P, Muzaaya G, Muldoon K, Dobrer S, Akello M, Birungi J, et al. High rates of unintended pregnancies among young women sex Workers in Conflict-affected Northern Uganda: the social contexts of brothels/lodges and substance use. Afr J Reprod Health. 2017;21(2):64–72.
58. Ngugi EW, Kim AA, Nyoka R, Mukui I, Ng’eno B, Rutherford GW, et al. Contraceptive practices and fertility desires among HIV-infected and uninfected women in Kenya: results from a nationally representative study. JAIDS J Acquir Immune Defic Syndr. 2014;66:S75–81.
59. Mubangizi L. Examining the association between future pregnancy intentions, contraceptive use and repeat pregnancies among women living with HIV in Cape Town, South Africa. 2020;
60. Bankole A, Singh S, Hussain R, Oestreicher G. Condom use for preventing STI/HIV and unintended pregnancy among young men in Sub-Saharan Africa. Am J Mens Health. 2009;3(1):60–78.
61. Dhakal S, Song JS, Shin DE, Lee TH, So AY, Nam EW. Unintended pregnancy and its correlates among currently pregnant women in the Kwango District, Democratic Republic of the Congo. Reprod Health. 2016;13:1–7.
62. Izugbara C, Egesa C. The management of unwanted pregnancy among women in Nairobi, Kenya. Int J Sex Heal. 2014;26(2):100–12.
63. Wall KM, Kilembe W, Vwalika B, Haddad LB, Khu NH, Brill I, et al. Optimizing prevention of HIV and unplanned pregnancy in discordant African couples. J women’s Heal. 2017;26(8):900–10.
64. Aragaw FM, Amare T, Teklu RE, Tegegne BA, Alem AZ. Magnitude of unintended pregnancy and its determinants among childbearing age women in low and middle-income countries: evidence from 61 low and middle income countries. Front Reprod Heal. 2023;5:1113926.
65. Chukwunyere AP, Stella KA. Unintended pregnancy among undergraduate students at a select university, Eastern Cape, South Africa: effects, influences, outcomes and solutions. Gend Behav. 2019;17(4):14272–86.
66. De Bruyn M. Living with HIV: challenges in reproductive health care in South Africa. Afr J Reprod Health. 2004;92–8.
67. Stuart GS. Fourteen million women with limited options: HIV/AIDS and highly effective reversible contraception in sub-Saharan Africa. Contraception. 2009;80(5):412–6.
68. Guta NM, Ruksi ST, Senbata GM, Seid K. Predictors of perceived poor social support status of pregnant women attending antiretroviral therapy clinics in south west Ethiopia, 2021. Heliyon. 2023;9(7).
69. Ayenew A. Women living with HIV and dual contraceptive use in Ethiopia: systematic review and meta-analysis. Contracept Reprod Med. 2022;7(1):11.
70. Mbuthia CW. Fertility Desires and Contraceptive Practices Among Hiv Positive Adults at Naivasha District Hospital. University of Nairobi; 2010.
71. Habte D, Teklu S, Melese T, Magafu MGMD. Correlates of unintended pregnancy in Ethiopia: results from a national survey. PLoS One. 2013;8(12):e82987.
72. Rodriguez MI, Reeves MF, Caughey AB. Evaluating the competing risks of HIV acquisition and maternal mortality in Africa: a decision analysis. BJOG An Int J Obstet Gynaecol. 2012;119(9):1067–73.
73. Wapmuk AE, Gbajabiamila TA, Ohihoin AG, Ezechi OC. Family Planning In The Context Of HIV Infection. Niger J Clin Biomed Res Wapmuk al. 2017;7(9):6–22.
74. Bernard C, Jakait B, Fadel WF, Mocello AR, Onono MA, Bukusi EA, et al. Preferences for multipurpose technology and non-oral methods of antiretroviral therapy among women living with HIV in western Kenya: a survey study. Front Glob Women’s Heal. 2022;3:869623.
75. Tuthill EL, Maltby AE, Odhiambo BC, Akama E, Pellowski JA, Cohen CR, et al. “i found out I was pregnant, and I started feeling stressed”: A longitudinal qualitative perspective of mental health experiences among perinatal women living with hiv. AIDS Behav. 2021;25:4154–68.
76. Sibanda MY. Factors influencing women living with HIV/AIDS’intention to fall pregnant among those attending the OI/ART clinic in Murambinda, Buhera District, Manicaland Province, Zimbabwe, 2010. 2010;
77. Kebede HG, Nahusenay H, Birhane Y, Tesfaye DJ. Assessment of contraceptive use and associated factors among HIV positive women in Bahir-Dar Town, Northwest Ethiopia. Open Access Libr J. 2015;2(10):1–19.
78. Astawesegn FH, Stulz V, Conroy E, Mannan H. Trends and effects of antiretroviral therapy coverage during pregnancy on mother-to-child transmission of HIV in Sub-Saharan Africa. Evidence from panel data analysis. BMC Infect Dis. 2022;22(1):134.
79. O’Reilly KR, Kennedy CE, Fonner VA, Sweat MD. Family planning counseling for women living with HIV: a systematic review of the evidence of effectiveness on contraceptive uptake and pregnancy incidence, 1990 to 2011. BMC Public Health. 2013;13:1–10.
80. Bauni EK, Jarabi BO. Family planning and sexual behavior in the era of HIV/AIDS: the case of Nakuru District, Kenya. Wiley Online Library; 2000.
81. Pokharel R, Bhattarai G, Shrestha N, Onta S. Knowledge and utilization of family planning methods among people living with HIV in Kathmandu, Nepal. BMC Health Serv Res. 2018;18:1–12.
82. Kisaakye VK. The Effectiveness of Sexual and Reproductive Health Counselling Services for HIV Positive Women in Comprehensive Care Centres in Langata, Kenya. COHES-JKUAT; 2018.
83. De Paoli MM, Manongi R, Klepp K-I. Factors influencing acceptability of voluntary counselling and HIV-testing among pregnant women in Northern Tanzania. AIDS Care. 2004;16(4):411–25.
84. Long JE, Waruguru G, Yuhas K, Wilson KS, Masese LN, Wanje G, et al. Prevalence and predictors of unmet contraceptive need in HIV-positive female sex workers in Mombasa, Kenya. PLoS One. 2019;14(6):e0218291.
85. Melaku YA, Zeleke EG. Contraceptive utilization and associated factors among HIV positive women on chronic follow up care in Tigray Region, Northern Ethiopia: a cross sectional study. PLoS One. 2014;9(4):e94682.
86. Bergsjø P, Vangen S, Lie RT, Lyatuu R, LIE‐NIELSEN E, Oneko O. Recording of maternal deaths in an East African university hospital. Acta Obstet Gynecol Scand. 2010;89(6):789–93.
87. Cohen S. Hiding in plain sight: the role of contraception in preventing HIV. Guttmacher Policy Rev. 2008;11(1):2–5.
88. Richter DL, Sowell RL, Pluto DM. Factors affecting reproductive decisions of African American women living with HIV. Women Health. 2002;36(1):81–96.
89. Vifeme M, Gwendoline N, Ernest B, Mboh E, Nshom E, Marie TP. Pregnancy and Associated Factors Among Adolescents and Young Adults Living with HIV in the Northwest Region of Cameroon. J Womens Heal Dev. 2022;5(3):221–33.
90. O’Shea MS, Rosenberg NE, Hosseinipour MC, Stuart GS, Miller WC, Kaliti SM, et al. Effect of HIV status on fertility desire and knowledge of long-acting reversible contraception of postpartum Malawian women. AIDS Care. 2015;27(4):489–98.
91. Nakaie N, Tuon S, Nozaki I, Yamaguchi F, Sasaki Y, Kakimoto K. Family planning practice and predictors of risk of inconsistent condom use among HIV-positive women on anti-retroviral therapy in Cambodia. BMC Public Health. 2014;14:1–9.
92. Lunani LL, Abaasa A, Omosa-Manyonyi G. Prevalence and factors associated with contraceptive use among Kenyan women aged 15–49 years. AIDS Behav. 2018;22:125–30.
93. Wilcher R, Cates W. Reaching the underserved: family planning for women with HIV. Stud Fam Plann. 2010;41(2):125–8.
94. Colombini M, Mayhew SH, Mutemwa R, Kivunaga J, Ndwiga C, Team I. Perceptions and experiences of integrated service delivery among women living with HIV attending reproductive health services in Kenya: a mixed methods study. AIDS Behav. 2016;20:2130–40.
95. Skerritt L, Kaida A, O’Brien N, Burchell AN, Bartlett G, Savoie É, et al. Patterns of changing pregnancy intentions among women living with HIV in Canada. BMC Womens Health. 2021;21:1–12.
96. Magadi MA, Agwanda AO. Determinants of transitions to first sexual intercourse, marriage and pregnancy among female adolescents: evidence from South Nyanza, Kenya. J Biosoc Sci. 2009;41(3):409–27.
97. Doherty K, Arena K, Wynn A, Offorjebe OA, Moshashane N, Sickboy O, et al. Unintended pregnancy in Gaborone, Botswana: A cross sectional study. Afr J Reprod Health. 2018;22(2):76–82.
98. Omollo C. Determinants of contraceptives preference and use among people living with hiv and aids in rural areas: a study of Nyamarambe division, Kisii county, Kenya. 2021.
99. Kebede YB, Geremew TT, Mehretie Y, Abejie AN, Bewket L, Dellie E. Associated factors of modern contraceptive use among women infected with human immunodeficiency virus in Enemay District, Northwest Ethiopia: a facility-based cross-sectional study. BMC Public Health. 2019;19:1–11.
100. Tumusiigirwe K. Factors associated with unwanted pregnancies among girls aged 15 to 19 years in Kakoba Division in Mbarara District. 2017;
101. Kamangu AA, Myeya HE. Exploring Young Peoples’ Sexual Behaviours and the Underlying Factors in East Africa: A Review of Literature from Four Countries. J Anthropol Surv India. 2023;72(1):149–62.
102. Zewdu LB, Reta MM, Yigzaw N, Tamirat KS. Prevalence of suicidal ideation and associated factors among HIV positive perinatal women on follow-up at Gondar town health institutions, Northwest Ethiopia: a cross-sectional study. BMC Pregnancy Childbirth. 2021;21:1–9.
103. De Bruyn M. Women, reproductive rights, and HIV/AIDS: Issues on which research and interventions are still needed. J Health Popul Nutr. 2006;24(4):413.
104. Arega T. HIV and Unintended Pregnancy Risk Perception and Contraceptive Use among Youth in Debre Birhan District, Ethiopia. Addis Ababa University; 2010.
105. Abubeker FA, Fanta MB, Dalton VK. Unmet Need for Contraception among HIV‐Positive Women Attending HIV Care and Treatment Service at Saint Paul’s Hospital Millennium Medical College, Addis Ababa, Ethiopia. Int J Reprod Med. 2019;2019(1):3276780.
106. Ma Q, Ono-Kihara M, Cong L, Xu G, Pan X, Zamani S, et al. Early initiation of sexual activity: a risk factor for sexually transmitted diseases, HIV infection, and unwanted pregnancy among university students in China. BMC Public Health. 2009;9:1–8.
107. Shikhansari S, Khalesi ZB, Rad EH. Factors associated with the reproductive health of women living with HIV in Iran. Eur J Obstet Gynecol Reprod Biol X. 2022;13:100136.
108. Nyanja TAN, Tulinius C. Relationships matter: contraceptive choices among HIV-positive women in Tanzania. African J AIDS Res. 2017;16(2):109–17.
109. Johnson LF, Mutemaringa T, Heekes A, Boulle A. Effect of HIV infection and antiretroviral treatment on pregnancy rates in the Western Cape province of South Africa. J Infect Dis. 2020;221(12):1953–62.
110. Nakku‐Joloba E, Pisarski EE, Wyatt MA, Muwonge TR, Asiimwe S, Celum CL, et al. Beyond HIV prevention: everyday life priorities and demand for PrEP among Ugandan HIV serodiscordant couples. African J Reprod Gynaecol Endosc. 2019;22(1).
111. Maharaj P. The dual risks of unwanted pregnancy and HIV/AIDS: the case of KwaZulu-Natal, South Africa. London School of Hygiene & Tropical Medicine; 2003.
112. Adilo TM, Wordofa HM. Prevalence of fertility desire and its associated factors among 15-to 49-year-old people living with HIV/AIDS in Addis Ababa, Ethiopia: a cross-sectional study design. HIV/AIDS-Research Palliat Care. 2017;167–76.
113. Ashimi AO, Amole TG, Abubakar MY, Ugwa EA. Fertility desire and utilization of family planning methods among HIV‑positive women attending a tertiary hospital in a suburban setting in Northern Nigeria. Trop J Obstet Gynaecol. 2017;34(1):54–60.
114. Selke HM, Kimaiyo S, Sidle JE, Vedanthan R, Tierney WM, Shen C, et al. Task-shifting of antiretroviral delivery from health care workers to persons living with HIV/AIDS: clinical outcomes of a community-based program in Kenya. JAIDS J Acquir Immune Defic Syndr. 2010;55(4):483–90.
115. Druce N, Nolan A. Seizing the big missed opportunity: linking HIV and maternity care services in sub-Saharan Africa. Reprod Health Matters. 2007;15(30):190–201.
116. Singh S, Bankole A, Woog V. Evaluating the need for sex education in developing countries: sexual behaviour, knowledge of preventing sexually transmitted infections/HIV and unplanned pregnancy. Sex Educ. 2005;5(4):307–31.
117. Nakanwagi M, Bulage L, Kwesiga B, Ario AR, Birungi DA, Lukabwe I, et al. Low proportion of women who came knowing their HIV status at first antenatal care visit, Uganda, 2012–2016: a descriptive analysis of surveillance data. BMC Pregnancy Childbirth. 2020;20:1–8.
118. Kanyangarara M, Sakyi K, Laar A. Availability of integrated family planning services in HIV care and support sites in sub-Saharan Africa: a secondary analysis of national health facility surveys. Reprod Health. 2019;16:1–9.
119. Bowring AL, Schwartz S, Lyons C, Rao A, Olawore O, Njindam IM, et al. Unmet need for family planning and experience of unintended pregnancy among female sex workers in urban Cameroon: results from a national cross-sectional study. Glob Heal Sci Pract. 2020;8(1):82–99.
120. Ofurum IC. Sexual Behaviour, Needs and Concerns Regarding Sexual and Reproductive Health among Adults Living with HIV in Sub-Saharan Africa-A Systematic Review. J Adv Med Med Res. 2021;33(11):113–32.
121. Abay F, Yeshita HY, Mekonnen FA, Sisay M. Dual contraception method utilization and associated factors among sexually active women on antiretroviral therapy in Gondar City, northwest, Ethiopia: a cross sectional study. BMC Womens Health. 2020;20:1–9.
122. Gelagay AA, Koye DN, Yeshita HY. Demand for long acting contraceptive methods among married HIV positive women attending care at public health facilities at Bahir Dar City, Northwest Ethiopia. Reprod Health. 2015;12:1–9.
123. Juliastuti D, Dean J, Fitzgerald L. Sexual and reproductive health of women living with HIV in Muslim-majority countries: a systematic mixed studies review. BMC Int Health Hum Rights. 2020;20:1–12.
124. Stuart GS. Fourteen million women with limited options: HIV/AIDS and highly effective reversible contraception in sub-Saharan Africa. Contraception. 2009;80(5):412–6.
125. Guta NM, Ruksi ST, Senbata GM, Seid K. Predictors of perceived poor social support status of pregnant women attending antiretroviral therapy clinics in south west Ethiopia, 2021. Heliyon. 2023;9(7).
126. Ayenew A. Women living with HIV and dual contraceptive use in Ethiopia: systematic review and meta-analysis. Contracept Reprod Med. 2022;7(1):11.
127. Mbuthia CW. Fertility Desires and Contraceptive Practices Among Hiv Positive Adults at Naivasha District Hospital. University of Nairobi; 2010.
128. Habte D, Teklu S, Melese T, Magafu MGMD. Correlates of unintended pregnancy in Ethiopia: results from a national survey. PLoS One. 2013;8(12):e82987.
129. Rodriguez MI, Reeves MF, Caughey AB. Evaluating the competing risks of HIV acquisition and maternal mortality in Africa: a decision analysis. BJOG An Int J Obstet Gynaecol. 2012;119(9):1067–73.
130. Wapmuk AE, Gbajabiamila TA, Ohihoin AG, Ezechi OC. Family Planning In The Context Of HIV Infection. Niger J Clin Biomed Res Wapmuk al. 2017;7(9):6–22.
131. Bernard C, Jakait B, Fadel WF, Mocello AR, Onono MA, Bukusi EA, et al. Preferences for multipurpose technology and non-oral methods of antiretroviral therapy among women living with HIV in western Kenya: a survey study. Front Glob Women’s Heal. 2022;3:869623.
132. Tuthill EL, Maltby AE, Odhiambo BC, Akama E, Pellowski JA, Cohen CR, et al. “i found out I was pregnant, and I started feeling stressed”: A longitudinal qualitative perspective of mental health experiences among perinatal women living with hiv. AIDS Behav. 2021;25:4154–68.
133. Sibanda MY. Factors influencing women living with HIV/AIDS’intention to fall pregnant among those attending the OI/ART clinic in Murambinda, Buhera District, Manicaland Province, Zimbabwe, 2010. 2010;
134. Kebede HG, Nahusenay H, Birhane Y, Tesfaye DJ. Assessment of contraceptive use and associated factors among HIV positive women in Bahir-Dar Town, Northwest Ethiopia. Open Access Libr J. 2015;2(10):1–19.
135. Astawesegn FH, Stulz V, Conroy E, Mannan H. Trends and effects of antiretroviral therapy coverage during pregnancy on mother-to-child transmission of HIV in Sub-Saharan Africa. Evidence from panel data analysis. BMC Infect Dis. 2022;22(1):134.
136. O’Reilly KR, Kennedy CE, Fonner VA, Sweat MD. Family planning counseling for women living with HIV: a systematic review of the evidence of effectiveness on contraceptive uptake and pregnancy incidence, 1990 to 2011. BMC Public Health. 2013;13:1–10.
137. Bauni EK, Jarabi BO. Family planning and sexual behavior in the era of HIV/AIDS: the case of Nakuru District, Kenya. Wiley Online Library; 2000.
138. Pokharel R, Bhattarai G, Shrestha N, Onta S. Knowledge and utilization of family planning methods among people living with HIV in Kathmandu, Nepal. BMC Health Serv Res. 2018;18:1–12.
139. Kisaakye VK. The Effectiveness of Sexual and Reproductive Health Counselling Services for HIV Positive Women in Comprehensive Care Centres in Langata, Kenya. COHES-JKUAT; 2018.
140. Akwara PA, Madise NJ, Hinde A. Perception of risk of HIV/AIDS and sexual behaviour in Kenya. J Biosoc Sci. 2003;35(3):385–411.
141. Rasch V, Silberschmidt M, Mchumvu Y, Mmary V. Adolescent girls with illegally induced abortion Dar es Salaam: The discrepancy between sexual behaviour and lack of access to contraception. Reprod Health Matters. 2000;8(15):52–62.
142. Kumar S, Gruskin S, Khosla R, Narasimhan M. Human rights and the sexual and reproductive health of women living with HIV–a literature review. J Int AIDS Soc. 2015;18:20290.
143. Onyeka IN, Miettola J, Vaskilampi T, Ilika AL. Unintended pregnancy and termination of studies among students in Anambra state, Nigeria: Are secondary schools playing their part? Afr J Reprod Health. 2011;15(2):109–15.
144. Mutiso SM, Kinuthia J, Qureshi Z. Contraceptive use among HIV infected women attending Comprehensive Care Centre. East Afr Med J. 2008;85(4):171–7.
145. Omollo C, Shivachi T, Agwanda AO. Genderized Perspectives on Contraceptive Use: An Exploratory Study of Persons Living with HIV in Rural Kenya. 2021;
146. Roxby AC, Unger JA, Slyker JA, Kinuthia J, Lewis A, John-Stewart G, et al. A lifecycle approach to HIV prevention in African women and children. Curr HIV/AIDS Rep. 2014;11:119–27.
147. Peel J, de Gersigny JB, Teague R, Howard J, Bradshaw C, Chen M, et al. Reproductive health among women living with HIV attending Melbourne Sexual Health Centre for HIV care from February 2019 to February 2020. Sex Health. 2024;21(1):NULL-NULL.
148. Mohamed SF, Izugbara C, Moore AM, Mutua M, Kimani-Murage EW, Ziraba AK, et al. The estimated incidence of induced abortion in Kenya: a cross-sectional study. BMC Pregnancy Childbirth. 2015;15:1–10.
149. Smith JM, Moss JA, Srinivasan P, Butkyavichene I, Gunawardana M, Fanter R, et al. Novel multipurpose pod-intravaginal ring for the prevention of HIV, HSV, and unintended pregnancy: Pharmacokinetic evaluation in a macaque model. PLoS One. 2017;1210):e0185946.
150. Worku A, Addisie M. Sexual violence among female high school students in Debark, northwest Ethiopia. East Afr Med J. 2002;79(2):96–9.
151. Newmann SJ, Zakaras JM, Tao AR, Onono M, Bukusi EA, Cohen CR, et al. Integrating family planning into HIV care in western Kenya: HIV care providers’ perspectives and experiences one year following integration. AIDS Care. 2016;28(2):209–13.
152. Toroitich-Ruto C. The determinants of teenage sexuality and their understanding of STDs/HIV/AIDS in Kenya. 1997;
153. Pallitto CC, O’Campo P. The relationship between intimate partner violence and unintended pregnancy: analysis of a national sample from Colombia. Int Fam Plan Perspect. 2004;165–73.
154. King R, Katuntu D, Lifshay J, Packel L, Batamwita R, Nakayiwa S, et al. Processes and outcomes of HIV serostatus disclosure to sexual partners among people living with HIV in Uganda. AIDS Behav. 2008;12:232–43.
155. McCoy SI, Ralph LJ, Wilson W, Padian NS. Alcohol production as an adaptive livelihood strategy for women farmers in Tanzania and its potential for unintended consequences on women’s reproductive health. PLoS One. 2013;8(3):e59343.
156. Genemo ES, Korsa AT, Bayisa HG. Emergency contraceptive pill use and its impact on condom utilization among university students: a cross-sectional study. Int J Womens Health. 2022;1115–26.
157. Gichangi PB. Reproductive health awareness among adolescents. East Afr Med J. 2003;80(7):337–8.
158. Matheson R, Moses‐Burton S, Hsieh AC, Dilmitis S, Happy M, Sinyemu E, et al. Fundamental concerns of women living with HIV around the implementation of Option B+. J Int AIDS Soc. 2015;18:20286.
159. Cuinhane CE, Roelens K, Vanroelen C, Quive S, Coene G. Perceptions and decision-making with regard to pregnancy among HIV positive women in rural Maputo Provinc, Mozambique–a qualitative study. BMC Womens Health. 2018;18:1–21.
160. Landolta NK, Phanuphaka N, Chaithongwongwatthanab S, Ananworanicha J. Sexual life and contraception in people living with HIV. Health (Irvine Calif). 1:2.
161. Jozani ZB, Bayanolhagh S, Mobaderi T, Pashangzadeh S, Sohrabi A, Tabatabai RA, et al. Effect of Hormonal Contraception on Disease Progression in Women Living With HIV in Tehran, Iran. 2020;
162. Lufuluabo NA. Role of contraception in HIV prevention. Stellenbosch: Stellenbosch University; 2013.
163. Erickson M, Goldenberg SM, Ajok M, Muldoon KA, Muzaaya G, Shannon K. Structural determinants of dual contraceptive use among female sex workers in Gulu, northern Uganda. Int J Gynecol Obstet. 2015;131(1):91–5.
164. Somba MJ, Mbonile M, Obure J, Mahande MJ. Sexual behaviour, contraceptive knowledge and use among female undergraduates’ students of Muhimbili and Dar es Salaam Universities, Tanzania: a cross-sectional study. BMC Womens Health. 2014;14:1–8.
165. Manzini N. Sexual initiation and childbearing among adolescent girls in KwaZulu Natal, South Africa. Reprod Health Matters. 2001;9(17):44–52.
166. Schaan MM, Taylor M, Gungqisa N, Marlink R. Personal views about womanhood amongst women living with HIV in Botswana. Cult Health Sex. 2016;18(2):171–83.
167. Tibebu NS, Kassie BA, Anteneh TA, Rade BK. Depression, anxiety and stress among HIV-positive pregnant women in Ethiopia during the COVID-19 pandemic. Trans R Soc Trop Med Hyg. 2023;117(5):317–25.
168. Ofurum IC. Sexual Behaviour, Needs and Concerns Regarding Sexual and Reproductive Health among Adults Living with HIV in Sub-Saharan Africa-A Systematic Review. J Adv Med Med Res. 2021;33(11):113–32.
169. Kownaklai J, Graham M, Hayter M. Pregnancy decision making among Thai women living with HIV: a grounded theory study. Midwifery. 2022;115:103490.
170. Esplen E. Women and girls living with HIV/AIDS: overview and annotated bibliography. Institute of Development Studies at the University of Sussex Brighton, UK; 2007.
171. Bouris A, Guilamo-Ramos V, Jaccard J, McCoy W, Aranda D, Pickard A, et al. The feasibility of a clinic-based parent intervention to prevent HIV, sexually transmitted infections, and unintended pregnancies among Latino and African American adolescents. AIDS Patient Care STDS. 2010;24(6):381–7.
172. Mbuthia CW. Fertility Desires and Contraceptive Practices Among Hiv Positive Adults at Naivasha District Hospital. University of Nairobi; 2010.
173. Sibanda MY. Factors influencing women living with HIV/AIDS’intention to fall pregnant among those attending the OI/ART clinic in Murambinda, Buhera District, Manicaland Province, Zimbabwe, 2010. 2010;
174. Bauni EK, Jarabi BO. Family planning and sexual behavior in the era of HIV/AIDS: the case of Nakuru District, Kenya. Wiley Online Library; 2000.
175. Bergsjø P, Vangen S, Lie RT, Lyatuu R, LIE‐NIELSEN E, Oneko O. Recording of maternal deaths in an East African university hospital. Acta Obstet Gynecol Scand. 2010;89(6):789–93.
176. Richter DL, Sowell RL, Pluto DM. Factors affecting reproductive decisions of African American women living with HIV. Women Health. 2002;36(1):81–96.
177. Grossman D, Onono M, Newmann SJ, Blat C, Bukusi EA, Shade SB, et al. Integration of family planning services into HIV care and treatment in Kenya: a cluster-randomized trial. Aids. 2013;27:S77–85.
178. Omollo C. Determinants of contraceptives preference and use among people living with hiv and aids in rural areas: a study of Nyamarambe division, Kisii county, Kenya. 2021.
179. Bachanas P, Kidder D, Medley A, Pals SL, Carpenter D, Howard A, et al. Delivering prevention interventions to people living with HIV in clinical care settings: results of a cluster randomized trial in Kenya, Namibia, and Tanzania. AIDS Behav. 2016;20:2110–8.
180. Adilo TM, Wordofa HM. Prevalence of fertility desire and its associated factors among 15-to 49-year-old people living with HIV/AIDS in Addis Ababa, Ethiopia: a cross-sectional study design. HIV/AIDS-Research Palliat Care. 2017;167–76.
181. Ashimi AO, Amole TG, Abubakar MY, Ugwa EA. Fertility desire and utilization of family planning methods among HIV‑positive women attending a tertiary hospital in a suburban setting in Northern Nigeria. Trop J Obstet Gynaecol. 2017;34(1):54–60.
182. Singh S, Bankole A, Woog V. Evaluating the need for sex education in developing countries: sexual behaviour, knowledge of preventing sexually transmitted infections/HIV and unplanned pregnancy. Sex Educ. 2005;5(4):307–31.
183. Nakakawa F, Mugisha J, Kaaya AN, Tumwesigye NM, Hennessey M. Nutrition education effects on food and nutrition security for women living with HIV/AIDS in Uganda. Food Policy. 2024;128:102715.
184. Omona K, Muhanuzi G. Factors influencing utilization of modern family planning services by persons living with Human Immunodeficiency Virus at Luwero Hospital, Uganda. Afr Health Sci. 2022;22(3):463–76.
185. Stephenson R, Vwalika B, Greenberg L, Ahmed Y, Vwalika C, Chomba E, et al. A randomized controlled trial to promote long-term contraceptive use among HIV-serodiscordant and concordant positive couples in Zambia. J women’s Heal. 2011;20(4):567–74.
186. UNINTENDED PREGNANCY AND ASSOCIATED FACTORS AMONG PREGNANT WOMEN LIVING WITH HIV BAHIRDAR TOWN PUBLIC HEALTH FACILITIES, NORTHWEST ETHIOPIA, 2022
187. Risky sexual practice, unintended pregnancy, contraceptive utilisation, and its determinants among HIV-infected women in Special Zone of Oromia regional state, Ethiopia
188. Ngugi EW, Kim AA, Nyoka R, Mukui I, Ng’eno B, Rutherford GW, et al. Contraceptive practices and fertility desires among HIV-infected and uninfected women in Kenya: results from a nationally representative study. JAIDS J Acquir Immune Defic Syndr. 2014;66:S75–81.
189. Mubangizi L. Examining the association between future pregnancy intentions, contraceptive use and repeat pregnancies among women living with HIV in Cape Town, South Africa. 2020;
190. Bankole A, Singh S, Hussain R, Oestreicher G. Condom use for preventing STI/HIV and unintended pregnancy among young men in Sub-Saharan Africa. Am J Mens Health. 2009;3(1):60–78.
191. Dhakal S, Song JS, Shin DE, Lee TH, So AY, Nam EW. Unintended pregnancy and its correlates among currently pregnant women in the Kwango District, Democratic Republic of the Congo. Reprod Health. 2016;13:1–7.
192. Izugbara C, Egesa C. The management of unwanted pregnancy among women in Nairobi, Kenya. Int J Sex Heal. 2014;26(2):100–12.
193. Wall KM, Kilembe W, Vwalika B, Haddad LB, Khu NH, Brill I, et al. Optimizing prevention of HIV and unplanned pregnancy in discordant African couples. J women’s Heal. 2017;26(8):900–10.
194. Aragaw FM, Amare T, Teklu RE, Tegegne BA, Alem AZ. Magnitude of unintended pregnancy and its determinants among childbearing age women in low and middle-income countries: evidence from 61 low and middle income countries. Front Reprod Heal. 2023;5:1113926.
195. Chukwunyere AP, Stella KA. Unintended pregnancy among undergraduate students at a select university, Eastern Cape, South Africa: effects, influences, outcomes and solutions. Gend Behav. 2019;17(4):14272–86.
196. De Bruyn M. Living with HIV: challenges in reproductive health care in South Africa. Afr J Reprod Health. 2004;92–8.
197. Stuart GS. Fourteen million women with limited options: HIV/AIDS and highly effective reversible contraception in sub-Saharan Africa. Contraception. 2009;80(5):412–6.
198. Guta NM, Ruksi ST, Senbata GM, Seid K. Predictors of perceived poor social support status of pregnant women attending antiretroviral therapy clinics in south west Ethiopia, 2021. Heliyon. 2023;9(7).
199. Ayenew A. Women living with HIV and dual contraceptive use in Ethiopia: systematic review and meta-analysis. Contracept Reprod Med. 2022;7(1):11.
200. Mbuthia CW. Fertility Desires and Contraceptive Practices Among Hiv Positive Adults at Naivasha District Hospital. University of Nairobi; 2010.
201. Habte D, Teklu S, Melese T, Magafu MGMD. Correlates of unintended pregnancy in Ethiopia: results from a national survey. PLoS One. 2013;8(12):e82987.
202. Rodriguez MI, Reeves MF, Caughey AB. Evaluating the competing risks of HIV acquisition and maternal mortality in Africa: a decision analysis. BJOG An Int J Obstet Gynaecol. 2012;119(9):1067–73.
203. Wapmuk AE, Gbajabiamila TA, Ohihoin AG, Ezechi OC. Family Planning In The Context Of HIV Infection. Niger J Clin Biomed Res Wapmuk al. 2017;7(9):6–22.
204. Bernard C, Jakait B, Fadel WF, Mocello AR, Onono MA, Bukusi EA, et al. Preferences for multipurpose technology and non-oral methods of antiretroviral therapy among women living with HIV in western Kenya: a survey study. Front Glob Women’s Heal. 2022;3:869623.
205. Tuthill EL, Maltby AE, Odhiambo BC, Akama E, Pellowski JA, Cohen CR, et al. “i found out I was pregnant, and I started feeling stressed”: A longitudinal qualitative perspective of mental health experiences among perinatal women living with hiv. AIDS Behav. 2021;25:4154–68.
206. Sibanda MY. Factors influencing women living with HIV/AIDS’intention to fall pregnant among those attending the OI/ART clinic in Murambinda, Buhera District, Manicaland Province, Zimbabwe, 2010. 2010;
207. Kebede HG, Nahusenay H, Birhane Y, Tesfaye DJ. Assessment of contraceptive use and associated factors among HIV positive women in Bahir-Dar Town, Northwest Ethiopia. Open Access Libr J. 2015;2(10):1–19.
208. Astawesegn FH, Stulz V, Conroy E, Mannan H. Trends and effects of antiretroviral therapy coverage during pregnancy on mother-to-child transmission of HIV in Sub-Saharan Africa. Evidence from panel data analysis. BMC Infect Dis. 2022;22(1):134.
209. O’Reilly KR, Kennedy CE, Fonner VA, Sweat MD. Family planning counseling for women living with HIV: a systematic review of the evidence of effectiveness on contraceptive uptake and pregnancy incidence, 1990 to 2011. BMC Public Health. 2013;13:1–10.
210. Bauni EK, Jarabi BO. Family planning and sexual behavior in the era of HIV/AIDS: the case of Nakuru District, Kenya. Wiley Online Library; 2000.
211. Pokharel R, Bhattarai G, Shrestha N, Onta S. Knowledge and utilization of family planning methods among people living with HIV in Kathmandu, Nepal. BMC Health Serv Res. 2018;18:1–12.
212. Kisaakye VK. The Effectiveness of Sexual and Reproductive Health Counselling Services for HIV Positive Women in Comprehensive Care Centres in Langata, Kenya. COHES-JKUAT; 2018.
213. De Paoli MM, Manongi R, Klepp K-I. Factors influencing acceptability of voluntary counselling and HIV-testing among pregnant women in Northern Tanzania. AIDS Care. 2004;16(4):411–25.
214. Long JE, Waruguru G, Yuhas K, Wilson KS, Masese LN, Wanje G, et al. Prevalence and predictors of unmet contraceptive need in HIV-positive female sex workers in Mombasa, Kenya. PLoS One. 2019;14(6):e0218291.
215. Melaku YA, Zeleke EG. Contraceptive utilization and associated factors among HIV positive women on chronic follow up care in Tigray Region, Northern Ethiopia: a cross sectional study. PLoS One. 2014;9(4):e94682.
216. Bergsjø P, Vangen S, Lie RT, Lyatuu R, LIE‐NIELSEN E, Oneko O. Recording of maternal deaths in an East African university hospital. Acta Obstet Gynecol Scand. 2010;89(6):789–93.
217. Cohen S. Hiding in plain sight: the role of contraception in preventing HIV. Guttmacher Policy Rev. 2008;11(1):2–5.
218. Richter DL, Sowell RL, Pluto DM. Factors affecting reproductive decisions of African American women living with HIV. Women Health. 2002;36(1):81–96.
219. Vifeme M, Gwendoline N, Ernest B, Mboh E, Nshom E, Marie TP. Pregnancy and Associated Factors Among Adolescents and Young Adults Living with HIV in the Northwest Region of Cameroon. J Womens Heal Dev. 2022;5(3):221–33.
220. O’Shea MS, Rosenberg NE, Hosseinipour MC, Stuart GS, Miller WC, Kaliti SM, et al. Effect of HIV status on fertility desire and knowledge of long-acting reversible contraception of postpartum Malawian women. AIDS Care. 2015;27(4):489–98.
221. Nakaie N, Tuon S, Nozaki I, Yamaguchi F, Sasaki Y, Kakimoto K. Family planning practice and predictors of risk of inconsistent condom use among HIV-positive women on anti-retroviral therapy in Cambodia. BMC Public Health. 2014;14:1–9.
222. Lunani LL, Abaasa A, Omosa-Manyonyi G. Prevalence and factors associated with contraceptive use among Kenyan women aged 15–49 years. AIDS Behav. 2018;22:125–30.
223. Wilcher R, Cates W. Reaching the underserved: family planning for women with HIV. Stud Fam Plann. 2010;41(2):125–8.
224. Colombini M, Mayhew SH, Mutemwa R, Kivunaga J, Ndwiga C, Team I. Perceptions and experiences of integrated service delivery among women living with HIV attending reproductive health services in Kenya: a mixed methods study. AIDS Behav. 2016;20:2130–40.
225. Skerritt L, Kaida A, O’Brien N, Burchell AN, Bartlett G, Savoie É, et al. Patterns of changing pregnancy intentions among women living with HIV in Canada. BMC Womens Health. 2021;21:1–12.
226. Magadi MA, Agwanda AO. Determinants of transitions to first sexual intercourse, marriage and pregnancy among female adolescents: evidence from South Nyanza, Kenya. J Biosoc Sci. 2009;41(3):409–27.
227. Doherty K, Arena K, Wynn A, Offorjebe OA, Moshashane N, Sickboy O, et al. Unintended pregnancy in Gaborone, Botswana: A cross sectional study. Afr J Reprod Health. 2018;22(2):76–82.
228. Omollo C. Determinants of contraceptives preference and use among people living with hiv and aids in rural areas: a study of Nyamarambe division, Kisii county, Kenya. 2021.
229. Kebede YB, Geremew TT, Mehretie Y, Abejie AN, Bewket L, Dellie E. Associated factors of modern contraceptive use among women infected with human immunodeficiency virus in Enemay District, Northwest Ethiopia: a facility-based cross-sectional study. BMC Public Health. 2019;19:1–11.
230. Tumusiigirwe K. Factors associated with unwanted pregnancies among girls aged 15 to 19 years in Kakoba Division in Mbarara District. 2017;
231. Kamangu AA, Myeya HE. Exploring Young Peoples’ Sexual Behaviours and the Underlying Factors in East Africa: A Review of Literature from Four Countries. J Anthropol Surv India. 2023;72(1):149–62.
232. Zewdu LB, Reta MM, Yigzaw N, Tamirat KS. Prevalence of suicidal ideation and associated factors among HIV positive perinatal women on follow-up at Gondar town health institutions, Northwest Ethiopia: a cross-sectional study. BMC Pregnancy Childbirth. 2021;21:1–9.
233. De Bruyn M. Women, reproductive rights, and HIV/AIDS: Issues on which research and interventions are still needed. J Health Popul Nutr. 2006;24(4):413.
234. Arega T. HIV and Unintended Pregnancy Risk Perception and Contraceptive Use among Youth in Debre Birhan District, Ethiopia. Addis Ababa University; 2010.
235. Abubeker FA, Fanta MB, Dalton VK. Unmet Need for Contraception among HIV‐Positive Women Attending HIV Care and Treatment Service at Saint Paul’s Hospital Millennium Medical College, Addis Ababa, Ethiopia. Int J Reprod Med. 2019;2019(1):3276780.
236. Speizer IS, Fotso JC, Davis JT, Saad A, Otai J. Timing and circumstances of first sex among female and male youth from select urban areas of Nigeria, Kenya, and Senegal. J Adolesc Heal. 2013;53(5):609–16.
237. Sofolahan YA, Airhihenbuwa CO. Childbearing decision making: A qualitative study of women living with HIV/AIDS in Southwest Nigeria. AIDS Res Treat. 2012;2012(1):478065.
238. Loutfy M, Johnson M, Walmsley S, Samarina A, Vasquez P, Hao-Lan H, et al. The association between HIV disclosure status and perceived barriers to care faced by women living with HIV in Latin America, China, Central/Eastern Europe, and Western Europe/Canada. AIDS Patient Care STDS. 2016;30(9):435–44.
239. Haram L. 10.‘Prostitutes’ or Modern Women? Negotiating Respectability in Northern Tanzania. Re-thinking Sex Africa. 2004;211.
240. McIntyre P, Organization WH. Pregnant adolescents: delivering on global promise of hope. 2006;
241. Izugbara CO, Egesa C, Okelo R. ‘High profile health facilities can add to your trouble’: women, stigma and un/safe abortion in Kenya. Soc Sci Med. 2015;141:9–18.
242. Deering KN, Logie C, Krüsi A, Ranville F, Braschel M, Duff P, et al. Prevalence and correlates of HIV stigma among women living with HIV in metro Vancouver, Canada. AIDS Behav. 2021;25(6):1688–98.
243. Saul J, Bachman G, Allen S, Toiv NF, Cooney C, Beamon T. The DREAMS core package of interventions: a comprehensive approach to preventing HIV among adolescent girls and young women. PLoS One. 2018;13(12):e0208167.
244. Mbonile L, Kayombo EJ. Assessing acceptability of parents/guardians of adolescents towards introduction of sex and reproductive health education in schools at Kinondoni Municipal in Dar es Salaam city. 2008;
245. Singh S, Prada E, Mirembe F, Kiggundu C. The incidence of induced abortion in Uganda. Int Fam Plan Perspect. 2005;183–91.
246. Becquet V, Nouaman M, Plazy M, Masumbuko J-M, Anoma C, Kouame S, et al. Sexual health needs of female sex workers in Côte d’Ivoire: a mixed-methods study to prepare the future implementation of pre-exposure prophylaxis (PrEP) for HIV prevention. BMJ Open. 2020;10(1):e028508.
247. Salih NA, Metaferia H, Reda AA, Biadgilign S. Premarital sexual activity among unmarried adolescents in northern Ethiopia: a cross-sectional study. Sex Reprod Healthc. 2015;6(1):9–13.
248. Todd CS, Nasir A, Raza Stanekzai M, Scott PT, Strathdee SA, Botros BA, et al. Contraceptive utilization and pregnancy termination among female sex workers in Afghanistan. J Women’s Heal. 2010;19(11):2057–62.
249. Vaina A, Perdikaris P. School-based sex education among adolescents worldwide: Interventions for the prevention of STIs and unintended pregnancies. Br J Child Heal. 2022;3(5):229–42.
250. Rodney P, Ndjakani Y, Ceesay FK, Wilson NO. Addressing the impact of HIV/AIDS on women and children in sub-Saharan Africa: PEPFAR, the US strategy. Afr Today. 2010;57(1):64–76.
251. Tessema M, Bayu H. Knowledge, attitude and practice on emergency contraception and associated factors among female students of Debre-Markos University, Debre-Markos Town, East Gojam Zone, North West Ethiopia, 2013. Glob J Med Res. 2015;15(1):1–8.
252. Miralles C, Mardarescu M, Sherr L. What do we know about the situation of women living with HIV in Europe? Antivir Ther. 2013;18(2_suppl):11–7.
253. Endriyas M, Eshete A, Mekonnen E, Misganaw T, Shiferaw M, Ayele S. Contraceptive utilization and associated factors among women of reproductive age group in Southern Nations Nationalities and Peoples’ Region, Ethiopia: cross-sectional survey, mixed-methods. Contracept Reprod Med. 2017;2:1–9.
254. Ezeh AC, Kodzi I, Emina J. Reaching the urban poor with family planning services. Stud Fam Plann. 2010;41(2):109–16.
255. Sinyange N, Sitali L, Jacobs C, Musonda P, Michelo C. Factors associated with late antenatal care booking: population based observations from the 2007 Zambia demographic and health survey. Pan Afr Med J. 2016;25.
256. Barker GK, Rich S. Influences on adolescent sexuality in Nigeria and Kenya: Findings from recent focus-group discussions. Stud Fam Plann. 1992;23(3):199–210.
257. KUMAR R, PITTROF R. Human Immunodeficiency Virus and Contraception. Mind Gaps Cases Gynaecol Sex Reprod Heal Mind Gaps Cases Gynaecol Sex Reprod Heal E-b. 2021;165.
258. Turi E, Merga BT, Fekadu G, Abajobir AA. Why too soon? Early initiation of sexual intercourse among adolescent females in Ethiopia: evidence from 2016 Ethiopian Demographic and Health Survey. Int J Womens Health. 2020;269–75.
259. Elmore-Meegan M, Conroy RM, Agala CB. Sex workers in Kenya, numbers of clients and associated risks: an exploratory survey. Reprod Health Matters. 2004;12(23):50–7.
260. Lüllmann H, Mohr K. Color atlas of pharmacology. Thieme Stuttgart; 1999.
261. Gribble J, Haffey J. Reproductive health in sub-Saharan Africa. Popul Ref Bur. 2008;8.
262. Haberland N, Rogow D. Sexuality education: emerging trends in evidence and practice. J Adolesc Heal. 2015;56(1):S15–21.
263. Agbemenu KA. A CRITICAL EXAMINATION OF COMPREHENSIVE SEX EDUCATION PROGRAMMES TARGETING GIRLS BETWEEN THE AGES OF 14-18, IN KENYA, EAST AFRICA. University of Pittsburgh; 2009.
264. Muhwava LS, Morojele N, London L. Psychosocial factors associated with early initiation and frequency of antenatal care (ANC) visits in a rural and urban setting in South Africa: a cross-sectional survey. BMC Pregnancy Childbirth. 2016;16:1–9.
265. Upadhyay UD, Gipson JD, Withers M, Lewis S, Ciaraldi EJ, Fraser A, et al. Womens empowerment and fertility: a review of the literature. Soc Sci Med. 2014;115:111–20.
266. Demissie TW, Nigatu AM, Beyene GM. Assessment of emergency contraceptives utilization and associated factors among female college students at Debre Tabor town. Contracept Reprod Med. 2020;5:1–9.
267. Mbugua N. Factors inhibiting educated mothers in Kenya from giving meaningful sex-education to their daughters. Soc Sci Med. 2007;64(5):1079–89.
268. Abdissa B, Addisie M, Seifu W. Premarital Sexual practices, consequences and associated factors among regular undergraduate female students in Ambo University, Oromia Regional State, Central Ethiopia, 2015. Heal Sci J. 2017;11(1):1.
269. Popoola BI. Sex-negotiation strategies and safer-sex practices among married women in South-western Nigeria. Sex Relatsh Ther. 2009;24(3–4):261–70.
270. Colombini M, James C, Ndwiga C, Mayhew SH. The risks of partner violence following HIV status disclosure, and health service responses: narratives of women attending reproductive health services in Kenya. African J Reprod Gynaecol Endosc. 2016;19(1).
271. Maternal N. Rapid Assessment of Maternal, Newborn and Child Health, Family Planning and HIV/AIDS Integration in Malawi. 2014;
272. Solomon O. Factors Associated with the Utilisation of Family Planning Services among Women of Reproductive Age (15-49 Years) Attending Hoima Regional Referral Hospital.
273. Osok J, Kigamwa P, Stoep A Vander, Huang K-Y, Kumar M. Depression and its psychosocial risk factors in pregnant Kenyan adolescents: a cross-sectional study in a community health Centre of Nairobi. BMC Psychiatry. 2018;18:1–10.
274. Bereda G. Knowledge, Attitude, and Practice towards Emergency Contraceptives use Among Negelle Health Sciience College Female Students, South Eastern, Ethiopia: a Descriptive Cross-Sectional Study, 2021. J Women Heal Care Issues. 2022;5(1).
275. Hoque ME, Ghuman S. Knowledge, practices, and attitudes of emergency contraception among female university students in KwaZulu-Natal, South Africa. 2012;
276. Najmabadi KM, Sharifi F. Sexual education and women empowerment in health: a review of the literature. Int J women’s Heal Reprod Sci. 2019;7(2):150–5.
277. Nanyonga B. Voices of Female Youths Living with HIV/AIDS on their Experiences regarding access and Utilisation of Contraceptives: A case of Kawempe Division Kampala City, Uganda. 2018;
278. Jijini A. HIV/AIDS TECHNICAL BRIEF.
279. Desai M, Phillips-Howard PA, Odhiambo FO, Katana A, Ouma P, Hamel MJ, et al. An analysis of pregnancy-related mortality in the KEMRI/CDC health and demographic surveillance system in western Kenya. PLoS One. 2013;8(7):e68733.
280. Durojaye E. Realizing access to sexual health information and services for adolescents through the protocol to the African Charter on the rights of women. Wash Lee J Civ Rts Soc Just. 2009;16:135.
281. Kebede YB, Geremew TT, Mehretie Y, Abejie AN, Bewket L, Dellie E. Associated factors of modern contraceptive use among women infected with human immunodeficiency virus in Enemay District, Northwest Ethiopia: a facility-based cross-sectional study. BMC Public Health. 2019;19:1–11.
282. Tumusiigirwe K. Factors associated with unwanted pregnancies among girls aged 15 to 19 years in Kakoba Division in Mbarara District. 2017;
283. Kamangu AA, Myeya HE. Exploring Young Peoples’ Sexual Behaviours and the Underlying Factors in East Africa: A Review of Literature from Four Countries. J Anthropol Surv India. 2023;72(1):149–62.
284. Zewdu LB, Reta MM, Yigzaw N, Tamirat KS. Prevalence of suicidal ideation and associated factors among HIV positive perinatal women on follow-up at Gondar town health institutions, Northwest Ethiopia: a cross-sectional study. BMC Pregnancy Childbirth. 2021;21:1–9.
285. De Bruyn M. Women, reproductive rights, and HIV/AIDS: Issues on which research and interventions are still needed. J Health Popul Nutr. 2006;24(4):413.
286. Arega T. HIV and Unintended Pregnancy Risk Perception and Contraceptive Use among Youth in Debre Birhan District, Ethiopia. Addis Ababa University; 2010.
287. Abubeker FA, Fanta MB, Dalton VK. Unmet Need for Contraception among HIV‐Positive Women Attending HIV Care and Treatment Service at Saint Paul’s Hospital Millennium Medical College, Addis Ababa, Ethiopia. Int J Reprod Med. 2019;2019(1):3276780.
288. Ma Q, Ono-Kihara M, Cong L, Xu G, Pan X, Zamani S, et al. Early initiation of sexual activity: a risk factor for sexually transmitted diseases, HIV infection, and unwanted pregnancy among university students in China. BMC Public Health. 2009;9:1–8.
289. Shikhansari S, Khalesi ZB, Rad EH. Factors associated with the reproductive health of women living with HIV in Iran. Eur J Obstet Gynecol Reprod Biol X. 2022;13:100136.
290. Nyanja TAN, Tulinius C. Relationships matter: contraceptive choices among HIV-positive women in Tanzania. African J AIDS Res. 2017;16(2):109–17.
291. Johnson LF, Mutemaringa T, Heekes A, Boulle A. Effect of HIV infection and antiretroviral treatment on pregnancy rates in the Western Cape province of South Africa. J Infect Dis. 2020;221(12):1953–62.
292. Nakku‐Joloba E, Pisarski EE, Wyatt MA, Muwonge TR, Asiimwe S, Celum CL, et al. Beyond HIV prevention: everyday life priorities and demand for PrEP among Ugandan HIV serodiscordant couples. African J Reprod Gynaecol Endosc. 2019;22(1).
293. Maharaj P. The dual risks of unwanted pregnancy and HIV/AIDS: the case of KwaZulu-Natal, South Africa. London School of Hygiene & Tropical Medicine; 2003.
294. Adilo TM, Wordofa HM. Prevalence of fertility desire and its associated factors among 15-to 49-year-old people living with HIV/AIDS in Addis Ababa, Ethiopia: a cross-sectional study design. HIV/AIDS-Research Palliat Care. 2017;167–76.
295. Ashimi AO, Amole TG, Abubakar MY, Ugwa EA. Fertility desire and utilization of family planning methods among HIV‑positive women attending a tertiary hospital in a suburban setting in Northern Nigeria. Trop J Obstet Gynaecol. 2017;34(1):54–60.
296. Selke HM, Kimaiyo S, Sidle JE, Vedanthan R, Tierney WM, Shen C, et al. Task-shifting of antiretroviral delivery from health care workers to persons living with HIV/AIDS: clinical outcomes of a community-based program in Kenya. JAIDS J Acquir Immune Defic Syndr. 2010;55(4):483–90.
297. Druce N, Nolan A. Seizing the big missed opportunity: linking HIV and maternity care services in sub-Saharan Africa. Reprod Health Matters. 2007;15(30):190–201.
298. Singh S, Bankole A, Woog V. Evaluating the need for sex education in developing countries: sexual behaviour, knowledge of preventing sexually transmitted infections/HIV and unplanned pregnancy. Sex Educ. 2005;5(4):307–31.
299. Nakanwagi M, Bulage L, Kwesiga B, Ario AR, Birungi DA, Lukabwe I, et al. Low proportion of women who came knowing their HIV status at first antenatal care visit, Uganda, 2012–2016: a descriptive analysis of surveillance data. BMC Pregnancy Childbirth. 2020;20:1–8.
300. 52. Kanyangarara M, Sakyi K, Laar A. Availability of integrated family planning services in HIV care and support sites in sub-Saharan Africa: a secondary analysis of national health facility surveys. Reprod Health. 2019;16:1–9.
301. Bowring AL, Schwartz S, Lyons C, Rao A, Olawore O, Njindam IM, et al. Unmet need for family planning and experience of unintended pregnancy among female sex workers in urban Cameroon: results from a national cross-sectional study. Glob Heal Sci Pract. 2020;8(1):82–99.
302. Ofurum IC. Sexual Behaviour, Needs and Concerns Regarding Sexual and Reproductive Health among Adults Living with HIV in Sub-Saharan Africa-A Systematic Review. J Adv Med Med Res. 2021;33(11):113–32.
303. Abay F, Yeshita HY, Mekonnen FA, Sisay M. Dual contraception method utilization and associated factors among sexually active women on antiretroviral therapy in Gondar City, northwest, Ethiopia: a cross sectional study. BMC Womens Health. 2020;20:1–9.
304. Gelagay AA, Koye DN, Yeshita HY. Demand for long acting contraceptive methods among married HIV positive women attending care at public health facilities at Bahir Dar City, Northwest Ethiopia. Reprod Health. 2015;12:1–9.
305. Juliastuti D, Dean J, Fitzgerald L. Sexual and reproductive health of women living with HIV in Muslim-majority countries: a systematic mixed studies review. BMC Int Health Hum Rights. 2020;20:1–12.
306. Gribble J, Haffey J. Reproductive health in sub-Saharan Africa. Popul Ref Bur. 2008;8.
307. Haberland N, Rogow D. Sexuality education: emerging trends in evidence and practice. J Adolesc Heal. 2015;56(1):S15–21.
308. Agbemenu KA. A CRITICAL EXAMINATION OF COMPREHENSIVE SEX EDUCATION PROGRAMMES TARGETING GIRLS BETWEEN THE AGES OF 14-18, IN KENYA, EAST AFRICA. University of Pittsburgh; 2009.
309. Muhwava LS, Morojele N, London L. Psychosocial factors associated with early initiation and frequency of antenatal care (ANC) visits in a rural and urban setting in South Africa: a cross-sectional survey. BMC Pregnancy Childbirth. 2016;16:1–9.
310. Upadhyay UD, Gipson JD, Withers M, Lewis S, Ciaraldi EJ, Fraser A, et al. Womens empowerment and fertility: a review of the literature. Soc Sci Med. 2014;115:111–20.
311. Demissie TW, Nigatu AM, Beyene GM. Assessment of emergency contraceptives utilization and associated factors among female college students at Debre Tabor town. Contracept Reprod Med. 2020;5:1–9.
312. Mbugua N. Factors inhibiting educated mothers in Kenya from giving meaningful sex-education to their daughters. Soc Sci Med. 2007;64(5):1079–89.
313. Abdissa B, Addisie M, Seifu W. Premarital Sexual practices, consequences and associated factors among regular undergraduate female students in Ambo University, Oromia Regional State, Central Ethiopia, 2015. Heal Sci J. 2017;11(1):1.
314. Popoola BI. Sex-negotiation strategies and safer-sex practices among married women in South-western Nigeria. Sex Relatsh Ther. 2009;24(3–4):261–70.
315. Colombini M, James C, Ndwiga C, Mayhew SH. The risks of partner violence following HIV status disclosure, and health service responses: narratives of women attending reproductive health services in Kenya. African J Reprod Gynaecol Endosc. 2016;19(1).
316. Maternal N. Rapid Assessment of Maternal, Newborn and Child Health, Family Planning and HIV/AIDS Integration in Malawi. 2014;
317. Solomon O. Factors Associated with the Utilisation of Family Planning Services among Women of Reproductive Age (15-49 Years) Attending Hoima Regional Referral Hospital.
318. Osok J, Kigamwa P, Stoep A Vander, Huang K-Y, Kumar M. Depression and its psychosocial risk factors in pregnant Kenyan adolescents: a cross-sectional study in a community health Centre of Nairobi. BMC Psychiatry. 2018;18:1–10.
319. Bereda G. Knowledge, Attitude, and Practice towards Emergency Contraceptives use Among Negelle Health Sciience College Female Students, South Eastern, Ethiopia: a Descriptive Cross-Sectional Study, 2021. J Women Heal Care Issues. 2022;5(1).
320. Hoque ME, Ghuman S. Knowledge, practices, and attitudes of emergency contraception among female university students in KwaZulu-Natal, South Africa. 2012;
321. Najmabadi KM, Sharifi F. Sexual education and women empowerment in health: a review of the literature. Int J women’s Heal Reprod Sci. 2019;7(2):150–5.
322. Nanyonga B. Voices of Female Youths Living with HIV/AIDS on their Experiences regarding access and Utilisation of Contraceptives: A case of Kawempe Division Kampala City, Uganda. 2018;
323. Jijini A. HIV/AIDS TECHNICAL BRIEF.
324. Desai M, Phillips-Howard PA, Odhiambo FO, Katana A, Ouma P, Hamel MJ, et al. An analysis of pregnancy-related mortality in the KEMRI/CDC health and demographic surveillance system in western Kenya. PLoS One. 2013;8(7):e68733.
325. Durojaye E. Realizing access to sexual health information and services for adolescents through the protocol to the African Charter on the rights of women. Wash Lee J Civ Rts Soc Just. 2009;16:135.
326. Meseret M, Shimeka A, Bekele A. Research Article Incidence and Predictors of Pregnancy among Women on ART in Debre Markos Referral Hospital, Northwest Ethiopia: A Five-Year Retrospective Cohort Study. 2017;
327. Gelagay AA, Koye DN, Yeshita HY. Demand for long acting contraceptive methods among married HIV positive women attending care at public health facilities at Bahir Dar City, Northwest Ethiopia. Reprod Health. 2015;12:1–9.
328. Juliastuti D, Dean J, Fitzgerald L. Sexual and reproductive health of women living with HIV in Muslim-majority countries: a systematic mixed studies review. BMC Int Health Hum Rights. 2020;20:1–12.
329. Manzini N. Sexual initiation and childbearing among adolescent girls in KwaZulu Natal, South Africa. Reprod Health Matters. 2001;9(17):44–52.
330. Khan MN, Harris ML, Shifti DM, Laar AS, Loxton D. Effects of unintended pregnancy on maternal healthcare services utilization in low-and lower-middle-income countries: systematic review and meta-analysis. Int J Public Health. 2019;64:743–54.
331. Ochako R, Temmerman M, Mbondo M, Askew I. Determinants of modern contraceptive use among sexually active men in Kenya. Reprod Health. 2017;14:1–15.
332. Ohnishi M, Leshabari S, Tanaka J, Nishihara M. Factors associated with the awareness of contraceptive methods, understanding the prevention of HIV/AIDS and the perception of HIV/AIDS risk among secondary school students in Dar es Salaam, Tanzania. J Rural Med. 2020;15(4):155–63.
333. Araya BM, Solomon AA, Gebreslasie KZ, Gudayu TW, Anteneh KT. The role of counseling on modern contraceptive utilization among HIV positive women: the case of Northwest Ethiopia. BMC Womens Health. 2018;18:1–9.
334. Yam EA, Kidanu A, Burnett‐Zieman B, Pilgrim N, Okal J, Bekele A, et al. Pregnancy experiences of female sex workers in Adama City, Ethiopia: Complexity of partner relationships and pregnancy intentions. Stud Fam Plann. 2017;48(2):107–19.
335. Ferede TA, Muluneh AG, Wagnew A, Walle AD. Prevalence and associated factors of early sexual initiation among youth female in sub-Saharan Africa: a multilevel analysis of recent demographic and health surveys. BMC Womens Health. 2023;23(1):147.
336. Bafana TNS. Factors influencing contraceptive use and unplanned pregnancy in a South African population. 2010.
337. Mbongueh MC, Nicholas T, Ndum AC, Gisèle EL, Nguedia A, Clement J. Unintended pregnancy and sexually transmissible infections amongst adolescents and young adults in Douala IV municipality, Cameroon: Prevalence, knowledge, and associated factors. 2023;
338. Khu NH, Vwalika B, Karita E, Kilembe W, Bayingana RA, Sitrin D, et al. Fertility goal-based counseling increases contraceptive implant and IUD use in HIV-discordant couples in Rwanda and Zambia. Contraception. 2013;88(1):74–82.
339. Haile D, Lagebo B. Magnitude of dual contraceptive method utilization and the associated factors among women on antiretroviral treatment in Wolaita zone, Southern Ethiopia. Heliyon. 2022;8(6).
340. Credé S, Hoke T, Constant D, Green MS, Moodley J, Harries J. Factors impacting knowledge and use of long acting and permanent contraceptive methods by postpartum HIV positive and negative women in Cape Town, South Africa: a cross-sectional study. BMC Public Health. 2012;12:1–9.
341. Wasie B, Belyhun Y, Moges B, Amare B. Effect of emergency oral contraceptive use on condom utilization and sexual risk taking behaviours among university students, Northwest Ethiopia: a cross-sectional study. BMC Res Notes. 2012;5:1–9.
342. Feyissa TR, Melka AS. Demand for modern family planning among married women living with HIV in western Ethiopia. PLoS One. 2014;9(11):e113008.
343. Young IC, Benhabbour SR. Multipurpose prevention technologies: oral, parenteral, and vaginal dosage forms for prevention of HIV/STIs and unplanned pregnancy. Polymers (Basel). 2021;13(15):2450.
344. Tirado V, Orsini N, Strömdahl S, Hanson C, Ekström AM. Knowledge gaps related to HIV and condom use for preventing pregnancy: a cross-sectional study among migrants in Sweden. BMC Public Health. 2024;24(1):2334.
345. Amuyunzu-Nyamongo M, Tendo-Wambua L, Babishangire B, Nyagero J, Yitbarek N, Matasha M, et al. Barriers to behaviour change as a response to STD including HIV/AIDS: the East African experience. In Citeseer; 1999.
346. Chanda P, JO EK, Ochieng LA. FACTORS AFFECTING UPTAKE OF CONTRACEPTIVES AMONG WOMEN AGED 15-25 IN THE CONTEXT OF EARLY PREGNANCY AND HIV/AIDS PREVENTION IN UGANDA.
347. Dibaba Y, Fantahun M, Hindin MJ. The association of unwanted pregnancy and social support with depressive symptoms in pregnancy: evidence from rural Southwestern Ethiopia. BMC Pregnancy Childbirth. 2013;13:1–8.
348. Türmen T. Gender and HIV/aids. Int J Gynecol Obstet. 2003;82(3):411–8.
349. Shrikhande L. HIV in women in South-East Asia and India. Population (Paris). 2008;423–586.
350. Schelar E, Polis CB, Essam T, Looker KJ, Bruni L, Chrisman CJ, et al. Multipurpose prevention technologies for sexual and reproductive health: mapping global needs for introduction of new preventive products. Contraception. 2016;93(1):32–43.
351. Harrington BJ, Pence BW, John M, Melhado CG, Phulusa J, Mthiko B, et al. Prevalence and factors associated with antenatal depressive symptoms among women enrolled in Option B+ antenatal HIV care in Malawi: a cross-sectional analysis. J Ment Heal. 2019;28(2):198–205.
352. Tesfa A, Bizuneh AD, Tesfaye T, Gebru AA, Ayene YY, Tamene BA. Assessment of knowledge, attitude and practice towards emergency contraceptive methods among female students in Seto Semero high school, Jimma town, south west Ethiopia. Sci J Public Heal. 2015;3(4):478–86.
353. Hoque ME. Reported risky sexual practices amongst female undergraduate students in KwaZulu-Natal, South Africa. African J Prim Heal care Fam Med. 2011;3(1):1–6.
354. Maharaj P. Male attitudes to family planning in the era of HIV/AIDS: evidence from KwaZulu-Natal, South Africa. J South Afr Stud. 2001;27(2):245–57.
355. Greene S, Ion A, Kwaramba G, Smith S, Loutfy MR. “Why are you pregnant? What were you thinking?”: How women navigate experiences of HIV-related stigma in medical settings during pregnancy and birth. Soc Work Health Care. 2016;55(2):161–79.
356. Tenkorang EY. Intimate partner violence and the sexual and reproductive health outcomes of women in Ghana. Heal Educ Behav. 2019;46(6):969–80.
357. DeJong J, Jawad R, Mortagy I, Shepard B. The sexual and reproductive health of young people in the Arab countries and Iran. Reprod Health Matters. 2005;13(25):49–59.
358. Tepper NK, Curtis KM, Jamieson DJ, Marchbanks PA. Update to CDC’s US Medical Eligibility Criteria for Contraceptive Use, 2010: revised recommendations for the use of hormonal contraception among women at high risk for HIV infection or infected with HIV. MMWR Morb Mortal Wkly Rep. 2012;61(24).
359. Bobrova N, Sergeev O, Grechukhina T, Kapiga S. Social‐cognitive predictors of consistent condom use among young people in Moscow. Perspect Sex Reprod Health. 2005;37(4):174–8.
360. Organization WH. Making the case for interventions linking sexual and reproductive health and HIV in proposals to the Global Fund to Fight AIDS, Tuberculosis and Malaria. World Health Organization; 2010.
361. Liamputtong P. Women, motherhood, and living with HIV/AIDS: an introduction. In: Women, Motherhood and Living with HIV/AIDS: A Cross-Cultural Perspective. Springer; 2013. p. 1–24.
362. Okereke CI. Unmet reproductive health needs and health-seeking behaviour of adolescents in Owerri, Nigeria. Afr J Reprod Health. 2010;14(1).
363. Mshweshwe-Pakela NT, Matlakala MC, Mbengo F. Attitudes to, and knowledge and use of contraception among female learners attending a high school in Mdantsane. Afr J Nurs Midwifery. 2017;19(1):170–89.
364. Mullu G, Gizachew A, Amare D, Alebel A, Wagnew F, Tiruneh C, et al. Prevalence of gender based violence and associated factors among female students of Menkorer high school in Debre Markos town, Northwest Ethiopia. Science (80- ). 2015;3(1):67–74.
365. Ewunetie AA, Alemayehu M, Endalew B, Abiye H, Gedif G, Simieneh MM. Sexual and reproductive health problems and needs of street youths in East Gojjam Zone Administrative Towns, Ethiopia: Exploratory qualitative study. Adolesc Health Med Ther. 2022;55–66.
366. K Shetty A. Epidemiology of HIV infection in women and children: a global perspective. Curr HIV Res. 2013;11(2):81–92.
367. Bharat S, Mahendra VS. Meeting the sexual and reproductive health needs of people living with HIV: challenges for health care providers. Reprod Health Matters. 2007;15(sup29):93–112.
368. Oyieke JBO, Obore S, Kigondu CS. Millennium development goal 5: a review of maternal mortality at the Kenyatta National Hospital, Nairobi. East Afr Med J. 2006;83(1):4–9.
369. Omoro T, Gray SC, Otieno G, Mbeda C, Phillips-Howard PA, Hayes T, et al. Teen pregnancy in rural western Kenya: a public health issue. Int J Adolesc Youth. 2018;23(4):399–408.
370. Hale F, Vazquez M. Violence against women living with HIV/AIDS: A background paper. Washingt DC Dev Connect. 2011;
371. Allen RH. The role of family planning in poverty reduction. Obstet Gynecol. 2007;110(5):999–1002.
372. Nedjat S, Moazen B, Rezaei F, Hajizadeh S, Majdzadeh R, Setayesh HR, et al. Sexual and reproductive health needs of HIV-positive people in Tehran, Iran: a mixed-method descriptive study. Int J Heal Policy Manag. 2015;4(9):591.
373. Oindo ML. Contraception and sexuality among the youth in Kisumu, Kenya. Afr Health Sci. 2002;2(1):33–9.
374. Pellowski JA, Price DM, Harrison AD, Tuthill EL, Myer L, Operario D, et al. A systematic review and meta-analysis of antiretroviral therapy (ART) adherence interventions for women living with HIV. AIDS Behav. 2019;23:1998–2013.
375. Bankole A, Biddlecom A, Singh S, Guiella G, Zulu E. Sexual behavior, knowledge and information sources of very young adolescents in four sub-Saharan African countries. Afr J Reprod Health. 2007;11(3):28–43.
376. Matseke G, Rodriguez VJ, Peltzer K, Jones D. Intimate partner violence among HIV positive pregnant women in South Africa. J Psychol Africa. 2016;26(3):259–66.
377. Chersich MF, Rees H V. Vulnerability of women in southern Africa to infection with HIV: biological determinants and priority health sector interventions. Aids. 2008;22:S27–40.
378. Juma M, Alaii J, Bartholomew LK, Askew I, Van den Born B. Understanding orphan and non-orphan adolescents’ sexual risks in the context of poverty: a qualitative study in Nyanza Province, Kenya. BMC Int Health Hum Rights. 2013;13:1–8.
379. Kriel Y, Milford C, Cordero J, Suleman F, Beksinska M, Steyn P, et al. Male partner influence on family planning and contraceptive use: perspectives from community members and healthcare providers in KwaZulu-Natal, South Africa. Reprod Health. 2019;16:1–15.
380. Moradi F, Balaghi Z, Joulaei H, Zare N, Mohammadi S, Moghadami M. Unmet Need for Prevention of Unwanted Pregnancy in Shiraz. 2014;
381. Tessema ZT, Teshale AB, Tesema GA, Tamirat KS. Determinants of completing recommended antenatal care utilization in sub-Saharan from 2006 to 2018: evidence from 36 countries using Demographic and Health Surveys. BMC Pregnancy Childbirth. 2021;21:1–12.
382. Bazant ES, Koenig MA. Women’s satisfaction with delivery care in Nairobi’s informal settlements. Int J Qual Heal Care. 2009;21(2):79–86.
383. Greene S, Ion A, Elston D, Kwaramba G, Smith S, Carvalhal A, et al. “why aren’t you breastfeeding?”: how mothers living with HIV talk about infant feeding in a “breast is best” world. Health Care Women Int. 2015;36(8):883–901.
384. Nkosi M. Male circumcision as an HIV prevention strategy and implications for woMen’s sexual and reproductive health rights. Agenda. 2008;22(75):141–54.
385. Weinrib R, Minnis A, Agot K, Ahmed K, Owino F, Manenzhe K, et al. End-users’ poduct preference across three multipurpose prevention technology delivery forms: baseline results from young women in Kenya and South Africa. AIDS Behav. 2018;22:133–45.
386. Osinde MO, Kaye DK, Kakaire O. Intimate partner violence among women with HIV infection in rural Uganda: critical implications for policy and practice. BMC Womens Health. 2011;11:1–7.
387. Adhikari R. Factors affecting awareness of emergency contraception among college students in Kathmandu, Nepal. BMC Womens Health. 2009;9:1–5.
388. Ward MC. A different disease: HIV/AIDS and health care for women in poverty. Cult Med Psychiatry. 1993;17:413–30.
389. Maina WK, Kim AA, Rutherford GW, Harper M, K’Oyugi BO, Sharif S, et al. Kenya AIDS Indicator Surveys 2007 and 2012: implications for public health policies for HIV prevention and treatment. JAIDS J Acquir Immune Defic Syndr. 2014;66:S130–7.
390. Birungi H, Obare F, Mugisha JF, Evelia H, Nyombi J. Preventive service needs of young people perinatally infected with HIV in Uganda. AIDS Care. 2009;21(6):725–31.
391. Renzaho AMN, Kamara JK, Georgeou N, Kamanga G. Sexual, reproductive health needs, and rights of young people in slum areas of Kampala, Uganda: a cross sectional study. PLoS One. 2017;12(1):e0169721.
392. Obiyan MO, Olaleye AO, Oyinlola FF, Folayan MO. Factors associated with pregnancy and induced abortion among street-involved female adolescents in two Nigeria urban cities: a mixed-method study. BMC Health Serv Res. 2023;23(1):25.
393. Joyce C, Keraka ÂM, Njagi J. Assessment of the knowledge on pre conception care among women of reproductive age in Ruiru sub-county, Kiambu county, Kenya. Glob J Heal Sci. 2018;3(1):82–100.
394. Mutsindikwa T, Ashipala DO, Tomas N, Endjala T. Knowledge, Attitudes and Practices of Contraception among tertiary students at the University Campus in Namibia. Glob J Heal Sci. 2019;11:180.
395. Musyimi CW, Mutiso VN, Nyamai DN, Ebuenyi I, Ndetei DM. Suicidal behavior risks during adolescent pregnancy in a low-resource setting: A qualitative study. PLoS One. 2020;15(7):e0236269.
396. Tadesse G, Yakob B. Risky sexual behaviors among female youth in Tiss Abay, a semi-urban area of the Amhara Region, Ethiopia. PLoS One. 2015;10(3):e0119050.
397. Roudi-Fahimi F. Women’s reproductive health in the Middle East and North Africa. Population Reference Bureau Washington, DC; 2003.
398. Harrington EK, Dworkin S, Withers M, Onono M, Kwena Z, Newmann SJ. Gendered power dynamics and women’s negotiation of family planning in a high HIV prevalence setting: a qualitative study of couples in western Kenya. Cult Health Sex. 2016;18(4):453–69.
399. Iran S, Moradi F. Unmet Need for Prevention of Unwanted Pregnancy in Shiraz.
400. Ezegbe C, Stephenson N. The reach and limits of the US president’s emergency plan for aids relief (PEPFAR) funding of prevention of mother-to-child transmission (PMTCT) of HIV in Nigeria. Afr J Reprod Health. 2012;16(1).
401. Mulugeta Y, Berhane Y. Factors associated with pre-marital sexual debut among unmarried high school female students in bahir Dar town, Ethiopia: cross-sectional study. Reprod Health. 2014;11:1–6.
402. Usman I, Adesina A, Usman S, Tshuma N, Olubayo G, Fatunmbi O, et al. Factors Associated with Inconsisitent Female Condom Use among Sexually Active Young Persons in Western Nigeria. Asian J Res Med Pharm Sci. 2017;1(3):1–6.
403. Folayan MO, Harrison A, Odetoyinbo M, Brown B. Tackling the sexual and reproductive health and rights of adolescents living with HIV/AIDS: a priority need in Nigeria. Afr J Reprod Health. 2014;18(1):102–8.
404. Raniga T, Mathe S. Private lives, public issues: childbearing experiences of adolescent mothers in the era of HIV and AIDS in Bhambayi, KwaZulu-Natal, South Africa. Soc Work W. 2011;47(3).
405. Ahenda D. Effectiveness Of Koch Fm community Radio Programmes In the Prevention Of Unintended Pregnancies Among Young Women In Korogocho Informal Settlement. University of Nairobi; 2018.
406. Chukwu LC, Onyeonoro UU, Ikechebelu JI. Knowledge and practice of prevention of maternal to child transmission among HIV positive women of reproductive age in a tertiary hospital, south east Nigeria. J Community Med Prim Heal Care. 2010;22(1–2).
407. Speizer IS, Fotso JC, Davis JT, Saad A, Otai J. Timing and circumstances of first sex among female and male youth from select urban areas of Nigeria, Kenya, and Senegal. J Adolesc Heal. 2013;53(5):609–16.
408. Gelagay AA, Koye DN, Yeshita HY. Demand for long acting contraceptive methods among married HIV positive women attending care at public health facilities at Bahir Dar City, Northwest Ethiopia. Reprod Health. 2015;12:1–9.
409. Juliastuti D, Dean J, Fitzgerald L. Sexual and reproductive health of women living with HIV in Muslim-majority countries: a systematic mixed studies review. BMC Int Health Hum Rights. 2020;20:1–12.
410. Manzini N. Sexual initiation and childbearing among adolescent girls in KwaZulu Natal, South Africa. Reprod Health Matters. 2001;9(17):44–52.
411. Khan MN, Harris ML, Shifti DM, Laar AS, Loxton D. Effects of unintended pregnancy on maternal healthcare services utilization in low-and lower-middle-income countries: systematic review and meta-analysis. Int J Public Health. 2019;64:743–54.
412. Ochako R, Temmerman M, Mbondo M, Askew I. Determinants of modern contraceptive use among sexually active men in Kenya. Reprod Health. 2017;14:1–15.
413. Ohnishi M, Leshabari S, Tanaka J, Nishihara M. Factors associated with the awareness of contraceptive methods, understanding the prevention of HIV/AIDS and the perception of HIV/AIDS risk among secondary school students in Dar es Salaam, Tanzania. J Rural Med. 2020;15(4):155–63.
414. Araya BM, Solomon AA, Gebreslasie KZ, Gudayu TW, Anteneh KT. The role of counseling on modern contraceptive utilization among HIV positive women: the case of Northwest Ethiopia. BMC Womens Health. 2018;18:1–9.
415. Yam EA, Kidanu A, Burnett‐Zieman B, Pilgrim N, Okal J, Bekele A, et al. Pregnancy experiences of female sex workers in Adama City, Ethiopia: Complexity of partner relationships and pregnancy intentions. Stud Fam Plann. 2017;48(2):107–19.
416. Ferede TA, Muluneh AG, Wagnew A, Walle AD. Prevalence and associated factors of early sexual initiation among youth female in sub-Saharan Africa: a multilevel analysis of recent demographic and health surveys. BMC Womens Health. 2023;23(1):147.
417. Bafana TNS. Factors influencing contraceptive use and unplanned pregnancy in a South African population. 2010.
418. Mbongueh MC, Nicholas T, Ndum AC, Gisèle EL, Nguedia A, Clement J. Unintended pregnancy and sexually transmissible infections amongst adolescents and young adults in Douala IV municipality, Cameroon: Prevalence, knowledge, and associated factors. 2023;
419. Khu NH, Vwalika B, Karita E, Kilembe W, Bayingana RA, Sitrin D, et al. Fertility goal-based counseling increases contraceptive implant and IUD use in HIV-discordant couples in Rwanda and Zambia. Contraception. 2013;88(1):74–82.
420. Haile D, Lagebo B. Magnitude of dual contraceptive method utilization and the associated factors among women on antiretroviral treatment in Wolaita zone, Southern Ethiopia. Heliyon. 2022;8(6).
421. Credé S, Hoke T, Constant D, Green MS, Moodley J, Harries J. Factors impacting knowledge and use of long acting and permanent contraceptive methods by postpartum HIV positive and negative women in Cape Town, South Africa: a cross-sectional study. BMC Public Health. 2012;12:1–9.
422. Wasie B, Belyhun Y, Moges B, Amare B. Effect of emergency oral contraceptive use on condom utilization and sexual risk taking behaviours among university students, Northwest Ethiopia: a cross-sectional study. BMC Res Notes. 2012;5:1–9.
423. Feyissa TR, Melka AS. Demand for modern family planning among married women living with HIV in western Ethiopia. PLoS One. 2014;9(11):e113008.
424. Allen RH. The role of family planning in poverty reduction. Obstet Gynecol. 2007;110(5):999–1002.
425. Nedjat S, Moazen B, Rezaei F, Hajizadeh S, Majdzadeh R, Setayesh HR, et al. Sexual and reproductive health needs of HIV-positive people in Tehran, Iran: a mixed-method descriptive study. Int J Heal Policy Manag. 2015;4(9):591.
426. Oindo ML. Contraception and sexuality among the youth in Kisumu, Kenya. Afr Health Sci. 2002;2(1):33–9.
427. Pellowski JA, Price DM, Harrison AD, Tuthill EL, Myer L, Operario D, et al. A systematic review and meta-analysis of antiretroviral therapy (ART) adherence interventions for women living with HIV. AIDS Behav. 2019;23:1998–2013.
428. Bankole A, Biddlecom A, Singh S, Guiella G, Zulu E. Sexual behavior, knowledge and information sources of very young adolescents in four sub-Saharan African countries. Afr J Reprod Health. 2007;11(3):28–43.
429. Matseke G, Rodriguez VJ, Peltzer K, Jones D. Intimate partner violence among HIV positive pregnant women in South Africa. J Psychol Africa. 2016;26(3):259–66.
430. Chersich MF, Rees H V. Vulnerability of women in southern Africa to infection with HIV: biological determinants and priority health sector interventions. Aids. 2008;22:S27–40.
431. Juma M, Alaii J, Bartholomew LK, Askew I, Van den Born B. Understanding orphan and non-orphan adolescents’ sexual risks in the context of poverty: a qualitative study in Nyanza Province, Kenya. BMC Int Health Hum Rights. 2013;13:1–8.
432. Kriel Y, Milford C, Cordero J, Suleman F, Beksinska M, Steyn P, et al. Male partner influence on family planning and contraceptive use: perspectives from community members and healthcare providers in KwaZulu-Natal, South Africa. Reprod Health. 2019;16:1–15.
433. Moradi F, Balaghi Z, Joulaei H, Zare N, Mohammadi S, Moghadami M. Unmet Need for Prevention of Unwanted Pregnancy in Shiraz. 2014;
434. Tessema ZT, Teshale AB, Tesema GA, Tamirat KS. Determinants of completing recommended antenatal care utilization in sub-Saharan from 2006 to 2018: evidence from 36 countries using Demographic and Health Surveys. BMC Pregnancy Childbirth. 2021;21:1–12.
435. Bazant ES, Koenig MA. Women’s satisfaction with delivery care in Nairobi’s informal settlements. Int J Qual Heal Care. 2009;21(2):79–86.
436. Greene S, Ion A, Elston D, Kwaramba G, Smith S, Carvalhal A, et al. “why aren’t you breastfeeding?”: how mothers living with HIV talk about infant feeding in a “breast is best” world. Health Care Women Int. 2015;36(8):883–901.
437. Nkosi M. Male circumcision as an HIV prevention strategy and implications for woMen’s sexual and reproductive health rights. Agenda. 2008;22(75):141–54.
438. Weinrib R, Minnis A, Agot K, Ahmed K, Owino F, Manenzhe K, et al. End-users’ poduct preference across three multipurpose prevention technology delivery forms: baseline results from young women in Kenya and South Africa. AIDS Behav. 2018;22:133–45.
439. Osinde MO, Kaye DK, Kakaire O. Intimate partner violence among women with HIV infection in rural Uganda: critical implications for policy and practice. BMC Womens Health. 2011;11:1–7.
440. Adhikari R. Factors affecting awareness of emergency contraception among college students in Kathmandu, Nepal. BMC Womens Health. 2009;9:1–5.
441. Ward MC. A different disease: HIV/AIDS and health care for women in poverty. Cult Med Psychiatry. 1993;17:413–30.
442. Maina WK, Kim AA, Rutherford GW, Harper M, K’Oyugi BO, Sharif S, et al. Kenya AIDS Indicator Surveys 2007 and 2012: implications for public health policies for HIV prevention and treatment. JAIDS J Acquir Immune Defic Syndr. 2014;66:S130–7.
443. Birungi H, Obare F, Mugisha JF, Evelia H, Nyombi J. Preventive service needs of young people perinatally infected with HIV in Uganda. AIDS Care. 2009;21(6):725–31.
444. Renzaho AMN, Kamara JK, Georgeou N, Kamanga G. Sexual, reproductive health needs, and rights of young people in slum areas of Kampala, Uganda: a cross sectional study. PLoS One. 2017;12(1):e0169721.
445. Obiyan MO, Olaleye AO, Oyinlola FF, Folayan MO. Factors associated with pregnancy and induced abortion among street-involved female adolescents in two Nigeria urban cities: a mixed-method study. BMC Health Serv Res. 2023;23(1):25.
446. Joyce C, Keraka ÂM, Njagi J. Assessment of the knowledge on pre conception care among women of reproductive age in Ruiru sub-county, Kiambu county, Kenya. Glob J Heal Sci. 2018;3(1):82–100.
447. Mutsindikwa T, Ashipala DO, Tomas N, Endjala T. Knowledge, Attitudes and Practices of Contraception among tertiary students at the University Campus in Namibia. Glob J Heal Sci. 2019;11:180.
448. Musyimi CW, Mutiso VN, Nyamai DN, Ebuenyi I, Ndetei DM. Suicidal behavior risks during adolescent pregnancy in a low-resource setting: A qualitative study. PLoS One. 2020;15(7):e0236269.
449. Tadesse G, Yakob B. Risky sexual behaviors among female youth in Tiss Abay, a semi-urban area of the Amhara Region, Ethiopia. PLoS One. 2015;10(3):e0119050.
450. Roudi-Fahimi F. Women’s reproductive health in the Middle East and North Africa. Population Reference Bureau Washington, DC; 2003.
451. Harrington EK, Dworkin S, Withers M, Onono M, Kwena Z, Newmann SJ. Gendered power dynamics and women’s negotiation of family planning in a high HIV prevalence setting: a qualitative study of couples in western Kenya. Cult Health Sex. 2016;18(4):453–69.
452. Iran S, Moradi F. Unmet Need for Prevention of Unwanted Pregnancy in Shiraz.
453. Ezegbe C, Stephenson N. The reach and limits of the US president’s emergency plan for aids relief (PEPFAR) funding of prevention of mother-to-child transmission (PMTCT) of HIV in Nigeria. Afr J Reprod Health. 2012;16(1).
454. Mulugeta Y, Berhane Y. Factors associated with pre-marital sexual debut among unmarried high school female students in bahir Dar town, Ethiopia: cross-sectional study. Reprod Health. 2014;11:1–6.
455. Usman I, Adesina A, Usman S, Tshuma N, Olubayo G, Fatunmbi O, et al. Factors Associated with Inconsisitent Female Condom Use among Sexually Active Young Persons in Western Nigeria. Asian J Res Med Pharm Sci. 2017;1(3):1–6.
456. Folayan MO, Harrison A, Odetoyinbo M, Brown B. Tackling the sexual and reproductive health and rights of adolescents living with HIV/AIDS: a priority need in Nigeria. Afr J Reprod Health. 2014;18(1):102–8.
457. Raniga T, Mathe S. Private lives, public issues: childbearing experiences of adolescent mothers in the era of HIV and AIDS in Bhambayi, KwaZulu-Natal, South Africa. Soc Work W. 2011;47(3).
458. Ahenda D. Effectiveness Of Koch Fm community Radio Programmes In the Prevention Of Unintended Pregnancies Among Young Women In Korogocho Informal Settlement. University of Nairobi; 2018.
459. Chukwu LC, Onyeonoro UU, Ikechebelu JI. Knowledge and practice of prevention of maternal to child transmission among HIV positive women of reproductive age in a tertiary hospital, south east Nigeria. J Community Med Prim Heal Care. 2010;22(1–2).
460. Speizer IS, Fotso JC, Davis JT, Saad A, Otai J. Timing and circumstances of first sex among female and male youth from select urban areas of Nigeria, Kenya, and Senegal. J Adolesc Heal. 2013;53(5):609–16.
461. Gelagay AA, Koye DN, Yeshita HY. Demand for long acting contraceptive methods among married HIV positive women attending care at public health facilities at Bahir Dar City, Northwest Ethiopia. Reprod Health. 2015;12:1–9.
462. Juliastuti D, Dean J, Fitzgerald L. Sexual and reproductive health of women living with HIV in Muslim-majority countries: a systematic mixed studies review. BMC Int Health Hum Rights. 2020;20:1–12.
463. Manzini N. Sexual initiation and childbearing among adolescent girls in KwaZulu Natal, South Africa. Reprod Health Matters. 2001;9(17):44–52.
464. Khan MN, Harris ML, Shifti DM, Laar AS, Loxton D. Effects of unintended pregnancy on maternal healthcare services utilization in low-and lower-middle-income countries: systematic review and meta-analysis. Int J Public Health. 2019;64:743–54.
465. Ochako R, Temmerman M, Mbondo M, Askew I. Determinants of modern contraceptive use among sexually active men in Kenya. Reprod Health. 2017;14:1–15.
466. Ohnishi M, Leshabari S, Tanaka J, Nishihara M. Factors associated with the awareness of contraceptive methods, understanding the prevention of HIV/AIDS and the perception of HIV/AIDS risk among secondary school students in Dar es Salaam, Tanzania. J Rural Med. 2020;15(4):155–63.
467. Araya BM, Solomon AA, Gebreslasie KZ, Gudayu TW, Anteneh KT. The role of counseling on modern contraceptive utilization among HIV positive women: the case of Northwest Ethiopia. BMC Womens Health. 2018;18:1–9.
468. Yam EA, Kidanu A, Burnett‐Zieman B, Pilgrim N, Okal J, Bekele A, et al. Pregnancy experiences of female sex workers in Adama City, Ethiopia: Complexity of partner relationships and pregnancy intentions. Stud Fam Plann. 2017;48(2):107–19.
469. Ferede TA, Muluneh AG, Wagnew A, Walle AD. Prevalence and associated factors of early sexual initiation among youth female in sub-Saharan Africa: a multilevel analysis of recent demographic and health surveys. BMC Womens Health. 2023;23(1):147.
470. Bafana TNS. Factors influencing contraceptive use and unplanned pregnancy in a South African population. 2010.
471. Mbongueh MC, Nicholas T, Ndum AC, Gisèle EL, Nguedia A, Clement J. Unintended pregnancy and sexually transmissible infections amongst adolescents and young adults in Douala IV municipality, Cameroon: Prevalence, knowledge, and associated factors. 2023;
472. Khu NH, Vwalika B, Karita E, Kilembe W, Bayingana RA, Sitrin D, et al. Fertility goal-based counseling increases contraceptive implant and IUD use in HIV-discordant couples in Rwanda and Zambia. Contraception. 2013;88(1):74–82.
473. Haile D, Lagebo B. Magnitude of dual contraceptive method utilization and the associated factors among women on antiretroviral treatment in Wolaita zone, Southern Ethiopia. Heliyon. 2022;8(6).
474. Lunani LL, Abaasa A, Omosa-Manyonyi G. Prevalence and factors associated with contraceptive use among Kenyan women aged 15–49 years. AIDS Behav. 2018;22:125–30.
475. Wilcher R, Cates W. Reaching the underserved: family planning for women with HIV. Stud Fam Plann. 2010;41(2):125–8.
476. Colombini M, Mayhew SH, Mutemwa R, Kivunaga J, Ndwiga C, Team I. Perceptions and experiences of integrated service delivery among women living with HIV attending reproductive health services in Kenya: a mixed methods study. AIDS Behav. 2016;20:2130–40.
477. Skerritt L, Kaida A, O’Brien N, Burchell AN, Bartlett G, Savoie É, et al. Patterns of changing pregnancy intentions among women living with HIV in Canada. BMC Womens Health. 2021;21:1–12.
478. Magadi MA, Agwanda AO. Determinants of transitions to first sexual intercourse, marriage and pregnancy among female adolescents: evidence from South Nyanza, Kenya. J Biosoc Sci. 2009;41(3):409–27.
479. Doherty K, Arena K, Wynn A, Offorjebe OA, Moshashane N, Sickboy O, et al. Unintended pregnancy in Gaborone, Botswana: A cross sectional study. Afr J Reprod Health. 2018;22(2):76–82.
480. Omollo C. Determinants of contraceptives preference and use among people living with hiv and aids in rural areas: a study of Nyamarambe division, Kisii county, Kenya. 2021.
481. Kebede YB, Geremew TT, Mehretie Y, Abejie AN, Bewket L, Dellie E. Associated factors of modern contraceptive use among women infected with human immunodeficiency virus in Enemay District, Northwest Ethiopia: a facility-based cross-sectional study. BMC Public Health. 2019;19:1–11.
482. Tumusiigirwe K. Factors associated with unwanted pregnancies among girls aged 15 to 19 years in Kakoba Division in Mbarara District. 2017;
483. Kamangu AA, Myeya HE. Exploring Young Peoples’ Sexual Behaviours and the Underlying Factors in East Africa: A Review of Literature from Four Countries. J Anthropol Surv India. 2023;72(1):149–62.
484. Zewdu LB, Reta MM, Yigzaw N, Tamirat KS. Prevalence of suicidal ideation and associated factors among HIV positive perinatal women on follow-up at Gondar town health institutions, Northwest Ethiopia: a cross-sectional study. BMC Pregnancy Childbirth. 2021;21:1–9.
485. De Bruyn M. Women, reproductive rights, and HIV/AIDS: Issues on which research and interventions are still needed. J Health Popul Nutr. 2006;24(4):413.
486. Arega T. HIV and Unintended Pregnancy Risk Perception and Contraceptive Use among Youth in Debre Birhan District, Ethiopia. Addis Ababa University; 2010.
487. Abubeker FA, Fanta MB, Dalton VK. Unmet Need for Contraception among HIV‐Positive Women Attending HIV Care and Treatment Service at Saint Paul’s Hospital Millennium Medical College, Addis Ababa, Ethiopia. Int J Reprod Med. 2019;2019(1):3276780.
488. Ma Q, Ono-Kihara M, Cong L, Xu G, Pan X, Zamani S, et al. Early initiation of sexual activity: a risk factor for sexually transmitted diseases, HIV infection, and unwanted pregnancy among university students in China. BMC Public Health. 2009;9:1–8.
489. Shikhansari S, Khalesi ZB, Rad EH. Factors associated with the reproductive health of women living with HIV in Iran. Eur J Obstet Gynecol Reprod Biol X. 2022;13:100136.
490. Nyanja TAN, Tulinius C. Relationships matter: contraceptive choices among HIV-positive women in Tanzania. African J AIDS Res. 2017;16(2):109–17.
491. Johnson LF, Mutemaringa T, Heekes A, Boulle A. Effect of HIV infection and antiretroviral treatment on pregnancy rates in the Western Cape province of South Africa. J Infect Dis. 2020;221(12):1953–62.
492. Nakku‐Joloba E, Pisarski EE, Wyatt MA, Muwonge TR, Asiimwe S, Celum CL, et al. Beyond HIV prevention: everyday life priorities and demand for PrEP among Ugandan HIV serodiscordant couples. African J Reprod Gynaecol Endosc. 2019;22(1).
493. Maharaj P. The dual risks of unwanted pregnancy and HIV/AIDS: the case of KwaZulu-Natal, South Africa. London School of Hygiene & Tropical Medicine; 2003.
494. Adilo TM, Wordofa HM. Prevalence of fertility desire and its associated factors among 15-to 49-year-old people living with HIV/AIDS in Addis Ababa, Ethiopia: a cross-sectional study design. HIV/AIDS-Research Palliat Care. 2017;167–76.
495. Ashimi AO, Amole TG, Abubakar MY, Ugwa EA. Fertility desire and utilization of family planning methods among HIV‑positive women attending a tertiary hospital in a suburban setting in Northern Nigeria. Trop J Obstet Gynaecol. 2017;34(1):54–60.
496. Selke HM, Kimaiyo S, Sidle JE, Vedanthan R, Tierney WM, Shen C, et al. Task-shifting of antiretroviral delivery from health care workers to persons living with HIV/AIDS: clinical outcomes of a community-based program in Kenya. JAIDS J Acquir Immune Defic Syndr. 2010;55(4):483–90.
497. Druce N, Nolan A. Seizing the big missed opportunity: linking HIV and maternity care services in sub-Saharan Africa. Reprod Health Matters. 2007;15(30):190–201.
498. Singh S, Bankole A, Woog V. Evaluating the need for sex education in developing countries: sexual behaviour, knowledge of preventing sexually transmitted infections/HIV and unplanned pregnancy. Sex Educ. 2005;5(4):307–31.
499. Nakanwagi M, Bulage L, Kwesiga B, Ario AR, Birungi DA, Lukabwe I, et al. Low proportion of women who came knowing their HIV status at first antenatal care visit, Uganda, 2012–2016: a descriptive analysis of surveillance data. BMC Pregnancy Childbirth. 2020;20:1–8.
500. 52. Kanyangarara M, Sakyi K, Laar A. Availability of integrated family planning services in HIV care and support sites in sub-Saharan Africa: a secondary analysis of national health facility surveys. Reprod Health. 2019;16:1–9.
501. Bowring AL, Schwartz S, Lyons C, Rao A, Olawore O, Njindam IM, et al. Unmet need for family planning and experience of unintended pregnancy among female sex workers in urban Cameroon: results from a national cross-sectional study. Glob Heal Sci Pract. 2020;8(1):82–99.
502. Ofurum IC. Sexual Behaviour, Needs and Concerns Regarding Sexual and Reproductive Health among Adults Living with HIV in Sub-Saharan Africa-A Systematic Review. J Adv Med Med Res. 2021;33(11):113–32.
503. Abay F, Yeshita HY, Mekonnen FA, Sisay M. Dual contraception method utilization and associated factors among sexually active women on antiretroviral therapy in Gondar City, northwest, Ethiopia: a cross sectional study. BMC Womens Health. 2020;20:1–9.
504. Gelagay AA, Koye DN, Yeshita HY. Demand for long acting contraceptive methods among married HIV positive women attending care at public health facilities at Bahir Dar City, Northwest Ethiopia. Reprod Health. 2015;12:1–9.
505. Juliastuti D, Dean J, Fitzgerald L. Sexual and reproductive health of women living with HIV in Muslim-majority countries: a systematic mixed studies review. BMC Int Health Hum Rights. 2020;20:1–12.
506. Manzini N. Sexual initiation and childbearing among adolescent girls in KwaZulu Natal, South Africa. Reprod Health Matters. 2001;9(17):44–52.
507. Khan MN, Harris ML, Shifti DM, Laar AS, Loxton D. Effects of unintended pregnancy on maternal healthcare services utilization in low-and lower-middle-income countries: systematic review and meta-analysis. Int J Public Health. 2019;64:743–54.
508. Ochako R, Temmerman M, Mbondo M, Askew I. Determinants of modern contraceptive use among sexually active men in Kenya. Reprod Health. 2017;14:1–15.
509. Ohnishi M, Leshabari S, Tanaka J, Nishihara M. Factors associated with the awareness of contraceptive methods, understanding the prevention of HIV/AIDS and the perception of HIV/AIDS risk among secondary school students in Dar es Salaam, Tanzania. J Rural Med. 2020;15(4):155–63.
510. Araya BM, Solomon AA, Gebreslasie KZ, Gudayu TW, Anteneh KT. The role of counseling on modern contraceptive utilization among HIV positive women: the case of Northwest Ethiopia. BMC Womens Health. 2018;18:1–9.
511. Yam EA, Kidanu A, Burnett‐Zieman B, Pilgrim N, Okal J, Bekele A, et al. Pregnancy experiences of female sex workers in Adama City, Ethiopia: Complexity of partner relationships and pregnancy intentions. Stud Fam Plann. 2017;48(2):107–19.
512. Ferede TA, Muluneh AG, Wagnew A, Walle AD. Prevalence and associated factors of early sexual initiation among youth female in sub-Saharan Africa: a multilevel analysis of recent demographic and health surveys. BMC Womens Health. 2023;23(1):147.
513. Bafana TNS. Factors influencing contraceptive use and unplanned pregnancy in a South African population. 2010.
514. Mbongueh MC, Nicholas T, Ndum AC, Gisèle EL, Nguedia A, Clement J. Unintended pregnancy and sexually transmissible infections amongst adolescents and young adults in Douala IV municipality, Cameroon: Prevalence, knowledge, and associated factors. 2023;
515. Khu NH, Vwalika B, Karita E, Kilembe W, Bayingana RA, Sitrin D, et al. Fertility goal-based counseling increases contraceptive implant and IUD use in HIV-discordant couples in Rwanda and Zambia. Contraception. 2013;88(1):74–82.
516. Haile D, Lagebo B. Magnitude of dual contraceptive method utilization and the associated factors among women on antiretroviral treatment in Wolaita zone, Southern Ethiopia. Heliyon. 2022;8(6).
517. Credé S, Hoke T, Constant D, Green MS, Moodley J, Harries J. Factors impacting knowledge and use of long acting and permanent contraceptive methods by postpartum HIV positive and negative women in Cape Town, South Africa: a cross-sectional study. BMC Public Health. 2012;12:1–9.
518. Wasie B, Belyhun Y, Moges B, Amare B. Effect of emergency oral contraceptive use on condom utilization and sexual risk taking behaviours among university students, Northwest Ethiopia: a cross-sectional study. BMC Res Notes. 2012;5:1–9.
519. Feyissa TR, Melka AS. Demand for modern family planning among married women living with HIV in western Ethiopia. PLoS One. 2014;9(11):e113008.
520. Young IC, Benhabbour SR. Multipurpose prevention technologies: oral, parenteral, and vaginal dosage forms for prevention of HIV/STIs and unplanned pregnancy. Polymers (Basel). 2021;13(15):2450.
521. Tirado V, Orsini N, Strömdahl S, Hanson C, Ekström AM. Knowledge gaps related to HIV and condom use for preventing pregnancy: a cross-sectional study among migrants in Sweden. BMC Public Health. 2024;24(1):2334.
522. Amuyunzu-Nyamongo M, Tendo-Wambua L, Babishangire B, Nyagero J, Yitbarek N, Matasha M, et al. Barriers to behaviour change as a response to STD including HIV/AIDS: the East African experience. In Citeseer; 1999.
523. Chanda P, JO EK, Ochieng LA. FACTORS AFFECTING UPTAKE OF CONTRACEPTIVES AMONG WOMEN AGED 15-25 IN THE CONTEXT OF EARLY PREGNANCY AND HIV/AIDS PREVENTION IN UGANDA.
524. Dibaba Y, Fantahun M, Hindin MJ. The association of unwanted pregnancy and social support with depressive symptoms in pregnancy: evidence from rural Southwestern Ethiopia. BMC Pregnancy Childbirth. 2013;13:1–8.
525. Türmen T. Gender and HIV/aids. Int J Gynecol Obstet. 2003;82(3):411–8.
526. Shrikhande L. HIV in women in South-East Asia and India. Population (Paris). 2008;423–586.
527. Schelar E, Polis CB, Essam T, Looker KJ, Bruni L, Chrisman CJ, et al. Multipurpose prevention technologies for sexual and reproductive health: mapping global needs for introduction of new preventive products. Contraception. 2016;93(1):32–43.
528. Harrington BJ, Pence BW, John M, Melhado CG, Phulusa J, Mthiko B, et al. Prevalence and factors associated with antenatal depressive symptoms among women enrolled in Option B+ antenatal HIV care in Malawi: a cross-sectional analysis. J Ment Heal. 2019;28(2):198–205.
529. Tesfa A, Bizuneh AD, Tesfaye T, Gebru AA, Ayene YY, Tamene BA. Assessment of knowledge, attitude and practice towards emergency contraceptive methods among female students in Seto Semero high school, Jimma town, south west Ethiopia. Sci J Public Heal. 2015;3(4):478–86.
530. Hoque ME. Reported risky sexual practices amongst female undergraduate students in KwaZulu-Natal, South Africa. African J Prim Heal care Fam Med. 2011;3(1):1–6.
531. Maharaj P. Male attitudes to family planning in the era of HIV/AIDS: evidence from KwaZulu-Natal, South Africa. J South Afr Stud. 2001;27(2):245–57.
532. Greene S, Ion A, Kwaramba G, Smith S, Loutfy MR. “Why are you pregnant? What were you thinking?”: How women navigate experiences of HIV-related stigma in medical settings during pregnancy and birth. Soc Work Health Care. 2016;55(2):161–79.
533. Tenkorang EY. Intimate partner violence and the sexual and reproductive health outcomes of women in Ghana. Heal Educ Behav. 2019;46(6):969–80.
534. DeJong J, Jawad R, Mortagy I, Shepard B. The sexual and reproductive health of young people in the Arab countries and Iran. Reprod Health Matters. 2005;13(25):49–59.
535. Tepper NK, Curtis KM, Jamieson DJ, Marchbanks PA. Update to CDC’s US Medical Eligibility Criteria for Contraceptive Use, 2010: revised recommendations for the use of hormonal contraception among women at high risk for HIV infection or infected with HIV. MMWR Morb Mortal Wkly Rep. 2012;61(24).
536. Bobrova N, Sergeev O, Grechukhina T, Kapiga S. Social‐cognitive predictors of consistent condom use among young people in Moscow. Perspect Sex Reprod Health. 2005;37(4):174–8.
537. Organization WH. Making the case for interventions linking sexual and reproductive health and HIV in proposals to the Global Fund to Fight AIDS, Tuberculosis and Malaria. World Health Organization; 2010.
538. Liamputtong P. Women, motherhood, and living with HIV/AIDS: an introduction. In: Women, Motherhood and Living with HIV/AIDS: A Cross-Cultural Perspective. Springer; 2013. p. 1–24.
539. Okereke CI. Unmet reproductive health needs and health-seeking behaviour of adolescents in Owerri, Nigeria. Afr J Reprod Health. 2010;14(1).
540. Mshweshwe-Pakela NT, Matlakala MC, Mbengo F. Attitudes to, and knowledge and use of contraception among female learners attending a high school in Mdantsane. Afr J Nurs Midwifery. 2017;19(1):170–89.
541. Mullu G, Gizachew A, Amare D, Alebel A, Wagnew F, Tiruneh C, et al. Prevalence of gender based violence and associated factors among female students of Menkorer high school in Debre Markos town, Northwest Ethiopia. Science (80- ). 2015;3(1):67–74.
542. Ewunetie AA, Alemayehu M, Endalew B, Abiye H, Gedif G, Simieneh MM. Sexual and reproductive health problems and needs of street youths in East Gojjam Zone Administrative Towns, Ethiopia: Exploratory qualitative study. Adolesc Health Med Ther. 2022;55–66.
543. K Shetty A. Epidemiology of HIV infection in women and children: a global perspective. Curr HIV Res. 2013;11(2):81–92.
544. Bharat S, Mahendra VS. Meeting the sexual and reproductive health needs of people living with HIV: challenges for health care providers. Reprod Health Matters. 2007;15(sup29):93–112.
545. Oyieke JBO, Obore S, Kigondu CS. Millennium development goal 5: a review of maternal mortality at the Kenyatta National Hospital, Nairobi. East Afr Med J. 2006;83(1):4–9.
546. Omoro T, Gray SC, Otieno G, Mbeda C, Phillips-Howard PA, Hayes T, et al. Teen pregnancy in rural western Kenya: a public health issue. Int J Adolesc Youth. 2018;23(4):399–408.
547. Hale F, Vazquez M. Violence against women living with HIV/AIDS: A background paper. Washingt DC Dev Connect. 2011;
548. Allen RH. The role of family planning in poverty reduction. Obstet Gynecol. 2007;110(5):999–1002.
549. Nedjat S, Moazen B, Rezaei F, Hajizadeh S, Majdzadeh R, Setayesh HR, et al. Sexual and reproductive health needs of HIV-positive people in Tehran, Iran: a mixed-method descriptive study. Int J Heal Policy Manag. 2015;4(9):591.
550. Oindo ML. Contraception and sexuality among the youth in Kisumu, Kenya. Afr Health Sci. 2002;2(1):33–9.
551. Pellowski JA, Price DM, Harrison AD, Tuthill EL, Myer L, Operario D, et al. A systematic review and meta-analysis of antiretroviral therapy (ART) adherence interventions for women living with HIV. AIDS Behav. 2019;23:1998–2013.
552. Bankole A, Biddlecom A, Singh S, Guiella G, Zulu E. Sexual behavior, knowledge and information sources of very young adolescents in four sub-Saharan African countries. Afr J Reprod Health. 2007;11(3):28–43.
553. Matseke G, Rodriguez VJ, Peltzer K, Jones D. Intimate partner violence among HIV positive pregnant women in South Africa. J Psychol Africa. 2016;26(3):259–66.
554. Chersich MF, Rees H V. Vulnerability of women in southern Africa to infection with HIV: biological determinants and priority health sector interventions. Aids. 2008;22:S27–40.
555. Juma M, Alaii J, Bartholomew LK, Askew I, Van den Born B. Understanding orphan and non-orphan adolescents’ sexual risks in the context of poverty: a qualitative study in Nyanza Province, Kenya. BMC Int Health Hum Rights. 2013;13:1–8.
556. Kriel Y, Milford C, Cordero J, Suleman F, Beksinska M, Steyn P, et al. Male partner influence on family planning and contraceptive use: perspectives from community members and healthcare providers in KwaZulu-Natal, South Africa. Reprod Health. 2019;16:1–15.
557. Moradi F, Balaghi Z, Joulaei H, Zare N, Mohammadi S, Moghadami M. Unmet Need for Prevention of Unwanted Pregnancy in Shiraz. 2014;
558. Tessema ZT, Teshale AB, Tesema GA, Tamirat KS. Determinants of completing recommended antenatal care utilization in sub-Saharan from 2006 to 2018: evidence from 36 countries using Demographic and Health Surveys. BMC Pregnancy Childbirth. 2021;21:1–12.
559. Bazant ES, Koenig MA. Women’s satisfaction with delivery care in Nairobi’s informal settlements. Int J Qual Heal Care. 2009;21(2):79–86.
560. Greene S, Ion A, Elston D, Kwaramba G, Smith S, Carvalhal A, et al. “why aren’t you breastfeeding?”: how mothers living with HIV talk about infant feeding in a “breast is best” world. Health Care Women Int. 2015;36(8):883–901.
561. Nkosi M. Male circumcision as an HIV prevention strategy and implications for woMen’s sexual and reproductive health rights. Agenda. 2008;22(75):141–54.
562. Weinrib R, Minnis A, Agot K, Ahmed K, Owino F, Manenzhe K, et al. End-users’ poduct preference across three multipurpose prevention technology delivery forms: baseline results from young women in Kenya and South Africa. AIDS Behav. 2018;22:133–45.
563. Osinde MO, Kaye DK, Kakaire O. Intimate partner violence among women with HIV infection in rural Uganda: critical implications for policy and practice. BMC Womens Health. 2011;11:1–7.
564. Adhikari R. Factors affecting awareness of emergency contraception among college students in Kathmandu, Nepal. BMC Womens Health. 2009;9:1–5.
565. Ward MC. A different disease: HIV/AIDS and health care for women in poverty. Cult Med Psychiatry. 1993;17:413–30.
566. Maina WK, Kim AA, Rutherford GW, Harper M, K’Oyugi BO, Sharif S, et al. Kenya AIDS Indicator Surveys 2007 and 2012: implications for public health policies for HIV prevention and treatment. JAIDS J Acquir Immune Defic Syndr. 2014;66:S130–7.
567. Birungi H, Obare F, Mugisha JF, Evelia H, Nyombi J. Preventive service needs of young people perinatally infected with HIV in Uganda. AIDS Care. 2009;21(6):725–31.
568. Renzaho AMN, Kamara JK, Georgeou N, Kamanga G. Sexual, reproductive health needs, and rights of young people in slum areas of Kampala, Uganda: a cross sectional study. PLoS One. 2017;12(1):e0169721.
569. Obiyan MO, Olaleye AO, Oyinlola FF, Folayan MO. Factors associated with pregnancy and induced abortion among street-involved female adolescents in two Nigeria urban cities: a mixed-method study. BMC Health Serv Res. 2023;23(1):25.
570. Joyce C, Keraka ÂM, Njagi J. Assessment of the knowledge on pre conception care among women of reproductive age in Ruiru sub-county, Kiambu county, Kenya. Glob J Heal Sci. 2018;3(1):82–100.
571. Mutsindikwa T, Ashipala DO, Tomas N, Endjala T. Knowledge, Attitudes and Practices of Contraception among tertiary students at the University Campus in Namibia. Glob J Heal Sci. 2019;11:180.
572. Musyimi CW, Mutiso VN, Nyamai DN, Ebuenyi I, Ndetei DM. Suicidal behavior risks during adolescent pregnancy in a low-resource setting: A qualitative study. PLoS One. 2020;15(7):e0236269.
573. Tadesse G, Yakob B. Risky sexual behaviors among female youth in Tiss Abay, a semi-urban area of the Amhara Region, Ethiopia. PLoS One. 2015;10(3):e0119050.
574. Roudi-Fahimi F. Women’s reproductive health in the Middle East and North Africa. Population Reference Bureau Washington, DC; 2003.
575. Harrington EK, Dworkin S, Withers M, Onono M, Kwena Z, Newmann SJ. Gendered power dynamics and women’s negotiation of family planning in a high HIV prevalence setting: a qualitative study of couples in western Kenya. Cult Health Sex. 2016;18(4):453–69.
576. Iran S, Moradi F. Unmet Need for Prevention of Unwanted Pregnancy in Shiraz.
577. Ezegbe C, Stephenson N. The reach and limits of the US president’s emergency plan for aids relief (PEPFAR) funding of prevention of mother-to-child transmission (PMTCT) of HIV in Nigeria. Afr J Reprod Health. 2012;16(1).
578. Mulugeta Y, Berhane Y. Factors associated with pre-marital sexual debut among unmarried high school female students in bahir Dar town, Ethiopia: cross-sectional study. Reprod Health. 2014;11:1–6.
579. Usman I, Adesina A, Usman S, Tshuma N, Olubayo G, Fatunmbi O, et al. Factors Associated with Inconsisitent Female Condom Use among Sexually Active Young Persons in Western Nigeria. Asian J Res Med Pharm Sci. 2017;1(3):1–6.
580. Folayan MO, Harrison A, Odetoyinbo M, Brown B. Tackling the sexual and reproductive health and rights of adolescents living with HIV/AIDS: a priority need in Nigeria. Afr J Reprod Health. 2014;18(1):102–8.
581. Raniga T, Mathe S. Private lives, public issues: childbearing experiences of adolescent mothers in the era of HIV and AIDS in Bhambayi, KwaZulu-Natal, South Africa. Soc Work W. 2011;47(3).
582. Ahenda D. Effectiveness Of Koch Fm community Radio Programmes In the Prevention Of Unintended Pregnancies Among Young Women In Korogocho Informal Settlement. University of Nairobi; 2018.
583. Chukwu LC, Onyeonoro UU, Ikechebelu JI. Knowledge and practice of prevention of maternal to child transmission among HIV positive women of reproductive age in a tertiary hospital, south east Nigeria. J Community Med Prim Heal Care. 2010;22(1–2).
584. Speizer IS, Fotso JC, Davis JT, Saad A, Otai J. Timing and circumstances of first sex among female and male youth from select urban areas of Nigeria, Kenya, and Senegal. J Adolesc Heal. 2013;53(5):609–16.
585. Sofolahan YA, Airhihenbuwa CO. Childbearing decision making: A qualitative study of women living with HIV/AIDS in Southwest Nigeria. AIDS Res Treat. 2012;2012(1):478065.
586. Loutfy M, Johnson M, Walmsley S, Samarina A, Vasquez P, Hao-Lan H, et al. The association between HIV disclosure status and perceived barriers to care faced by women living with HIV in Latin America, China, Central/Eastern Europe, and Western Europe/Canada. AIDS Patient Care STDS. 2016;30(9):435–44.
587. Haram L. 10.‘Prostitutes’ or Modern Women? Negotiating Respectability in Northern Tanzania. Re-thinking Sex Africa. 2004;211.
588. McIntyre P, Organization WH. Pregnant adolescents: delivering on global promise of hope. 2006;
589. Izugbara CO, Egesa C, Okelo R. ‘High profile health facilities can add to your trouble’: women, stigma and un/safe abortion in Kenya. Soc Sci Med. 2015;141:9–18.
590. Deering KN, Logie C, Krüsi A, Ranville F, Braschel M, Duff P, et al. Prevalence and correlates of HIV stigma among women living with HIV in metro Vancouver, Canada. AIDS Behav. 2021;25(6):1688–98.
591. Saul J, Bachman G, Allen S, Toiv NF, Cooney C, Beamon T. The DREAMS core package of interventions: a comprehensive approach to preventing HIV among adolescent girls and young women. PLoS One. 2018;13(12):e0208167.
592. Mbonile L, Kayombo EJ. Assessing acceptability of parents/guardians of adolescents towards introduction of sex and reproductive health education in schools at Kinondoni Municipal in Dar es Salaam city. 2008;
593. Singh S, Prada E, Mirembe F, Kiggundu C. The incidence of induced abortion in Uganda. Int Fam Plan Perspect. 2005;183–91.
594. Becquet V, Nouaman M, Plazy M, Masumbuko J-M, Anoma C, Kouame S, et al. Sexual health needs of female sex workers in Côte d’Ivoire: a mixed-methods study to prepare the future implementation of pre-exposure prophylaxis (PrEP) for HIV prevention. BMJ Open. 2020;10(1):e028508.
595. Salih NA, Metaferia H, Reda AA, Biadgilign S. Premarital sexual activity among unmarried adolescents in northern Ethiopia: a cross-sectional study. Sex Reprod Healthc. 2015;6(1):9–13.
596. Todd CS, Nasir A, Raza Stanekzai M, Scott PT, Strathdee SA, Botros BA, et al. Contraceptive utilization and pregnancy termination among female sex workers in Afghanistan. J Women’s Heal. 2010;19(11):2057–62.
597. Vaina A, Perdikaris P. School-based sex education among adolescents worldwide: Interventions for the prevention of STIs and unintended pregnancies. Br J Child Heal. 2022;3(5):229–42.
598. Rodney P, Ndjakani Y, Ceesay FK, Wilson NO. Addressing the impact of HIV/AIDS on women and children in sub-Saharan Africa: PEPFAR, the US strategy. Afr Today. 2010;57(1):64–76.
599. Tessema M, Bayu H. Knowledge, attitude and practice on emergency contraception and associated factors among female students of Debre-Markos University, Debre-Markos Town, East Gojam Zone, North West Ethiopia, 2013. Glob J Med Res. 2015;15(1):1–8.
600. Miralles C, Mardarescu M, Sherr L. What do we know about the situation of women living with HIV in Europe? Antivir Ther. 2013;18(2_suppl):11–7.
601. Endriyas M, Eshete A, Mekonnen E, Misganaw T, Shiferaw M, Ayele S. Contraceptive utilization and associated factors among women of reproductive age group in Southern Nations Nationalities and Peoples’ Region, Ethiopia: cross-sectional survey, mixed-methods. Contracept Reprod Med. 2017;2:1–9.
602. Ezeh AC, Kodzi I, Emina J. Reaching the urban poor with family planning services. Stud Fam Plann. 2010;41(2):109–16.
603. Sinyange N, Sitali L, Jacobs C, Musonda P, Michelo C. Factors associated with late antenatal care booking: population based observations from the 2007 Zambia demographic and health survey. Pan Afr Med J. 2016;25.
604. Barker GK, Rich S. Influences on adolescent sexuality in Nigeria and Kenya: Findings from recent focus-group discussions. Stud Fam Plann. 1992;23(3):199–210.
605. KUMAR R, PITTROF R. Human Immunodeficiency Virus and Contraception. Mind Gaps Cases Gynaecol Sex Reprod Heal Mind Gaps Cases Gynaecol Sex Reprod Heal E-b. 2021;165.
606. Turi E, Merga BT, Fekadu G, Abajobir AA. Why too soon? Early initiation of sexual intercourse among adolescent females in Ethiopia: evidence from 2016 Ethiopian Demographic and Health Survey. Int J Womens Health. 2020;269–75.
607. Elmore-Meegan M, Conroy RM, Agala CB. Sex workers in Kenya, numbers of clients and associated risks: an exploratory survey. Reprod Health Matters. 2004;12(23):50–7.
608. Lüllmann H, Mohr K. Color atlas of pharmacology. Thieme Stuttgart; 1999.
609. Gribble J, Haffey J. Reproductive health in sub-Saharan Africa. Popul Ref Bur. 2008;8.
610. Haberland N, Rogow D. Sexuality education: emerging trends in evidence and practice. J Adolesc Heal. 2015;56(1):S15–21.
611. Agbemenu KA. A CRITICAL EXAMINATION OF COMPREHENSIVE SEX EDUCATION PROGRAMMES TARGETING GIRLS BETWEEN THE AGES OF 14-18, IN KENYA, EAST AFRICA. University of Pittsburgh; 2009.
612. Muhwava LS, Morojele N, London L. Psychosocial factors associated with early initiation and frequency of antenatal care (ANC) visits in a rural and urban setting in South Africa: a cross-sectional survey. BMC Pregnancy Childbirth. 2016;16:1–9.
613. Upadhyay UD, Gipson JD, Withers M, Lewis S, Ciaraldi EJ, Fraser A, et al. Womens empowerment and fertility: a review of the literature. Soc Sci Med. 2014;115:111–20.
614. Demissie TW, Nigatu AM, Beyene GM. Assessment of emergency contraceptives utilization and associated factors among female college students at Debre Tabor town. Contracept Reprod Med. 2020;5:1–9.
615. Mbugua N. Factors inhibiting educated mothers in Kenya from giving meaningful sex-education to their daughters. Soc Sci Med. 2007;64(5):1079–89.
616. Abdissa B, Addisie M, Seifu W. Premarital Sexual practices, consequences and associated factors among regular undergraduate female students in Ambo University, Oromia Regional State, Central Ethiopia, 2015. Heal Sci J. 2017;11(1):1.
617. Popoola BI. Sex-negotiation strategies and safer-sex practices among married women in South-western Nigeria. Sex Relatsh Ther. 2009;24(3–4):261–70.
618. Colombini M, James C, Ndwiga C, Mayhew SH. The risks of partner violence following HIV status disclosure, and health service responses: narratives of women attending reproductive health services in Kenya. African J Reprod Gynaecol Endosc. 2016;19(1).
619. Maternal N. Rapid Assessment of Maternal, Newborn and Child Health, Family Planning and HIV/AIDS Integration in Malawi. 2014;
620. Solomon O. Factors Associated with the Utilisation of Family Planning Services among Women of Reproductive Age (15-49 Years) Attending Hoima Regional Referral Hospital.
621. Osok J, Kigamwa P, Stoep A Vander, Huang K-Y, Kumar M. Depression and its psychosocial risk factors in pregnant Kenyan adolescents: a cross-sectional study in a community health Centre of Nairobi. BMC Psychiatry. 2018;18:1–10.
622. Bereda G. Knowledge, Attitude, and Practice towards Emergency Contraceptives use Among Negelle Health Sciience College Female Students, South Eastern, Ethiopia: a Descriptive Cross-Sectional Study, 2021. J Women Heal Care Issues. 2022;5(1).
623. Hoque ME, Ghuman S. Knowledge, practices, and attitudes of emergency contraception among female university students in KwaZulu-Natal, South Africa. 2012;
624. Najmabadi KM, Sharifi F. Sexual education and women empowerment in health: a review of the literature. Int J women’s Heal Reprod Sci. 2019;7(2):150–5.
625. Nanyonga B. Voices of Female Youths Living with HIV/AIDS on their Experiences regarding access and Utilisation of Contraceptives: A case of Kawempe Division Kampala City, Uganda. 2018;
626. Jijini A. HIV/AIDS TECHNICAL BRIEF.
627. Desai M, Phillips-Howard PA, Odhiambo FO, Katana A, Ouma P, Hamel MJ, et al. An analysis of pregnancy-related mortality in the KEMRI/CDC health and demographic surveillance system in western Kenya. PLoS One. 2013;8(7):e68733.
628. Durojaye E. Realizing access to sexual health information and services for adolescents through the protocol to the African Charter on the rights of women. Wash Lee J Civ Rts Soc Just. 2009;16:135.
629. Meseret M, Shimeka A, Bekele A. Research Article Incidence and Predictors of Pregnancy among Women on ART in Debre Markos Referral Hospital, Northwest Ethiopia: A Five-Year Retrospective Cohort Study. 2017;
630. Gelagay AA, Koye DN, Yeshita HY. Demand for long acting contraceptive methods among married HIV positive women attending care at public health facilities at Bahir Dar City, Northwest Ethiopia. Reprod Health. 2015;12:1–9.
631. Juliastuti D, Dean J, Fitzgerald L. Sexual and reproductive health of women living with HIV in Muslim-majority countries: a systematic mixed studies review. BMC Int Health Hum Rights. 2020;20:1–12.
632. Manzini N. Sexual initiation and childbearing among adolescent girls in KwaZulu Natal, South Africa. Reprod Health Matters. 2001;9(17):44–52.
633. Khan MN, Harris ML, Shifti DM, Laar AS, Loxton D. Effects of unintended pregnancy on maternal healthcare services utilization in low-and lower-middle-income countries: systematic review and meta-analysis. Int J Public Health. 2019;64:743–54.
634. Ochako R, Temmerman M, Mbondo M, Askew I. Determinants of modern contraceptive use among sexually active men in Kenya. Reprod Health. 2017;14:1–15.
635. Ohnishi M, Leshabari S, Tanaka J, Nishihara M. Factors associated with the awareness of contraceptive methods, understanding the prevention of HIV/AIDS and the perception of HIV/AIDS risk among secondary school students in Dar es Salaam, Tanzania. J Rural Med. 2020;15(4):155–63.
636. Araya BM, Solomon AA, Gebreslasie KZ, Gudayu TW, Anteneh KT. The role of counseling on modern contraceptive utilization among HIV positive women: the case of Northwest Ethiopia. BMC Womens Health. 2018;18:1–9.
637. Yam EA, Kidanu A, Burnett‐Zieman B, Pilgrim N, Okal J, Bekele A, et al. Pregnancy experiences of female sex workers in Adama City, Ethiopia: Complexity of partner relationships and pregnancy intentions. Stud Fam Plann. 2017;48(2):107–19.
638. Ferede TA, Muluneh AG, Wagnew A, Walle AD. Prevalence and associated factors of early sexual initiation among youth female in sub-Saharan Africa: a multilevel analysis of recent demographic and health surveys. BMC Womens Health. 2023;23(1):147.
639. Bafana TNS. Factors influencing contraceptive use and unplanned pregnancy in a South African population. 2010.
640. Mbongueh MC, Nicholas T, Ndum AC, Gisèle EL, Nguedia A, Clement J. Unintended pregnancy and sexually transmissible infections amongst adolescents and young adults in Douala IV municipality, Cameroon: Prevalence, knowledge, and associated factors. 2023;
641. Khu NH, Vwalika B, Karita E, Kilembe W, Bayingana RA, Sitrin D, et al. Fertility goal-based counseling increases contraceptive implant and IUD use in HIV-discordant couples in Rwanda and Zambia. Contraception. 2013;88(1):74–82.
642. Haile D, Lagebo B. Magnitude of dual contraceptive method utilization and the associated factors among women on antiretroviral treatment in Wolaita zone, Southern Ethiopia. Heliyon. 2022;8(6).
643. Credé S, Hoke T, Constant D, Green MS, Moodley J, Harries J. Factors impacting knowledge and use of long acting and permanent contraceptive methods by postpartum HIV positive and negative women in Cape Town, South Africa: a cross-sectional study. BMC Public Health. 2012;12:1–9.
644. Wasie B, Belyhun Y, Moges B, Amare B. Effect of emergency oral contraceptive use on condom utilization and sexual risk taking behaviours among university students, Northwest Ethiopia: a cross-sectional study. BMC Res Notes. 2012;5:1–9.
645. Feyissa TR, Melka AS. Demand for modern family planning among married women living with HIV in western Ethiopia. PLoS One. 2014;9(11):e113008.
646. Young IC, Benhabbour SR. Multipurpose prevention technologies: oral, parenteral, and vaginal dosage forms for prevention of HIV/STIs and unplanned pregnancy. Polymers (Basel). 2021;13(15):2450.
647. Tirado V, Orsini N, Strömdahl S, Hanson C, Ekström AM. Knowledge gaps related to HIV and condom use for preventing pregnancy: a cross-sectional study among migrants in Sweden. BMC Public Health. 2024;24(1):2334.
648. Amuyunzu-Nyamongo M, Tendo-Wambua L, Babishangire B, Nyagero J, Yitbarek N, Matasha M, et al. Barriers to behaviour change as a response to STD including HIV/AIDS: the East African experience. In Citeseer; 1999.
649. Chanda P, JO EK, Ochieng LA. FACTORS AFFECTING UPTAKE OF CONTRACEPTIVES AMONG WOMEN AGED 15-25 IN THE CONTEXT OF EARLY PREGNANCY AND HIV/AIDS PREVENTION IN UGANDA.
650. Dibaba Y, Fantahun M, Hindin MJ. The association of unwanted pregnancy and social support with depressive symptoms in pregnancy: evidence from rural Southwestern Ethiopia. BMC Pregnancy Childbirth. 2013;13:1–8.
651. Türmen T. Gender and HIV/aids. Int J Gynecol Obstet. 2003;82(3):411–8.
652. Shrikhande L. HIV in women in South-East Asia and India. Population (Paris). 2008;423–586.
653. Schelar E, Polis CB, Essam T, Looker KJ, Bruni L, Chrisman CJ, et al. Multipurpose prevention technologies for sexual and reproductive health: mapping global needs for introduction of new preventive products. Contraception. 2016;93(1):32–43.
654. Harrington BJ, Pence BW, John M, Melhado CG, Phulusa J, Mthiko B, et al. Prevalence and factors associated with antenatal depressive symptoms among women enrolled in Option B+ antenatal HIV care in Malawi: a cross-sectional analysis. J Ment Heal. 2019;28(2):198–205.
655. Tesfa A, Bizuneh AD, Tesfaye T, Gebru AA, Ayene YY, Tamene BA. Assessment of knowledge, attitude and practice towards emergency contraceptive methods among female students in Seto Semero high school, Jimma town, south west Ethiopia. Sci J Public Heal. 2015;3(4):478–86.
656. Hoque ME. Reported risky sexual practices amongst female undergraduate students in KwaZulu-Natal, South Africa. African J Prim Heal care Fam Med. 2011;3(1):1–6.
657. Maharaj P. Male attitudes to family planning in the era of HIV/AIDS: evidence from KwaZulu-Natal, South Africa. J South Afr Stud. 2001;27(2):245–57.
658. Greene S, Ion A, Kwaramba G, Smith S, Loutfy MR. “Why are you pregnant? What were you thinking?”: How women navigate experiences of HIV-related stigma in medical settings during pregnancy and birth. Soc Work Health Care. 2016;55(2):161–79.
659. Tenkorang EY. Intimate partner violence and the sexual and reproductive health outcomes of women in Ghana. Heal Educ Behav. 2019;46(6):969–80.
660. DeJong J, Jawad R, Mortagy I, Shepard B. The sexual and reproductive health of young people in the Arab countries and Iran. Reprod Health Matters. 2005;13(25):49–59.
661. Tepper NK, Curtis KM, Jamieson DJ, Marchbanks PA. Update to CDC’s US Medical Eligibility Criteria for Contraceptive Use, 2010: revised recommendations for the use of hormonal contraception among women at high risk for HIV infection or infected with HIV. MMWR Morb Mortal Wkly Rep. 2012;61(24).
662. Bobrova N, Sergeev O, Grechukhina T, Kapiga S. Social‐cognitive predictors of consistent condom use among young people in Moscow. Perspect Sex Reprod Health. 2005;37(4):174–8.
663. Organization WH. Making the case for interventions linking sexual and reproductive health and HIV in proposals to the Global Fund to Fight AIDS, Tuberculosis and Malaria. World Health Organization; 2010.
664. Liamputtong P. Women, motherhood, and living with HIV/AIDS: an introduction. In: Women, Motherhood and Living with HIV/AIDS: A Cross-Cultural Perspective. Springer; 2013. p. 1–24.
665. Okereke CI. Unmet reproductive health needs and health-seeking behaviour of adolescents in Owerri, Nigeria. Afr J Reprod Health. 2010;14(1).
666. Mshweshwe-Pakela NT, Matlakala MC, Mbengo F. Attitudes to, and knowledge and use of contraception among female learners attending a high school in Mdantsane. Afr J Nurs Midwifery. 2017;19(1):170–89.
667. Mullu G, Gizachew A, Amare D, Alebel A, Wagnew F, Tiruneh C, et al. Prevalence of gender based violence and associated factors among female students of Menkorer high school in Debre Markos town, Northwest Ethiopia. Science (80- ). 2015;3(1):67–74.
668. Ewunetie AA, Alemayehu M, Endalew B, Abiye H, Gedif G, Simieneh MM. Sexual and reproductive health problems and needs of street youths in East Gojjam Zone Administrative Towns, Ethiopia: Exploratory qualitative study. Adolesc Health Med Ther. 2022;55–66.
669. K Shetty A. Epidemiology of HIV infection in women and children: a global perspective. Curr HIV Res. 2013;11(2):81–92.
670. Bharat S, Mahendra VS. Meeting the sexual and reproductive health needs of people living with HIV: challenges for health care providers. Reprod Health Matters. 2007;15(sup29):93–112.
671. Oyieke JBO, Obore S, Kigondu CS. Millennium development goal 5: a review of maternal mortality at the Kenyatta National Hospital, Nairobi. East Afr Med J. 2006;83(1):4–9.
672. Omoro T, Gray SC, Otieno G, Mbeda C, Phillips-Howard PA, Hayes T, et al. Teen pregnancy in rural western Kenya: a public health issue. Int J Adolesc Youth. 2018;23(4):399–408.
673. Hale F, Vazquez M. Violence against women living with HIV/AIDS: A background paper. Washingt DC Dev Connect. 2011;
674. Ochieng Arunda M, Agardh A, Larsson M, Asamoah BO. Survival patterns of neonates born to adolescent mothers and the effect of pregnancy intentions and marital status on newborn survival in Kenya, Uganda, and Tanzania, 2014–2016. Glob Health Action. 2022;15(1):2101731.
675. Ma Q, Ono-Kihara M, Cong L, Xu G, Pan X, Zamani S, et al. Unintended pregnancy and its risk factors among university students in eastern China. Contraception. 2008;77(2):108–13.
676. Mokwena K, Bogale YR. Fertility intention and use of contraception among women living with the human immunodeficiency virus in Oromia Region, Ethiopia. South African Fam Pract. 2017;59(1):46–51.
677. Bakibinga P, Matanda DJ, Ayiko R, Rujumba J, Muiruri C, Amendah D, et al. Pregnancy history and current use of contraception among women of reproductive age in Burundi, Kenya, Rwanda, Tanzania and Uganda: analysis of demographic and health survey data. BMJ Open. 2016;6(3):e009991.
678. Okigbo CC, Speizer IS. Determinants of sexual activity and pregnancy among unmarried young women in urban Kenya: a cross-sectional study. PLoS One. 2015;10(6):e0129286.
679. Ampt FH, Willenberg L, Agius PA, Chersich M, Luchters S, Lim MSC. Incidence of unintended pregnancy among female sex workers in low-income and middle-income countries: a systematic review and meta-analysis. BMJ Open. 2018;8(9):e021779.
680. Duff P, Muzaaya G, Muldoon K, Dobrer S, Akello M, Birungi J, et al. High rates of unintended pregnancies among young women sex Workers in Conflict-affected Northern Uganda: the social contexts of brothels/lodges and substance use. Afr J Reprod Health. 2017;21(2):64–72.
681. Ngugi EW, Kim AA, Nyoka R, Mukui I, Ng’eno B, Rutherford GW, et al. Contraceptive practices and fertility desires among HIV-infected and uninfected women in Kenya: results from a nationally representative study. JAIDS J Acquir Immune Defic Syndr. 2014;66:S75–81.
682. Mubangizi L. Examining the association between future pregnancy intentions, contraceptive use and repeat pregnancies among women living with HIV in Cape Town, South Africa. 2020;
683. Bankole A, Singh S, Hussain R, Oestreicher G. Condom use for preventing STI/HIV and unintended pregnancy among young men in Sub-Saharan Africa. Am J Mens Health. 2009;3(1):60–78.
684. Dhakal S, Song JS, Shin DE, Lee TH, So AY, Nam EW. Unintended pregnancy and its correlates among currently pregnant women in the Kwango District, Democratic Republic of the Congo. Reprod Health. 2016;13:1–7.
685. Izugbara C, Egesa C. The management of unwanted pregnancy among women in Nairobi, Kenya. Int J Sex Heal. 2014;26(2):100–12.
686. Wall KM, Kilembe W, Vwalika B, Haddad LB, Khu NH, Brill I, et al. Optimizing prevention of HIV and unplanned pregnancy in discordant African couples. J women’s Heal. 2017;26(8):900–10.
687. Aragaw FM, Amare T, Teklu RE, Tegegne BA, Alem AZ. Magnitude of unintended pregnancy and its determinants among childbearing age women in low and middle-income countries: evidence from 61 low and middle income countries. Front Reprod Heal. 2023;5:1113926.
688. Chukwunyere AP, Stella KA. Unintended pregnancy among undergraduate students at a select university, Eastern Cape, South Africa: effects, influences, outcomes and solutions. Gend Behav. 2019;17(4):14272–86.
689. De Bruyn M. Living with HIV: challenges in reproductive health care in South Africa. Afr J Reprod Health. 2004;92–8.
690. Awiti Ujiji O, Ekström AM, Ilako F, Indalo D, Wamalwa D, Rubenson B. Reasoning and deciding PMTCT-adherence during pregnancy among women living with HIV in Kenya. Cult Health Sex. 2011;13(7):829–40.
691. Hailegebreal S, Gilano G, Seboka BT, Sidelil H, Awol SM, Haile Y, et al. Prevalence and associated factors of early sexual initiation among female youth in East Africa: further analysis of recent demographic and health survey. BMC Womens Health. 2022;22(1):304.
692. Arunda MO, Agardh A, Larsson M, Asamoah BO. Survival patterns of neonates born to adolescent mothers and the effect of pregnancy intentions and marital status on newborn survival in Kenya, Uganda, and Tanzania, 2014–2016. Glob Health Action. 2022;15(1).
693. Ddumba-Nyanzi I, Kaawa-Mafigiri D, Johannessen H. Barriers to communication between HIV care providers (HCPs) and women living with HIV about child bearing: A qualitative study. Patient Educ Couns. 2016;99(5):754–9.
694. King R, Khana K, Nakayiwa S, Katuntu D, Homsy J, Lindkvist P, et al. “Pregnancy comes accidentally-like it did with me”: reproductive decisions among women on ART and their partners in rural Uganda. BMC Public Health. 2011;11:1–11.
695. Anand A, Shiraishi RW, Bunnell RE, Jacobs K, Solehdin N, Abdul-Quader AS, et al. Knowledge of HIV status, sexual risk behaviors and contraceptive need among people living with HIV in Kenya and Malawi. Aids. 2009;23(12):1565–73.
696. Haddad LB, Feldacker C, Jamieson DJ, Tweya H, Cwiak C, Chaweza T, et al. Pregnancy prevention and condom use practices among HIV-infected women on antiretroviral therapy seeking family planning in Lilongwe, Malawi. PLoS One. 2015;10(3):e0121039.
697. Damian DJ, George JM, Martin E, Temba B, Msuya SE. Prevalence and factors influencing modern contraceptive use among HIV-positive women in Kilimanjaro region, northern Tanzania. Contracept Reprod Med. 2018;3:1–9.
698. Bankole A, Keogh S, Akinyemi O, Dzekedzeke K, Awolude O, Adewole I. Differences in unintended pregnancy, contraceptive use and abortion by HIV status among women in Nigeria and Zambia. Int Perspect Sex Reprod Health. 2014;40(1):28–38.
699. Okoli ML, Alao S, Ojukwu S, Emechebe NC, Ikhuoria A, Kip KE. Predictive and spatial analysis for estimating the impact of sociodemographic factors on contraceptive use among women living with HIV/AIDS (WLWHA) in Kenya: Implications for policies and practice. BMJ Open. 2019;9(1):e022221.
700. Mageda K, Mohamed MA, Kulemba K. Incidence and determinants of pregnancy among women receiving HAART in Simiyu region: 14‑year retrospective follow‑up. Trop J Obstet Gynaecol. 2020;37(1):38–45.
701. Olowookere SA, Abioye-Kuteyi EA, Bamiwuye SO. Fertility intentions of people living with HIV/AIDS at Osogbo, Southwest Nigeria. Eur J Contracept Reprod Heal Care. 2013;18(1):61–7.
702. Tusiime S, Musinguzi G, Tinkitina B, Mwebaza N, Kisa R, Anguzu R, et al. Prevalence of sexual coercion and its association with unwanted pregnancies among young pregnant females in Kampala, Uganda: a facility based cross-sectional study. BMC Womens Health. 2015;15:1–12.
703. Machiyama K, Mumah JN, Mutua M, Cleland J. Childbearing desires and behaviour: a prospective assessment in Nairobi slums. BMC Pregnancy Childbirth. 2019;19:1–12.
704. Ngocho JS, Watt MH, Minja L, Knettel BA, Mmbaga BT, Williams PP, et al. Depression and anxiety among pregnant women living with HIV in Kilimanjaro region, Tanzania. PLoS One. 2019;14(10):e0224515.
705. Lange KN. The impact of mental health on unintended pregnancies within HIV serodiscordant heterosexual couples in Uganda and Kenya. 2019.
706. Ekorinyang R. Utilization of contraceptives by persons living with HIV in Eastern Uganda: a cross sectional study. Reprod Health. 2015;12:1–8.
707. Awuor SO. Prevalence of Pregnancy among Adolescent Living with HIV in Muhoroni Sub County Kisumu County, Kenya. Int J Anesth Clin Med. 2021;9(1):7–10.
708. Solanke BL. Do community characteristics influence unintended pregnancies in Kenya? Malawi Med J. 2019;31(1):56–64.
709. Teklu T, Davey G. Which factors influence North Ethiopian adults’ use of dual protection from unintended pregnancy and HIV/AIDS? Ethiop J Heal Dev. 2008;22(3).
710. Chukwunyere AP, Stella KA. Unintended pregnancy among undergraduate students at a select university, Eastern Cape, South Africa: effects, influences, outcomes and solutions. Gend Behav. 2019;17(4):14272–86. 1. Josephine AM. “Motherhood is hard”: Exploring the complexities of unplanned motherhood among HIV-positive adolescents in South Africa. SAGE Open. 2019;9(2):2158244019848802.
711. Colombini M, Mutemwa R, Kivunaga J, Stackpool Moore L, Mayhew SH. Experiences of stigma among women living with HIV attending sexual and reproductive health services in Kenya: a qualitative study. BMC Health Serv Res. 2014;14:1–9.
712. De Bruyn M. Reproductive choice and women living with HIV/AIDS. Citeseer; 2002.
713. Feyissa TR, Harris ML, Loxton D. “They haven’t asked me. I haven’t told them either”: fertility plan discussions between women living with HIV and healthcare providers in western Ethiopia. Reprod Health. 2020;17:1–16.
714. Munsakul W, Lolekha R, Kowadisaiburana B, Roongpisuthipong A, Jirajariyavej S, Asavapiriyanont S, et al. Dual contraceptive method use and pregnancy intention among people living with HIV receiving HIV care at six hospitals in Thailand. Reprod Health. 2015;13:1–11.
715. Stalter RM, Amorim G, Mocello AR, Jakait B, Shepherd BE, Musick B, et al. Contraceptive implant use duration is not associated with breakthrough pregnancy among women living with HIV and using efavirenz: a retrospective, longitudinal analysis. J Int AIDS Soc. 2022;25(9):e26001.
716. Assefa N, Berhane Y, Worku A, Tsui A. The hazard of pregnancy loss and stillbirth among women in Kersa, East Ethiopia: a follow up study. Sex Reprod Healthc. 2012;3(3):107–12.
717. Izugbara CO, Ochako R, Izugbara C. Gender scripts and unwanted pregnancy among urban Kenyan women. Cult Health Sex. 2011;13(9):1031–45.
718. Liamputtong P. Women, motherhood and living with HIV/AIDS: A cross-cultural perspective. Springer Science & Business Media; 2013.
719. Faini D, Munseri P, Bakari M, Sandström E, Faxelid E, Hanson C. “I did not plan to have a baby. This is the outcome of our work”: a qualitative study exploring unintended pregnancy among female sex workers. BMC Womens Health. 2020;20:1–13.
720. Ampt FH, Lim MSC, Agius PA, L’Engle K, Manguro G, Gichuki C, et al. Effect of a mobile phone intervention for female sex workers on unintended pregnancy in Kenya (WHISPER or SHOUT): a cluster-randomised controlled trial. Lancet Glob Heal. 2020;8(12):e1534–45.
721. Awuor SO. Prevalence of Pregnancy among Adolescent Living with HIV in Muhoroni Sub County Kisumu County, Kenya. Int J Anesth Clin Med. 2021;9(1):7–10.
722. Ngocho JS, Watt MH, Minja L, Knettel BA, Mmbaga BT, Williams PP, et al. Depression and anxiety among pregnant women living with HIV in Kilimanjaro region, Tanzania. PLoS One. 2019;14(10):e0224515.
723. Lange KN. The impact of mental health on unintended pregnancies within HIV serodiscordant heterosexual couples in Uganda and Kenya. 2019.
724. Machiyama K, Mumah JN, Mutua M, Cleland J. Childbearing desires and behaviour: a prospective assessment in Nairobi slums. BMC Pregnancy Childbirth. 2019;19:1–12.
725. Organization WH. WHO recommendation on advance misoprostol distribution to pregnant women for prevention of postpartum haemorrhage. 2020;
726. Raifman J, Chetty T, Tanser F, Mutevedzi T, Matthews P, Herbst K, et al. Preventing unintended pregnancy and HIV transmission: effects of the HIV treatment cascade on contraceptive use and choice in rural KwaZulu-Natal. JAIDS J Acquir Immune Defic Syndr. 2014;67:S218–27.
727. Ahinkorah BO, Seidu A-A, Appiah F, Oduro JK, Sambah F, Baatiema L, et al. Effect of sexual violence on planned, mistimed and unwanted pregnancies among women of reproductive age in sub-Saharan Africa: A multi-country analysis of Demographic and Health Surveys. SSM-population Heal. 2020;11:100601.
728. Bakeera-Kitaka S, Smekens T, Jespers V, Wobudeya E, Loos J, Colebunders R, et al. Factors influencing the risk of becoming sexually active among HIV infected adolescents in Kampala and Kisumu, East Africa. AIDS Behav. 2019;23:1375–86.
729. Nzioka C. Unwanted pregnancy and sexually transmitted infection among young women in rural Kenya. Cult Health Sex. 2004;6(1):31–44.
730. Mantell JE, Harrison A, Hoffman S, Smit JA, Stein ZA, Exner TM. The Mpondombili project: Preventing HIV/AIDS and unintended pregnancy among rural South African school-going adolescents. Reprod Health Matters. 2006;14(28):113–22.
731. Gachigua SG, Karuga R, Ngunjiri A, Jarrahian C, Coffey PS, Kilbourne-Brook M, et al. Microarray patch for HIV prevention and as a multipurpose prevention technology to prevent HIV and unplanned pregnancy: an assessment of potential acceptability, usability, and programmatic fit in Kenya. Front Reprod Heal. 2023;5:1125159.
732. Van Zyl C, Visser MJ. Reproductive desires of men and women living with HIV: implications for family planning counselling. Reprod Biomed Online. 2015;31(3):434–42.
733. Johnson KB, Akwara P, Rutstein SO, Bernstein S. Fertility preferences and the need for contraception among women living with HIV: the basis for a joint action agenda. Aids. 2009;23:S7–17.
734. Liyew AM, Tesema GA, Alamneh TS, Worku MG, Teshale AB, Alem AZ, et al. Prevalence and determinants of anemia among pregnant women in East Africa; A multi-level analysis of recent Demographic and Health Surveys. PLoS One. 2021;16(4):e0250560.
735. Gedefaw G, Wondmieneh A, Demis A. Contraceptive Use and Method Preferences among HIV Positive Women in Ethiopia: A Systematic Review and Meta‐analysis. Biomed Res Int. 2020;2020(1):6465242.
736. Gay J, Hardee K, Croce-Galis M, Hall C. What works to meet the sexual and reproductive health needs of women living with HIV/AIDS. J Int AIDS Soc. 2011;14:1–10.
737. Steiner RJ, Black V, Rees H, Schwartz SR. Low receipt and uptake of safer conception messages in routine HIV care: findings from a prospective cohort of women living with HIV in South Africa. JAIDS J Acquir Immune Defic Syndr. 2016;72(1):105–13.
738. Ashaba S, Kaida A, Coleman JN, Burns BF, Dunkley E, O’Neil K, et al. Psychosocial challenges facing women living with HIV during the perinatal period in rural Uganda. PLoS One. 2017;12(5):e0176256.
739. Bongomin F, Chelangat M, Eriatu A, Chan Onen B, Cheputyo P, Godmercy SA, et al. Prevalence and Factors Associated with Contraceptive Use among HIV‐Infected Women of Reproductive Age Attending Infectious Disease Clinic at Gulu Regional Referral Hospital, Northern Uganda. Biomed Res Int. 2018;2018(1):9680514.
740. Kamangu AA, Myeya HE. Exploring Young Peoples’ Sexual Behaviours and the Underlying Factors in East Africa: A Review of Literature from Four Countries. J Anthropol Surv India. 2023;72(1):149–62.
741. Zewdu LB, Reta MM, Yigzaw N, Tamirat KS. Prevalence of suicidal ideation and associated factors among HIV positive perinatal women on follow-up at Gondar town health institutions, Northwest Ethiopia: a cross-sectional study. BMC Pregnancy Childbirth. 2021;21:1–9.
742. Heys J, Kipp W, Jhangri GS, Alibhai A, Rubaale T. Fertility desires and infection with the HIV: results from a survey in rural Uganda. Aids. 2009;23:S37–45.
743. De Bruyn M. Women, reproductive rights, and HIV/AIDS: Issues on which research and interventions are still needed. J Health Popul Nutr. 2006;24(4):413.
744. Arega T. HIV and Unintended Pregnancy Risk Perception and Contraceptive Use among Youth in Debre Birhan District, Ethiopia. Addis Ababa University; 2010.
745. Abubeker FA, Fanta MB, Dalton VK. Unmet Need for Contraception among HIV‐Positive Women Attending HIV Care and Treatment Service at Saint Paul’s Hospital Millennium Medical College, Addis Ababa, Ethiopia. Int J Reprod Med. 2019;2019(1):3276780.
746. Ma Q, Ono-Kihara M, Cong L, Xu G, Pan X, Zamani S, et al. Early initiation of sexual activity: a risk factor for sexually transmitted diseases, HIV infection, and unwanted pregnancy among university students in China. BMC Public Health. 2009;9:1–8.
747. Shikhansari S, Khalesi ZB, Rad EH. Factors associated with the reproductive health of women living with HIV in Iran. Eur J Obstet Gynecol Reprod Biol X. 2022;13:100136.
748. Nyanja TAN, Tulinius C. Relationships matter: contraceptive choices among HIV-positive women in Tanzania. African J AIDS Res. 2017;16(2):109–17.
749. Johnson LF, Mutemaringa T, Heekes A, Boulle A. Effect of HIV infection and antiretroviral treatment on pregnancy rates in the Western Cape province of South Africa. J Infect Dis. 2020;221(12):1953–62.
750. Nakku‐Joloba E, Pisarski EE, Wyatt MA, Muwonge TR, Asiimwe S, Celum CL, et al. Beyond HIV prevention: everyday life priorities and demand for PrEP among Ugandan HIV serodiscordant couples. African J Reprod Gynaecol Endosc. 2019;22(1).
751. Maharaj P. The dual risks of unwanted pregnancy and HIV/AIDS: the case of KwaZulu-Natal, South Africa. London School of Hygiene & Tropical Medicine; 2003.
752. Bradley H, Tsui A, Kidanu A, Gillespie D. HIV infection and contraceptive need among female Ethiopian voluntary HIV counseling and testing clients. AIDS Care. 2010;22(10):1295–304.
753. Mburu G, Ndimbii J, Ayon S, Mlewa O, Mbizvo M, Kihara C, et al. Contraceptive use among women who inject drugs: motivators, barriers, and unmet needs. Women’s Reprod Heal. 2018;5(2):99–116.
754. Adilo TM, Wordofa HM. Prevalence of fertility desire and its associated factors among 15-to 49-year-old people living with HIV/AIDS in Addis Ababa, Ethiopia: a cross-sectional study design. HIV/AIDS-Research Palliat Care. 2017;167–76.
755. Ashimi AO, Amole TG, Abubakar MY, Ugwa EA. Fertility desire and utilization of family planning methods among HIV‑positive women attending a tertiary hospital in a suburban setting in Northern Nigeria. Trop J Obstet Gynaecol. 2017;34(1):54–60.
756. Selke HM, Kimaiyo S, Sidle JE, Vedanthan R, Tierney WM, Shen C, et al. Task-shifting of antiretroviral delivery from health care workers to persons living with HIV/AIDS: clinical outcomes of a community-based program in Kenya. JAIDS J Acquir Immune Defic Syndr. 2010;55(4):483–90.
757. Druce N, Nolan A. Seizing the big missed opportunity: linking HIV and maternity care services in sub-Saharan Africa. Reprod Health Matters. 2007;15(30):190–201.
758. Schaan MM, Taylor M, Gungqisa N, Marlink R. Personal views about womanhood amongst women living with HIV in Botswana. Cult Health Sex. 2016;18(2):171–83.
759. Singh S, Bankole A, Woog V. Evaluating the need for sex education in developing countries: sexual behaviour, knowledge of preventing sexually transmitted infections/HIV and unplanned pregnancy. Sex Educ. 2005;5(4):307–31.
760. Myer L, Carter RJ, Katyal M, Toro P, El-Sadr WM, Abrams EJ. Impact of antiretroviral therapy on incidence of pregnancy among HIV-infected women in Sub-Saharan Africa: a cohort study. PLoS Med. 2010;7(2):e1000229.
761. Nakanwagi M, Bulage L, Kwesiga B, Ario AR, Birungi DA, Lukabwe I, et al. Low proportion of women who came knowing their HIV status at first antenatal care visit, Uganda, 2012–2016: a descriptive analysis of surveillance data. BMC Pregnancy Childbirth. 2020;20:1–8.
762. Kanyangarara M, Sakyi K, Laar A. Availability of integrated family planning services in HIV care and support sites in sub-Saharan Africa: a secondary analysis of national health facility surveys. Reprod Health. 2019;16:1–9.
763. Berer M. HIV/AIDS, sexual and reproductive health: intersections and implications for national programmes. Health Policy Plan. 2004;19(suppl_1):i62–70.
764. Bowring AL, Schwartz S, Lyons C, Rao A, Olawore O, Njindam IM, et al. Unmet need for family planning and experience of unintended pregnancy among female sex workers in urban Cameroon: results from a national cross-sectional study. Glob Heal Sci Pract. 2020;8(1):82–99.
765. Tibebu NS, Kassie BA, Anteneh TA, Rade BK. Depression, anxiety and stress among HIV-positive pregnant women in Ethiopia during the COVID-19 pandemic. Trans R Soc Trop Med Hyg. 2023;117(5):317–25.
766. Ofurum IC. Sexual Behaviour, Needs and Concerns Regarding Sexual and Reproductive Health among Adults Living with HIV in Sub-Saharan Africa-A Systematic Review. J Adv Med Med Res. 2021;33(11):113–32.
767. Kownaklai J, Graham M, Hayter M. Pregnancy decision making among Thai women living with HIV: a grounded theory study. Midwifery. 2022;115:103490.
768. Abay F, Yeshita HY, Mekonnen FA, Sisay M. Dual contraception method utilization and associated factors among sexually active women on antiretroviral therapy in Gondar City, northwest, Ethiopia: a cross sectional study. BMC Womens Health. 2020;20:1–9.
769. Gelagay AA, Koye DN, Yeshita HY. Demand for long acting contraceptive methods among married HIV positive women attending care at public health facilities at Bahir Dar City, Northwest Ethiopia. Reprod Health. 2015;12:1–9.
770. Juliastuti D, Dean J, Fitzgerald L. Sexual and reproductive health of women living with HIV in Muslim-majority countries: a systematic mixed studies review. BMC Int Health Hum Rights. 2020;20:1–12.
771. Esplen E. Women and girls living with HIV/AIDS: overview and annotated bibliography. Institute of Development Studies at the University of Sussex Brighton, UK; 2007.
772. Hagey JM, Akama E, Ayieko J, Bukusi EA, Cohen CR, Patel RC. Barriers and facilitators adolescent females living with HIV face in accessing contraceptive services: a qualitative assessment of providers’ perceptions in western Kenya. African J Reprod Gynaecol Endosc. 2015;18(1).
773. Tewabe T, Abdanur A, Jenbere D, Ayehu M, Talema G. Contraceptive Use among sexually active reproductive age HIV Positive Women Attending ART Clinic at Felege Hiwot Referral Hospital, Northwest Ethiopia: A cross-sectional study. 2020;
774. Adilo TM. Prevalence and determinants of contraceptive utilization among reproductive age women living with HIV/AIDS in Addis Ababa, Ethiopia; a cross sectional study design. EC Gynaecol. 2017;4(3):97–112.
775. Lewinsohn R, Crankshaw T, Tomlinson M, Gibbs A, Butler L, Smit J. “This baby came up and then he said,“I give up!”: The interplay between unintended pregnancy, sexual partnership dynamics and social support and the impact on women’s well-being in KwaZulu-Natal, South Africa. Midwifery. 2018;62:29–35.
776. Lunani LL, Abaasa A, Omosa-Manyonyi G. Prevalence and factors associated with contraceptive use among Kenyan women aged 15–49 years. AIDS Behav. 2018;22:125–30.
777. Grossman D, Onono M, Newmann SJ, Blat C, Bukusi EA, Shade SB, et al. Integration of family planning services into HIV care and treatment in Kenya: a cluster-randomized trial. Aids. 2013;27:S77–85.
778. Ahinkorah BO, Seidu A-A, Appiah F, Baatiema L, Sambah F, Budu E, et al. What has reproductive health decision-making capacity got to do with unintended pregnancy? Evidence from the 2014 Ghana Demographic and Health Survey. PLoS One. 2019;14(10):e0223389.
779. Wilcher R, Cates W. Reaching the underserved: family planning for women with HIV. Stud Fam Plann. 2010;41(2):125–8.
780. Alene KA, Atalell KA. Contraceptive use and method preference among HIV-positive women in Amhara region, Ethiopia. BMC Womens Health. 2018;18:1–9.
781. Berhane Y, Berhe H, Abera GB, Berhe H. Utilization of modern contraceptives among HIV positive reproductive age women in Tigray, Ethiopia: a cross sectional study. Int Sch Res Not. 2013;2013(1):319724.
782. Colombini M, Mayhew SH, Mutemwa R, Kivunaga J, Ndwiga C, Team I. Perceptions and experiences of integrated service delivery among women living with HIV attending reproductive health services in Kenya: a mixed methods study. AIDS Behav. 2016;20:2130–40.
783. Skerritt L, Kaida A, O’Brien N, Burchell AN, Bartlett G, Savoie É, et al. Patterns of changing pregnancy intentions among women living with HIV in Canada. BMC Womens Health. 2021;21:1–12.
784. Kakaire O, Tumwesigye NM, Byamugisha JK, Gemzell-Danielsson K. Acceptability of intrauterine contraception among women living with human immunodeficiency virus: a randomised clinical trial. Eur J Contracept Reprod Heal Care. 2016;21(3):220–6.
785. Shabiby MM, Karanja JG, Odawa F, Kosgei R, Kibore MW, Kiarie JN, et al. Factors influencing uptake of contraceptive implants in the immediate postpartum period among HIV infected and uninfected women at two Kenyan District Hospitals. BMC Womens Health. 2015;15:1–8.
786. Magadi MA, Agwanda AO. Determinants of transitions to first sexual intercourse, marriage and pregnancy among female adolescents: evidence from South Nyanza, Kenya. J Biosoc Sci. 2009;41(3):409–27.
787. Doherty K, Arena K, Wynn A, Offorjebe OA, Moshashane N, Sickboy O, et al. Unintended pregnancy in Gaborone, Botswana: A cross sectional study. Afr J Reprod Health. 2018;22(2):76–82.
788. Omollo C. Determinants of contraceptives preference and use among people living with hiv and aids in rural areas: a study of Nyamarambe division, Kisii county, Kenya. 2021.
789. Bachanas P, Kidder D, Medley A, Pals SL, Carpenter D, Howard A, et al. Delivering prevention interventions to people living with HIV in clinical care settings: results of a cluster randomized trial in Kenya, Namibia, and Tanzania. AIDS Behav. 2016;20:2110–8.
790. Kebede YB, Geremew TT, Mehretie Y, Abejie AN, Bewket L, Dellie E. Associated factors of modern contraceptive use among women infected with human immunodeficiency virus in Enemay District, Northwest Ethiopia: a facility-based cross-sectional study. BMC Public Health. 2019;19:1–11.
791. Tumusiigirwe K. Factors associated with unwanted pregnancies among girls aged 15 to 19 years in Kakoba Division in Mbarara District. 2017;
792. Kamangu AA, Myeya HE. Exploring Young Peoples’ Sexual Behaviours and the Underlying Factors in East Africa: A Review of Literature from Four Countries. J Anthropol Surv India. 2023;72(1):149–62.
793. Zewdu LB, Reta MM, Yigzaw N, Tamirat KS. Prevalence of suicidal ideation and associated factors among HIV positive perinatal women on follow-up at Gondar town health institutions, Northwest Ethiopia: a cross-sectional study. BMC Pregnancy Childbirth. 2021;21:1–9.
794. Heys J, Kipp W, Jhangri GS, Alibhai A, Rubaale T. Fertility desires and infection with the HIV: results from a survey in rural Uganda. Aids. 2009;23:S37–45.
795. De Bruyn M. Women, reproductive rights, and HIV/AIDS: Issues on which research and interventions are still needed. J Health Popul Nutr. 2006;24(4):413.
796. Arega T. HIV and Unintended Pregnancy Risk Perception and Contraceptive Use among Youth in Debre Birhan District, Ethiopia. Addis Ababa University; 2010.
797. Abubeker FA, Fanta MB, Dalton VK. Unmet Need for Contraception among HIV‐Positive Women Attending HIV Care and Treatment Service at Saint Paul’s Hospital Millennium Medical College, Addis Ababa, Ethiopia. Int J Reprod Med. 2019;2019(1):3276780.
798. Ma Q, Ono-Kihara M, Cong L, Xu G, Pan X, Zamani S, et al. Early initiation of sexual activity: a risk factor for sexually transmitted diseases, HIV infection, and unwanted pregnancy among university students in China. BMC Public Health. 2009;9:1–8.
799. Shikhansari S, Khalesi ZB, Rad EH. Factors associated with the reproductive health of women living with HIV in Iran. Eur J Obstet Gynecol Reprod Biol X. 2022;13:100136.
800. Nyanja TAN, Tulinius C. Relationships matter: contraceptive choices among HIV-positive women in Tanzania. African J AIDS Res. 2017;16(2):109–17.
801. Johnson LF, Mutemaringa T, Heekes A, Boulle A. Effect of HIV infection and antiretroviral treatment on pregnancy rates in the Western Cape province of South Africa. J Infect Dis. 2020;221(12):1953–62.
802. Nakku‐Joloba E, Pisarski EE, Wyatt MA, Muwonge TR, Asiimwe S, Celum CL, et al. Beyond HIV prevention: everyday life priorities and demand for PrEP among Ugandan HIV serodiscordant couples. African J Reprod Gynaecol Endosc. 2019;22(1).
803. Maharaj P. The dual risks of unwanted pregnancy and HIV/AIDS: the case of KwaZulu-Natal, South Africa. London School of Hygiene & Tropical Medicine; 2003.
804. Bradley H, Tsui A, Kidanu A, Gillespie D. HIV infection and contraceptive need among female Ethiopian voluntary HIV counseling and testing clients. AIDS Care. 2010;22(10):1295–304.
805. Mburu G, Ndimbii J, Ayon S, Mlewa O, Mbizvo M, Kihara C, et al. Contraceptive use among women who inject drugs: motivators, barriers, and unmet needs. Women’s Reprod Heal. 2018;5(2):99–116.
806. Adilo TM, Wordofa HM. Prevalence of fertility desire and its associated factors among 15-to 49-year-old people living with HIV/AIDS in Addis Ababa, Ethiopia: a cross-sectional study design. HIV/AIDS-Research Palliat Care. 2017;167–76.
807. Ashimi AO, Amole TG, Abubakar MY, Ugwa EA. Fertility desire and utilization of family planning methods among HIV‑positive women attending a tertiary hospital in a suburban setting in Northern Nigeria. Trop J Obstet Gynaecol. 2017;34(1):54–60.
808. Selke HM, Kimaiyo S, Sidle JE, Vedanthan R, Tierney WM, Shen C, et al. Task-shifting of antiretroviral delivery from health care workers to persons living with HIV/AIDS: clinical outcomes of a community-based program in Kenya. JAIDS J Acquir Immune Defic Syndr. 2010;55(4):483–90.
809. Druce N, Nolan A. Seizing the big missed opportunity: linking HIV and maternity care services in sub-Saharan Africa. Reprod Health Matters. 2007;15(30):190–201.
810. Schaan MM, Taylor M, Gungqisa N, Marlink R. Personal views about womanhood amongst women living with HIV in Botswana. Cult Health Sex. 2016;18(2):171–83.
811. Singh S, Bankole A, Woog V. Evaluating the need for sex education in developing countries: sexual behaviour, knowledge of preventing sexually transmitted infections/HIV and unplanned pregnancy. Sex Educ. 2005;5(4):307–31.
812. Myer L, Carter RJ, Katyal M, Toro P, El-Sadr WM, Abrams EJ. Impact of antiretroviral therapy on incidence of pregnancy among HIV-infected women in Sub-Saharan Africa: a cohort study. PLoS Med. 2010;7(2):e1000229.
813. Nakanwagi M, Bulage L, Kwesiga B, Ario AR, Birungi DA, Lukabwe I, et al. Low proportion of women who came knowing their HIV status at first antenatal care visit, Uganda, 2012–2016: a descriptive analysis of surveillance data. BMC Pregnancy Childbirth. 2020;20:1–8.
814. Kanyangarara M, Sakyi K, Laar A. Availability of integrated family planning services in HIV care and support sites in sub-Saharan Africa: a secondary analysis of national health facility surveys. Reprod Health. 2019;16:1–9.
815. Berer M. HIV/AIDS, sexual and reproductive health: intersections and implications for national programmes. Health Policy Plan. 2004;19(suppl_1):i62–70.
816. Bowring AL, Schwartz S, Lyons C, Rao A, Olawore O, Njindam IM, et al. Unmet need for family planning and experience of unintended pregnancy among female sex workers in urban Cameroon: results from a national cross-sectional study. Glob Heal Sci Pract. 2020;8(1):82–99.
817. Tibebu NS, Kassie BA, Anteneh TA, Rade BK. Depression, anxiety and stress among HIV-positive pregnant women in Ethiopia during the COVID-19 pandemic. Trans R Soc Trop Med Hyg. 2023;117(5):317–25.
818. Fotso JC, Izugbara C, Saliku T, Ochako R. Unintended pregnancy and subsequent use of modern contraceptive among slum and non-slum women in Nairobi, Kenya. BMC Pregnancy Childbirth. 2014;14:1–10.
819. Bakari HM, Alo O, Mbwana MS, Salim SM, Ludeman E, Lascko T, et al. Prevalence of unmet need for family planning and unintended pregnancies among women of reproductive age living with HIV in sub-Saharan Africa: a systematic review and meta-analysis. Afr Health Sci. 2024;24(2):41–53.
820. Mayhew SH, Colombini M, Kimani JK, Tomlin K, Warren CE, Initiative I, et al. Fertility intentions and contraceptive practices among clinic-users living with HIV in Kenya: a mixed methods study. BMC Public Health. 2017;17:1–15.
821. Atukunda EC, Mugyenyi GR, Atuhumuza EB, Kaida A, Boatin A, Agaba AG, et al. Factors associated with pregnancy intentions amongst postpartum women living with HIV in rural Southwestern Uganda. AIDS Behav. 2019;23:1552–60.
822. Wall KM, Haddad L, Vwalika B, Htee Khu N, Brill I, Kilembe W, et al. Unintended pregnancy among HIV positive couples receiving integrated HIV counseling, testing, and family planning services in Zambia. PLoS One. 2013;8(9):e75353.
823. Kimani J, Warren C, Abuya T, Mutemwa R, Initiative I, Mayhew S, et al. Family planning use and fertility desires among women living with HIV in Kenya. BMC Public Health. 2015;15:1–10.
824. Namukisa M, Kamacooko O, Lunkuse JF, Ruzagira E, Price MA, Mayanja Y. Incidence of unintended pregnancy and associated factors among adolescent girls and young women at risk of HIV infection in Kampala, Uganda. Front Reprod Heal. 2023;5:1089104.
825. Ikamari L, Izugbara C, Ochako R. Prevalence and determinants of unintended pregnancy among women in Nairobi, Kenya. BMC Pregnancy Childbirth. 2013;13:1–9.
826. Amongi PR. Factors Associated With Unintended Pregnancy Among Hiv Positive Women On Anti Retroviral Therapy In Gulu District. CIU; 2018.
827. Feyissa TR, Harris ML, Melka AS, Loxton D. Unintended pregnancy in women living with HIV in Sub-Saharan Africa: a systematic review and meta-analysis. AIDS Behav. 2019;23:1431–51.
828. Ingabire R, Parker R, Nyombayire J, Ko JE, Mukamuyango J, Bizimana J, et al. Female sex workers in Kigali, Rwanda: a key population at risk of HIV, sexually transmitted infections, and unplanned pregnancy. Int J STD AIDS. 2019;30(6):557–68.
829. Mumah JN, Mulupi S, Wado YD, Ushie BA, Nai D, Kabiru CW, et al. Adolescents’ narratives of coping with unintended pregnancy in Nairobi’s informal settlements. PLoS One. 2020;15(10):e0240797.
830. Mumah J, Kabiru CW, Mukiira C, Brinton J, Mutua M, Izugbara CO, et al. Unintended pregnancies in Kenya: a country profile. 2014;
831. Warren CE, Abuya T, Askew I. Family planning practices and pregnancy intentions among HIV-positive and HIV-negative postpartum women in Swaziland: a cross sectional survey. BMC Pregnancy Childbirth. 2013;13:1–10.
832. Solanke BL. Do community characteristics influence unintended pregnancies in Kenya? Malawi Med J. 2019;31(1):56–64.
833. Luchters S, Bosire W, Feng A, Richter ML, King’ola N, Ampt F, et al. “A baby was an added burden”: predictors and consequences of unintended pregnancies for female sex Workers in Mombasa, Kenya: a mixed-methods study. PLoS One. 2016;11(9):e0162871.
834. 7. Teklu T, Davey G. Which factors influence North Ethiopian adults’ use of dual protection from unintended pregnancy and HIV/AIDS? Ethiop J Heal Dev. 2008;22(3).
835. 8. Raifman J, Chetty T, Tanser F, Mutevedzi T, Matthews P, Herbst K, et al. Preventing unintended pregnancy and HIV transmission: effects of the HIV treatment cascade on contraceptive use and choice in rural KwaZulu-Natal. JAIDS J Acquir Immune Defic Syndr. 2014;67:S218–27.
836. 9. Magadi MA. HIV and Unintended Fertility in Sub-Saharan Africa: Multilevel Predictors of Mistimed and Unwanted Fertility Among HIV-Positive Women. Popul Res Policy Rev. 2021;40(5):987–1024.
837. Mwalye PJ. Impact of Unintended pregnancy on HIV viral load outcomes among postpartum women living with HIV in Cape Town, South Africa: clues from postpartum adherence clubs for antiretroviral therapy trial. 2022;
838. Harrington EK, Newmann SJ, Onono M, Schwartz KD, Bukusi EA, Cohen CR, et al. Fertility intentions and interest in integrated family planning services among women living with HIV in Nyanza Province, Kenya: a qualitative study. Infect Dis Obstet Gynecol. 2012;2012(1):809682.
839. Antelman G, Medley A, Mbatia R, Pals S, Arthur G, Haberlen S, et al. Pregnancy desire and dual method contraceptive use among people living with HIV attending clinical care in Kenya, Namibia and Tanzania. J Fam Plan Reprod Heal Care. 2015;41(1):e1–e1.
840. Heffron R, Thomson K, Celum C, Haberer J, Ngure K, Mugo N, et al. Fertility intentions, pregnancy, and use of PrEP and ART for safer conception among East African HIV serodiscordant couples. AIDS Behav. 2018;22:1758–65.
841. Adeniyi OV, Ajayi AI, Somefun OD, Lambert JS. Provision of immediate postpartum contraception to women living with HIV in the Eastern Cape, South Africa; a cross-sectional analysis. Reprod Health. 2020;17:1–12.
842. Patel RC, Amorim G, Jakait B, Shepherd BE, Mocello AR, Musick B, et al. Pregnancies among women living with HIV using contraceptives and antiretroviral therapy in western Kenya: a retrospective, cohort study. BMC Med. 2021;19:1–11.
843. Bain LE, Zweekhorst MBM, de Cock Buning T. Prevalence and determinants of unintended pregnancy in sub–saharan Africa: a systematic review. Afr J Reprod Health. 2020;24(2):187–205.
844. Fotso JC, Izugbara C, Saliku T, Ochako R. Unintended pregnancy and subsequent use of modern contraceptive among slum and non-slum women in Nairobi, Kenya. BMC Pregnancy Childbirth. 2014;14:1–10.
845. Bakari HM, Alo O, Mbwana MS, Salim SM, Ludeman E, Lascko T, et al. Prevalence of unmet need for family planning and unintended pregnancies among women of reproductive age living with HIV in sub-Saharan Africa: a systematic review and meta-analysis. Afr Health Sci. 2024;24(2):41–53.
846. Njuguna E, Ilovi S, Muiruri P, Mutai K, Kinuthia J, Njoroge P. Factors influencing the utilization of family planning services among HIV infected women in a Kenyan health facility. Int J Reprod Contracept Obs Gynecol. 2017;6(5):1746–52.
847. Lawani LO, Onyebuchi AK, Iyoke CA. Dual method use for protection of pregnancy and disease prevention among HIV-infected women in South East Nigeria. BMC Womens Health. 2014;14:1–6.
848. Habte D, Namasasu J. Family planning use among women living with HIV: knowing HIV positive status helps-results from a national survey. Reprod Health. 2015;12:1–11.
849. Patel RC, Onono M, Gandhi M, Blat C, Hagey J, Shade SB, et al. Pregnancy rates in HIV-positive women using contraceptives and efavirenz-based or nevirapine-based antiretroviral therapy in Kenya: a retrospective cohort study. lancet HIV. 2015;2(11):e474–82.
850. Lewinsohn R, Crankshaw T, Tomlinson M, Gibbs A, Butler L, Smit J. “This baby came up and then he said,“I give up!”: The interplay between unintended pregnancy, sexual partnership dynamics and social support and the impact on women’s well-being in KwaZulu-Natal, South Africa. Midwifery. 2018;62:29–35.
851. Ahinkorah BO, Seidu A-A, Appiah F, Oduro JK, Sambah F, Baatiema L, et al. Effect of sexual violence on planned, mistimed and unwanted pregnancies among women of reproductive age in sub-Saharan Africa: A multi-country analysis of Demographic and Health Surveys. SSM-population Heal. 2020;11:100601.
852. Ochieng Arunda M, Agardh A, Larsson M, Asamoah BO. Survival patterns of neonates born to adolescent mothers and the effect of pregnancy intentions and marital status on newborn survival in Kenya, Uganda, and Tanzania, 2014–2016. Glob Health Action. 2022;15(1):2101731.
853. Ma Q, Ono-Kihara M, Cong L, Xu G, Pan X, Zamani S, et al. Unintended pregnancy and its risk factors among university students in eastern China. Contraception. 2008;77(2):108–13.
854. Mokwena K, Bogale YR. Fertility intention and use of contraception among women living with the human immunodeficiency virus in Oromia Region, Ethiopia. South African Fam Pract. 2017;59(1):46–51.
855. Bakibinga P, Matanda DJ, Ayiko R, Rujumba J, Muiruri C, Amendah D, et al. Pregnancy history and current use of contraception among women of reproductive age in Burundi, Kenya, Rwanda, Tanzania and Uganda: analysis of demographic and health survey data. BMJ Open. 2016;6(3):e009991.
856. Okigbo CC, Speizer IS. Determinants of sexual activity and pregnancy among unmarried young women in urban Kenya: a cross-sectional study. PLoS One. 2015;10(6):e0129286.
857. Ampt FH, Willenberg L, Agius PA, Chersich M, Luchters S, Lim MSC. Incidence of unintended pregnancy among female sex workers in low-income and middle-income countries: a systematic review and meta-analysis. BMJ Open. 2018;8(9):e021779.
858. Duff P, Muzaaya G, Muldoon K, Dobrer S, Akello M, Birungi J, et al. High rates of unintended pregnancies among young women sex Workers in Conflict-affected Northern Uganda: the social contexts of brothels/lodges and substance use. Afr J Reprod Health. 2017;21(2):64–72.
859. Ngugi EW, Kim AA, Nyoka R, Mukui I, Ng’eno B, Rutherford GW, et al. Contraceptive practices and fertility desires among HIV-infected and uninfected women in Kenya: results from a nationally representative study. JAIDS J Acquir Immune Defic Syndr. 2014;66:S75–81.
860. Mubangizi L. Examining the association between future pregnancy intentions, contraceptive use and repeat pregnancies among women living with HIV in Cape Town, South Africa. 2020;
861. Mbongueh MC, Nicholas T, Ndum AC, Gisèle EL, Nguedia A, Clement J. Unintended pregnancy and sexually transmissible infections amongst adolescents and young adults in Douala IV municipality, Cameroon: Prevalence, knowledge, and associated factors. 2023;
862. Khu NH, Vwalika B, Karita E, Kilembe W, Bayingana RA, Sitrin D, et al. Fertility goal-based counseling increases contraceptive implant and IUD use in HIV-discordant couples in Rwanda and Zambia. Contraception. 2013;88(1):74–82.
863. Haile D, Lagebo B. Magnitude of dual contraceptive method utilization and the associated factors among women on antiretroviral treatment in Wolaita zone, Southern Ethiopia. Heliyon. 2022;8(6).
864. Credé S, Hoke T, Constant D, Green MS, Moodley J, Harries J. Factors impacting knowledge and use of long acting and permanent contraceptive methods by postpartum HIV positive and negative women in Cape Town, South Africa: a cross-sectional study. BMC Public Health. 2012;12:1–9.
865. Wasie B, Belyhun Y, Moges B, Amare B. Effect of emergency oral contraceptive use on condom utilization and sexual risk taking behaviours among university students, Northwest Ethiopia: a cross-sectional study. BMC Res Notes. 2012;5:1–9.
866. Feyissa TR, Melka AS. Demand for modern family planning among married women living with HIV in western Ethiopia. PLoS One. 2014;9(11):e113008.
867. Young IC, Benhabbour SR. Multipurpose prevention technologies: oral, parenteral, and vaginal dosage forms for prevention of HIV/STIs and unplanned pregnancy. Polymers (Basel). 2021;13(15):2450.
868. Tirado V, Orsini N, Strömdahl S, Hanson C, Ekström AM. Knowledge gaps related to HIV and condom use for preventing pregnancy: a cross-sectional study among migrants in Sweden. BMC Public Health. 2024;24(1):2334.
869. Amuyunzu-Nyamongo M, Tendo-Wambua L, Babishangire B, Nyagero J, Yitbarek N, Matasha M, et al. Barriers to behaviour change as a response to STD including HIV/AIDS: the East African experience. In Citeseer; 1999.
870. Chanda P, JO EK, Ochieng LA. FACTORS AFFECTING UPTAKE OF CONTRACEPTIVES AMONG WOMEN AGED 15-25 IN THE CONTEXT OF EARLY PREGNANCY AND HIV/AIDS PREVENTION IN UGANDA.
871. Dibaba Y, Fantahun M, Hindin MJ. The association of unwanted pregnancy and social support with depressive symptoms in pregnancy: evidence from rural Southwestern Ethiopia. BMC Pregnancy Childbirth. 2013;13:1–8.
872. Abebe EC, Ayele TM, Dejenie TA, Muche ZT. Assessment of Modern Contraceptive Utilization and Associated Factors Among Women Living With HIV/AIDS In DTRH In Debre Tabor Town, South Gondar Zone, Ethiopia. 2021;
873. Othieno C, Babigumira JB, Richardson B. Are women with complications of an incomplete abortion more likely to be HIV infected than women without complications? BMC Womens Health. 2015;15:1–9.
874. Kwame KA, Bain LE, Manu E, Tarkang EE. Use and awareness of emergency contraceptives among women of reproductive age in sub-Saharan Africa: a scoping review. Contracept Reprod Med. 2022;7(1):1.
875. Reynolds HW, Janowitz B, Homan R, Johnson L. The value of contraception to prevent perinatal HIV transmission. Sex Transm Dis. 2006;33(6):350–6.
876. Kisaakye P. Contraceptive histories: A comparative analysis of switching behaviour in five East African countries. 2019;
877. Mamboleo N. Unwanted pregnancy and induced abortion among female youths: a case study of Temeke district. Muhimbili University of Health and Allied Sciences; 2012.
878. Bastola K. Unintended pregnancy among currently pregnant maried women in Nepal. 2013.
879. Shehu AU, Joshua IA, Umar Z. Knowledge of contraception and contraceptive choices among human immunodeficiency virus-positive women attending antiretroviral clinics in Zaria, Nigeria. Sub-Saharan African J Med. 2016;3(2):84–90.
880. Uwamariya J, Nyandwi J, Mukanyangezi M, Kadima J. Sexual activity and emergency contraception among female students in the University of Rwanda. Int J Trop Dis Heal. 2015;8(4):170–7.
881. Maloiy L, Wawire V. Status and Dynamics of Gender Mainstreaming in East Africa Community COVID-19 Social and Economic Response Policies, Strategies and Interventions. 2021;
882. Pretorius L, Gibbs A, Crankshaw T, Willan S. Interventions targeting sexual and reproductive health and rights outcomes of young people living with HIV: a comprehensive review of current interventions from sub-Saharan Africa. Glob Health Action. 2015;8(1):28454.
883. Haddad L, Wall KM, Vwalika B, Khu NH, Brill I, Kilembe W, et al. Contraceptive discontinuation and switching among couples receiving integrated HIV and family planning services in Lusaka, Zambia. Aids. 2013;27:S93–103.
884. Warren CE, Mayhew SH, Hopkins J. The current status of research on the integration of sexual and reproductive health and HIV services. Stud Fam Plann. 2017;48(2):91–105.
885. Warren CE, Mayhew SH, Vassall A, Kimani JK, Church K, Obure CD, et al. Study protocol for the Integra Initiative to assess the benefits and costs of integrating sexual and reproductive health and HIV services in Kenya and Swaziland. BMC Public Health. 2012;12:1–16.
886. Wilcher R, Cates W. Reproductive choices for women with HIV. Bull World Health Organ. 2009;87(11):833–9.
887. Abdool Karim S, Baxter C, Frohlich J, Abdool Karim Q. The need for multipurpose prevention technologies in sub‐Saharan Africa. BJOG An Int J Obstet Gynaecol. 2014;121:27–34.
888. Matthews LT, Crankshaw T, Giddy J, Kaida A, Smit JA, Ware NC, et al. Reproductive decision-making and periconception practices among HIV-positive men and women attending HIV services in Durban, South Africa. AIDS Behav. 2013;17:461–70.
889. Hancock NL, Chibwesha CJ, Bosomprah S, Newman J, Mubiana-Mbewe M, Sitali ES, et al. Contraceptive use among HIV-infected women and men receiving antiretroviral therapy in Lusaka, Zambia: a cross-sectional survey. BMC Public Health. 2016;16:1–8.
890. Mbirimtengerenji ND. Is HIV/AIDS epidemic outcome of poverty in sub-saharan Africa? Croat Med J. 2007;48(5):605.
891. Zaidi SS, Ocholla AM, Otieno RA, Sandfort TGM. Women who have sex with women in Kenya and their sexual and reproductive health. LGBT Heal. 2016;3(2):139–45.
892. Marlow HM, Maman S, Groves AK, Moodley D. Fertility intent and contraceptive decision-making among HIV positive and negative antenatal clinic attendees in Durban, South Africa. Health Care Women Int. 2012;33(4):342–58.
893. Morrison JS, Fleischman J. Integrating reproductive health and HIV/AIDS programs. Strateg Oppor PEPFAR. 2006;6–7.
894. Darteh EKM, Abraham SA, Seidu A-A, Chattu VK, Yaya S. Knowledge and determinants of women’s knowledge on vertical transmission of HIV and AIDS in South Africa. AIDS Res Ther. 2021;18:1–9.
895. Amo-Adjei J, Tuoyire DA. Timing of sexual debut among unmarried youths aged 15–24 years in sub-Saharan Africa. J Biosoc Sci. 2018;50(2):161–77.
896. Papworth E, Schwartz S, Ky-Zerbo O, Leistman B, Ouedraogo G, Samadoulougou C, et al. Mothers who sell sex: a potential paradigm for integrated HIV, sexual, and reproductive health interventions among women at high risk of HIV in Burkina Faso. JAIDS J Acquir Immune Defic Syndr. 2015;68:S154–61.
897. Fotso JC, Izugbara C, Saliku T, Ochako R. Unintended pregnancy and subsequent use of modern contraceptive among slum and non-slum women in Nairobi, Kenya. BMC Pregnancy Childbirth. 2014;14:1–10.
898. Bakari HM, Alo O, Mbwana MS, Salim SM, Ludeman E, Lascko T, et al. Prevalence of unmet need for family planning and unintended pregnancies among women of reproductive age living with HIV in sub-Saharan Africa: a systematic review and meta-analysis. Afr Health Sci. 2024;24(2):41–53.
899. Mayhew SH, Colombini M, Kimani JK, Tomlin K, Warren CE, Initiative I, et al. Fertility intentions and contraceptive practices among clinic-users living with HIV in Kenya: a mixed methods study. BMC Public Health. 2017;17:1–15.
900. Atukunda EC, Mugyenyi GR, Atuhumuza EB, Kaida A, Boatin A, Agaba AG, et al. Factors associated with pregnancy intentions amongst postpartum women living with HIV in rural Southwestern Uganda. AIDS Behav. 2019;23:1552–60.
901. Wall KM, Haddad L, Vwalika B, Htee Khu N, Brill I, Kilembe W, et al. Unintended pregnancy among HIV positive couples receiving integrated HIV counseling, testing, and family planning services in Zambia. PLoS One. 2013;8(9):e75353.
902. Kimani J, Warren C, Abuya T, Mutemwa R, Initiative I, Mayhew S, et al. Family planning use and fertility desires among women living with HIV in Kenya. BMC Public Health. 2015;15:1–10.
903. Namukisa M, Kamacooko O, Lunkuse JF, Ruzagira E, Price MA, Mayanja Y. Incidence of unintended pregnancy and associated factors among adolescent girls and young women at risk of HIV infection in Kampala, Uganda. Front Reprod Heal. 2023;5:1089104.
904. Ikamari L, Izugbara C, Ochako R. Prevalence and determinants of unintended pregnancy among women in Nairobi, Kenya. BMC Pregnancy Childbirth. 2013;13:1–9.
905. Amongi PR. Factors Associated With Unintended Pregnancy Among Hiv Positive Women On Anti Retroviral Therapy In Gulu District. CIU; 2018.
906. Feyissa TR, Harris ML, Melka AS, Loxton D. Unintended pregnancy in women living with HIV in Sub-Saharan Africa: a systematic review and meta-analysis. AIDS Behav. 2019;23:1431–51.
907. Ingabire R, Parker R, Nyombayire J, Ko JE, Mukamuyango J, Bizimana J, et al. Female sex workers in Kigali, Rwanda: a key population at risk of HIV, sexually transmitted infections, and unplanned pregnancy. Int J STD AIDS. 2019;30(6):557–68.
908. Mumah JN, Mulupi S, Wado YD, Ushie BA, Nai D, Kabiru CW, et al. Adolescents’ narratives of coping with unintended pregnancy in Nairobi’s informal settlements. PLoS One. 2020;15(10):e0240797.
909. Mumah J, Kabiru CW, Mukiira C, Brinton J, Mutua M, Izugbara CO, et al. Unintended pregnancies in Kenya: a country profile. 2014;
910. Warren CE, Abuya T, Askew I. Family planning practices and pregnancy intentions among HIV-positive and HIV-negative postpartum women in Swaziland: a cross sectional survey. BMC Pregnancy Childbirth. 2013;13:1–10.
911. Solanke BL. Do community characteristics influence unintended pregnancies in Kenya? Malawi Med J. 2019;31(1):56–64.
912. Luchters S, Bosire W, Feng A, Richter ML, King’ola N, Ampt F, et al. “A baby was an added burden”: predictors and consequences of unintended pregnancies for female sex Workers in Mombasa, Kenya: a mixed-methods study. PLoS One. 2016;11(9):e0162871.
913. 7. Teklu T, Davey G. Which factors influence North Ethiopian adults’ use of dual protection from unintended pregnancy and HIV/AIDS? Ethiop J Heal Dev. 2008;22(3).
914. 8. Raifman J, Chetty T, Tanser F, Mutevedzi T, Matthews P, Herbst K, et al. Preventing unintended pregnancy and HIV transmission: effects of the HIV treatment cascade on contraceptive use and choice in rural KwaZulu-Natal. JAIDS J Acquir Immune Defic Syndr. 2014;67:S218–27.
915. 9. Magadi MA. HIV and Unintended Fertility in Sub-Saharan Africa: Multilevel Predictors of Mistimed and Unwanted Fertility Among HIV-Positive Women. Popul Res Policy Rev. 2021;40(5):987–1024.
916. Mwalye PJ. Impact of Unintended pregnancy on HIV viral load outcomes among postpartum women living with HIV in Cape Town, South Africa: clues from postpartum adherence clubs for antiretroviral therapy trial. 2022;
917. Harrington EK, Newmann SJ, Onono M, Schwartz KD, Bukusi EA, Cohen CR, et al. Fertility intentions and interest in integrated family planning services among women living with HIV in Nyanza Province, Kenya: a qualitative study. Infect Dis Obstet Gynecol. 2012;2012(1):809682.
918. Antelman G, Medley A, Mbatia R, Pals S, Arthur G, Haberlen S, et al. Pregnancy desire and dual method contraceptive use among people living with HIV attending clinical care in Kenya, Namibia and Tanzania. J Fam Plan Reprod Heal Care. 2015;41(1):e1–e1.
919. Heffron R, Thomson K, Celum C, Haberer J, Ngure K, Mugo N, et al. Fertility intentions, pregnancy, and use of PrEP and ART for safer conception among East African HIV serodiscordant couples. AIDS Behav. 2018;22:1758–65.
920. Adeniyi OV, Ajayi AI, Somefun OD, Lambert JS. Provision of immediate postpartum contraception to women living with HIV in the Eastern Cape, South Africa; a cross-sectional analysis. Reprod Health. 2020;17:1–12.
921. Patel RC, Amorim G, Jakait B, Shepherd BE, Mocello AR, Musick B, et al. Pregnancies among women living with HIV using contraceptives and antiretroviral therapy in western Kenya: a retrospective, cohort study. BMC Med. 2021;19:1–11.
922. Bain LE, Zweekhorst MBM, de Cock Buning T. Prevalence and determinants of unintended pregnancy in sub–saharan Africa: a systematic review. Afr J Reprod Health. 2020;24(2):187–205.
923. Fotso JC, Izugbara C, Saliku T, Ochako R. Unintended pregnancy and subsequent use of modern contraceptive among slum and non-slum women in Nairobi, Kenya. BMC Pregnancy Childbirth. 2014;14:1–10.
924. Bakari HM, Alo O, Mbwana MS, Salim SM, Ludeman E, Lascko T, et al. Prevalence of unmet need for family planning and unintended pregnancies among women of reproductive age living with HIV in sub-Saharan Africa: a systematic review and meta-analysis. Afr Health Sci. 2024;24(2):41–53.
925. Njuguna E, Ilovi S, Muiruri P, Mutai K, Kinuthia J, Njoroge P. Factors influencing the utilization of family planning services among HIV infected women in a Kenyan health facility. Int J Reprod Contracept Obs Gynecol. 2017;6(5):1746–52.
926. Lawani LO, Onyebuchi AK, Iyoke CA. Dual method use for protection of pregnancy and disease prevention among HIV-infected women in South East Nigeria. BMC Womens Health. 2014;14:1–6.
927. Habte D, Namasasu J. Family planning use among women living with HIV: knowing HIV positive status helps-results from a national survey. Reprod Health. 2015;12:1–11.
928. Patel RC, Onono M, Gandhi M, Blat C, Hagey J, Shade SB, et al. Pregnancy rates in HIV-positive women using contraceptives and efavirenz-based or nevirapine-based antiretroviral therapy in Kenya: a retrospective cohort study. lancet HIV. 2015;2(11):e474–82.
929. Lewinsohn R, Crankshaw T, Tomlinson M, Gibbs A, Butler L, Smit J. “This baby came up and then he said,“I give up!”: The interplay between unintended pregnancy, sexual partnership dynamics and social support and the impact on women’s well-being in KwaZulu-Natal, South Africa. Midwifery. 2018;62:29–35.
930. Ahinkorah BO, Seidu A-A, Appiah F, Oduro JK, Sambah F, Baatiema L, et al. Effect of sexual violence on planned, mistimed and unwanted pregnancies among women of reproductive age in sub-Saharan Africa: A multi-country analysis of Demographic and Health Surveys. SSM-population Heal. 2020;11:100601.
931. Ochieng Arunda M, Agardh A, Larsson M, Asamoah BO. Survival patterns of neonates born to adolescent mothers and the effect of pregnancy intentions and marital status on newborn survival in Kenya, Uganda, and Tanzania, 2014–2016. Glob Health Action. 2022;15(1):2101731.
932. Ma Q, Ono-Kihara M, Cong L, Xu G, Pan X, Zamani S, et al. Unintended pregnancy and its risk factors among university students in eastern China. Contraception. 2008;77(2):108–13.
933. Mokwena K, Bogale YR. Fertility intention and use of contraception among women living with the human immunodeficiency virus in Oromia Region, Ethiopia. South African Fam Pract. 2017;59(1):46–51.
934. Bakibinga P, Matanda DJ, Ayiko R, Rujumba J, Muiruri C, Amendah D, et al. Pregnancy history and current use of contraception among women of reproductive age in Burundi, Kenya, Rwanda, Tanzania and Uganda: analysis of demographic and health survey data. BMJ Open. 2016;6(3):e009991.
935. Okigbo CC, Speizer IS. Determinants of sexual activity and pregnancy among unmarried young women in urban Kenya: a cross-sectional study. PLoS One. 2015;10(6):e0129286.
936. Ampt FH, Willenberg L, Agius PA, Chersich M, Luchters S, Lim MSC. Incidence of unintended pregnancy among female sex workers in low-income and middle-income countries: a systematic review and meta-analysis. BMJ Open. 2018;8(9):e021779.
937. Duff P, Muzaaya G, Muldoon K, Dobrer S, Akello M, Birungi J, et al. High rates of unintended pregnancies among young women sex Workers in Conflict-affected Northern Uganda: the social contexts of brothels/lodges and substance use. Afr J Reprod Health. 2017;21(2):64–72.
938. Ngugi EW, Kim AA, Nyoka R, Mukui I, Ng’eno B, Rutherford GW, et al. Contraceptive practices and fertility desires among HIV-infected and uninfected women in Kenya: results from a nationally representative study. JAIDS J Acquir Immune Defic Syndr. 2014;66:S75–81.
939. Mubangizi L. Examining the association between future pregnancy intentions, contraceptive use and repeat pregnancies among women living with HIV in Cape Town, South Africa. 2020;
940. Bankole A, Singh S, Hussain R, Oestreicher G. Condom use for preventing STI/HIV and unintended pregnancy among young men in Sub-Saharan Africa. Am J Mens Health. 2009;3(1):60–78.
941. Dhakal S, Song JS, Shin DE, Lee TH, So AY, Nam EW. Unintended pregnancy and its correlates among currently pregnant women in the Kwango District, Democratic Republic of the Congo. Reprod Health. 2016;13:1–7.
942. Izugbara C, Egesa C. The management of unwanted pregnancy among women in Nairobi, Kenya. Int J Sex Heal. 2014;26(2):100–12.
943. Wall KM, Kilembe W, Vwalika B, Haddad LB, Khu NH, Brill I, et al. Optimizing prevention of HIV and unplanned pregnancy in discordant African couples. J women’s Heal. 2017;26(8):900–10.
944. Aragaw FM, Amare T, Teklu RE, Tegegne BA, Alem AZ. Magnitude of unintended pregnancy and its determinants among childbearing age women in low and middle-income countries: evidence from 61 low and middle income countries. Front Reprod Heal. 2023;5:1113926.
945. Chukwunyere AP, Stella KA. Unintended pregnancy among undergraduate students at a select university, Eastern Cape, South Africa: effects, influences, outcomes and solutions. Gend Behav. 2019;17(4):14272–86.
946. De Bruyn M. Living with HIV: challenges in reproductive health care in South Africa. Afr J Reprod Health. 2004;92–8.
947. Awiti Ujiji O, Ekström AM, Ilako F, Indalo D, Wamalwa D, Rubenson B. Reasoning and deciding PMTCT-adherence during pregnancy among women living with HIV in Kenya. Cult Health Sex. 2011;13(7):829–40.
948. Hailegebreal S, Gilano G, Seboka BT, Sidelil H, Awol SM, Haile Y, et al. Prevalence and associated factors of early sexual initiation among female youth in East Africa: further analysis of recent demographic and health survey. BMC Womens Health. 2022;22(1):304.
949. Arunda MO, Agardh A, Larsson M, Asamoah BO. Survival patterns of neonates born to adolescent mothers and the effect of pregnancy intentions and marital status on newborn survival in Kenya, Uganda, and Tanzania, 2014–2016. Glob Health Action. 2022;15(1).
950. Guta NM, Ruksi ST, Senbata GM, Seid K. Predictors of perceived poor social support status of pregnant women attending antiretroviral therapy clinics in south west Ethiopia, 2021. Heliyon. 2023;9(7).
951. Ayenew A. Women living with HIV and dual contraceptive use in Ethiopia: systematic review and meta-analysis. Contracept Reprod Med. 2022;7(1):11.
952. Mbuthia CW. Fertility Desires and Contraceptive Practices Among Hiv Positive Adults at Naivasha District Hospital. University of Nairobi; 2010.
953. Habte D, Teklu S, Melese T, Magafu MGMD. Correlates of unintended pregnancy in Ethiopia: results from a national survey. PLoS One. 2013;8(12):e82987.
954. Rodriguez MI, Reeves MF, Caughey AB. Evaluating the competing risks of HIV acquisition and maternal mortality in Africa: a decision analysis. BJOG An Int J Obstet Gynaecol. 2012;119(9):1067–73.
955. Wapmuk AE, Gbajabiamila TA, Ohihoin AG, Ezechi OC. Family Planning In The Context Of HIV Infection. Niger J Clin Biomed Res Wapmuk al. 2017;7(9):6–22.
956. Bernard C, Jakait B, Fadel WF, Mocello AR, Onono MA, Bukusi EA, et al. Preferences for multipurpose technology and non-oral methods of antiretroviral therapy among women living with HIV in western Kenya: a survey study. Front Glob Women’s Heal. 2022;3:869623.
957. Tuthill EL, Maltby AE, Odhiambo BC, Akama E, Pellowski JA, Cohen CR, et al. “i found out I was pregnant, and I started feeling stressed”: A longitudinal qualitative perspective of mental health experiences among perinatal women living with hiv. AIDS Behav. 2021;25:4154–68.
958. Sibanda MY. Factors influencing women living with HIV/AIDS’intention to fall pregnant among those attending the OI/ART clinic in Murambinda, Buhera District, Manicaland Province, Zimbabwe, 2010. 2010;
959. Kebede HG, Nahusenay H, Birhane Y, Tesfaye DJ. Assessment of contraceptive use and associated factors among HIV positive women in Bahir-Dar Town, Northwest Ethiopia. Open Access Libr J. 2015;2(10):1–19.
960. Astawesegn FH, Stulz V, Conroy E, Mannan H. Trends and effects of antiretroviral therapy coverage during pregnancy on mother-to-child transmission of HIV in Sub-Saharan Africa. Evidence from panel data analysis. BMC Infect Dis. 2022;22(1):134.
961. O’Reilly KR, Kennedy CE, Fonner VA, Sweat MD. Family planning counseling for women living with HIV: a systematic review of the evidence of effectiveness on contraceptive uptake and pregnancy incidence, 1990 to 2011. BMC Public Health. 2013;13:1–10.
962. Bauni EK, Jarabi BO. Family planning and sexual behavior in the era of HIV/AIDS: the case of Nakuru District, Kenya. Wiley Online Library; 2000.
963. Pokharel R, Bhattarai G, Shrestha N, Onta S. Knowledge and utilization of family planning methods among people living with HIV in Kathmandu, Nepal. BMC Health Serv Res. 2018;18:1–12.
964. Kisaakye VK. The Effectiveness of Sexual and Reproductive Health Counselling Services for HIV Positive Women in Comprehensive Care Centres in Langata, Kenya. COHES-JKUAT; 2018.
965. De Paoli MM, Manongi R, Klepp K-I. Factors influencing acceptability of voluntary counselling and HIV-testing among pregnant women in Northern Tanzania. AIDS Care. 2004;16(4):411–25.
966. Long JE, Waruguru G, Yuhas K, Wilson KS, Masese LN, Wanje G, et al. Prevalence and predictors of unmet contraceptive need in HIV-positive female sex worker in Mombasa, Kenya. PLoS One. 2019;14(6):e0218291.
967. Melaku YA, Zeleke EG. Contraceptive utilization and associated factors among HIV positive women on chronic follow up care in Tigray Region, Northern Ethiopia: a cross sectional study. PLoS One. 2014;9(4):e94682.
968. Bergsjø P, Vangen S, Lie RT, Lyatuu R, LIE‐NIELSEN E, Oneko O. Recording of maternal deaths in an East African university hospital. Acta Obstet Gynecol Scand. 2010;89(6):789–93.
969. Cohen S. Hiding in plain sight: the role of contraception in preventing HIV. Guttmacher Policy Rev. 2008;11(1):2–5.
970. Richter DL, Sowell RL, Pluto DM. Factors affecting reproductive decisions of African American women living with HIV. Women Health. 2002;36(1):81–96.
971. Vifeme M, Gwendoline N, Ernest B, Mboh E, Nshom E, Marie TP. Pregnancy and Associated Factors Among Adolescents and Young Adults Living with HIV in the Northwest Region of Cameroon. J Womens Heal Dev. 2022;5(3):221–33.
972. O’Shea MS, Rosenberg NE, Hosseinipour MC, Stuart GS, Miller WC, Kaliti SM, et al. Effect of HIV status on fertility desire and knowledge of long-acting reversible contraception of postpartum Malawian women. AIDS Care. 2015;27(4):489–98.
973. Nakaie N, Tuon S, Nozaki I, Yamaguchi F, Sasaki Y, Kakimoto K. Family planning practice and predictors of risk of inconsistent condom use among HIV-positive women on anti-retroviral therapy in Cambodia. BMC Public Health. 2014;14:1–9.
974. Hancock NL, Chibwesha CJ, Bosomprah S, Newman J, Mubiana-Mbewe M, Sitali ES, et al. Contraceptive use among HIV-infected women and men receiving antiretroviral therapy in Lusaka, Zambia: a cross-sectional survey. BMC Public Health. 2016;16:1–8.
975. Mbirimtengerenji ND. Is HIV/AIDS epidemic outcome of poverty in sub-saharan Africa? Croat Med J. 2007;48(5):605.
976. Zaidi SS, Ocholla AM, Otieno RA, Sandfort TGM. Women who have sex with women in Kenya and their sexual and reproductive health. LGBT Heal. 2016;3(2):139–45.
977. Marlow HM, Maman S, Groves AK, Moodley D. Fertility intent and contraceptive decision-making among HIV positive and negative antenatal clinic attendees in Durban, South Africa. Health Care Women Int. 2012;33(4):342–58.
978. Morrison JS, Fleischman J. Integrating reproductive health and HIV/AIDS programs. Strateg Oppor PEPFAR. 2006;6–7.
979. Darteh EKM, Abraham SA, Seidu A-A, Chattu VK, Yaya S. Knowledge and determinants of women’s knowledge on vertical transmission of HIV and AIDS in South Africa. AIDS Res Ther. 2021;18:1–9.
980. Amo-Adjei J, Tuoyire DA. Timing of sexual debut among unmarried youths aged 15–24 years in sub-Saharan Africa. J Biosoc Sci. 2018;50(2):161–77.
981. Papworth E, Schwartz S, Ky-Zerbo O, Leistman B, Ouedraogo G, Samadoulougou C, et al. Mothers who sell sex: a potential paradigm for integrated HIV, sexual, and reproductive health interventions among women at high risk of HIV in Burkina Faso. JAIDS J Acquir Immune Defic Syndr. 2015;68:S154–61.
982. MacCarthy S, Rasanathan JJK, Ferguson L, Gruskin S. The pregnancy decisions of HIV-positive women: the state of knowledge and way forward. Reprod Health Matters. 2012;20(sup39):119–40.
983. Polisi A, Gebrehanna E, Tesfaye G, Asefa F. Modern contraceptive utilization among female ART attendees in health facilities of Gimbie town, West Ethiopia. Reprod Health. 2014;11:1–6.
984. Turan B, Stringer KL, Onono M, Bukusi EA, Weiser SD, Cohen CR, et al. Linkage to HIV care, postpartum depression, and HIV-related stigma in newly diagnosed pregnant women living with HIV in Kenya: a longitudinal observational study. BMC Pregnancy Childbirth. 2014;14:1–10.
985. Birungi H, Obare F, van der Kwaak A, Namwebya JH. Maternal health care utilization among HIV-positive female adolescents in Kenya. Int Perspect Sex Reprod Health. 2011;143–9.
986. Patel RC, Morroni C, Scarsi KK, Sripipatana T, Kiarie J, Cohen CR. Concomitant contraceptive implant and efavirenz use in women living with HIV: perspectives on current evidence and policy implications for family planning and HIV treatment guidelines. African J Reprod Gynaecol Endosc. 2017;20(1).
987. Mba CJ. Sexual Behaviour and The risks of HIV/AIDS and other STDs among young people in sub-Saharan Africa: a REVIEW. Inst African Stud Res Rev. 2003;19(1):15–25.
988. Todd CS, Stibich MA, Laher F, Malta MS, Bastos FI, Imbuki K, et al. Influence of culture on contraceptive utilization among HIV-positive women in Brazil, Kenya, and South Africa. AIDS Behav. 2011;15:454–68.
989. Suryavanshi N, Erande A, Pisal H, Shankar A V, Bhosale RA, Bollinger RC, et al. Repeated pregnancy among women with known HIV status in Pune, India. AIDS Care. 2008;20(9):1111–8.
990. Levandowski BA, Kalilani‐Phiri L, Kachale F, Awah P, Kangaude G, Mhango C. Investigating social consequences of unwanted pregnancy and unsafe abortion in Malawi: the role of stigma. Int J Gynecol Obstet. 2012;118:S167–71.
991. MONEM AA. UNINTENDED PREGNANCIES IN THE MIDDLE EAST AND NORTH AFRICA. 2010;
992. Willard Cates JR, Steiner MJ. Dual protection against unintended pregnancy and sexually transmitted infections: what is the best contraceptive approach? Sex Transm Dis. 2002;29(3):168–74.
993. Amin A. Addressing gender inequalities to improve the sexual and reproductive health and wellbeing of women living with HIV. J Int AIDS Soc. 2015;18:20302.
994. Ezeh AC, Mberu BU, Emina JO. Stall in fertility decline in Eastern African countries: regional analysis of patterns, determinants and implications. Philos Trans R Soc B Biol Sci. 2009;364(1532):2991–3007.
995. Hofmeyr GJ, Singata-Madliki M, Lawrie TA, Bergel E, Temmerman M. Effects of the copper intrauterine device versus injectable progestin contraception on pregnancy rates and method discontinuation among women attending termination of pregnancy services in South Africa: a pragmatic randomized controlled trial. Reprod Health. 2016;13:1–8.
996. Knopf AS, McNealy KR, Al-Khattab H, Carter-Harris L, Oruche UM, Naanyu V, et al. Sexual learning among East African adolescents in the context of generalized HIV epidemics: A systematic qualitative meta-synthesis. PLoS One. 2017;12(3):e0173225.
997. Lusi O, Ronen K, Larsen AM, Richardson B, Khasimwa B, Chohan B, et al. Antenatal depressive symptoms in Kenyan women living with HIV: contributions of recent HIV diagnosis, stigma, and partner violence. 2022;
998. Grabbe K, Stephenson R, Vwalika B, Ahmed Y, Vwalika C, Chomba E, et al. Knowledge, use, and concerns about contraceptive methods among sero-discordant couples in Rwanda and Zambia. J women’s Heal. 2009;18(9):1449–56.
999. Ivanova O, Rai M, Kemigisha E. A systematic review of sexual and reproductive health knowledge, experiences and access to services among refugee, migrant and displaced girls and young women in Africa. Int J Environ Res Public Health. 2018;15(8):1583.
1000. Debela SM, Adinew YM, Geleta LA, Guye AH. Dual Contraceptive Utilization and Associated Factors among Women Attending Antiretroviral Therapy Clinics in Central Ethiopia, 2022: The Need for a Better Control of Ever-Increasing New Strain of HIV Infection and its Transmission. Int J Women’s Heal Care. 2023;8(1):39–49.
1001. Ampt FH, Mudogo C, Gichangi P, Lim MSC, Manguro G, Chersich M, et al. WHISPER or SHOUT study: protocol of a cluster-randomised controlled trial assessing mHealth sexual reproductive health and nutrition interventions among female sex workers in Mombasa, Kenya. BMJ Open. 2017;7(8):e017388.
1002. Huertas-Zurriaga A, Palmieri PA, Aguayo-Gonzalez MP, Dominguez-Cancino KA, Casanovas-Cuellar C, Linden KL Vander, et al. Reproductive decision-making of Black women living with HIV: A systematic review. Women’s Heal. 2022;18:17455057221090828.
1003. Dugg P, Chhabra P, Sharma AK. Contraceptive use and unmet need for family planning among HIV-positive women: a hospital-based study. Indian J Public Health. 2020;64(1):32–8.
1004. Gebrehiwot SW, Azeze GA, Robles CC, Adinew YM. Utilization of dual contraception method among reproductive age women on antiretroviral therapy in selected public hospitals of Northern Ethiopia. Reprod Health. 2017;14:1–9.
1005. Bouris A, Guilamo-Ramos V, Jaccard J, McCoy W, Aranda D, Pickard A, et al. The feasibility of a clinic-based parent intervention to prevent HIV, sexually transmitted infections, and unintended pregnancies among Latino and African American adolescents. AIDS Patient Care STDS. 2010;24(6):381–7.
1006. Mbuthia CW. Fertility Desires and Contraceptive Practices Among Hiv Positive Adults at Naivasha District Hospital. University of Nairobi; 2010.
1007. Sibanda MY. Factors influencing women living with HIV/AIDS’intention to fall pregnant among those attending the OI/ART clinic in Murambinda, Buhera District, Manicaland Province, Zimbabwe, 2010. 2010;
1008. Bauni EK, Jarabi BO. Family planning and sexual behavior in the era of HIV/AIDS: the case of Nakuru District, Kenya. Wiley Online Library; 2000.
1009. Bergsjø P, Vangen S, Lie RT, Lyatuu R, LIE‐NIELSEN E, Oneko O. Recording of maternal deaths in an East African university hospital. Acta Obstet Gynecol Scand. 2010;89(6):789–93.
1010. Richter DL, Sowell RL, Pluto DM. Factors affecting reproductive decisions of African American women living with HIV. Women Health. 2002;36(1):81–96.
1011. Grossman D, Onono M, Newmann SJ, Blat C, Bukusi EA, Shade SB, et al. Integration of family planning services into HIV care and treatment in Kenya: a cluster-randomized trial. Aids. 2013;27:S77–85.
1012. Omollo C. Determinants of contraceptives preference and use among people living with hiv and aids in rural areas: a study of Nyamarambe division, Kisii county, Kenya. 2021.
1013. Bachanas P, Kidder D, Medley A, Pals SL, Carpenter D, Howard A, et al. Delivering prevention interventions to people living with HIV in clinical care settings: results of a cluster randomized trial in Kenya, Namibia, and Tanzania. AIDS Behav. 2016;20:2110–8.
1014. Adilo TM, Wordofa HM. Prevalence of fertility desire and its associated factors among 15-to 49-year-old people living with HIV/AIDS in Addis Ababa, Ethiopia: a cross-sectional study design. HIV/AIDS-Research Palliat Care. 2017;167–76.
1015. Ashimi AO, Amole TG, Abubakar MY, Ugwa EA. Fertility desire and utilization of family planning methods among HIV‑positive women attending a tertiary hospital in a suburban setting in Northern Nigeria. Trop J Obstet Gynaecol. 2017;34(1):54–60.
1016. Singh S, Bankole A, Woog V. Evaluating the need for sex education in developing countries: sexual behaviour, knowledge of preventing sexually transmitted infections/HIV and unplanned pregnancy. Sex Educ. 2005;5(4):307–31.
1017. Nakakawa F, Mugisha J, Kaaya AN, Tumwesigye NM, Hennessey M. Nutrition education effects on food and nutrition security for women living with HIV/AIDS in Uganda. Food Policy. 2024;128:102715.
1018. Omona K, Muhanuzi G. Factors influencing utilization of modern family planning services by persons living with Human Immunodeficiency Virus at Luwero Hospital, Uganda. Afr Health Sci. 2022;22(3):463–76.
1019. Stephenson R, Vwalika B, Greenberg L, Ahmed Y, Vwalika C, Chomba E, et al. A randomized controlled trial to promote long-term contraceptive use among HIV-serodiscordant and concordant positive couples in Zambia. J women’s Heal. 2011;20(4):567–74.
1020. LYATUU JG. Literature review on factors affecting the utilization of family planning services among women living with HIV in Tanzania. 2012;
1021. Chinaeke EE, Fan-Osuala C, Bathnna M, Ozigbu CE, Olakunde B, Ramadhani HO, et al. Correlates of reported modern contraceptive use among postpartum HIV-positive women in rural Nigeria: an analysis from the MoMent prospective cohort study. Reprod Health. 2019;16:1–11.
1022. Wangima NP. Factors influencing uptake of contraceptive services among undergraduate students aged 18-35 years at Jomo Kenyatta University of Agriculture and Technology, Kenya. COHRED, JKUAT; 2016.
1023. Adinma ED, Adinma JIB-D, Eke NO, Iwuoha C, Akiode A, Oji E. Awareness and use of contraception by women seeking termination of pregnancy in south eastern Nigeria. Asian Pacific J Trop Dis. 2011;1(1):71–5.
1024. Zachek CM, Coelho LE, Domingues RMSM, Clark JL, De Boni RB, Luz PM, et al. The intersection of HIV, social vulnerability, and reproductive health: analysis of women living with HIV in Rio de Janeiro, Brazil from 1996 to 2016. AIDS Behav. 2019;23:1541–51.
1025. First B. An exploration of preventive measures of unwanted pregnancies among teenage secondary school girls in Chunya district. The Open University of Tanzania; 2022.
1026. Kaswa R, Rupesinghe GFD, Longo-Mbenza B. Exploring the pregnant women’s perspective of late booking of antenatal care services at Mbekweni Health Centre in Eastern Cape, South Africa. African J Prim Heal Care Fam Med. 2018;10(1):1–9.
1027. Kaida A, Patterson S, Carter A, Loutfy M, Ding E, Sereda P, et al. Contraceptive choice and use of dual protection among women living with HIV in Canada: priorities for integrated care. Perspect Sex Reprod Health. 2017;49(4):223–36.
1028. Chen PF. HIV/AIDS Prevention among Young People in East and South-East Asia in the Context of Reproductive and Sexual Health. Asia-Pacific Popul J. 2008;23(1).
1029. Cuca YP, Onono M, Bukusi E, Turan JM. Factors associated with pregnant women’s anticipations and experiences of HIV-related stigma in rural Kenya. AIDS Care. 2012;24(9):1173–80.
1030. Dabee S, Mugo N, Mudhune V, McLellan-Lemal E, Peacock S, O’Connor S, et al. Genital microbiota of women using a 90 day tenofovir or tenofovir and levonorgestrel intravaginal ring in a placebo controlled randomized safety trial in Kenya. Sci Rep. 2022;12(1):12040.
1031. Onguru PA, Ogungu D, Ouma AT, Onyango OS. Factors Associated With Pregnancy Occurrence Among Known HIV Positive Women In Rangwe Sub-County, Homa Bay County, Kenya.
1032. Biseck T, Kumwenda S, Kalulu K, Chidziwisano K, Kalumbi L. Exploring fertility decisions among pregnant HIVpositive women on antiretroviral therapy at a health centre in Balaka, Malawi: A descriptive qualitative. Malawi Med J. 2015;27(4):128–34.
1033. Narasimhan M, Yeh PT, Haberlen S, Warren CE, Kennedy CE. Integration of HIV testing services into family planning services: a systematic review. Reprod Health. 2019;16:1–12.
1034. Twimukye A, Alhassan Y, Ringwald B, Malaba T, Myer L, Waitt C, et al. Support, not blame: safe partner disclosure among women diagnosed with HIV late in pregnancy in South Africa and Uganda. AIDS Res Ther. 2024;21(1):14.
1035. Kefale B, Kefale Y. Knowledge, attitude, practice and determinants of condom use among people living with HIV/AIDS in Gondar University Hospital, North West Ethiopia. J Phys Pharm Adv. 2013;3(10):247–60.
1036. Erku TA, Megabiaw B, Wubshet M. Predictors of HIV status disclosure to sexual partners among people living with HIV/AIDS in Ethiopia. Pan Afr Med J. 2012;13.
1037. Wagner AC, Ivanova EL, Hart TA, Loutfy MR. Examining the Traits-Desires-Intentions-Behavior (TDIB) model for fertility planning in women living with HIV in Ontario, Canada. AIDS Patient Care STDS. 2014;28(11):594–601.
1038. Kreitchmann R, Megazzini K, Melo VH, Coelho DF, Watts DH, Krauss M, et al. Repeat pregnancy in women with HIV infection in Latin America and the Caribbean. AIDS Care. 2015;27(10):1289–97.
1039. Mwalabu G, Evans C, Redsell S. Factors influencing the experience of sexual and reproductive healthcare for female adolescents with perinatally-acquired HIV: a qualitative case study. BMC Womens Health. 2017;17:1–13.
1040. LUMONJE IK. Utilization of the sexual and reproductive health care services among youth living on the streets in Nakuru County, Kenya. KENYATTA UNIVERSITY; 2020.
1041. Nsubuga H, Sekandi JN, Sempeera H, Makumbi FE. Contraceptive use, knowledge, attitude, perceptions and sexual behavior among female University students in Uganda: a cross-sectional survey. BMC Womens Health. 2015;16:1–11.
1042. Yonge SA, Opiyo B, Kibira AW, Sharma RR. Utilization of Reproductive Health Services and Associated Factors among Youths in Mombasa County, Kenya. Int J Trop Dis Heal. 2023;44(14):41–50.
1043. Psaros C, Remmert JE, Bangsberg DR, Safren SA, Smit JA. Adherence to HIV care after pregnancy among women in sub-Saharan Africa: falling off the cliff of the treatment cascade. Curr HIV/AIDS Rep. 2015;12:1–5.
1044. Otieno AJ, Karanja S, Kagira J. Knowledge and attitude as determinant factors in HIV care among pregnant women in Rachuonyo North, Homa-Bay County, Kenya. East Afr Med J. 2018;95(4):1413–25.
1045. Okech TC, Wawire NW, Mburu TK. Contraceptive use among women of reproductive age in Kenya’s city slums. Int J Bus Soc Sci. 2011;2(1).
1046. ADENUGA FA. CONTRACEPTIVE USE AMONG WOMEN LIVING WITH HIV AND AIDS RECEIVING CARE AT SECONDARY AND TERTIARY HEALTH FACILITIES IN IBADAN NIGERIA. 2016.
1047. Zgambo M, Kalembo FW, Mbakaya BC. Risky behaviours and their correlates among adolescents living with HIV in sub-Saharan Africa: a systematic review. Reprod Health. 2018;15:1–12.
1048. Ambade PN, Hajjar J, Adjei NK, Yaya S. Using the Family Planning Estimation Tool (FPET) to assess national-level family planning trends and future projections for contraceptive prevalence and associated demand for HIV-infected women in sub-Saharan Africa. PLOS Glob Public Heal. 2024;4(8):e0002637.
1049. DeJong J, Shepard B, Roudi-Fahimi F, Ashford L. Young people’s sexual and reproductive health in the Middle East and North Africa. Reprod Heal. 2007;14(78):8.
1050. Kaida A, Laher F, Strathdee SA, Money D, Janssen PA, Hogg RS, et al. Contraceptive use and method preference among women in Soweto, South Africa: the influence of expanding access to HIV care and treatment services. PLoS One. 2010;5(11):e13868.
1051. Nalwadda G, Mirembe F, Byamugisha J, Faxelid E. Persistent high fertility in Uganda: young people recount obstacles and enabling factors to use of contraceptives. BMC Public Health. 2010;10:1–13.
1052. Gust DA, Gvetadze R, Furtado M, Makanga M, Akelo V, Ondenge K, et al. Factors associated with psychological distress among young women in Kisumu, Kenya. Int J Womens Health. 2017;255–64.
1053. Gari T, Habte D, Markos E. HIV positive status disclosure among women attending art clinic at Hawassa University Referral Hospital, South Ethiopia. East Afr J Public Health. 2010;7(1).
1054. Ochako R, Mbondo M, Aloo S, Kaimenyi S, Thompson R, Temmerman M, et al. Barriers to modern contraceptive methods uptake among young women in Kenya: a qualitative study. BMC Public Health. 2015;15:1–9.
1055. Bambra CS. Current status of reproductive behaviour in Africa. Hum Reprod Update. 1999;5(1):1–20.
1056. Ahinkorah BO, Hagan Jr JE, Seidu A-A, Sambah F, Adoboi F, Schack T, et al. Female adolescents’ reproductive health decision-making capacity and contraceptive use in sub-Saharan Africa: What does the future hold? PLoS One. 2020;15(7):e0235601.
1057. Aluzimbi G, Barker J, King R, Rutherford G, Ssenkusu JM, Lubwama GW, et al. Risk factors for unplanned sex among university students in Kampala, Uganda: a qualitative study. Int J Adolesc Youth. 2013;18(3):191–203.
1058. Bongomin F, Pebolo PF, Kibone W, Apio PO, Nsenga L, Okot J, et al. Dual contraceptive use and associated factors among female sex workers in Gulu City, Uganda in 2023 Trop Med Health. 2023;51(1):45.
1059. Biddlecom AE, Hessburg L, Singh S, Bankole A. Protecting the Next Generation in Sub-Saharan AfricaLearning from Adolescents to Prevent HIV and Unintended Pregnancy. 2007;
1060. Kyaw KWY, Mon AA, Phyo KH, Kyaw NTT, Kumar AM V, Lwin TT, et al. Initiation of antiretroviral therapy or antiretroviral prophylaxis in pregnant women living with HIV registered in five townships of Mandalay, Myanmar: A cross sectional study. BMC Pregnancy Childbirth. 2019;19:1–9.
1061. Nzioka C. Perspectives of adolescent boys on the risks of unwanted pregnancy and sexually transmitted infections: Kenya. Reprod Health Matters. 2001;9(17):108–17.
1062. Eyakuze C, Jones DA, Starrs AM, Sorkin N. From PMTCT to a more comprehensive AIDS response for women: a much‐needed shift. Dev World Bioeth. 2008;8(1):33–42.
1063. Duby Z, McClinton Appollis T, Jonas K, Maruping K, Dietrich J, LoVette A, et al. “As a young pregnant girl… the challenges you face”: exploring the intersection between mental health and sexual and reproductive health amongst adolescent girls and young women in South Africa. AIDS Behav. 2021;25:344–53.
1064. Strachan M, Kwateng-Addo A, Hardee K, Subramaniam S, Judice N, Agarwal K. An analysis of family planning content in HIV/AIDS, VCT, and PMTCT policies in 16 countries. POLICY Work Pap Ser. 2004;(9):28–30.
1065. Amare T, Tessema F, Shaweno T. Trend of Unintended Pregnancy, Induced Abortion and Associated Factors among Adolescents in Ethiopia: Evidence from the 2000, 2005, 2011 and 2016 EDHS Data. 2022;
1066. NEEDS U. SEXUAL AND REPRODUCTIVE HEALTH OF WOMEN LIVING WITH HIV IN EGYPT.
1067. Bell E, Mthembu P, O’Sullivan S, Moody K, HIV/AIDS IC of WL with. Sexual and reproductive health services and HIV testing: perspectives and experiences of women and men living with HIV and AIDS. Reprod Health Matters. 2007;15(29):113–35.
1068. Kalayu H. Dual Contraceptive Use and Associated Factors among HIV Positive Women on Art Follow up In Mekelle Town Tigray, Ethiopia. 2017;
1069. Regassa T, Fantahun M. Fertility desire and reproductive health care needs of men and women living with HIV/AIDS in Nekemte, East Wollega, Ethiopia. Sci Technol Arts Res J. 2012;1(3):31–8.
1070. Akwara PA, Madise NJ, Hinde A. Perception of risk of HIV/AIDS and sexual behaviour in Kenya. J Biosoc Sci. 2003;35(3):385–411.
1071. Rasch V, Silberschmidt M, Mchumvu Y, Mmary V. Adolescent girls with illegally induced abortion Dar es Salaam: The discrepancy between sexual behaviour and lack of access to contraception. Reprod Health Matters. 2000;8(15):52–62.
1072. Kumar S, Gruskin S, Khosla R, Narasimhan M. Human rights and the sexual and reproductive health of women living with HIV–a literature review. J Int AIDS Soc. 2015;18:20290.
1073. Onyeka IN, Miettola J, Vaskilampi T, Ilika AL. Unintended pregnancy and termination of studies among students in Anambra state, Nigeria: Are secondary schools playing their part? Afr J Reprod Health. 2011;15(2):109–15.
1074. Mutiso SM, Kinuthia J, Qureshi Z. Contraceptive use among HIV infected women attending Comprehensive Care Centre. East Afr Med J. 2008;85(4):171–7.
1075. Omollo C, Shivachi T, Agwanda AO. Genderized Perspectives on Contraceptive Use: An Exploratory Study of Persons Living with HIV in Rural Kenya. 2021;
1076. Roxby AC, Unger JA, Slyker JA, Kinuthia J, Lewis A, John-Stewart G, et al. A lifecycle approach to HIV prevention in African women and children. Curr HIV/AIDS Rep. 2014;11:119–27.
1077. Peel J, de Gersigny JB, Teague R, Howard J, Bradshaw C, Chen M, et al. Reproductive health among women living with HIV attending Melbourne Sexual Health Centre for HIV care from February 2019 to February 2020. Sex Health. 2024;21(1):NULL-NULL.
1078. Mohamed SF, Izugbara C, Moore AM, Mutua M, Kimani-Murage EW, Ziraba AK, et al. The estimated incidence of induced abortion in Kenya: a cross-sectional study. BMC Pregnancy Childbirth. 2015;15:1–10.
1079. Smith JM, Moss JA, Srinivasan P, Butkyavichene I, Gunawardana M, Fanter R, et al. Novel multipurpose pod-intravaginal ring for the prevention of HIV, HSV, and unintended pregnancy: Pharmacokinetic evaluation in a macaque model. PLoS One. 2017;1210):e0185946.
1080. Worku A, Addisie M. Sexual violence among female high school students in Debark, northwest Ethiopia. East Afr Med J. 2002;79(2):96–9.
1081. Newmann SJ, Zakaras JM, Tao AR, Onono M, Bukusi EA, Cohen CR, et al. Integrating family planning into HIV care in western Kenya: HIV care providers’ perspectives and experiences one year following integration. AIDS Care. 2016;28(2):209–13.
1082. Toroitich-Ruto C. The determinants of teenage sexuality and their understanding of STDs/HIV/AIDS in Kenya. 1997;
1083. Pallitto CC, O’Campo P. The relationship between intimate partner violence and unintended pregnancy: analysis of a national sample from Colombia. Int Fam Plan Perspect. 2004;165–73.
1084. King R, Katuntu D, Lifshay J, Packel L, Batamwita R, Nakayiwa S, et al. Processes and outcomes of HIV serostatus disclosure to sexual partners among people living with HIV in Uganda. AIDS Behav. 2008;12:232–43.
1085. McCoy SI, Ralph LJ, Wilson W, Padian NS. Alcohol production as an adaptive livelihood strategy for women farmers in Tanzania and its potential for unintended consequences on women’s reproductive health. PLoS One. 2013;8(3):e59343.
1086. Genemo ES, Korsa AT, Bayisa HG. Emergency contraceptive pill use and its impact on condom utilization among university students: a cross-sectional study. Int J Womens Health. 2022;1115–26.
1087. Gichangi PB. Reproductive health awareness among adolescents. East Afr Med J. 2003;80(7):337–8.
1088. Matheson R, Moses‐Burton S, Hsieh AC, Dilmitis S, Happy M, Sinyemu E, et al. Fundamental concerns of women living with HIV around the implementation of Option B+. J Int AIDS Soc. 2015;18:20286.
1089. Cuinhane CE, Roelens K, Vanroelen C, Quive S, Coene G. Perceptions and decision-making with regard to pregnancy among HIV positive women in rural Maputo Provinc, Mozambique–a qualitative study. BMC Womens Health. 2018;18:1–21.
1090. Landolta NK, Phanuphaka N, Chaithongwongwatthanab S, Ananworanicha J. Sexual life and contraception in people living with HIV. Health (Irvine Calif). 1:2.
1091. Jozani ZB, Bayanolhagh S, Mobaderi T, Pashangzadeh S, Sohrabi A, Tabatabai RA, et al. Effect of Hormonal Contraception on Disease Progression in Women Living With HIV in Tehran, Iran. 2020;
1092. Lufuluabo NA. Role of contraception in HIV prevention. Stellenbosch: Stellenbosch University; 2013.
1093. Erickson M, Goldenberg SM, Ajok M, Muldoon KA, Muzaaya G, Shannon K. Structural determinants of dual contraceptive use among female sex workers in Gulu, northern Uganda. Int J Gynecol Obstet. 2015;131(1):91–5.
1094. Somba MJ, Mbonile M, Obure J, Mahande MJ. Sexual behaviour, contraceptive knowledge and use among female undergraduates’ students of Muhimbili and Dar es Salaam Universities, Tanzania: a cross-sectional study. BMC Womens Health. 2014;14:1–8.
1095. Manzini N. Sexual initiation and childbearing among adolescent girls in KwaZulu Natal, South Africa. Reprod Health Matters. 2001;9(17):44–52.
1096. Schaan MM, Taylor M, Gungqisa N, Marlink R. Personal views about womanhood amongst women living with HIV in Botswana. Cult Health Sex. 2016;18(2):171–83.
1097. Tibebu NS, Kassie BA, Anteneh TA, Rade BK. Depression, anxiety and stress among HIV-positive pregnant women in Ethiopia during the COVID-19 pandemic. Trans R Soc Trop Med Hyg. 2023;117(5):317–25.
1098. Ofurum IC. Sexual Behaviour, Needs and Concerns Regarding Sexual and Reproductive Health among Adults Living with HIV in Sub-Saharan Africa-A Systematic Review. J Adv Med Med Res. 2021;33(11):113–32.
1099. Kownaklai J, Graham M, Hayter M. Pregnancy decision making among Thai women living with HIV: a grounded theory study. Midwifery. 2022;115:103490.
1100. Esplen E. Women and girls living with HIV/AIDS: overview and annotated bibliography. Institute of Development Studies at the University of Sussex Brighton, UK; 2007.
1101. Bouris A, Guilamo-Ramos V, Jaccard J, McCoy W, Aranda D, Pickard A, et al. The feasibility of a clinic-based parent intervention to prevent HIV, sexually transmitted infections, and unintended pregnancies among Latino and African American adolescents. AIDS Patient Care STDS. 2010;24(6):381–7.
1102. Mbuthia CW. Fertility Desires and Contraceptive Practices Among Hiv Positive Adults at Naivasha District Hospital. University of Nairobi; 2010.
1103. Sibanda MY. Factors influencing women living with HIV/AIDS’intention to fall pregnant among those attending the OI/ART clinic in Murambinda, Buhera District, Manicaland Province, Zimbabwe, 2010. 2010;
1104. Bauni EK, Jarabi BO. Family planning and sexual behavior in the era of HIV/AIDS: the case of Nakuru District, Kenya. Wiley Online Library; 2000.
1105. Bergsjø P, Vangen S, Lie RT, Lyatuu R, LIE‐NIELSEN E, Oneko O. Recording of maternal deaths in an East African university hospital. Acta Obstet Gynecol Scand. 2010;89(6):789–93.
1106. Richter DL, Sowell RL, Pluto DM. Factors affecting reproductive decisions of African American women living with HIV. Women Health. 2002;36(1):81–96.
1107. Grossman D, Onono M, Newmann SJ, Blat C, Bukusi EA, Shade SB, et al. Integration of family planning services into HIV care and treatment in Kenya: a cluster-randomized trial. Aids. 2013;27:S77–85.
1108. Omollo C. Determinants of contraceptives preference and use among people living with hiv and aids in rural areas: a study of Nyamarambe division, Kisii county, Kenya. 2021.
1109. Bachanas P, Kidder D, Medley A, Pals SL, Carpenter D, Howard A, et al. Delivering prevention interventions to people living with HIV in clinical care settings: results of a cluster randomized trial in Kenya, Namibia, and Tanzania. AIDS Behav. 2016;20:2110–8.
1110. Adilo TM, Wordofa HM. Prevalence of fertility desire and its associated factors among 15-to 49-year-old people living with HIV/AIDS in Addis Ababa, Ethiopia: a cross-sectional study design. HIV/AIDS-Research Palliat Care. 2017;167–76.
1111. Ashimi AO, Amole TG, Abubakar MY, Ugwa EA. Fertility desire and utilization of family planning methods among HIV‑positive women attending a tertiary hospital in a suburban setting in Northern Nigeria. Trop J Obstet Gynaecol. 2017;34(1):54–60.
1112. Singh S, Bankole A, Woog V. Evaluating the need for sex education in developing countries: sexual behaviour, knowledge of preventing sexually transmitted infections/HIV and unplanned pregnancy. Sex Educ. 2005;5(4):307–31.
1113. Nakakawa F, Mugisha J, Kaaya AN, Tumwesigye NM, Hennessey M. Nutrition education effects on food and nutrition security for women living with HIV/AIDS in Uganda. Food Policy. 2024;128:102715.
1114. Omona K, Muhanuzi G. Factors influencing utilization of modern family planning services by persons living with Human Immunodeficiency Virus at Luwero Hospital, Uganda. Afr Health Sci. 2022;22(3):463–76.
1115. Stephenson R, Vwalika B, Greenberg L, Ahmed Y, Vwalika C, Chomba E, et al. A randomized controlled trial to promote long-term contraceptive use among HIV-serodiscordant and concordant positive couples in Zambia. J women’s Heal. 2011;20(4):567–74.
1116. LYATUU JG. Literature review on factors affecting the utilization of family planning services among women living with HIV in Tanzania. 2012;
1117. Chinaeke EE, Fan-Osuala C, Bathnna M, Ozigbu CE, Olakunde B, Ramadhani HO, et al. Correlates of reported modern contraceptive use among postpartum HIV-positive women in rural Nigeria: an analysis from the MoMent prospective cohort study. Reprod Health. 2019;16:1–11.
1118. Wangima NP. Factors influencing uptake of contraceptive services among undergraduate students aged 18-35 years at Jomo Kenyatta University of Agriculture and Technology, Kenya. COHRED, JKUAT; 2016.
1119. Adinma ED, Adinma JIB-D, Eke NO, Iwuoha C, Akiode A, Oji E. Awareness and use of contraception by women seeking termination of pregnancy in south eastern Nigeria. Asian Pacific J Trop Dis. 2011;1(1):71–5.
1120. Zachek CM, Coelho LE, Domingues RMSM, Clark JL, De Boni RB, Luz PM, et al. The intersection of HIV, social vulnerability, and reproductive health: analysis of women living with HIV in Rio de Janeiro, Brazil from 1996 to 2016. AIDS Behav. 2019;23:1541–51.
1121. First B. An exploration of preventive measures of unwanted pregnancies among teenage secondary school girls in Chunya district. The Open University of Tanzania; 2022.
1122. Kaswa R, Rupesinghe GFD, Longo-Mbenza B. Exploring the pregnant women’s perspective of late booking of antenatal care services at Mbekweni Health Centre in Eastern Cape, South Africa. African J Prim Heal Care Fam Med. 2018;10(1):1–9.
1123. Kaida A, Patterson S, Carter A, Loutfy M, Ding E, Sereda P, et al. Contraceptive choice and use of dual protection among women living with HIV in Canada: priorities for integrated care. Perspect Sex Reprod Health. 2017;49(4):223–36.
1124. Chen PF. HIV/AIDS Prevention among Young People in East and South-East Asia in the Context of Reproductive and Sexual Health. Asia-Pacific Popul J. 2008;23(1).
1125. Cuca YP, Onono M, Bukusi E, Turan JM. Factors associated with pregnant women’s anticipations and experiences of HIV-related stigma in rural Kenya. AIDS Care. 2012;24(9):1173–80.
1126. Dabee S, Mugo N, Mudhune V, McLellan-Lemal E, Peacock S, O’Connor S, et al. Genital microbiota of women using a 90 day tenofovir or tenofovir and levonorgestrel intravaginal ring in a placebo controlled randomized safety trial in Kenya. Sci Rep. 2022;12(1):12040.
1127. Onguru PA, Ogungu D, Ouma AT, Onyango OS. Factors Associated With Pregnancy Occurrence Among Known HIV Positive Women In Rangwe Sub-County, Homa Bay County, Kenya.
1128. Biseck T, Kumwenda S, Kalulu K, Chidziwisano K, Kalumbi L. Exploring fertility decisions among pregnant HIVpositive women on antiretroviral therapy at a health centre in Balaka, Malawi: A descriptive qualitative. Malawi Med J. 2015;27(4):128–34.
1129. Narasimhan M, Yeh PT, Haberlen S, Warren CE, Kennedy CE. Integration of HIV testing services into family planning services: a systematic review. Reprod Health. 2019;16:1–12.
1130. Twimukye A, Alhassan Y, Ringwald B, Malaba T, Myer L, Waitt C, et al. Support, not blame: safe partner disclosure among women diagnosed with HIV late in pregnancy in South Africa and Uganda. AIDS Res Ther. 2024;21(1):14.
1131. Kefale B, Kefale Y. Knowledge, attitude, practice and determinants of condom use among people living with HIV/AIDS in Gondar University Hospital, North West Ethiopia. J Phys Pharm Adv. 2013;3(10):247–60.
1132. Erku TA, Megabiaw B, Wubshet M. Predictors of HIV status disclosure to sexual partners among people living with HIV/AIDS in Ethiopia. Pan Afr Med J. 2012;13.
1133. Wagner AC, Ivanova EL, Hart TA, Loutfy MR. Examining the Traits-Desires-Intentions-Behavior (TDIB) model for fertility planning in women living with HIV in Ontario, Canada. AIDS Patient Care STDS. 2014;28(11):594–601.
1134. Kreitchmann R, Megazzini K, Melo VH, Coelho DF, Watts DH, Krauss M, et al. Repeat pregnancy in women with HIV infection in Latin America and the Caribbean. AIDS Care. 2015;27(10):1289–97.
1135. Mwalabu G, Evans C, Redsell S. Factors influencing the experience of sexual and reproductive healthcare for female adolescents with perinatally-acquired HIV: a qualitative case study. BMC Womens Health. 2017;17:1–13.
1136. LUMONJE IK. Utilization of the sexual and reproductive health care services among youth living on the streets in Nakuru County, Kenya. KENYATTA UNIVERSITY; 2020.
1137. Nsubuga H, Sekandi JN, Sempeera H, Makumbi FE. Contraceptive use, knowledge, attitude, perceptions and sexual behavior among female University students in Uganda: a cross-sectional survey. BMC Womens Health. 2015;16:1–11.
1138. Yonge SA, Opiyo B, Kibira AW, Sharma RR. Utilization of Reproductive Health Services and Associated Factors among Youths in Mombasa County, Kenya. Int J Trop Dis Heal. 2023;44(14):41–50.
1139. Psaros C, Remmert JE, Bangsberg DR, Safren SA, Smit JA. Adherence to HIV care after pregnancy among women in sub-Saharan Africa: falling off the cliff of the treatment cascade. Curr HIV/AIDS Rep. 2015;12:1–5.
1140. Otieno AJ, Karanja S, Kagira J. Knowledge and attitude as determinant factors in HIV care among pregnant women in Rachuonyo North, Homa-Bay County, Kenya. East Afr Med J. 2018;95(4):1413–25.
1141. Okech TC, Wawire NW, Mburu TK. Contraceptive use among women of reproductive age in Kenya’s city slums. Int J Bus Soc Sci. 2011;2(1).
1142. ADENUGA FA. CONTRACEPTIVE USE AMONG WOMEN LIVING WITH HIV AND AIDS RECEIVING CARE AT SECONDARY AND TERTIARY HEALTH FACILITIES IN IBADAN NIGERIA. 2016.
1143. Zgambo M, Kalembo FW, Mbakaya BC. Risky behaviours and their correlates among adolescents living with HIV in sub-Saharan Africa: a systematic review. Reprod Health. 2018;15:1–12.
1144. Ambade PN, Hajjar J, Adjei NK, Yaya S. Using the Family Planning Estimation Tool (FPET) to assess national-level family planning trends and future projections for contraceptive prevalence and associated demand for HIV-infected women in sub-Saharan Africa. PLOS Glob Public Heal. 2024;4(8):e0002637.
1145. DeJong J, Shepard B, Roudi-Fahimi F, Ashford L. Young people’s sexual and reproductive health in the Middle East and North Africa. Reprod Heal. 2007;14(78):8.
1146. Kaida A, Laher F, Strathdee SA, Money D, Janssen PA, Hogg RS, et al. Contraceptive use and method preference among women in Soweto, South Africa: the influence of expanding access to HIV care and treatment services. PLoS One. 2010;5(11):e13868.
1147. Nalwadda G, Mirembe F, Byamugisha J, Faxelid E. Persistent high fertility in Uganda: young people recount obstacles and enabling factors to use of contraceptives. BMC Public Health. 2010;10:1–13.
1148. Gust DA, Gvetadze R, Furtado M, Makanga M, Akelo V, Ondenge K, et al. Factors associated with psychological distress among young women in Kisumu, Kenya. Int J Womens Health. 2017;255–64.
1149. Gari T, Habte D, Markos E. HIV positive status disclosure among women attending art clinic at Hawassa University Referral Hospital, South Ethiopia. East Afr J Public Health. 2010;7(1).
1150. Ochako R, Mbondo M, Aloo S, Kaimenyi S, Thompson R, Temmerman M, et al. Barriers to modern contraceptive methods uptake among young women in Kenya: a qualitative study. BMC Public Health. 2015;15:1–9.
1151. Bambra CS. Current status of reproductive behaviour in Africa. Hum Reprod Update. 1999;5(1):1–20.
1152. Ahinkorah BO, Hagan Jr JE, Seidu A-A, Sambah F, Adoboi F, Schack T, et al. Female adolescents’ reproductive health decision-making capacity and contraceptive use in sub-Saharan Africa: What does the future hold? PLoS One. 2020;15(7):e0235601.
1153. Aluzimbi G, Barker J, King R, Rutherford G, Ssenkusu JM, Lubwama GW, et al. Risk factors for unplanned sex among university students in Kampala, Uganda: a qualitative study. Int J Adolesc Youth. 2013;18(3):191–203.
1154. Bongomin F, Pebolo PF, Kibone W, Apio PO, Nsenga L, Okot J, et al. Dual contraceptive use and associated factors among female sex workers in Gulu City, Uganda in 2023 Trop Med Health. 2023;51(1):45.
1155. Biddlecom AE, Hessburg L, Singh S, Bankole A. Protecting the Next Generation in Sub-Saharan AfricaLearning from Adolescents to Prevent HIV and Unintended Pregnancy. 2007;
1156. Kyaw KWY, Mon AA, Phyo KH, Kyaw NTT, Kumar AM V, Lwin TT, et al. Initiation of antiretroviral therapy or antiretroviral prophylaxis in pregnant women living with HIV registered in five townships of Mandalay, Myanmar: A cross sectional study. BMC Pregnancy Childbirth. 2019;19:1–9.
1157. Nzioka C. Perspectives of adolescent boys on the risks of unwanted pregnancy and sexually transmitted infections: Kenya. Reprod Health Matters. 2001;9(17):108–17.
1158. Eyakuze C, Jones DA, Starrs AM, Sorkin N. From PMTCT to a more comprehensive AIDS response for women: a much‐needed shift. Dev World Bioeth. 2008;8(1):33–42.
1159. Duby Z, McClinton Appollis T, Jonas K, Maruping K, Dietrich J, LoVette A, et al. “As a young pregnant girl… the challenges you face”: exploring the intersection between mental health and sexual and reproductive health amongst adolescent girls and young women in South Africa. AIDS Behav. 2021;25:344–53.
1160. Strachan M, Kwateng-Addo A, Hardee K, Subramaniam S, Judice N, Agarwal K. An analysis of family planning content in HIV/AIDS, VCT, and PMTCT policies in 16 countries. POLICY Work Pap Ser. 2004;(9):28–30.
1161. Amare T, Tessema F, Shaweno T. Trend of Unintended Pregnancy, Induced Abortion and Associated Factors among Adolescents in Ethiopia: Evidence from the 2000, 2005, 2011 and 2016 EDHS Data. 2022;
1162. NEEDS U. SEXUAL AND REPRODUCTIVE HEALTH OF WOMEN LIVING WITH HIV IN EGYPT.
1163. Bell E, Mthembu P, O’Sullivan S, Moody K, HIV/AIDS IC of WL with. Sexual and reproductive health services and HIV testing: perspectives and experiences of women and men living with HIV and AIDS. Reprod Health Matters. 2007;15(29):113–35.
1164. Kalayu H. Dual Contraceptive Use and Associated Factors among HIV Positive Women on Art Follow up In Mekelle Town Tigray, Ethiopia. 2017;
1165. Regassa T, Fantahun M. Fertility desire and reproductive health care needs of men and women living with HIV/AIDS in Nekemte, East Wollega, Ethiopia. Sci Technol Arts Res J. 2012;1(3):31–8.
1166. Akwara PA, Madise NJ, Hinde A. Perception of risk of HIV/AIDS and sexual behaviour in Kenya. J Biosoc Sci. 2003;35(3):385–411.
1167. Rasch V, Silberschmidt M, Mchumvu Y, Mmary V. Adolescent girls with illegally induced abortion Dar es Salaam: The discrepancy between sexual behaviour and lack of access to contraception. Reprod Health Matters. 2000;8(15):52–62.
1168. Kumar S, Gruskin S, Khosla R, Narasimhan M. Human rights and the sexual and reproductive health of women living with HIV–a literature review. J Int AIDS Soc. 2015;18:20290.
1169. Onyeka IN, Miettola J, Vaskilampi T, Ilika AL. Unintended pregnancy and termination of studies among students in Anambra state, Nigeria: Are secondary schools playing their part? Afr J Reprod Health. 2011;15(2):109–15.
1170. Mutiso SM, Kinuthia J, Qureshi Z. Contraceptive use among HIV infected women attending Comprehensive Care Centre. East Afr Med J. 2008;85(4):171–7.
1171. Omollo C, Shivachi T, Agwanda AO. Genderized Perspectives on Contraceptive Use: An Exploratory Study of Persons Living with HIV in Rural Kenya. 2021;
1172. Roxby AC, Unger JA, Slyker JA, Kinuthia J, Lewis A, John-Stewart G, et al. A lifecycle approach to HIV prevention in African women and children. Curr HIV/AIDS Rep. 2014;11:119–27.
1173. Peel J, de Gersigny JB, Teague R, Howard J, Bradshaw C, Chen M, et al. Reproductive health among women living with HIV attending Melbourne Sexual Health Centre for HIV care from February 2019 to February 2020. Sex Health. 2024;21(1):NULL-NULL.
1174. Mohamed SF, Izugbara C, Moore AM, Mutua M, Kimani-Murage EW, Ziraba AK, et al. The estimated incidence of induced abortion in Kenya: a cross-sectional study. BMC Pregnancy Childbirth. 2015;15:1–10.
1175. Smith JM, Moss JA, Srinivasan P, Butkyavichene I, Gunawardana M, Fanter R, et al. Novel multipurpose pod-intravaginal ring for the prevention of HIV, HSV, and unintended pregnancy: Pharmacokinetic evaluation in a macaque model. PLoS One. 2017;1210):e0185946.
1176. Worku A, Addisie M. Sexual violence among female high school students in Debark, northwest Ethiopia. East Afr Med J. 2002;79(2):96–9.
1177. Newmann SJ, Zakaras JM, Tao AR, Onono M, Bukusi EA, Cohen CR, et al. Integrating family planning into HIV care in western Kenya: HIV care providers’ perspectives and experiences one year following integration. AIDS Care. 2016;28(2):209–13.
1178. Toroitich-Ruto C. The determinants of teenage sexuality and their understanding of STDs/HIV/AIDS in Kenya. 1997;
1179. Pallitto CC, O’Campo P. The relationship between intimate partner violence and unintended pregnancy: analysis of a national sample from Colombia. Int Fam Plan Perspect. 2004;165–73.
1180. King R, Katuntu D, Lifshay J, Packel L, Batamwita R, Nakayiwa S, et al. Processes and outcomes of HIV serostatus disclosure to sexual partners among people living with HIV in Uganda. AIDS Behav. 2008;12:232–43.
1181. McCoy SI, Ralph LJ, Wilson W, Padian NS. Alcohol production as an adaptive livelihood strategy for women farmers in Tanzania and its potential for unintended consequences on women’s reproductive health. PLoS One. 2013;8(3):e59343.
1182. Genemo ES, Korsa AT, Bayisa HG. Emergency contraceptive pill use and its impact on condom utilization among university students: a cross-sectional study. Int J Womens Health. 2022;1115–26.
1183. Gichangi PB. Reproductive health awareness among adolescents. East Afr Med J. 2003;80(7):337–8.
1184. Matheson R, Moses‐Burton S, Hsieh AC, Dilmitis S, Happy M, Sinyemu E, et al. Fundamental concerns of women living with HIV around the implementation of Option B+. J Int AIDS Soc. 2015;18:20286.
1185. Cuinhane CE, Roelens K, Vanroelen C, Quive S, Coene G. Perceptions and decision-making with regard to pregnancy among HIV positive women in rural Maputo Provinc, Mozambique–a qualitative study. BMC Womens Health. 2018;18:1–21.
1186. Landolta NK, Phanuphaka N, Chaithongwongwatthanab S, Ananworanicha J. Sexual life and contraception in people living with HIV. Health (Irvine Calif). 1:2.
1187. Jozani ZB, Bayanolhagh S, Mobaderi T, Pashangzadeh S, Sohrabi A, Tabatabai RA, et al. Effect of Hormonal Contraception on Disease Progression in Women Living With HIV in Tehran, Iran. 2020;
1188. Lufuluabo NA. Role of contraception in HIV prevention. Stellenbosch: Stellenbosch University; 2013.
1189. Erickson M, Goldenberg SM, Ajok M, Muldoon KA, Muzaaya G, Shannon K. Structural determinants of dual contraceptive use among female sex workers in Gulu, northern Uganda. Int J Gynecol Obstet. 2015;131(1):91–5.
1190. Somba MJ, Mbonile M, Obure J, Mahande MJ. Sexual behaviour, contraceptive knowledge and use among female undergraduates’ students of Muhimbili and Dar es Salaam Universities, Tanzania: a cross-sectional study. BMC Womens Health. 2014;14:1–8.
1191. Manzini N. Sexual initiation and childbearing among adolescent girls in KwaZulu Natal, South Africa. Reprod Health Matters. 2001;9(17):44–52.
1192. Schaan MM, Taylor M, Gungqisa N, Marlink R. Personal views about womanhood amongst women living with HIV in Botswana. Cult Health Sex. 2016;18(2):171–83.
1193. Tibebu NS, Kassie BA, Anteneh TA, Rade BK. Depression, anxiety and stress among HIV-positive pregnant women in Ethiopia during the COVID-19 pandemic. Trans R Soc Trop Med Hyg. 2023;117(5):317–25.
1194. Ofurum IC. Sexual Behaviour, Needs and Concerns Regarding Sexual and Reproductive Health among Adults Living with HIV in Sub-Saharan Africa-A Systematic Review. J Adv Med Med Res. 2021;33(11):113–32.
1195. Kownaklai J, Graham M, Hayter M. Pregnancy decision making among Thai women living with HIV: a grounded theory study. Midwifery. 2022;115:103490.
1196. Esplen E. Women and girls living with HIV/AIDS: overview and annotated bibliography. Institute of Development Studies at the University of Sussex Brighton, UK; 2007.
1197. Wado YD, Bangha M, Kabiru CW, Feyissa GT. Nature of, and responses to key sexual and reproductive health challenges for adolescents in urban slums in sub-Saharan Africa: a scoping review. Reprod Health. 2020;17:1–14.
1198. Organization WH. Sexual and reproductive health of women living with HIV. 2006;
1199. Reis RK, Melo ES, Gir E. Factors associated with inconsistent condom use among people living with HIV/Aids. Rev Bras Enferm. 2016;69:47–53.
1200. Lema VM, Mpanga V, Makanani BS. Socio-demographic characterists of adolescent post-abortion patients in Blantyre, Malawi. East Afr Med J. 2002;79(6):306–10.
1201. Brookman-Amissah E, Moyo JB. Abortion law reform in sub-Saharan Africa: no turning back. Reprod Health Matters. 2004;12(24):227–34.
1202. Richter L, Komárek A, Desmond C, Celentano D, Morin S, Sweat M, et al. Reported physical and sexual abuse in childhood and adult HIV risk behaviour in three African countries: findings from Project Accept (HPTN-043). AIDS Behav. 2014;18:381–9.
1203. Derek A, Seme A, Anye CS, Nkfusai CN, Cumber SN. Modern family planning use among people living with HIV/AIDS: a facility based study in Ethiopia. Pan Afr Med J. 2019;33(1).
1204. Mbizvo MT, Zaidi S. Addressing critical gaps in achieving universal access to sexual and reproductive health (SRH): the case for improving adolescent SRH, preventing unsafe abortion, and enhancing linkages between SRH and HIV interventions. Int J Gynecol Obstet. 2010;110:S3–6.
1205. Ngom P, Magadi MA, Owuor T. Parental presence and adolescent reproductive health among the Nairobi urban poor. J Adolesc Heal. 2003;33(5):369–77.
1206. Clark JT, Clark MR, Shelke NB, Johnson TJ, Smith EM, Andreasen AK, et al. Engineering a segmented dual-reservoir polyurethane intravaginal ring for simultaneous prevention of HIV transmission and unwanted pregnancy. PLoS One. 2014;9(3):e88509.
1207. Ikamari LD, Towett R. Sexual initiation and contraceptive use among female adolescents in Kenya. Afr J Health Sci. 2007;14(1):1–13.
1208. Hlongwa M, Mashamba-Thompson T, Makhunga S, Hlongwana K. Evidence on factors influencing contraceptive use and sexual behavior among women in South Africa: a scoping review. Medicine (Baltimore). 2020;99(12):e19490.
1209. Oraby D. Women with HIV Living in the MENA Region. Handb Healthc Arab World. 2021;3157–75.
1210. Chandra-Mouli V, McCarraher DR, Phillips SJ, Williamson NE, Hainsworth G. Contraception for adolescents in low and middle income countries: needs, barriers, and access. Reprod Health. 2014;11:1–8.
1211. Abdallah HT, Khalfan FR. Towards Realization of Women’s Sexual and Reproductive Rights in Tanzania: The Case of HIV and AIDS Act of 2008, its compatibility with International Norms and Standards.
1212. Maharaj P, Munthree C. Coerced first sexual intercourse and selected reproductive health outcomes among young women in KwaZulu-Natal, South Africa. J Biosoc Sci. 2007;39(2):231–44.
1213. Bamgboye EA, Ajayi I. Changing patterns of unmet needs for family planning among women of reproductive age in Nigeria. Afr J Reprod Health. 2016;20(3):127–35.
1214. Shallie PD, Haffejee F. Systematic review and meta-analysis assessing the knowledge and use of the female condom among Nigerians. Afr Health Sci. 2021;21(3):1362–74.
1215. Mrabure KO, Omonoseh A. Recognizing the Inalienable Reproductive Health Rights of Women in Nigeria. Customary and Sharia Law as Hindrances: The Way Forward. Commonw Law Rev J. 2022;8:239–53.
1216. Nordensved J, Dahlqvist J. Sex, a one mans show: Perceptions and experience of sexuality, contraceptives, unwanted pregnancy and unsafe abortion among young people in Kisumu, Kenya–A qualitative study. 2011.
1217. Ezechi OC, Kalejaiye OO, Gab-Okafor CV, Oladele DA, Oke BO, Musa ZA, et al. Sero-prevalence and factors associated with Hepatitis B and C co-infection in pregnant Nigerian women living with HIV Infection. Pan Afr Med J. 2014;17(1).
1218. Ashford LS. How HIV and AIDS affect populations. World. 2006;1:38–600.
1219. Bankole A, Moore AM, Singh S, Mirembe F. Unintended Pregnancy And Induced Abortion In Uganda. 2006;
1220. Turan JM, Miller S, Bukusi EA, Sande J, Cohen CR. HIV/AIDS and maternity care in Kenya: how fears of stigma and discrimination affect uptake and provision of labor and delivery services. AIDS Care. 2008;20(8):938–45.
1221. Mbarushimana V, Ntaganira J. Knowledge and attitude to female condom use among undergraduates of Kigali Health Institute. Rwanda J Heal Sci. 2013;2(1):16–25.
1222. Emaway D. Demand for family planning among women voluntary counseling and testing clients: The need for integretion, Dessie town North East Ethiopia. Ethiopia: AAU; 2009.
1223. Hindin MJ, Fatusi AO. Adolescent sexual and reproductive health in developing countries: an overview of trends and interventions. Int Perspect Sex Reprod Health. 2009;35(2):58–62.
1224. Exavery A, Kanté AM, Jackson E, Noronha J, Sikustahili G, Tani K, et al. Role of condom negotiation on condom use among women of reproductive age in three districts in Tanzania. BMC Public Health. 2012;12:1–11.
1225. Kyaw Min M, MCTM PDPH, Surasak Taneepanichskul MD, FRCOGT KC, Somrongthong R, Damrong Reinprayoon MD. The model development of participatory education on adolescent reproductive life (PEARL) programme to prevent unintended pregnancy among Myanmar migrant adolescent and youth in samut sakhon province, Thailand:(situational analysis). 2011;
1226. Awusabo-Asare K, Biddlecom A, Kumi-Kyereme A, Patterson K. Adolescent sexual and reproductive health in Ghana: results from the 2004 National Survey of Adolescents. Occas Rep. 2006;22.
1227. Mtayangulwa R, Kayombo EJ. Knowledge, attitude and use of female condoms among female undergraduate students in University of Dar-Es-Salaam. J Sci Res Reports. 2015;9(2):1–10.
1228. Otieno AJW, Karanja SM, Kagira J. Socio-cultural factors influencing utilization of prevention-of-mother-to child-transmission of HIV strategies among women attending antenatal care clinics in Rachuonyo North Sub-County-Homa-Bay County, Kenya. East Afr Med J. 2017;94(11):946–59.
1229. Hopkins J, Collins L. How linked are national HIV and SRHR strategies? A review of SRHR and HIV strategies in 60 countries. Health Policy Plan. 2017;32(suppl_4):iv57–66.
1230. Owolabi AT, Onayade AA, Ogunlola IO, Ogunniyi SO, Kuti O. Sexual behaviour of secondary school adolescents in Ilesa, Nigeria: implications for the spread of STIs including HIV/AIDS. J Obstet Gynaecol (Lahore). 2005;25(2):174–8.
1231. Akinsolu FT, Adewole IE, Lawale AA, Olagunju MT, Abodunrin OR, Ola OM, et al. HIV and Pregnancy among Adolescents in Sub-Saharan Africa: A Scoping Review. medRxiv. 2024;2004–24.
1232. Sukeri S, Sulaiman Z, Hamid NA, Ibrahim SA. Decision-making on contraceptive use among women living with human immunodeficiency virus in Malaysia: a qualitative inquiry. Korean J Fam Med. 2024;45(1):27.
1233. Abdullahi IS, Chukwudike CO, Sangari JS, Chikwendu JI, Fulani GJ. The causes of unwanted pregnancy and abortion among female students and its impact on their academic performance in FCE Pankshin, Plateau state, Nigeria.
1234. Shaw D. Access to sexual and reproductive health for young people: bridging the disconnect between rights and reality. Int J Gynecol Obstet. 2009;106(2):132–6.
1235. Warren C. Exploring the quality and effect of comprehensive postnatal care models in East and Southern Africa. Ghent University; 2015.
1236. Wilcher R, Hoke T, Adamchak SE, Cates Jr W. Integration of family planning into HIV services: a synthesis of recent evidence. Aids. 2013;27:S65–75.
1237. Timiun GA. Sexual webs model for the examination of unsafe sexual behaviors and the spread of sexually transmitted diseases including HIV/AIDS. Asian Soc Sci. 2012;8(7):119.
1238. Ayalew M, Mengistie B, Semahegn A. Adolescent-parent communication on sexual and reproductive health issues among high school students in Dire Dawa, Eastern Ethiopia: a cross sectional study. Reprod Health. 2014;11:1–8.
1239. Dabis F, Ekpini ER. HIV-1/AIDS and maternal and child health in Africa. Lancet. 2002;359(9323):2097–104.
1240. Li Y, Marshall CM, Rees HC, Nunez A, Ezeanolue EE, Ehiri JE. Intimate partner violence and HIV infection among women: a systematic review and meta‐analysis. African J Reprod Gynaecol Endosc. 2014;17(1).
1241. Lindegren M Lou, Kennedy CE, Bain‐Brickley D, Azman H, Creanga AA, Butler LM, et al. Integration of HIV/AIDS services with maternal, neonatal and child health, nutrition, and family planning services. Cochrane Database Syst Rev. 1996;2012(10).
1242. Bunnell R, Mermin J, De Cock KM. HIV prevention for a threatened continent: implementing positive prevention in Africa. Jama. 2006;296(7):855–8.
1243. Curtis C. Meeting health care needs of women experiencing complications of miscarriage and unsafe abortion: USAID’s postabortion care program. J Midwifery Womens Health. 2007;52(4):368–75.
1244. Ziraba AK, Madise N, Mills S, Kyobutungi C, Ezeh A. Maternal mortality in the informal settlements of Nairobi city: what do we know? Reprod Health. 2009;6:1–8.
1245. Maharaj P. Obstacles to negotiating dual protection: perspectives of men and women. Afr J Reprod Health. 2001;150–61.
1246. Amelya M, Andriyana H, Nababan B, Gunardi ER. Postpartum contraceptive use among HIV positive women in Cipto Mangunkusumo Hospital Jakarta, Indonesia: A cross sectional study. KnE Med. 2016;161–8.
1247. Ensor S, Mechie I, Ryan R, Mussa A, Bame B, Tamuthiba L, et al. Measuring the impact of COVID-19 social distancing measures on sexual health behaviours and access to HIV and sexual and reproductive health services for people living with HIV in Botswana. Front Glob Women’s Heal. 2023;4:981478.
1248. Harrison A, Colvin CJ, Kuo C, Swartz A, Lurie M. Sustained high HIV incidence in young women in Southern Africa: social, behavioral, and structural factors and emerging intervention approaches. Curr HIV/AIDS Rep. 2015;12:207–15.
1249. Kamangu AA, John MR, Nyakoki SJ. Barriers to parent-child communication on sexual and reproductive health issues in East Africa: A review of qualitative research in four countries. J African Stud Dev. 2017;9(4):45–50.
1250. Karim QA, Havlir D, Phanuphak N. Putting women in the centre of the global HIV response is key to achieving epidemic control! African J Reprod Gynaecol Endosc. 2020;23(3):e25473.
1251. Kungu W, Agwanda A, Khasakhala A. Trends and determinants of contraceptive method choice among women aged 15-24 years in Kenya. F1000Research. 2020;9:197.
1252. Bauni EK, Jarabi BO. The low acceptability and use of condoms within marriage: evidence from Nakuru district, Kenya. 2003;
1253. Anguko AA. Determinants of contraceptive use among women of reproductive age in North Eastern Kenya. University of Nairobi; 2014.
1254. Mondal MNI, Shitan M. Factors affecting the HIV/AIDS epidemic: an ecological analysis of global data. Afr Health Sci. 2013;13(2):301–10.
1255. Thurman AR, Clark MR, Doncel GF. Multipurpose prevention technologies: biomedical tools to prevent HIV‐1, HSV‐2, and unintended pregnancies. Infect Dis Obstet Gynecol. 2011;2011(1):429403.
1256. Muturi NW. Communication for HIV/AIDS prevention in Kenya: Social–cultural considerations. J Health Commun. 2005;10(1):77–98.
1257. Haddad LB, Polis CB, Sheth AN, Brown J, Kourtis AP, King C, et al. Contraceptive methods and risk of HIV acquisition or female-to-male transmission. Curr HIV/AIDS Rep. 2014;11:447–58.
1258. Schwartz SR, Papworth E, Ky-Zerbo O, Sithole B, Anato S, Grosso A, et al. Reproductive health needs of female sex workers and opportunities for enhanced prevention of mother-to-child transmission efforts in sub-Saharan Africa. J Fam Plan Reprod Heal Care. 2017;43(1):50–9.
1259. UNION A. SPECIAL SESSION OF THE AFRICAN UNION CONFERENCE OF MINISTERS OF HEALTH MAPUTO, MOZAMBIQUE 18–22 SEPTEMBER 2006 Sp/MIN/CAMH/4a (I).
1260. Kaljee LM, Green M, Riel R, Lerdboon P, Minh TT. Sexual stigma, sexual behaviors, and abstinence among Vietnamese adolescents: implications for risk and protective behaviors for HIV, sexually transmitted infections, and unwanted pregnancy. J Assoc Nurses AIDS Care. 2007;18(2):48–59.
1261. Ajayi OA, Ogunsola OO, Akinro Y, Adamu-Oyegun S, Wudiri K, Ojo TO, et al. Consistent condom use and associated factors among HIV positive women of reproductive age on anti-retroviral treatment in Ogun State, Nigeria. Pan Afr Med J. 2022;43(1).
1262. Akintade OL, Pengpid S, Peltzer K. Awareness and use of and barriers to family planning services among female university students in Lesotho. S Afr J Obstet Gynaecol. 2011;17(3):72–8.
1263. Myer L, Rabkin M, Abrams EJ, Rosenfield A, El-Sadr WM. Focus on women: linking HIV care and treatment with reproductive health services in the MTCT-Plus Initiative. Reprod Health Matters. 2005;13(25):136–46.
1264. Yaya S, Bishwajit G. Age at first sexual intercourse and multiple sexual partnerships among women in Nigeria: A cross-sectional analysis. Front Med. 2018;5:171.
1265. Bana A, Bhat VG, Godlwana X, Libazi S, Maholwana Y, Marafungana N, et al. Knowledge, attitudes and behaviours of adolescents in relation to STIs, pregnancy, contraceptive utilization and substance abuse in the Mhlakulo region, Eastern Cape. South African Fam Pract. 2010;52(2):154–8.
1266. Wilcher R, Petruney T, Cates W. The role of family planning in elimination of new pediatric HIV infection. Curr Opin HIV AIDS. 2013;8(5):490–7.
1267. Ilika CP, Eleje GU, Anyaoku CS, Ikechukwu E, Okaforcha ICA. Emerging determinants of contraceptive practices among HIV-positive women on antiretroviral therapy in Nigeria. 2018;
1268. Gaughran M, Asgary R. On-site comprehensive curriculum to teach reproductive health to female adolescents in Kenya. J women’s Heal. 2014;23(4):358–64.
1269. Spaulding AB, Brickley DB, Kennedy C, Almers L, Packel L, Mirjahangir J, et al. Linking family planning with HIV/AIDS interventions: a systematic review of the evidence. Aids. 2009;23:S79–88.
1270. Wilcher R, Cates Jr W, Gregson S. Family planning and HIV: strange bedfellows no longer. Aids. 2009;23:S1–6.
1271. Amoah EJ, Hinneh T, Aklie R. Determinants and prevalence of modern contraceptive use among sexually active female youth in the Berekum East Municipality, Ghana. PLoS One. 2023;18(6):e0286585.
1272. Yaya S, Odusina EK, Bishwajit G. Prevalence of child marriage and its impact on fertility outcomes in 34 sub-Saharan African countries. BMC Int Health Hum Rights. 2019;19:1–11.
1273. Medley A, Garcia-Moreno C, McGill S, Maman S. Rates, barriers and outcomes of HIV serostatus disclosure among women in developing countries: implications for prevention of mother-to-child transmission programmes. Bull World Health Organ. 2004;82:299–307.
1274. Fetene N, Mekonnen W. The prevalence of risky sexual behaviors among youth center reproductive health clinics users and non-users in Addis Ababa, Ethiopia: a comparative cross-sectional study. PLoS One. 2018;13(6):e0198657.
1275. Orza L, Bewley S, Chung C, Crone ET, Nagadya H, Vazquez M, et al. “Violence. Enough already”: findings from a global participatory survey among women living with HIV. J Int AIDS Soc. 2015;18:20285.
1276. Nwaorgu OC, Onyeneho NG, Okolo M, Obadike E, Enibe G. Reproductive health knowledge and practices among junior secondary school grade one students in Enugu State: threat to achieving millennium development goals in Nigeria. East Afr J Public Health. 2008;5(2):126–31.
1277. De Vogli R, Birbeck GL. Potential impact of adjustment policies on vulnerability of women and children to HIV/AIDS in sub-Saharan Africa. J Heal Popul Nutr. 2005;105–20.
1278. Olagbuji BN. Meeting the contraceptive needs of HIV positive adolescent females living in urban townships in Western Cape, South Africa: perspectives of clients and primary health care providers. 2020;
1279. Kidayi PL, Msuya S, Todd J, Mtuya CC, Mtuy T, Mahande MJ. Determinants of modern contraceptive use among women of reproductive age in Tanzania: evidence from Tanzania demographic and health survey data. Adv Sex Med. 2015;5(03):43–52.
1280. Ojule JD, Oranu EO, Unamba BC. Contraceptive practice among HIV positive women attending anti-retroviral clinic at the University of Port Harcourt Teaching Hospital, Port Harcourt, southern Nigeria. East Afr Med J. 2015;92(1):9–14.
1281. Doyle AM, Mavedzenge SN, Plummer ML, Ross DA. The sexual behaviour of adolescents in sub‐Saharan Africa: patterns and trends from national surveys. Trop Med Int Heal. 2012;17(7):796–807.
1282. Khosla R, Van Belle N, Temmerman M. Advancing the sexual and reproductive health and human rights of women living with HIV: a review of UN, regional and national human rights norms and standards. J Int AIDS Soc. 2015;18:20280.
1283. Leyva-Moral JM, Palmieri PA, Feijoo-Cid M, Cesario SK, Membrillo-Pillpe NJ, Piscoya-Angeles PN, et al. Reproductive decision-making in women living with human immunodeficiency virus: A systematic review. Int J Nurs Stud. 2018;77:207–21.
1284. Harling G, Muya A, Ortblad KF, Mashasi I, Dambach P, Ulenga N, et al. HIV risk and pre-exposure prophylaxis interest among female bar workers in Dar es Salaam: cross-sectional survey. BMJ Open. 2019;9(3):e023272.
1285. Ghanotakis E, Peacock D, Wilcher R. The importance of addressing gender inequality in efforts to end vertical transmission of HIV. J Int AIDS Soc. 2012;15:17385.
1286. Mchunu G, Peltzer K, Tutshana B, Seutlwadi L. Adolescent pregnancy and associated factors in South African youth. Afr Health Sci. 2012;12(4):426–34.
1287. Carter AJ, Bourgeois S, O’Brien N, Abelsohn K, Tharao W, Greene S, et al. Women‐specific HIV/AIDS services: identifying and defining the components of holistic service delivery for women living with HIV/AIDS. African J Reprod Gynaecol Endosc. 2013;16(1).
1288. Wado YD, Bangha M, Kabiru CW, Feyissa GT. Nature of, and responses to key sexual and reproductive health challenges for adolescents in urban slums in sub-Saharan Africa: a scoping review. Reprod Health. 2020;17:1–14.
1289. Organization WH. Sexual and reproductive health of women living with HIV. 2006;
1290. Reis RK, Melo ES, Gir E. Factors associated with inconsistent condom use among people living with HIV/Aids. Rev Bras Enferm. 2016;69:47–53.
1291. Lema VM, Mpanga V, Makanani BS. Socio-demographic characterists of adolescent post-abortion patients in Blantyre, Malawi. East Afr Med J. 2002;79(6):306–10.
1292. Brookman-Amissah E, Moyo JB. Abortion law reform in sub-Saharan Africa: no turning back. Reprod Health Matters. 2004;12(24):227–34.
1293. Richter L, Komárek A, Desmond C, Celentano D, Morin S, Sweat M, et al. Reported physical and sexual abuse in childhood and adult HIV risk behaviour in three African countries: findings from Project Accept (HPTN-043). AIDS Behav. 2014;18:381–9.
1294. Derek A, Seme A, Anye CS, Nkfusai CN, Cumber SN. Modern family planning use among people living with HIV/AIDS: a facility based study in Ethiopia. Pan Afr Med J. 2019;33(1).
1295. Mbizvo MT, Zaidi S. Addressing critical gaps in achieving universal access to sexual and reproductive health (SRH): the case for improving adolescent SRH, preventing unsafe abortion, and enhancing linkages between SRH and HIV interventions. Int J Gynecol Obstet. 2010;110:S3–6.
1296. Ngom P, Magadi MA, Owuor T. Parental presence and adolescent reproductive health among the Nairobi urban poor. J Adolesc Heal. 2003;33(5):369–77.
1297. Clark JT, Clark MR, Shelke NB, Johnson TJ, Smith EM, Andreasen AK, et al. Engineering a segmented dual-reservoir polyurethane intravaginal ring for simultaneous prevention of HIV transmission and unwanted pregnancy. PLoS One. 2014;9(3):e88509.
1298. Ikamari LD, Towett R. Sexual initiation and contraceptive use among female adolescents in Kenya. Afr J Health Sci. 2007;14(1):1–13.
1299. Hlongwa M, Mashamba-Thompson T, Makhunga S, Hlongwana K. Evidence on factors influencing contraceptive use and sexual behavior among women in South Africa: a scoping review. Medicine (Baltimore). 2020;99(12):e19490.
1300. Oraby D. Women with HIV Living in the MENA Region. Handb Healthc Arab World. 2021;3157–75.
1301. Chandra-Mouli V, McCarraher DR, Phillips SJ, Williamson NE, Hainsworth G. Contraception for adolescents in low and middle income countries: needs, barriers, and access. Reprod Health. 2014;11:1–8.
1302. Abdallah HT, Khalfan FR. Towards Realization of Women’s Sexual and Reproductive Rights in Tanzania: The Case of HIV and AIDS Act of 2008, its compatibility with International Norms and Standards.
1303. Maharaj P, Munthree C. Coerced first sexual intercourse and selected reproductive health outcomes among young women in KwaZulu-Natal, South Africa. J Biosoc Sci. 2007;39(2):231–44.
1304. Bamgboye EA, Ajayi I. Changing patterns of unmet needs for family planning among women of reproductive age in Nigeria. Afr J Reprod Health. 2016;20(3):127–35.
1305. Shallie PD, Haffejee F. Systematic review and meta-analysis assessing the knowledge and use of the female condom among Nigerians. Afr Health Sci. 2021;21(3):1362–74.
1306. Mrabure KO, Omonoseh A. Recognizing the Inalienable Reproductive Health Rights of Women in Nigeria. Customary and Sharia Law as Hindrances: The Way Forward. Commonw Law Rev J. 2022;8:239–53.
1307. Nordensved J, Dahlqvist J. Sex, a one mans show: Perceptions and experience of sexuality, contraceptives, unwanted pregnancy and unsafe abortion among young people in Kisumu, Kenya–A qualitative study. 2011.
1308. Ezechi OC, Kalejaiye OO, Gab-Okafor CV, Oladele DA, Oke BO, Musa ZA, et al. Sero-prevalence and factors associated with Hepatitis B and C co-infection in pregnant Nigerian women living with HIV Infection. Pan Afr Med J. 2014;17(1).
1309. Ashford LS. How HIV and AIDS affect populations. World. 2006;1:38–600.
1310. Bankole A, Moore AM, Singh S, Mirembe F. Unintended Pregnancy And Induced Abortion In Uganda. 2006;
1311. Turan JM, Miller S, Bukusi EA, Sande J, Cohen CR. HIV/AIDS and maternity care in Kenya: how fears of stigma and discrimination affect uptake and provision of labor and delivery services. AIDS Care. 2008;20(8):938–45.
1312. Mbarushimana V, Ntaganira J. Knowledge and attitude to female condom use among undergraduates of Kigali Health Institute. Rwanda J Heal Sci. 2013;2(1):16–25.
1313. Emaway D. Demand for family planning among women voluntary counseling and testing clients: The need for integretion, Dessie town North East Ethiopia. Ethiopia: AAU; 2009.
1314. Hindin MJ, Fatusi AO. Adolescent sexual and reproductive health in developing countries: an overview of trends and interventions. Int Perspect Sex Reprod Health. 2009;35(2):58–62.
1315. Exavery A, Kanté AM, Jackson E, Noronha J, Sikustahili G, Tani K, et al. Role of condom negotiation on condom use among women of reproductive age in three districts in Tanzania. BMC Public Health. 2012;12:1–11.
1316. Kyaw Min M, MCTM PDPH, Surasak Taneepanichskul MD, FRCOGT KC, Somrongthong R, Damrong Reinprayoon MD. The model development of participatory education on adolescent reproductive life (PEARL) programme to prevent unintended pregnancy among Myanmar migrant adolescent and youth in samut sakhon province, Thailand:(situational analysis). 2011;
1317. Awusabo-Asare K, Biddlecom A, Kumi-Kyereme A, Patterson K. Adolescent sexual and reproductive health in Ghana: results from the 2004 National Survey of Adolescents. Occas Rep. 2006;22.
1318. Mtayangulwa R, Kayombo EJ. Knowledge, attitude and use of female condoms among female undergraduate students in University of Dar-Es-Salaam. J Sci Res Reports. 2015;9(2):1–10.
1319. Otieno AJW, Karanja SM, Kagira J. Socio-cultural factors influencing utilization of prevention-of-mother-to child-transmission of HIV strategies among women attending antenatal care clinics in Rachuonyo North Sub-County-Homa-Bay County, Kenya. East Afr Med J. 2017;94(11):946–59.
1320. Hopkins J, Collins L. How linked are national HIV and SRHR strategies? A review of SRHR and HIV strategies in 60 countries. Health Policy Plan. 2017;32(suppl_4):iv57–66.
1321. Owolabi AT, Onayade AA, Ogunlola IO, Ogunniyi SO, Kuti O. Sexual behaviour of secondary school adolescents in Ilesa, Nigeria: implications for the spread of STIs including HIV/AIDS. J Obstet Gynaecol (Lahore). 2005;25(2):174–8.
1322. Akinsolu FT, Adewole IE, Lawale AA, Olagunju MT, Abodunrin OR, Ola OM, et al. HIV and Pregnancy among Adolescents in Sub-Saharan Africa: A Scoping Review. medRxiv. 2024;2004–24.
1323. Sukeri S, Sulaiman Z, Hamid NA, Ibrahim SA. Decision-making on contraceptive use among women living with human immunodeficiency virus in Malaysia: a qualitative inquiry. Korean J Fam Med. 2024;45(1):27.
1324. Abdullahi IS, Chukwudike CO, Sangari JS, Chikwendu JI, Fulani GJ. The causes of unwanted pregnancy and abortion among female students and its impact on their academic performance in FCE Pankshin, Plateau state, Nigeria.
1325. Shaw D. Access to sexual and reproductive health for young people: bridging the disconnect between rights and reality. Int J Gynecol Obstet. 2009;106(2):132–6.
1326. Warren C. Exploring the quality and effect of comprehensive postnatal care models in East and Southern Africa. Ghent University; 2015.
1327. Wilcher R, Hoke T, Adamchak SE, Cates Jr W. Integration of family planning into HIV services: a synthesis of recent evidence. Aids. 2013;27:S65–75.
1328. Timiun GA. Sexual webs model for the examination of unsafe sexual behaviors and the spread of sexually transmitted diseases including HIV/AIDS. Asian Soc Sci. 2012;8(7):119.
1329. Ayalew M, Mengistie B, Semahegn A. Adolescent-parent communication on sexual and reproductive health issues among high school students in Dire Dawa, Eastern Ethiopia: a cross sectional study. Reprod Health. 2014;11:1–8.
1330. Dabis F, Ekpini ER. HIV-1/AIDS and maternal and child health in Africa. Lancet. 2002;359(9323):2097–104.
1331. Li Y, Marshall CM, Rees HC, Nunez A, Ezeanolue EE, Ehiri JE. Intimate partner violence and HIV infection among women: a systematic review and meta‐analysis. African J Reprod Gynaecol Endosc. 2014;17(1).
1332. Lindegren M Lou, Kennedy CE, Bain‐Brickley D, Azman H, Creanga AA, Butler LM, et al. Integration of HIV/AIDS services with maternal, neonatal and child health, nutrition, and family planning services. Cochrane Database Syst Rev. 1996;2012(10).
1333. Bunnell R, Mermin J, De Cock KM. HIV prevention for a threatened continent: implementing positive prevention in Africa. Jama. 2006;296(7):855–8.
1334. Curtis C. Meeting health care needs of women experiencing complications of miscarriage and unsafe abortion: USAID’s postabortion care program. J Midwifery Womens Health. 2007;52(4):368–75.
1335. Ziraba AK, Madise N, Mills S, Kyobutungi C, Ezeh A. Maternal mortality in the informal settlements of Nairobi city: what do we know? Reprod Health. 2009;6:1–8.
1336. Maharaj P. Obstacles to negotiating dual protection: perspectives of men and women. Afr J Reprod Health. 2001;150–61.
1337. Amelya M, Andriyana H, Nababan B, Gunardi ER. Postpartum contraceptive use among HIV positive women in Cipto Mangunkusumo Hospital Jakarta, Indonesia: A cross sectional study. KnE Med. 2016;161–8.
1338. Ensor S, Mechie I, Ryan R, Mussa A, Bame B, Tamuthiba L, et al. Measuring the impact of COVID-19 social distancing measures on sexual health behaviours and access to HIV and sexual and reproductive health services for people living with HIV in Botswana. Front Glob Women’s Heal. 2023;4:981478.
1339. Harrison A, Colvin CJ, Kuo C, Swartz A, Lurie M. Sustained high HIV incidence in young women in Southern Africa: social, behavioral, and structural factors and emerging intervention approaches. Curr HIV/AIDS Rep. 2015;12:207–15.
1340. Kamangu AA, John MR, Nyakoki SJ. Barriers to parent-child communication on sexual and reproductive health issues in East Africa: A review of qualitative research in four countries. J African Stud Dev. 2017;9(4):45–50.
1341. Karim QA, Havlir D, Phanuphak N. Putting women in the centre of the global HIV response is key to achieving epidemic control! African J Reprod Gynaecol Endosc. 2020;23(3):e25473.
1342. Kungu W, Agwanda A, Khasakhala A. Trends and determinants of contraceptive method choice among women aged 15-24 years in Kenya. F1000Research. 2020;9:197.
1343. Bauni EK, Jarabi BO. The low acceptability and use of condoms within marriage: evidence from Nakuru district, Kenya. 2003;
1344. Anguko AA. Determinants of contraceptive use among women of reproductive age in North Eastern Kenya. University of Nairobi; 2014.
1345. Mondal MNI, Shitan M. Factors affecting the HIV/AIDS epidemic: an ecological analysis of global data. Afr Health Sci. 2013;13(2):301–10.
1346. Thurman AR, Clark MR, Doncel GF. Multipurpose prevention technologies: biomedical tools to prevent HIV‐1, HSV‐2, and unintended pregnancies. Infect Dis Obstet Gynecol. 2011;2011(1):429403.
1347. Muturi NW. Communication for HIV/AIDS prevention in Kenya: Social–cultural considerations. J Health Commun. 2005;10(1):77–98.
1348. Haddad LB, Polis CB, Sheth AN, Brown J, Kourtis AP, King C, et al. Contraceptive methods and risk of HIV acquisition or female-to-male transmission. Curr HIV/AIDS Rep. 2014;11:447–58.
1349. Schwartz SR, Papworth E, Ky-Zerbo O, Sithole B, Anato S, Grosso A, et al. Reproductive health needs of female sex workers and opportunities for enhanced prevention of mother-to-child transmission efforts in sub-Saharan Africa. J Fam Plan Reprod Heal Care. 2017;43(1):50–9.
1350. UNION A. SPECIAL SESSION OF THE AFRICAN UNION CONFERENCE OF MINISTERS OF HEALTH MAPUTO, MOZAMBIQUE 18–22 SEPTEMBER 2006 Sp/MIN/CAMH/4a (I).
1351. Kaljee LM, Green M, Riel R, Lerdboon P, Minh TT. Sexual stigma, sexual behaviors, and abstinence among Vietnamese adolescents: implications for risk and protective behaviors for HIV, sexually transmitted infections, and unwanted pregnancy. J Assoc Nurses AIDS Care. 2007;18(2):48–59.
1352. Ajayi OA, Ogunsola OO, Akinro Y, Adamu-Oyegun S, Wudiri K, Ojo TO, et al. Consistent condom use and associated factors among HIV positive women of reproductive age on anti-retroviral treatment in Ogun State, Nigeria. Pan Afr Med J. 2022;43(1).
1353. Akintade OL, Pengpid S, Peltzer K. Awareness and use of and barriers to family planning services among female university students in Lesotho. S Afr J Obstet Gynaecol. 2011;17(3):72–8.
1354. Myer L, Rabkin M, Abrams EJ, Rosenfield A, El-Sadr WM. Focus on women: linking HIV care and treatment with reproductive health services in the MTCT-Plus Initiative. Reprod Health Matters. 2005;13(25):136–46.
1355. Yaya S, Bishwajit G. Age at first sexual intercourse and multiple sexual partnerships among women in Nigeria: A cross-sectional analysis. Front Med. 2018;5:171.
1356. Bana A, Bhat VG, Godlwana X, Libazi S, Maholwana Y, Marafungana N, et al. Knowledge, attitudes and behaviours of adolescents in relation to STIs, pregnancy, contraceptive utilization and substance abuse in the Mhlakulo region, Eastern Cape. South African Fam Pract. 2010;52(2):154–8.
1357. Wilcher R, Petruney T, Cates W. The role of family planning in elimination of new pediatric HIV infection. Curr Opin HIV AIDS. 2013;8(5):490–7.
1358. Ilika CP, Eleje GU, Anyaoku CS, Ikechukwu E, Okaforcha ICA. Emerging determinants of contraceptive practices among HIV-positive women on antiretroviral therapy in Nigeria. 2018;
1359. Gaughran M, Asgary R. On-site comprehensive curriculum to teach reproductive health to female adolescents in Kenya. J women’s Heal. 2014;23(4):358–64.
1360. Spaulding AB, Brickley DB, Kennedy C, Almers L, Packel L, Mirjahangir J, et al. Linking family planning with HIV/AIDS interventions: a systematic review of the evidence. Aids. 2009;23:S79–88.
1361. Wilcher R, Cates Jr W, Gregson S. Family planning and HIV: strange bedfellows no longer. Aids. 2009;23:S1–6.
1362. Amoah EJ, Hinneh T, Aklie R. Determinants and prevalence of modern contraceptive use among sexually active female youth in the Berekum East Municipality, Ghana. PLoS One. 2023;18(6):e0286585.
1363. Yaya S, Odusina EK, Bishwajit G. Prevalence of child marriage and its impact on fertility outcomes in 34 sub-Saharan African countries. BMC Int Health Hum Rights. 2019;19:1–11.
1364. Medley A, Garcia-Moreno C, McGill S, Maman S. Rates, barriers and outcomes of HIV serostatus disclosure among women in developing countries: implications for prevention of mother-to-child transmission programmes. Bull World Health Organ. 2004;82:299–307.
1365. Fetene N, Mekonnen W. The prevalence of risky sexual behaviors among youth center reproductive health clinics users and non-users in Addis Ababa, Ethiopia: a comparative cross-sectional study. PLoS One. 2018;13(6):e0198657.
1366. Orza L, Bewley S, Chung C, Crone ET, Nagadya H, Vazquez M, et al. “Violence. Enough already”: findings from a global participatory survey among women living with HIV. J Int AIDS Soc. 2015;18:20285.
1367. Nwaorgu OC, Onyeneho NG, Okolo M, Obadike E, Enibe G. Reproductive health knowledge and practices among junior secondary school grade one students in Enugu State: threat to achieving millennium development goals in Nigeria. East Afr J Public Health. 2008;5(2):126–31.
1368. De Vogli R, Birbeck GL. Potential impact of adjustment policies on vulnerability of women and children to HIV/AIDS in sub-Saharan Africa. J Heal Popul Nutr. 2005;105–20.
1369. Olagbuji BN. Meeting the contraceptive needs of HIV positive adolescent females living in urban townships in Western Cape, South Africa: perspectives of clients and primary health care providers. 2020;
1370. Kidayi PL, Msuya S, Todd J, Mtuya CC, Mtuy T, Mahande MJ. Determinants of modern contraceptive use among women of reproductive age in Tanzania: evidence from Tanzania demographic and health survey data. Adv Sex Med. 2015;5(03):43–52.
1371. Ojule JD, Oranu EO, Unamba BC. Contraceptive practice among HIV positive women attending anti-retroviral clinic at the University of Port Harcourt Teaching Hospital, Port Harcourt, southern Nigeria. East Afr Med J. 2015;92(1):9–14.
1372. Doyle AM, Mavedzenge SN, Plummer ML, Ross DA. The sexual behaviour of adolescents in sub‐Saharan Africa: patterns and trends from national surveys. Trop Med Int Heal. 2012;17(7):796–807.
1373. Khosla R, Van Belle N, Temmerman M. Advancing the sexual and reproductive health and human rights of women living with HIV: a review of UN, regional and national human rights norms and standards. J Int AIDS Soc. 2015;18:20280.
1374. Leyva-Moral JM, Palmieri PA, Feijoo-Cid M, Cesario SK, Membrillo-Pillpe NJ, Piscoya-Angeles PN, et al. Reproductive decision-making in women living with human immunodeficiency virus: A systematic review. Int J Nurs Stud. 2018;77:207–21.
1375. Harling G, Muya A, Ortblad KF, Mashasi I, Dambach P, Ulenga N, et al. HIV risk and pre-exposure prophylaxis interest among female bar workers in Dar es Salaam: cross-sectional survey. BMJ Open. 2019;9(3):e023272.
1376. Ghanotakis E, Peacock D, Wilcher R. The importance of addressing gender inequality in efforts to end vertical transmission of HIV. J Int AIDS Soc. 2012;15:17385.
1377. Mchunu G, Peltzer K, Tutshana B, Seutlwadi L. Adolescent pregnancy and associated factors in South African youth. Afr Health Sci. 2012;12(4):426–34.
1378. Carter AJ, Bourgeois S, O’Brien N, Abelsohn K, Tharao W, Greene S, et al. Women‐specific HIV/AIDS services: identifying and defining the components of holistic service delivery for women living with HIV/AIDS. African J Reprod Gynaecol Endosc. 2013;16(1).
1379. Shikhansari S, Khalesi ZB, Rad EH. Factors associated with the reproductive health of women living with HIV in Iran. Eur J Obstet Gynecol Reprod Biol X. 2022;13:100136.
1380. Nyanja TAN, Tulinius C. Relationships matter: contraceptive choices among HIV-positive women in Tanzania. African J AIDS Res. 2017;16(2):109–17.
1381. Johnson LF, Mutemaringa T, Heekes A, Boulle A. Effect of HIV infection and antiretroviral treatment on pregnancy rates in the Western Cape province of South Africa. J Infect Dis. 2020;221(12):1953–62.
1382. Nakku‐Joloba E, Pisarski EE, Wyatt MA, Muwonge TR, Asiimwe S, Celum CL, et al. Beyond HIV prevention: everyday life priorities and demand for PrEP among Ugandan HIV serodiscordant couples. African J Reprod Gynaecol Endosc. 2019;22(1).
1383. Maharaj P. The dual risks of unwanted pregnancy and HIV/AIDS: the case of KwaZulu-Natal, South Africa. London School of Hygiene & Tropical Medicine; 2003.
1384. Adilo TM, Wordofa HM. Prevalence of fertility desire and its associated factors among 15-to 49-year-old people living with HIV/AIDS in Addis Ababa, Ethiopia: a cross-sectional study design. HIV/AIDS-Research Palliat Care. 2017;167–76.
1385. Ashimi AO, Amole TG, Abubakar MY, Ugwa EA. Fertility desire and utilization of family planning methods among HIV‑positive women attending a tertiary hospital in a suburban setting in Northern Nigeria. Trop J Obstet Gynaecol. 2017;34(1):54–60.
1386. Selke HM, Kimaiyo S, Sidle JE, Vedanthan R, Tierney WM, Shen C, et al. Task-shifting of antiretroviral delivery from health care workers to persons living with HIV/AIDS: clinical outcomes of a community-based program in Kenya. JAIDS J Acquir Immune Defic Syndr. 2010;55(4):483–90.
1387. Druce N, Nolan A. Seizing the big missed opportunity: linking HIV and maternity care services in sub-Saharan Africa. Reprod Health Matters. 2007;15(30):190–201.
1388. Singh S, Bankole A, Woog V. Evaluating the need for sex education in developing countries: sexual behaviour, knowledge of preventing sexually transmitted infections/HIV and unplanned pregnancy. Sex Educ. 2005;5(4):307–31.
1389. Nakanwagi M, Bulage L, Kwesiga B, Ario AR, Birungi DA, Lukabwe I, et al. Low proportion of women who came knowing their HIV status at first antenatal care visit, Uganda, 2012–2016: a descriptive analysis of surveillance data. BMC Pregnancy Childbirth. 2020;20:1–8.
1390. 52. Kanyangarara M, Sakyi K, Laar A. Availability of integrated family planning services in HIV care and support sites in sub-Saharan Africa: a secondary analysis of national health facility surveys. Reprod Health. 2019;16:1–9.
1391. Bowring AL, Schwartz S, Lyons C, Rao A, Olawore O, Njindam IM, et al. Unmet need for family planning and experience of unintended pregnancy among female sex workers in urban Cameroon: results from a national cross-sectional study. Glob Heal Sci Pract. 2020;8(1):82–99.
1392. Ofurum IC. Sexual Behaviour, Needs and Concerns Regarding Sexual and Reproductive Health among Adults Living with HIV in Sub-Saharan Africa-A Systematic Review. J Adv Med Med Res. 2021;33(11):113–32.
1393. Abay F, Yeshita HY, Mekonnen FA, Sisay M. Dual contraception method utilization and associated factors among sexually active women on antiretroviral therapy in Gondar City, northwest, Ethiopia: a cross sectional study. BMC Womens Health. 2020;20:1–9.
1394. Gelagay AA, Koye DN, Yeshita HY. Demand for long acting contraceptive methods among married HIV positive women attending care at public health facilities at Bahir Dar City, Northwest Ethiopia. Reprod Health. 2015;12:1–9.
1395. Juliastuti D, Dean J, Fitzgerald L. Sexual and reproductive health of women living with HIV in Muslim-majority countries: a systematic mixed studies review. BMC Int Health Hum Rights. 2020;20:1–12.
1396. Manzini N. Sexual initiation and childbearing among adolescent girls in KwaZulu Natal, South Africa. Reprod Health Matters. 2001;9(17):44–52.
1397. Khan MN, Harris ML, Shifti DM, Laar AS, Loxton D. Effects of unintended pregnancy on maternal healthcare services utilization in low-and lower-middle-income countries: systematic review and meta-analysis. Int J Public Health. 2019;64:743–54.
1398. Ochako R, Temmerman M, Mbondo M, Askew I. Determinants of modern contraceptive use among sexually active men in Kenya. Reprod Health. 2017;14:1–15.
1399. Ohnishi M, Leshabari S, Tanaka J, Nishihara M. Factors associated with the awareness of contraceptive methods, understanding the prevention of HIV/AIDS and the perception of HIV/AIDS risk among secondary school students in Dar es Salaam, Tanzania. J Rural Med. 2020;15(4):155–63.
1400. Araya BM, Solomon AA, Gebreslasie KZ, Gudayu TW, Anteneh KT. The role of counseling on modern contraceptive utilization among HIV positive women: the case of Northwest Ethiopia. BMC Womens Health. 2018;18:1–9.
1401. Yam EA, Kidanu A, Burnett‐Zieman B, Pilgrim N, Okal J, Bekele A, et al. Pregnancy experiences of female sex workers in Adama City, Ethiopia: Complexity of partner relationships and pregnancy intentions. Stud Fam Plann. 2017;48(2):107–19.
1402. Ferede TA, Muluneh AG, Wagnew A, Walle AD. Prevalence and associated factors of early sexual initiation among youth female in sub-Saharan Africa: a multilevel analysis of recent demographic and health surveys. BMC Womens Health. 2023;23(1):147.
1403. Bafana TNS. Factors influencing contraceptive use and unplanned pregnancy in a South African population. 2010.
1404. Mbongueh MC, Nicholas T, Ndum AC, Gisèle EL, Nguedia A, Clement J. Unintended pregnancy and sexually transmissible infections amongst adolescents and young adults in Douala IV municipality, Cameroon: Prevalence, knowledge, and associated factors. 2023;
1405. Khu NH, Vwalika B, Karita E, Kilembe W, Bayingana RA, Sitrin D, et al. Fertility goal-based counseling increases contraceptive implant and IUD use in HIV-discordant couples in Rwanda and Zambia. Contraception. 2013;88(1):74–82.
1406. Haile D, Lagebo B. Magnitude of dual contraceptive method utilization and the associated factors among women on antiretroviral treatment in Wolaita zone, Southern Ethiopia. Heliyon. 2022;8(6).
1407. Credé S, Hoke T, Constant D, Green MS, Moodley J, Harries J. Factors impacting knowledge and use of long acting and permanent contraceptive methods by postpartum HIV positive and negative women in Cape Town, South Africa: a cross-sectional study. BMC Public Health. 2012;12:1–9.
1408. Wasie B, Belyhun Y, Moges B, Amare B. Effect of emergency oral contraceptive use on condom utilization and sexual risk taking behaviours among university students, Northwest Ethiopia: a cross-sectional study. BMC Res Notes. 2012;5:1–9.
1409. Feyissa TR, Melka AS. Demand for modern family planning among married women living with HIV in western Ethiopia. PLoS One. 2014;9(11):e113008.
1410. Young IC, Benhabbour SR. Multipurpose prevention technologies: oral, parenteral, and vaginal dosage forms for prevention of HIV/STIs and unplanned pregnancy. Polymers (Basel). 2021;13(15):2450.
1411. Tirado V, Orsini N, Strömdahl S, Hanson C, Ekström AM. Knowledge gaps related to HIV and condom use for preventing pregnancy: a cross-sectional study among migrants in Sweden. BMC Public Health. 2024;24(1):2334.
1412. Amuyunzu-Nyamongo M, Tendo-Wambua L, Babishangire B, Nyagero J, Yitbarek N, Matasha M, et al. Barriers to behaviour change as a response to STD including HIV/AIDS: the East African experience. In Citeseer; 1999.
1413. Chanda P, JO EK, Ochieng LA. FACTORS AFFECTING UPTAKE OF CONTRACEPTIVES AMONG WOMEN AGED 15-25 IN THE CONTEXT OF EARLY PREGNANCY AND HIV/AIDS PREVENTION IN UGANDA.
1414. Dibaba Y, Fantahun M, Hindin MJ. The association of unwanted pregnancy and social support with depressive symptoms in pregnancy: evidence from rural Southwestern Ethiopia. BMC Pregnancy Childbirth. 2013;13:1–8.
1415. Abebe EC, Ayele TM, Dejenie TA, Muche ZT. Assessment of Modern Contraceptive Utilization and Associated Factors Among Women Living With HIV/AIDS In DTRH In Debre Tabor Town, South Gondar Zone, Ethiopia. 2021;
1416. Othieno C, Babigumira JB, Richardson B. Are women with complications of an incomplete abortion more likely to be HIV infected than women without complications? BMC Womens Health. 2015;15:1–9.
1417. Kwame KA, Bain LE, Manu E, Tarkang EE. Use and awareness of emergency contraceptives among women of reproductive age in sub-Saharan Africa: a scoping review. Contracept Reprod Med. 2022;7(1):1.
1418. Reynolds HW, Janowitz B, Homan R, Johnson L. The value of contraception to prevent perinatal HIV transmission. Sex Transm Dis. 2006;33(6):350–6.
1419. Kisaakye P. Contraceptive histories: A comparative analysis of switching behaviour in five East African countries. 2019;
1420. Mamboleo N. Unwanted pregnancy and induced abortion among female youths: a case study of Temeke district. Muhimbili University of Health and Allied Sciences; 2012.
1421. Bastola K. Unintended pregnancy among currently pregnant maried women in Nepal. 2013.
1422. Shehu AU, Joshua IA, Umar Z. Knowledge of contraception and contraceptive choices among human immunodeficiency virus-positive women attending antiretroviral clinics in Zaria, Nigeria. Sub-Saharan African J Med. 2016;3(2):84–90.
1423. Uwamariya J, Nyandwi J, Mukanyangezi M, Kadima J. Sexual activity and emergency contraception among female students in the University of Rwanda. Int J Trop Dis Heal. 2015;8(4):170–7.
1424. Maloiy L, Wawire V. Status and Dynamics of Gender Mainstreaming in East Africa Community COVID-19 Social and Economic Response Policies, Strategies and Interventions. 2021;
1425. Pretorius L, Gibbs A, Crankshaw T, Willan S. Interventions targeting sexual and reproductive health and rights outcomes of young people living with HIV: a comprehensive review of current interventions from sub-Saharan Africa. Glob Health Action. 2015;8(1):28454.
1426. Haddad L, Wall KM, Vwalika B, Khu NH, Brill I, Kilembe W, et al. Contraceptive discontinuation and switching among couples receiving integrated HIV and family planning services in Lusaka, Zambia. Aids. 2013;27:S93–103.
1427. Warren CE, Mayhew SH, Hopkins J. The current status of research on the integration of sexual and reproductive health and HIV services. Stud Fam Plann. 2017;48(2):91–105.
1428. Warren CE, Mayhew SH, Vassall A, Kimani JK, Church K, Obure CD, et al. Study protocol for the Integra Initiative to assess the benefits and costs of integrating sexual and reproductive health and HIV services in Kenya and Swaziland. BMC Public Health. 2012;12:1–16.
1429. Wilcher R, Cates W. Reproductive choices for women with HIV. Bull World Health Organ. 2009;87(11):833–9.
1430. Abdool Karim S, Baxter C, Frohlich J, Abdool Karim Q. The need for multipurpose prevention technologies in sub‐Saharan Africa. BJOG An Int J Obstet Gynaecol. 2014;121:27–34.
1431. Matthews LT, Crankshaw T, Giddy J, Kaida A, Smit JA, Ware NC, et al. Reproductive decision-making and periconception practices among HIV-positive men and women attending HIV services in Durban, South Africa. AIDS Behav. 2013;17:461–70.
1432. Hancock NL, Chibwesha CJ, Bosomprah S, Newman J, Mubiana-Mbewe M, Sitali ES, et al. Contraceptive use among HIV-infected women and men receiving antiretroviral therapy in Lusaka, Zambia: a cross-sectional survey. BMC Public Health. 2016;16:1–8.
1433. Mbirimtengerenji ND. Is HIV/AIDS epidemic outcome of poverty in sub-saharan Africa? Croat Med J. 2007;48(5):605.
1434. Zaidi SS, Ocholla AM, Otieno RA, Sandfort TGM. Women who have sex with women in Kenya and their sexual and reproductive health. LGBT Heal. 2016;3(2):139–45.
1435. Marlow HM, Maman S, Groves AK, Moodley D. Fertility intent and contraceptive decision-making among HIV positive and negative antenatal clinic attendees in Durban, South Africa. Health Care Women Int. 2012;33(4):342–58.
1436. Morrison JS, Fleischman J. Integrating reproductive health and HIV/AIDS programs. Strateg Oppor PEPFAR. 2006;6–7.
1437. Darteh EKM, Abraham SA, Seidu A-A, Chattu VK, Yaya S. Knowledge and determinants of women’s knowledge on vertical transmission of HIV and AIDS in South Africa. AIDS Res Ther. 2021;18:1–9.
1438. Amo-Adjei J, Tuoyire DA. Timing of sexual debut among unmarried youths aged 15–24 years in sub-Saharan Africa. J Biosoc Sci. 2018;50(2):161–77.
1439. Papworth E, Schwartz S, Ky-Zerbo O, Leistman B, Ouedraogo G, Samadoulougou C, et al. Mothers who sell sex: a potential paradigm for integrated HIV, sexual, and reproductive health interventions among women at high risk of HIV in Burkina Faso. JAIDS J Acquir Immune Defic Syndr. 2015;68:S154–61.
1440. Fotso JC, Izugbara C, Saliku T, Ochako R. Unintended pregnancy and subsequent use of modern contraceptive among slum and non-slum women in Nairobi, Kenya. BMC Pregnancy Childbirth. 2014;14:1–10.
1441. Bakari HM, Alo O, Mbwana MS, Salim SM, Ludeman E, Lascko T, et al. Prevalence of unmet need for family planning and unintended pregnancies among women of reproductive age living with HIV in sub-Saharan Africa: a systematic review and meta-analysis. Afr Health Sci. 2024;24(2):41–53.
1442. Mayhew SH, Colombini M, Kimani JK, Tomlin K, Warren CE, Initiative I, et al. Fertility intentions and contraceptive practices among clinic-users living with HIV in Kenya: a mixed methods study. BMC Public Health. 2017;17:1–15.
1443. Atukunda EC, Mugyenyi GR, Atuhumuza EB, Kaida A, Boatin A, Agaba AG, et al. Factors associated with pregnancy intentions amongst postpartum women living with HIV in rural Southwestern Uganda. AIDS Behav. 2019;23:1552–60.
1444. Wall KM, Haddad L, Vwalika B, Htee Khu N, Brill I, Kilembe W, et al. Unintended pregnancy among HIV positive couples receiving integrated HIV counseling, testing, and family planning services in Zambia. PLoS One. 2013;8(9):e75353.
1445. Kimani J, Warren C, Abuya T, Mutemwa R, Initiative I, Mayhew S, et al. Family planning use and fertility desires among women living with HIV in Kenya. BMC Public Health. 2015;15:1–10.
1446. Namukisa M, Kamacooko O, Lunkuse JF, Ruzagira E, Price MA, Mayanja Y. Incidence of unintended pregnancy and associated factors among adolescent girls and young women at risk of HIV infection in Kampala, Uganda. Front Reprod Heal. 2023;5:1089104.
1447. Ikamari L, Izugbara C, Ochako R. Prevalence and determinants of unintended pregnancy among women in Nairobi, Kenya. BMC Pregnancy Childbirth. 2013;13:1–9.
1448. Amongi PR. Factors Associated With Unintended Pregnancy Among Hiv Positive Women On Anti Retroviral Therapy In Gulu District. CIU; 2018.
1449. Feyissa TR, Harris ML, Melka AS, Loxton D. Unintended pregnancy in women living with HIV in Sub-Saharan Africa: a systematic review and meta-analysis. AIDS Behav. 2019;23:1431–51.
1450. Ingabire R, Parker R, Nyombayire J, Ko JE, Mukamuyango J, Bizimana J, et al. Female sex workers in Kigali, Rwanda: a key population at risk of HIV, sexually transmitted infections, and unplanned pregnancy. Int J STD AIDS. 2019;30(6):557–68.
1451. Mumah JN, Mulupi S, Wado YD, Ushie BA, Nai D, Kabiru CW, et al. Adolescents’ narratives of coping with unintended pregnancy in Nairobi’s informal settlements. PLoS One. 2020;15(10):e0240797.
1452. Mumah J, Kabiru CW, Mukiira C, Brinton J, Mutua M, Izugbara CO, et al. Unintended pregnancies in Kenya: a country profile. 2014;
1453. Warren CE, Abuya T, Askew I. Family planning practices and pregnancy intentions among HIV-positive and HIV-negative postpartum women in Swaziland: a cross sectional survey. BMC Pregnancy Childbirth. 2013;13:1–10.
1454. Solanke BL. Do community characteristics influence unintended pregnancies in Kenya? Malawi Med J. 2019;31(1):56–64.
1455. Luchters S, Bosire W, Feng A, Richter ML, King’ola N, Ampt F, et al. “A baby was an added burden”: predictors and consequences of unintended pregnancies for female sex Workers in Mombasa, Kenya: a mixed-methods study. PLoS One. 2016;11(9):e0162871.
1456. 7. Teklu T, Davey G. Which factors influence North Ethiopian adults’ use of dual protection from unintended pregnancy and HIV/AIDS? Ethiop J Heal Dev. 2008;22(3).
1457. 8. Raifman J, Chetty T, Tanser F, Mutevedzi T, Matthews P, Herbst K, et al. Preventing unintended pregnancy and HIV transmission: effects of the HIV treatment cascade on contraceptive use and choice in rural KwaZulu-Natal. JAIDS J Acquir Immune Defic Syndr. 2014;67:S218–27.
1458. 9. Magadi MA. HIV and Unintended Fertility in Sub-Saharan Africa: Multilevel Predictors of Mistimed and Unwanted Fertility Among HIV-Positive Women. Popul Res Policy Rev. 2021;40(5):987–1024.
1459. Mwalye PJ. Impact of Unintended pregnancy on HIV viral load outcomes among postpartum women living with HIV in Cape Town, South Africa: clues from postpartum adherence clubs for antiretroviral therapy trial. 2022;
1460. Harrington EK, Newmann SJ, Onono M, Schwartz KD, Bukusi EA, Cohen CR, et al. Fertility intentions and interest in integrated family planning services among women living with HIV in Nyanza Province, Kenya: a qualitative study. Infect Dis Obstet Gynecol. 2012;2012(1):809682.
1461. Antelman G, Medley A, Mbatia R, Pals S, Arthur G, Haberlen S, et al. Pregnancy desire and dual method contraceptive use among people living with HIV attending clinical care in Kenya, Namibia and Tanzania. J Fam Plan Reprod Heal Care. 2015;41(1):e1–e1.
1462. Heffron R, Thomson K, Celum C, Haberer J, Ngure K, Mugo N, et al. Fertility intentions, pregnancy, and use of PrEP and ART for safer conception among East African HIV serodiscordant couples. AIDS Behav. 2018;22:1758–65.
1463. Adeniyi OV, Ajayi AI, Somefun OD, Lambert JS. Provision of immediate postpartum contraception to women living with HIV in the Eastern Cape, South Africa; a cross-sectional analysis. Reprod Health. 2020;17:1–12.
1464. Patel RC, Amorim G, Jakait B, Shepherd BE, Mocello AR, Musick B, et al. Pregnancies among women living with HIV using contraceptives and antiretroviral therapy in western Kenya: a retrospective, cohort study. BMC Med. 2021;19:1–11.
1465. Bain LE, Zweekhorst MBM, de Cock Buning T. Prevalence and determinants of unintended pregnancy in sub–saharan Africa: a systematic review. Afr J Reprod Health. 2020;24(2):187–205.
1466. Fotso JC, Izugbara C, Saliku T, Ochako R. Unintended pregnancy and subsequent use of modern contraceptive among slum and non-slum women in Nairobi, Kenya. BMC Pregnancy Childbirth. 2014;14:1–10.
1467. Bakari HM, Alo O, Mbwana MS, Salim SM, Ludeman E, Lascko T, et al. Prevalence of unmet need for family planning and unintended pregnancies among women of reproductive age living with HIV in sub-Saharan Africa: a systematic review and meta-analysis. Afr Health Sci. 2024;24(2):41–53.
1468. Njuguna E, Ilovi S, Muiruri P, Mutai K, Kinuthia J, Njoroge P. Factors influencing the utilization of family planning services among HIV infected women in a Kenyan health facility. Int J Reprod Contracept Obs Gynecol. 2017;6(5):1746–52.
1469. Lawani LO, Onyebuchi AK, Iyoke CA. Dual method use for protection of pregnancy and disease prevention among HIV-infected women in South East Nigeria. BMC Womens Health. 2014;14:1–6.
1470. Habte D, Namasasu J. Family planning use among women living with HIV: knowing HIV positive status helps-results from a national survey. Reprod Health. 2015;12:1–11.
1471. Patel RC, Onono M, Gandhi M, Blat C, Hagey J, Shade SB, et al. Pregnancy rates in HIV-positive women using contraceptives and efavirenz-based or nevirapine-based antiretroviral therapy in Kenya: a retrospective cohort study. lancet HIV. 2015;2(11):e474–82.
1472. Lewinsohn R, Crankshaw T, Tomlinson M, Gibbs A, Butler L, Smit J. “This baby came up and then he said,“I give up!”: The interplay between unintended pregnancy, sexual partnership dynamics and social support and the impact on women’s well-being in KwaZulu-Natal, South Africa. Midwifery. 2018;62:29–35.
1473. Ahinkorah BO, Seidu A-A, Appiah F, Oduro JK, Sambah F, Baatiema L, et al. Effect of sexual violence on planned, mistimed and unwanted pregnancies among women of reproductive age in sub-Saharan Africa: A multi-country analysis of Demographic and Health Surveys. SSM-population Heal. 2020;11:100601.
1474. Ochieng Arunda M, Agardh A, Larsson M, Asamoah BO. Survival patterns of neonates born to adolescent mothers and the effect of pregnancy intentions and marital status on newborn survival in Kenya, Uganda, and Tanzania, 2014–2016. Glob Health Action. 2022;15(1):2101731.
1475. Nyanja TAN, Tulinius C. Relationships matter: contraceptive choices among HIV-positive women in Tanzania. African J AIDS Res. 2017;16(2):109–17.
1476. Johnson LF, Mutemaringa T, Heekes A, Boulle A. Effect of HIV infection and antiretroviral treatment on pregnancy rates in the Western Cape province of South Africa. J Infect Dis. 2020;221(12):1953–62.
1477. Nakku‐Joloba E, Pisarski EE, Wyatt MA, Muwonge TR, Asiimwe S, Celum CL, et al. Beyond HIV prevention: everyday life priorities and demand for PrEP among Ugandan HIV serodiscordant couples. African J Reprod Gynaecol Endosc. 2019;22(1).
1478. Maharaj P. The dual risks of unwanted pregnancy and HIV/AIDS: the case of KwaZulu-Natal, South Africa. London School of Hygiene & Tropical Medicine; 2003.
1479. Bradley H, Tsui A, Kidanu A, Gillespie D. HIV infection and contraceptive need among female Ethiopian voluntary HIV counseling and testing clients. AIDS Care. 2010;22(10):1295–304.
1480. Mburu G, Ndimbii J, Ayon S, Mlewa O, Mbizvo M, Kihara C, et al. Contraceptive use among women who inject drugs: motivators, barriers, and unmet needs. Women’s Reprod Heal. 2018;5(2):99–116.
1481. Adilo TM, Wordofa HM. Prevalence of fertility desire and its associated factors among 15-to 49-year-old people living with HIV/AIDS in Addis Ababa, Ethiopia: a cross-sectional study design. HIV/AIDS-Research Palliat Care. 2017;167–76.
1482. Ashimi AO, Amole TG, Abubakar MY, Ugwa EA. Fertility desire and utilization of family planning methods among HIV‑positive women attending a tertiary hospital in a suburban setting in Northern Nigeria. Trop J Obstet Gynaecol. 2017;34(1):54–60.
1483. Selke HM, Kimaiyo S, Sidle JE, Vedanthan R, Tierney WM, Shen C, et al. Task-shifting of antiretroviral delivery from health care workers to persons living with HIV/AIDS: clinical outcomes of a community-based program in Kenya. JAIDS J Acquir Immune Defic Syndr. 2010;55(4):483–90.
1484. Druce N, Nolan A. Seizing the big missed opportunity: linking HIV and maternity care services in sub-Saharan Africa. Reprod Health Matters. 2007;15(30):190–201.
1485. Schaan MM, Taylor M, Gungqisa N, Marlink R. Personal views about womanhood amongst women living with HIV in Botswana. Cult Health Sex. 2016;18(2):171–83.
1486. Singh S, Bankole A, Woog V. Evaluating the need for sex education in developing countries: sexual behaviour, knowledge of preventing sexually transmitted infections/HIV and unplanned pregnancy. Sex Educ. 2005;5(4):307–31.
1487. Myer L, Carter RJ, Katyal M, Toro P, El-Sadr WM, Abrams EJ. Impact of antiretroviral therapy on incidence of pregnancy among HIV-infected women in Sub-Saharan Africa: a cohort study. PLoS Med. 2010;7(2):e1000229.
1488. Nakanwagi M, Bulage L, Kwesiga B, Ario AR, Birungi DA, Lukabwe I, et al. Low proportion of women who came knowing their HIV status at first antenatal care visit, Uganda, 2012–2016: a descriptive analysis of surveillance data. BMC Pregnancy Childbirth. 2020;20:1–8.
1489. Kanyangarara M, Sakyi K, Laar A. Availability of integrated family planning services in HIV care and support sites in sub-Saharan Africa: a secondary analysis of national health facility surveys. Reprod Health. 2019;16:1–9.
1490. Berer M. HIV/AIDS, sexual and reproductive health: intersections and implications for national programmes. Health Policy Plan. 2004;19(suppl_1):i62–70.
1491. Bowring AL, Schwartz S, Lyons C, Rao A, Olawore O, Njindam IM, et al. Unmet need for family planning and experience of unintended pregnancy among female sex workers in urban Cameroon: results from a national cross-sectional study. Glob Heal Sci Pract. 2020;8(1):82–99.
1492. Tibebu NS, Kassie BA, Anteneh TA, Rade BK. Depression, anxiety and stress among HIV-positive pregnant women in Ethiopia during the COVID-19 pandemic. Trans R Soc Trop Med Hyg. 2023;117(5):317–25.
1493. Fotso JC, Izugbara C, Saliku T, Ochako R. Unintended pregnancy and subsequent use of modern contraceptive among slum and non-slum women in Nairobi, Kenya. BMC Pregnancy Childbirth. 2014;14:1–10.
1494. Bakari HM, Alo O, Mbwana MS, Salim SM, Ludeman E, Lascko T, et al. Prevalence of unmet need for family planning and unintended pregnancies among women of reproductive age living with HIV in sub-Saharan Africa: a systematic review and meta-analysis. Afr Health Sci. 2024;24(2):41–53.
1495. Mayhew SH, Colombini M, Kimani JK, Tomlin K, Warren CE, Initiative I, et al. Fertility intentions and contraceptive practices among clinic-users living with HIV in Kenya: a mixed methods study. BMC Public Health. 2017;17:1–15.
1496. Atukunda EC, Mugyenyi GR, Atuhumuza EB, Kaida A, Boatin A, Agaba AG, et al. Factors associated with pregnancy intentions amongst postpartum women living with HIV in rural Southwestern Uganda. AIDS Behav. 2019;23:1552–60.
1497. Wall KM, Haddad L, Vwalika B, Htee Khu N, Brill I, Kilembe W, et al. Unintended pregnancy among HIV positive couples receiving integrated HIV counseling, testing, and family planning services in Zambia. PLoS One. 2013;8(9):e75353.
1498. Kimani J, Warren C, Abuya T, Mutemwa R, Initiative I, Mayhew S, et al. Family planning use and fertility desires among women living with HIV in Kenya. BMC Public Health. 2015;15:1–10.
1499. Namukisa M, Kamacooko O, Lunkuse JF, Ruzagira E, Price MA, Mayanja Y. Incidence of unintended pregnancy and associated factors among adolescent girls and young women at risk of HIV infection in Kampala, Uganda. Front Reprod Heal. 2023;5:1089104.
1500. Amongi PR. Factors Associated With Unintended Pregnancy Among Hiv Positive Women On Anti Retroviral Therapy In Gulu District. CIU; 2018.
1501. Feyissa TR, Harris ML, Melka AS, Loxton D. Unintended pregnancy in women living with HIV in Sub-Saharan Africa: a systematic review and meta-analysis. AIDS Behav. 2019;23:1431–51.
1502. Ingabire R, Parker R, Nyombayire J, Ko JE, Mukamuyango J, Bizimana J, et al. Female sex workers in Kigali, Rwanda: a key population at risk of HIV, sexually transmitted infections, and unplanned pregnancy. Int J STD AIDS. 2019;30(6):557–68.
1503. Mumah JN, Mulupi S, Wado YD, Ushie BA, Nai D, Kabiru CW, et al. Adolescents’ narratives of coping with unintended pregnancy in Nairobi’s informal settlements. PLoS One. 2020;15(10):e0240797.
1504. Mumah J, Kabiru CW, Mukiira C, Brinton J, Mutua M, Izugbara CO, et al. Unintended pregnancies in Kenya: a country profile. 2014;
1505. Warren CE, Abuya T, Askew I. Family planning practices and pregnancy intentions among HIV-positive and HIV-negative postpartum women in Swaziland: a cross sectional survey. BMC Pregnancy Childbirth. 2013;13:1–10.
1506. Solanke BL. Do community characteristics influence unintended pregnancies in Kenya? Malawi Med J. 2019;31(1):56–64.
1507. Luchters S, Bosire W, Feng A, Richter ML, King’ola N, Ampt F, et al. “A baby was an added burden”: predictors and consequences of unintended pregnancies for female sex Workers in Mombasa, Kenya: a mixed-methods study. PLoS One. 2016;11(9):e0162871.
1508. Teklu T, Davey G. Which factors influence North Ethiopian adults’ use of dual protection from unintended pregnancy and HIV/AIDS? Ethiop J Heal Dev. 2008;22(3).
1509. Raifman J, Chetty T, Tanser F, Mutevedzi T, Matthews P, Herbst K, et al. Preventing unintended pregnancy and HIV transmission: effects of the HIV treatment cascade on contraceptive use and choice in rural KwaZulu-Natal. JAIDS J Acquir Immune Defic Syndr. 2014;67:S218–27.
1510. Magadi MA. HIV and Unintended Fertility in Sub-Saharan Africa: Multilevel Predictors of Mistimed and Unwanted Fertility Among HIV-Positive Women. Popul Res Policy Rev. 2021;40(5):987–1024.
1511. Mwalye PJ. Impact of Unintended pregnancy on HIV viral load outcomes among postpartum women living with HIV in Cape Town, South Africa: clues from postpartum adherence clubs for antiretroviral therapy trial. 2022;
1512. Harrington EK, Newmann SJ, Onono M, Schwartz KD, Bukusi EA, Cohen CR, et al. Fertility intentions and interest in integrated family planning services among women living with HIV in Nyanza Province, Kenya: a qualitative study. Infect Dis Obstet Gynecol. 2012;2012(1):809682.
1513. Antelman G, Medley A, Mbatia R, Pals S, Arthur G, Haberlen S, et al. Pregnancy desire and dual method contraceptive use among people living with HIV attending clinical care in Kenya, Namibia and Tanzania. J Fam Plan Reprod Heal Care. 2015;41(1):e1–e1.
1514. Heffron R, Thomson K, Celum C, Haberer J, Ngure K, Mugo N, et al. Fertility intentions, pregnancy, and use of PrEP and ART for safer conception among East African HIV serodiscordant couples. AIDS Behav. 2018;22:1758–65.
1515. Adeniyi OV, Ajayi AI, Somefun OD, Lambert JS. Provision of immediate postpartum contraception to women living with HIV in the Eastern Cape, South Africa; a cross-sectional analysis. Reprod Health. 2020;17:1–12.
1516. Patel RC, Amorim G, Jakait B, Shepherd BE, Mocello AR, Musick B, et al. Pregnancies among women living with HIV using contraceptives and antiretroviral therapy in western Kenya: a retrospective, cohort study. BMC Med. 2021;19:1–11.
1517. Bain LE, Zweekhorst MBM, de Cock Buning T. Prevalence and determinants of unintended pregnancy in sub–saharan Africa: a systematic review. Afr J Reprod Health. 2020;24(2):187–205.
1518. Fotso JC, Izugbara C, Saliku T, Ochako R. Unintended pregnancy and subsequent use of modern contraceptive among slum and non-slum women in Nairobi, Kenya. BMC Pregnancy Childbirth. 2014;14:1–10.
1519. Bakari HM, Alo O, Mbwana MS, Salim SM, Ludeman E, Lascko T, et al. Prevalence of unmet need for family planning and unintended pregnancies among women of reproductive age living with HIV in sub-Saharan Africa: a systematic review and meta-analysis. Afr Health Sci. 2024;24(2):41–53.
1520. Njuguna E, Ilovi S, Muiruri P, Mutai K, Kinuthia J, Njoroge P. Factors influencing the utilization of family planning services among HIV infected women in a Kenyan health facility. Int J Reprod Contracept Obs Gynecol. 2017;6(5):1746–52.
1521. Lawani LO, Onyebuchi AK, Iyoke CA. Dual method use for protection of pregnancy and disease prevention among HIV-infected women in South East Nigeria. BMC Womens Health. 2014;14:1–6.
1522. Habte D, Namasasu J. Family planning use among women living with HIV: knowing HIV positive status helps-results from a national survey. Reprod Health. 2015;12:1–11.
1523. Patel RC, Onono M, Gandhi M, Blat C, Hagey J, Shade SB, et al. Pregnancy rates in HIV-positive women using contraceptives and efavirenz-based or nevirapine-based antiretroviral therapy in Kenya: a retrospective cohort study. lancet HIV. 2015;2(11):e474–82.
1524. Lewinsohn R, Crankshaw T, Tomlinson M, Gibbs A, Butler L, Smit J. “This baby came up and then he said,“I give up!”: The interplay between unintended pregnancy, sexual partnership dynamics and social support and the impact on women’s well-being in KwaZulu-Natal, South Africa. Midwifery. 2018;62:29–35.
1525. Ahinkorah BO, Seidu A-A, Appiah F, Oduro JK, Sambah F, Baatiema L, et al. Effect of sexual violence on planned, mistimed and unwanted pregnancies among women of reproductive age in sub-Saharan Africa: A multi-country analysis of Demographic and Health Surveys. SSM-population Heal. 2020;11:100601.
1526. Ochieng Arunda M, Agardh A, Larsson M, Asamoah BO. Survival patterns of neonates born to adolescent mothers and the effect of pregnancy intentions and marital status on newborn survival in Kenya, Uganda, and Tanzania, 2014–2016. Glob Health Action. 2022;15(1):2101731.
1527. Ma Q, Ono-Kihara M, Cong L, Xu G, Pan X, Zamani S, et al. Unintended pregnancy and its risk factors among university students in eastern China. Contraception. 2008;77(2):108–13.
1528. Mokwena K, Bogale YR. Fertility intention and use of contraception among women living with the human immunodeficiency virus in Oromia Region, Ethiopia. South African Fam Pract. 2017;59(1):46–51.
1529. Bakibinga P, Matanda DJ, Ayiko R, Rujumba J, Muiruri C, Amendah D, et al. Pregnancy history and current use of contraception among women of reproductive age in Burundi, Kenya, Rwanda, Tanzania and Uganda: analysis of demographic and health survey data. BMJ Open. 2016;6(3):e009991.
1530. Okigbo CC, Speizer IS. Determinants of sexual activity and pregnancy among unmarried young women in urban Kenya: a cross-sectional study. PLoS One. 2015;10(6):e0129286.
1531. Ampt FH, Willenberg L, Agius PA, Chersich M, Luchters S, Lim MSC. Incidence of unintended pregnancy among female sex workers in low-income and middle-income countries: a systematic review and meta-analysis. BMJ Open. 2018;8(9):e021779.
1532. Duff P, Muzaaya G, Muldoon K, Dobrer S, Akello M, Birungi J, et al. High rates of unintended pregnancies among young women sex Workers in Conflict-affected Northern Uganda: the social contexts of brothels/lodges and substance use. Afr J Reprod Health. 2017;21(2):64–72.
1533. Ngugi EW, Kim AA, Nyoka R, Mukui I, Ng’eno B, Rutherford GW, et al. Contraceptive practices and fertility desires among HIV-infected and uninfected women in Kenya: results from a nationally representative study. JAIDS J Acquir Immune Defic Syndr. 2014;66:S75–81.
1534. Mubangizi L. Examining the association between future pregnancy intentions, contraceptive use and repeat pregnancies among women living with HIV in Cape Town, South Africa. 2020;
1535. Bankole A, Singh S, Hussain R, Oestreicher G. Condom use for preventing STI/HIV and unintended pregnancy among young men in Sub-Saharan Africa. Am J Mens Health. 2009;3(1):60–78.
1536. Dhakal S, Song JS, Shin DE, Lee TH, So AY, Nam EW. Unintended pregnancy and its correlates among currently pregnant women in the Kwango District, Democratic Republic of the Congo. Reprod Health. 2016;13:1–7.
1537. Izugbara C, Egesa C. The management of unwanted pregnancy among women in Nairobi, Kenya. Int J Sex Heal. 2014;26(2):100–12.
1538. Wall KM, Kilembe W, Vwalika B, Haddad LB, Khu NH, Brill I, et al. Optimizing prevention of HIV and unplanned pregnancy in discordant African couples. J women’s Heal. 2017;26(8):900–10.
1539. Aragaw FM, Amare T, Teklu RE, Tegegne BA, Alem AZ. Magnitude of unintended pregnancy and its determinants among childbearing age women in low and middle-income countries: evidence from 61 low and middle income countries. Front Reprod Heal. 2023;5:1113926.
1540. Chukwunyere AP, Stella KA. Unintended pregnancy among undergraduate students at a select university, Eastern Cape, South Africa: effects, influences, outcomes and solutions. Gend Behav. 2019;17(4):14272–86.
1541. De Bruyn M. Living with HIV: challenges in reproductive health care in South Africa. Afr J Reprod Health. 2004;92–8.
1542. Stuart GS. Fourteen million women with limited options: HIV/AIDS and highly effective reversible contraception in sub-Saharan Africa. Contraception. 2009;80(5):412–6.
1543. Guta NM, Ruksi ST, Senbata GM, Seid K. Predictors of perceived poor social support status of pregnant women attending antiretroviral therapy clinics in south west Ethiopia, 2021. Heliyon. 2023;9(7).
1544. Ayenew A. Women living with HIV and dual contraceptive use in Ethiopia: systematic review and meta-analysis. Contracept Reprod Med. 2022;7(1):11.
1545. Mbuthia CW. Fertility Desires and Contraceptive Practices Among Hiv Positive Adults at Naivasha District Hospital. University of Nairobi; 2010.
1546. Habte D, Teklu S, Melese T, Magafu MGMD. Correlates of unintended pregnancy in Ethiopia: results from a national survey. PLoS One. 2013;8(12):e82987.
1547. Rodriguez MI, Reeves MF, Caughey AB. Evaluating the competing risks of HIV acquisition and maternal mortality in Africa: a decision analysis. BJOG An Int J Obstet Gynaecol. 2012;119(9):1067–73.
1548. Wapmuk AE, Gbajabiamila TA, Ohihoin AG, Ezechi OC. Family Planning In The Context Of HIV Infection. Niger J Clin Biomed Res Wapmuk al. 2017;7(9):6–22.
1549. Bernard C, Jakait B, Fadel WF, Mocello AR, Onono MA, Bukusi EA, et al. Preferences for multipurpose technology and non-oral methods of antiretroviral therapy among women living with HIV in western Kenya: a survey study. Front Glob Women’s Heal. 2022;3:869623.
1550. Tuthill EL, Maltby AE, Odhiambo BC, Akama E, Pellowski JA, Cohen CR, et al. “i found out I was pregnant, and I started feeling stressed”: A longitudinal qualitative perspective of mental health experiences among perinatal women living with hiv. AIDS Behav. 2021;25:4154–68.
1551. Sibanda MY. Factors influencing women living with HIV/AIDS’intention to fall pregnant among those attending the OI/ART clinic in Murambinda, Buhera District, Manicaland Province, Zimbabwe, 2010. 2010;
1552. Kebede HG, Nahusenay H, Birhane Y, Tesfaye DJ. Assessment of contraceptive use and associated factors among HIV positive women in Bahir-Dar Town, Northwest Ethiopia. Open Access Libr J. 2015;2(10):1–19.
1553. Astawesegn FH, Stulz V, Conroy E, Mannan H. Trends and effects of antiretroviral therapy coverage during pregnancy on mother-to-child transmission of HIV in Sub-Saharan Africa. Evidence from panel data analysis. BMC Infect Dis. 2022;22(1):134.
1554. O’Reilly KR, Kennedy CE, Fonner VA, Sweat MD. Family planning counseling for women living with HIV: a systematic review of the evidence of effectiveness on contraceptive uptake and pregnancy incidence, 1990 to 2011. BMC Public Health. 2013;13:1–10.
1555. Bauni EK, Jarabi BO. Family planning and sexual behavior in the era of HIV/AIDS: the case of Nakuru District, Kenya. Wiley Online Library; 2000.
1556. Pokharel R, Bhattarai G, Shrestha N, Onta S. Knowledge and utilization of family planning methods among people living with HIV in Kathmandu, Nepal. BMC Health Serv Res. 2018;18:1–12.
1557. Kisaakye VK. The Effectiveness of Sexual and Reproductive Health Counselling Services for HIV Positive Women in Comprehensive Care Centres in Langata, Kenya. COHES-JKUAT; 2018.
1558. De Paoli MM, Manongi R, Klepp K-I. Factors influencing acceptability of voluntary counselling and HIV-testing among pregnant women in Northern Tanzania. AIDS Care. 2004;16(4):411–25.
1559. Long JE, Waruguru G, Yuhas K, Wilson KS, Masese LN, Wanje G, et al. Prevalence and predictors of unmet contraceptive need in HIV-positive female sex workers in Mombasa, Kenya. PLoS One. 2019;14(6):e0218291.
1560. Melaku YA, Zeleke EG. Contraceptive utilization and associated factors among HIV positive women on chronic follow up care in Tigray Region, Northern Ethiopia: a cross sectional study. PLoS One. 2014;9(4):e94682.
1561. Bergsjø P, Vangen S, Lie RT, Lyatuu R, LIE‐NIELSEN E, Oneko O. Recording of maternal deaths in an East African university hospital. Acta Obstet Gynecol Scand. 2010;89(6):789–93.
1562. Cohen S. Hiding in plain sight: the role of contraception in preventing HIV. Guttmacher Policy Rev. 2008;11(1):2–5.
1563. Richter DL, Sowell RL, Pluto DM. Factors affecting reproductive decisions of African American women living with HIV. Women Health. 2002;36(1):81–96.
1564. Vifeme M, Gwendoline N, Ernest B, Mboh E, Nshom E, Marie TP. Pregnancy and Associated Factors Among Adolescents and Young Adults Living with HIV in the Northwest Region of Cameroon. J Womens Heal Dev. 2022;5(3):221–33.
1565. O’Shea MS, Rosenberg NE, Hosseinipour MC, Stuart GS, Miller WC, Kaliti SM, et al. Effect of HIV status on fertility desire and knowledge of long-acting reversible contraception of postpartum Malawian women. AIDS Care. 2015;27(4):489–98.
1566. Nakaie N, Tuon S, Nozaki I, Yamaguchi F, Sasaki Y, Kakimoto K. Family planning practice and predictors of risk of inconsistent condom use among HIV-positive women on anti-retroviral therapy in Cambodia. BMC Public Health. 2014;14:1–9.
1567. Lunani LL, Abaasa A, Omosa-Manyonyi G. Prevalence and factors associated with contraceptive use among Kenyan women aged 15–49 years. AIDS Behav. 2018;22:125–30.
1568. Wilcher R, Cates W. Reaching the underserved: family planning for women with HIV. Stud Fam Plann. 2010;41(2):125–8.
1569. Colombini M, Mayhew SH, Mutemwa R, Kivunaga J, Ndwiga C, Team I. Perceptions and experiences of integrated service delivery among women living with HIV attending reproductive health services in Kenya: a mixed methods study. AIDS Behav. 2016;20:2130–40.
1570. Skerritt L, Kaida A, O’Brien N, Burchell AN, Bartlett G, Savoie É, et al. Patterns of changing pregnancy intentions among women living with HIV in Canada. BMC Womens Health. 2021;21:1–12.
1571. Magadi MA, Agwanda AO. Determinants of transitions to first sexual intercourse, marriage and pregnancy among female adolescents: evidence from South Nyanza, Kenya. J Biosoc Sci. 2009;41(3):409–27.
1572. Doherty K, Arena K, Wynn A, Offorjebe OA, Moshashane N, Sickboy O, et al. Unintended pregnancy in Gaborone, Botswana: A cross sectional study. Afr J Reprod Health. 2018;22(2):76–82.
1573. Omollo C. Determinants of contraceptives preference and use among people living with hiv and aids in rural areas: a study of Nyamarambe division, Kisii county, Kenya. 2021.
1574. Kebede YB, Geremew TT, Mehretie Y, Abejie AN, Bewket L, Dellie E. Associated factors of modern contraceptive use among women infected with human immunodeficiency virus in Enemay District, Northwest Ethiopia: a facility-based cross-sectional study. BMC Public Health. 2019;19:1–11.
1575. Tumusiigirwe K. Factors associated with unwanted pregnancies among girls aged 15 to 19 years in Kakoba Division in Mbarara District. 2017;
1576. Kamangu AA, Myeya HE. Exploring Young Peoples’ Sexual Behaviours and the Underlying Factors in East Africa: A Review of Literature from Four Countries. J Anthropol Surv India. 2023;72(1):149–62.
1577. Zewdu LB, Reta MM, Yigzaw N, Tamirat KS. Prevalence of suicidal ideation and associated factors among HIV positive perinatal women on follow-up at Gondar town health institutions, Northwest Ethiopia: a cross-sectional study. BMC Pregnancy Childbirth. 2021;21:1–9.
1578. De Bruyn M. Women, reproductive rights, and HIV/AIDS: Issues on which research and interventions are still needed. J Health Popul Nutr. 2006;24(4):413.
1579. Arega T. HIV and Unintended Pregnancy Risk Perception and Contraceptive Use among Youth in Debre Birhan District, Ethiopia. Addis Ababa University; 2010.
1580. Abubeker FA, Fanta MB, Dalton VK. Unmet Need for Contraception among HIV‐Positive Women Attending HIV Care and Treatment Service at Saint Paul’s Hospital Millennium Medical College, Addis Ababa, Ethiopia. Int J Reprod Med. 2019;2019(1):3276780.
1581. Ma Q, Ono-Kihara M, Cong L, Xu G, Pan X, Zamani S, et al. Early initiation of sexual activity: a risk factor for sexually transmitted diseases, HIV infection, and unwanted pregnancy among university students in China. BMC Public Health. 2009;9:1–8.
1582. Shikhansari S, Khalesi ZB, Rad EH. Factors associated with the reproductive health of women living with HIV in Iran. Eur J Obstet Gynecol Reprod Biol X. 2022;13:100136.
1583. Nyanja TAN, Tulinius C. Relationships matter: contraceptive choices among HIV-positive women in Tanzania. African J AIDS Res. 2017;16(2):109–17.
1584. Johnson LF, Mutemaringa T, Heekes A, Boulle A. Effect of HIV infection and antiretroviral treatment on pregnancy rates in the Western Cape province of South Africa. J Infect Dis. 2020;221(12):1953–62.
1585. Nakku‐Joloba E, Pisarski EE, Wyatt MA, Muwonge TR, Asiimwe S, Celum CL, et al. Beyond HIV prevention: everyday life priorities and demand for PrEP among Ugandan HIV serodiscordant couples. African J Reprod Gynaecol Endosc. 2019;22(1).
1586. Maharaj P. The dual risks of unwanted pregnancy and HIV/AIDS: the case of KwaZulu-Natal, South Africa. London School of Hygiene & Tropical Medicine; 2003.
1587. Adilo TM, Wordofa HM. Prevalence of fertility desire and its associated factors among 15-to 49-year-old people living with HIV/AIDS in Addis Ababa, Ethiopia: a cross-sectional study design. HIV/AIDS-Research Palliat Care. 2017;167–76.
1588. Ashimi AO, Amole TG, Abubakar MY, Ugwa EA. Fertility desire and utilization of family planning methods among HIV‑positive women attending a tertiary hospital in a suburban setting in Northern Nigeria. Trop J Obstet Gynaecol. 2017;34(1):54–60.
1589. Selke HM, Kimaiyo S, Sidle JE, Vedanthan R, Tierney WM, Shen C, et al. Task-shifting of antiretroviral delivery from health care workers to persons living with HIV/AIDS: clinical outcomes of a community-based program in Kenya. JAIDS J Acquir Immune Defic Syndr. 2010;55(4):483–90.
1590. Druce N, Nolan A. Seizing the big missed opportunity: linking HIV and maternity care services in sub-Saharan Africa. Reprod Health Matters. 2007;15(30):190–201.
1591. Singh S, Bankole A, Woog V. Evaluating the need for sex education in developing countries: sexual behaviour, knowledge of preventing sexually transmitted infections/HIV and unplanned pregnancy. Sex Educ. 2005;5(4):307–31.
1592. Nakanwagi M, Bulage L, Kwesiga B, Ario AR, Birungi DA, Lukabwe I, et al. Low proportion of women who came knowing their HIV status at first antenatal care visit, Uganda, 2012–2016: a descriptive analysis of surveillance data. BMC Pregnancy Childbirth. 2020;20:1–8.
1593. Kanyangarara M, Sakyi K, Laar A. Availability of integrated family planning services in HIV care and support sites in sub-Saharan Africa: a secondary analysis of national health facility surveys. Reprod Health. 2019;16:1–9.
1594. Bowring AL, Schwartz S, Lyons C, Rao A, Olawore O, Njindam IM, et al. Unmet need for family planning and experience of unintended pregnancy among female sex workers in urban Cameroon: results from a national cross-sectional study. Glob Heal Sci Pract. 2020;8(1):82–99.
1595. Ofurum IC. Sexual Behaviour, Needs and Concerns Regarding Sexual and Reproductive Health among Adults Living with HIV in Sub-Saharan Africa-A Systematic Review. J Adv Med Med Res. 2021;33(11):113–32.
1596. Abay F, Yeshita HY, Mekonnen FA, Sisay M. Dual contraception method utilization and associated factors among sexually active women on antiretroviral therapy in Gondar City, northwest, Ethiopia: a cross sectional study. BMC Womens Health. 2020;20:1–9.
1597. Gelagay AA, Koye DN, Yeshita HY. Demand for long acting contraceptive methods among married HIV positive women attending care at public health facilities at Bahir Dar City, Northwest Ethiopia. Reprod Health. 2015;12:1–9.
1598. Juliastuti D, Dean J, Fitzgerald L. Sexual and reproductive health of women living with HIV in Muslim-majority countries: a systematic mixed studies review. BMC Int Health Hum Rights. 2020;20:1–12.
1599. Stuart GS. Fourteen million women with limited options: HIV/AIDS and highly effective reversible contraception in sub-Saharan Africa. Contraception. 2009;80(5):412–6.
1600. Guta NM, Ruksi ST, Senbata GM, Seid K. Predictors of perceived poor social support status of pregnant women attending antiretroviral therapy clinics in south west Ethiopia, 2021. Heliyon. 2023;9(7).
1601. Ayenew A. Women living with HIV and dual contraceptive use in Ethiopia: systematic review and meta-analysis. Contracept Reprod Med. 2022;7(1):11.
1602. Mbuthia CW. Fertility Desires and Contraceptive Practices Among Hiv Positive Adults at Naivasha District Hospital. University of Nairobi; 2010.
1603. Habte D, Teklu S, Melese T, Magafu MGMD. Correlates of unintended pregnancy in Ethiopia: results from a national survey. PLoS One. 2013;8(12):e82987.
1604. Rodriguez MI, Reeves MF, Caughey AB. Evaluating the competing risks of HIV acquisition and maternal mortality in Africa: a decision analysis. BJOG An Int J Obstet Gynaecol. 2012;119(9):1067–73.
1605. Wapmuk AE, Gbajabiamila TA, Ohihoin AG, Ezechi OC. Family Planning In The Context Of HIV Infection. Niger J Clin Biomed Res Wapmuk al. 2017;7(9):6–22.
1606. Bernard C, Jakait B, Fadel WF, Mocello AR, Onono MA, Bukusi EA, et al. Preferences for multipurpose technology and non-oral methods of antiretroviral therapy among women living with HIV in western Kenya: a survey study. Front Glob Women’s Heal. 2022;3:869623.
1607. Tuthill EL, Maltby AE, Odhiambo BC, Akama E, Pellowski JA, Cohen CR, et al. “i found out I was pregnant, and I started feeling stressed”: A longitudinal qualitative perspective of mental health experiences among perinatal women living with hiv. AIDS Behav. 2021;25:4154–68.
1608. Sibanda MY. Factors influencing women living with HIV/AIDS’intention to fall pregnant among those attending the OI/ART clinic in Murambinda, Buhera District, Manicaland Province, Zimbabwe, 2010. 2010;
1609. Kebede HG, Nahusenay H, Birhane Y, Tesfaye DJ. Assessment of contraceptive use and associated factors among HIV positive women in Bahir-Dar Town, Northwest Ethiopia. Open Access Libr J. 2015;2(10):1–19.
1610. Astawesegn FH, Stulz V, Conroy E, Mannan H. Trends and effects of antiretroviral therapy coverage during pregnancy on mother-to-child transmission of HIV in Sub-Saharan Africa. Evidence from panel data analysis. BMC Infect Dis. 2022;22(1):134.
1611. O’Reilly KR, Kennedy CE, Fonner VA, Sweat MD. Family planning counseling for women living with HIV: a systematic review of the evidence of effectiveness on contraceptive uptake and pregnancy incidence, 1990 to 2011. BMC Public Health. 2013;13:1–10.
1612. Bauni EK, Jarabi BO. Family planning and sexual behavior in the era of HIV/AIDS: the case of Nakuru District, Kenya. Wiley Online Library; 2000.
1613. Pokharel R, Bhattarai G, Shrestha N, Onta S. Knowledge and utilization of family planning methods among people living with HIV in Kathmandu, Nepal. BMC Health Serv Res. 2018;18:1–12.
1614. Kisaakye VK. The Effectiveness of Sexual and Reproductive Health Counselling Services for HIV Positive Women in Comprehensive Care Centres in Langata, Kenya. COHES-JKUAT; 2018.
1615. De Paoli MM, Manongi R, Klepp K-I. Factors influencing acceptability of voluntary counselling and HIV-testing among pregnant women in Northern Tanzania. AIDS Care. 2004;16(4):411–25.
1616. Long JE, Waruguru G, Yuhas K, Wilson KS, Masese LN, Wanje G, et al. Prevalence and predictors of unmet contraceptive need in HIV-positive female sex worker in Mombasa, Kenya. PLoS One. 2019;14(6):e0218291.
1617. Melaku YA, Zeleke EG. Contraceptive utilization and associated factors among HIV positive women on chronic follow up care in Tigray Region, Northern Ethiopia: a cross sectional study. PLoS One. 2014;9(4):e94682.
1618. Bergsjø P, Vangen S, Lie RT, Lyatuu R, LIE‐NIELSEN E, Oneko O. Recording of maternal deaths in an East African university hospital. Acta Obstet Gynecol Scand. 2010;89(6):789–93.
1619. Cohen S. Hiding in plain sight: the role of contraception in preventing HIV. Guttmacher Policy Rev. 2008;11(1):2–5.
1620. Richter DL, Sowell RL, Pluto DM. Factors affecting reproductive decisions of African American women living with HIV. Women Health. 2002;36(1):81–96.
1621. Vifeme M, Gwendoline N, Ernest B, Mboh E, Nshom E, Marie TP. Pregnancy and Associated Factors Among Adolescents and Young Adults Living with HIV in the Northwest Region of Cameroon. J Womens Heal Dev. 2022;5(3):221–33.
1622. O’Shea MS, Rosenberg NE, Hosseinipour MC, Stuart GS, Miller WC, Kaliti SM, et al. Effect of HIV status on fertility desire and knowledge of long-acting reversible contraception of postpartum Malawian women. AIDS Care. 2015;27(4):489–98.
1623. Nakaie N, Tuon S, Nozaki I, Yamaguchi F, Sasaki Y, Kakimoto K. Family planning practice and predictors of risk of inconsistent condom use among HIV-positive women on anti-retroviral therapy in Cambodia. BMC Public Health. 2014;14:1–9.
1624. Lunani LL, Abaasa A, Omosa-Manyonyi G. Prevalence and factors associated with contraceptive use among Kenyan women aged 15–49 years. AIDS Behav. 2018;22:125–30.
1625. Wilcher R, Cates W. Reaching the underserved: family planning for women with HIV. Stud Fam Plann. 2010;41(2):125–8.
1626. Colombini M, Mayhew SH, Mutemwa R, Kivunaga J, Ndwiga C, Team I. Perceptions and experiences of integrated service delivery among women living with HIV attending reproductive health services in Kenya: a mixed methods study. AIDS Behav. 2016;20:2130–40.
1627. Skerritt L, Kaida A, O’Brien N, Burchell AN, Bartlett G, Savoie É, et al. Patterns of changing pregnancy intentions among women living with HIV in Canada. BMC Womens Health. 2021;21:1–12.
1628. Magadi MA, Agwanda AO. Determinants of transitions to first sexual intercourse, marriage and pregnancy among female adolescents: evidence from South Nyanza, Kenya. J Biosoc Sci. 2009;41(3):409–27.
1629. Doherty K, Arena K, Wynn A, Offorjebe OA, Moshashane N, Sickboy O, et al. Unintended pregnancy in Gaborone, Botswana: A cross sectional study. Afr J Reprod Health. 2018;22(2):76–82.
1630. Omollo C. Determinants of contraceptives preference and use among people living with hiv and aids in rural areas: a study of Nyamarambe division, Kisii county, Kenya. 2021.
1631. Kebede YB, Geremew TT, Mehretie Y, Abejie AN, Bewket L, Dellie E. Associated factors of modern contraceptive use among women infected with human immunodeficiency virus in Enemay District, Northwest Ethiopia: a facility-based cross-sectional study. BMC Public Health. 2019;19:1–11.
1632. Tumusiigirwe K. Factors associated with unwanted pregnancies among girls aged 15 to 19 years in Kakoba Division in Mbarara District. 2017;
1633. Kamangu AA, Myeya HE. Exploring Young Peoples’ Sexual Behaviours and the Underlying Factors in East Africa: A Review of Literature from Four Countries. J Anthropol Surv India. 2023;72(1):149–62.
1634. Zewdu LB, Reta MM, Yigzaw N, Tamirat KS. Prevalence of suicidal ideation and associated factors among HIV positive perinatal women on follow-up at Gondar town health institutions, Northwest Ethiopia: a cross-sectional study. BMC Pregnancy Childbirth. 2021;21:1–9.
1635. De Bruyn M. Women, reproductive rights, and HIV/AIDS: Issues on which research and interventions are still needed. J Health Popul Nutr. 2006;24(4):413.
1636. Arega T. HIV and Unintended Pregnancy Risk Perception and Contraceptive Use among Youth in Debre Birhan District, Ethiopia. Addis Ababa University; 2010.
1637. Abubeker FA, Fanta MB, Dalton VK. Unmet Need for Contraception among HIV‐Positive Women Attending HIV Care and Treatment Service at Saint Paul’s Hospital Millennium Medical College, Addis Ababa, Ethiopia. Int J Reprod Med. 2019;2019(1):3276780.
1638. Ma Q, Ono-Kihara M, Cong L, Xu G, Pan X, Zamani S, et al. Early initiation of sexual activity: a risk factor for sexually transmitted diseases, HIV infection, and unwanted pregnancy among university students in China. BMC Public Health. 2009;9:1–8.
1639. Shikhansari S, Khalesi ZB, Rad EH. Factors associated with the reproductive health of women living with HIV in Iran. Eur J Obstet Gynecol Reprod Biol X. 2022;13:100136.
1640. Nyanja TAN, Tulinius C. Relationships matter: contraceptive choices among HIV-positive women in Tanzania. African J AIDS Res. 2017;16(2):109–17.
1641. Johnson LF, Mutemaringa T, Heekes A, Boulle A. Effect of HIV infection and antiretroviral treatment on pregnancy rates in the Western Cape province of South Africa. J Infect Dis. 2020;221(12):1953–62.
1642. Nakku‐Joloba E, Pisarski EE, Wyatt MA, Muwonge TR, Asiimwe S, Celum CL, et al. Beyond HIV prevention: everyday life priorities and demand for PrEP among Ugandan HIV serodiscordant couples. African J Reprod Gynaecol Endosc. 2019;22(1).
1643. Maharaj P. The dual risks of unwanted pregnancy and HIV/AIDS: the case of KwaZulu-Natal, South Africa. London School of Hygiene & Tropical Medicine; 2003.
1644. Adilo TM, Wordofa HM. Prevalence of fertility desire and its associated factors among 15-to 49-year-old people living with HIV/AIDS in Addis Ababa, Ethiopia: a cross-sectional study design. HIV/AIDS-Research Palliat Care. 2017;167–76.
1645. Ashimi AO, Amole TG, Abubakar MY, Ugwa EA. Fertility desire and utilization of family planning methods among HIV‑positive women attending a tertiary hospital in a suburban setting in Northern Nigeria. Trop J Obstet Gynaecol. 2017;34(1):54–60.
1646. Selke HM, Kimaiyo S, Sidle JE, Vedanthan R, Tierney WM, Shen C, et al. Task-shifting of antiretroviral delivery from health care workers to persons living with HIV/AIDS: clinical outcomes of a community-based program in Kenya. JAIDS J Acquir Immune Defic Syndr. 2010;55(4):483–90.
1647. Druce N, Nolan A. Seizing the big missed opportunity: linking HIV and maternity care services in sub-Saharan Africa. Reprod Health Matters. 2007;15(30):190–201.
1648. Singh S, Bankole A, Woog V. Evaluating the need for sex education in developing countries: sexual behaviour, knowledge of preventing sexually transmitted infections/HIV and unplanned pregnancy. Sex Educ. 2005;5(4):307–31.
1649. Nakanwagi M, Bulage L, Kwesiga B, Ario AR, Birungi DA, Lukabwe I, et al. Low proportion of women who came knowing their HIV status at first antenatal care visit, Uganda, 2012–2016: a descriptive analysis of surveillance data. BMC Pregnancy Childbirth. 2020;20:1–8.
1650. 52. Kanyangarara M, Sakyi K, Laar A. Availability of integrated family planning services in HIV care and support sites in sub-Saharan Africa: a secondary analysis of national health facility surveys. Reprod Health. 2019;16:1–9.
1651. Bowring AL, Schwartz S, Lyons C, Rao A, Olawore O, Njindam IM, et al. Unmet need for family planning and experience of unintended pregnancy among female sex workers in urban Cameroon: results from a national cross-sectional study. Glob Heal Sci Pract. 2020;8(1):82–99.
1652. Ofurum IC. Sexual Behaviour, Needs and Concerns Regarding Sexual and Reproductive Health among Adults Living with HIV in Sub-Saharan Africa-A Systematic Review. J Adv Med Med Res. 2021;33(11):113–32.
1653. Abay F, Yeshita HY, Mekonnen FA, Sisay M. Dual contraception method utilization and associated factors among sexually active women on antiretroviral therapy in Gondar City, northwest, Ethiopia: a cross sectional study. BMC Womens Health. 2020;20:1–9.
1654. Gelagay AA, Koye DN, Yeshita HY. Demand for long acting contraceptive methods among married HIV positive women attending care at public health facilities at Bahir Dar City, Northwest Ethiopia. Reprod Health. 2015;12:1–9.
1655. Juliastuti D, Dean J, Fitzgerald L. Sexual and reproductive health of women living with HIV in Muslim-majority countries: a systematic mixed studies review. BMC Int Health Hum Rights. 2020;20:1–12.
1656. Manzini N. Sexual initiation and childbearing among adolescent girls in KwaZulu Natal, South Africa. Reprod Health Matters. 2001;9(17):44–52.
1657. Khan MN, Harris ML, Shifti DM, Laar AS, Loxton D. Effects of unintended pregnancy on maternal healthcare services utilization in low-and lower-middle-income countries: systematic review and meta-analysis. Int J Public Health. 2019;64:743–54.
1658. Ochako R, Temmerman M, Mbondo M, Askew I. Determinants of modern contraceptive use among sexually active men in Kenya. Reprod Health. 2017;14:1–15.
1659. Ohnishi M, Leshabari S, Tanaka J, Nishihara M. Factors associated with the awareness of contraceptive methods, understanding the prevention of HIV/AIDS and the perception of HIV/AIDS risk among secondary school students in Dar es Salaam, Tanzania. J Rural Med. 2020;15(4):155–63.
1660. Araya BM, Solomon AA, Gebreslasie KZ, Gudayu TW, Anteneh KT. The role of counseling on modern contraceptive utilization among HIV positive women: the case of Northwest Ethiopia. BMC Womens Health. 2018;18:1–9.
1661. Yam EA, Kidanu A, Burnett‐Zieman B, Pilgrim N, Okal J, Bekele A, et al. Pregnancy experiences of female sex workers in Adama City, Ethiopia: Complexity of partner relationships and pregnancy intentions. Stud Fam Plann. 2017;48(2):107–19.
1662. Ferede TA, Muluneh AG, Wagnew A, Walle AD. Prevalence and associated factors of early sexual initiation among youth female in sub-Saharan Africa: a multilevel analysis of recent demographic and health surveys. BMC Womens Health. 2023;23(1):147.
1663. Bafana TNS. Factors influencing contraceptive use and unplanned pregnancy in a South African population. 2010.
1664. Mbongueh MC, Nicholas T, Ndum AC, Gisèle EL, Nguedia A, Clement J. Unintended pregnancy and sexually transmissible infections amongst adolescents and young adults in Douala IV municipality, Cameroon: Prevalence, knowledge, and associated factors. 2023;
1665. Khu NH, Vwalika B, Karita E, Kilembe W, Bayingana RA, Sitrin D, et al. Fertility goal-based counseling increases contraceptive implant and IUD use in HIV-discordant couples in Rwanda and Zambia. Contraception. 2013;88(1):74–82.
1666. Haile D, Lagebo B. Magnitude of dual contraceptive method utilization and the associated factors among women on antiretroviral treatment in Wolaita zone, Southern Ethiopia. Heliyon. 2022;8(6).
1667. Credé S, Hoke T, Constant D, Green MS, Moodley J, Harries J. Factors impacting knowledge and use of long acting and permanent contraceptive methods by postpartum HIV positive and negative women in Cape Town, South Africa: a cross-sectional study. BMC Public Health. 2012;12:1–9.
1668. Wasie B, Belyhun Y, Moges B, Amare B. Effect of emergency oral contraceptive use on condom utilization and sexual risk taking behaviours among university students, Northwest Ethiopia: a cross-sectional study. BMC Res Notes. 2012;5:1–9.
1669. Feyissa TR, Melka AS. Demand for modern family planning among married women living with HIV in western Ethiopia. PLoS One. 2014;9(11):e113008.
1670. Young IC, Benhabbour SR. Multipurpose prevention technologies: oral, parenteral, and vaginal dosage forms for prevention of HIV/STIs and unplanned pregnancy. Polymers (Basel). 2021;13(15):2450.
1671. Tirado V, Orsini N, Strömdahl S, Hanson C, Ekström AM. Knowledge gaps related to HIV and condom use for preventing pregnancy: a cross-sectional study among migrants in Sweden. BMC Public Health. 2024;24(1):2334.
1672. Amuyunzu-Nyamongo M, Tendo-Wambua L, Babishangire B, Nyagero J, Yitbarek N, Matasha M, et al. Barriers to behaviour change as a response to STD including HIV/AIDS: the East African experience. In Citeseer; 1999.
1673. Chanda P, JO EK, Ochieng LA. FACTORS AFFECTING UPTAKE OF CONTRACEPTIVES AMONG WOMEN AGED 15-25 IN THE CONTEXT OF EARLY PREGNANCY AND HIV/AIDS PREVENTION IN UGANDA.
1674. Dibaba Y, Fantahun M, Hindin MJ. The association of unwanted pregnancy and social support with depressive symptoms in pregnancy: evidence from rural Southwestern Ethiopia. BMC Pregnancy Childbirth. 2013;13:1–8.
1675. Abebe EC, Ayele TM, Dejenie TA, Muche ZT. Assessment of Modern Contraceptive Utilization and Associated Factors Among Women Living With HIV/AIDS In DTRH In Debre Tabor Town, South Gondar Zone, Ethiopia. 2021;
1676. Othieno C, Babigumira JB, Richardson B. Are women with complications of an incomplete abortion more likely to be HIV infected than women without complications? BMC Womens Health. 2015;15:1–9.
1677. Kwame KA, Bain LE, Manu E, Tarkang EE. Use and awareness of emergency contraceptives among women of reproductive age in sub-Saharan Africa: a scoping review. Contracept Reprod Med. 2022;7(1):1.
1678. Reynolds HW, Janowitz B, Homan R, Johnson L. The value of contraception to prevent perinatal HIV transmission. Sex Transm Dis. 2006;33(6):350–6.
1679. Kisaakye P. Contraceptive histories: A comparative analysis of switching behaviour in five East African countries. 2019;
1680. Mamboleo N. Unwanted pregnancy and induced abortion among female youths: a case study of Temeke district. Muhimbili University of Health and Allied Sciences; 2012.
1681. Bastola K. Unintended pregnancy among currently pregnant maried women in Nepal. 2013.
1682. Shehu AU, Joshua IA, Umar Z. Knowledge of contraception and contraceptive choices among human immunodeficiency virus-positive women attending antiretroviral clinics in Zaria, Nigeria. Sub-Saharan African J Med. 2016;3(2):84–90.
1683. Uwamariya J, Nyandwi J, Mukanyangezi M, Kadima J. Sexual activity and emergency contraception among female students in the University of Rwanda. Int J Trop Dis Heal. 2015;8(4):170–7.
1684. Maloiy L, Wawire V. Status and Dynamics of Gender Mainstreaming in East Africa Community COVID-19 Social and Economic Response Policies, Strategies and Interventions. 2021;
1685. Pretorius L, Gibbs A, Crankshaw T, Willan S. Interventions targeting sexual and reproductive health and rights outcomes of young people living with HIV: a comprehensive review of current interventions from sub-Saharan Africa. Glob Health Action. 2015;8(1):28454.
1686. Haddad L, Wall KM, Vwalika B, Khu NH, Brill I, Kilembe W, et al. Contraceptive discontinuation and switching among couples receiving integrated HIV and family planning services in Lusaka, Zambia. Aids. 2013;27:S93–103.
1687. Warren CE, Mayhew SH, Hopkins J. The current status of research on the integration of sexual and reproductive health and HIV services. Stud Fam Plann. 2017;48(2):91–105.
1688. Warren CE, Mayhew SH, Vassall A, Kimani JK, Church K, Obure CD, et al. Study protocol for the Integra Initiative to assess the benefits and costs of integrating sexual and reproductive health and HIV services in Kenya and Swaziland. BMC Public Health. 2012;12:1–16.
1689. Wilcher R, Cates W. Reproductive choices for women with HIV. Bull World Health Organ. 2009;87(11):833–9.
1690. Abdool Karim S, Baxter C, Frohlich J, Abdool Karim Q. The need for multipurpose prevention technologies in sub‐Saharan Africa. BJOG An Int J Obstet Gynaecol. 2014;121:27–34.
1691. Matthews LT, Crankshaw T, Giddy J, Kaida A, Smit JA, Ware NC, et al. Reproductive decision-making and periconception practices among HIV-positive men and women attending HIV services in Durban, South Africa. AIDS Behav. 2013;17:461–70.
1692. Hancock NL, Chibwesha CJ, Bosomprah S, Newman J, Mubiana-Mbewe M, Sitali ES, et al. Contraceptive use among HIV-infected women and men receiving antiretroviral therapy in Lusaka, Zambia: a cross-sectional survey. BMC Public Health. 2016;16:1–8.
1693. Mbirimtengerenji ND. Is HIV/AIDS epidemic outcome of poverty in sub-saharan Africa? Croat Med J. 2007;48(5):605.
1694. Zaidi SS, Ocholla AM, Otieno RA, Sandfort TGM. Women who have sex with women in Kenya and their sexual and reproductive health. LGBT Heal. 2016;3(2):139–45.
1695. Marlow HM, Maman S, Groves AK, Moodley D. Fertility intent and contraceptive decision-making among HIV positive and negative antenatal clinic attendees in Durban, South Africa. Health Care Women Int. 2012;33(4):342–58.
1696. Morrison JS, Fleischman J. Integrating reproductive health and HIV/AIDS programs. Strateg Oppor PEPFAR. 2006;6–7.
1697. Darteh EKM, Abraham SA, Seidu A-A, Chattu VK, Yaya S. Knowledge and determinants of women’s knowledge on vertical transmission of HIV and AIDS in South Africa. AIDS Res Ther. 2021;18:1–9.
1698. Amo-Adjei J, Tuoyire DA. Timing of sexual debut among unmarried youths aged 15–24 years in sub-Saharan Africa. J Biosoc Sci. 2018;50(2):161–77.
1699. Papworth E, Schwartz S, Ky-Zerbo O, Leistman B, Ouedraogo G, Samadoulougou C, et al. Mothers who sell sex: a potential paradigm for integrated HIV, sexual, and reproductive health interventions among women at high risk of HIV in Burkina Faso. JAIDS J Acquir Immune Defic Syndr. 2015;68:S154–61.
1700. MacCarthy S, Rasanathan JJK, Ferguson L, Gruskin S. The pregnancy decisions of HIV-positive women: the state of knowledge and way forward. Reprod Health Matters. 2012;20(sup39):119–40.
1701. Polisi A, Gebrehanna E, Tesfaye G, Asefa F. Modern contraceptive utilization among female ART attendees in health facilities of Gimbie town, West Ethiopia. Reprod Health. 2014;11:1–6.
1702. Turan B, Stringer KL, Onono M, Bukusi EA, Weiser SD, Cohen CR, et al. Linkage to HIV care, postpartum depression, and HIV-related stigma in newly diagnosed pregnant women living with HIV in Kenya: a longitudinal observational study. BMC Pregnancy Childbirth. 2014;14:1–10.
1703. Birungi H, Obare F, van der Kwaak A, Namwebya JH. Maternal health care utilization among HIV-positive female adolescents in Kenya. Int Perspect Sex Reprod Health. 2011;143–9.
1704. Patel RC, Morroni C, Scarsi KK, Sripipatana T, Kiarie J, Cohen CR. Concomitant contraceptive implant and efavirenz use in women living with HIV: perspectives on current evidence and policy implications for family planning and HIV treatment guidelines. African J Reprod Gynaecol Endosc. 2017;20(1).
1705. Mba CJ. Sexual Behaviour and The risks of HIV/AIDS and other STDs among young people in sub-Saharan Africa: a REVIEW. Inst African Stud Res Rev. 2003;19(1):15–25.
1706. Todd CS, Stibich MA, Laher F, Malta MS, Bastos FI, Imbuki K, et al. Influence of culture on contraceptive utilization among HIV-positive women in Brazil, Kenya, and South Africa. AIDS Behav. 2011;15:454–68.
1707. Suryavanshi N, Erande A, Pisal H, Shankar A V, Bhosale RA, Bollinger RC, et al. Repeated pregnancy among women with known HIV status in Pune, India. AIDS Care. 2008;20(9):1111–8.
1708. Levandowski BA, Kalilani‐Phiri L, Kachale F, Awah P, Kangaude G, Mhango C. Investigating social consequences of unwanted pregnancy and unsafe abortion in Malawi: the role of stigma. Int J Gynecol Obstet. 2012;118:S167–71.
1709. MONEM AA. UNINTENDED PREGNANCIES IN THE MIDDLE EAST AND NORTH AFRICA. 2010;
1710. Willard Cates JR, Steiner MJ. Dual protection against unintended pregnancy and sexually transmitted infections: what is the best contraceptive approach? Sex Transm Dis. 2002;29(3):168–74.
1711. Amin A. Addressing gender inequalities to improve the sexual and reproductive health and wellbeing of women living with HIV. J Int AIDS Soc. 2015;18:20302.
1712. Ezeh AC, Mberu BU, Emina JO. Stall in fertility decline in Eastern African countries: regional analysis of patterns, determinants and implications. Philos Trans R Soc B Biol Sci. 2009;364(1532):2991–3007.
1713. Hofmeyr GJ, Singata-Madliki M, Lawrie TA, Bergel E, Temmerman M. Effects of the copper intrauterine device versus injectable progestin contraception on pregnancy rates and method discontinuation among women attending termination of pregnancy services in South Africa: a pragmatic randomized controlled trial. Reprod Health. 2016;13:1–8.
1714. Knopf AS, McNealy KR, Al-Khattab H, Carter-Harris L, Oruche UM, Naanyu V, et al. Sexual learning among East African adolescents in the context of generalized HIV epidemics: A systematic qualitative meta-synthesis. PLoS One. 2017;12(3):e0173225.
1715. Lusi O, Ronen K, Larsen AM, Richardson B, Khasimwa B, Chohan B, et al. Antenatal depressive symptoms in Kenyan women living with HIV: contributions of recent HIV diagnosis, stigma, and partner violence. 2022;
1716. Grabbe K, Stephenson R, Vwalika B, Ahmed Y, Vwalika C, Chomba E, et al. Knowledge, use, and concerns about contraceptive methods among sero-discordant couples in Rwanda and Zambia. J women’s Heal. 2009;18(9):1449–56.
1717. Ivanova O, Rai M, Kemigisha E. A systematic review of sexual and reproductive health knowledge, experiences and access to services among refugee, migrant and displaced girls and young women in Africa. Int J Environ Res Public Health. 2018;15(8):1583.
1718. Debela SM, Adinew YM, Geleta LA, Guye AH. Dual Contraceptive Utilization and Associated Factors among Women Attending Antiretroviral Therapy Clinics in Central Ethiopia, 2022: The Need for a Better Control of Ever-Increasing New Strain of HIV Infection and its Transmission. Int J Women’s Heal Care. 2023;8(1):39–49.
1719. Ampt FH, Mudogo C, Gichangi P, Lim MSC, Manguro G, Chersich M, et al. WHISPER or SHOUT study: protocol of a cluster-randomised controlled trial assessing mHealth sexual reproductive health and nutrition interventions among female sex workers in Mombasa, Kenya. BMJ Open. 2017;7(8):e017388.
1720. Huertas-Zurriaga A, Palmieri PA, Aguayo-Gonzalez MP, Dominguez-Cancino KA, Casanovas-Cuellar C, Linden KL Vander, et al. Reproductive decision-making of Black women living with HIV: A systematic review. Women’s Heal. 2022;18:17455057221090828.
1721. Dugg P, Chhabra P, Sharma AK. Contraceptive use and unmet need for family planning among HIV-positive women: a hospital-based study. Indian J Public Health. 2020;64(1):32–8.
1722. Gebrehiwot SW, Azeze GA, Robles CC, Adinew YM. Utilization of dual contraception method among reproductive age women on antiretroviral therapy in selected public hospitals of Northern Ethiopia. Reprod Health. 2017;14:1–9.
1723. Bouris A, Guilamo-Ramos V, Jaccard J, McCoy W, Aranda D, Pickard A, et al. The feasibility of a clinic-based parent intervention to prevent HIV, sexually transmitted infections, and unintended pregnancies among Latino and African American adolescents. AIDS Patient Care STDS. 2010;24(6):381–7.
1724. Mbuthia CW. Fertility Desires and Contraceptive Practices Among Hiv Positive Adults at Naivasha District Hospital. University of Nairobi; 2010.
1725. Sibanda MY. Factors influencing women living with HIV/AIDS’intention to fall pregnant among those attending the OI/ART clinic in Murambinda, Buhera District, Manicaland Province, Zimbabwe, 2010. 2010;
1726. Bauni EK, Jarabi BO. Family planning and sexual behavior in the era of HIV/AIDS: the case of Nakuru District, Kenya. Wiley Online Library; 2000.
1727. Bergsjø P, Vangen S, Lie RT, Lyatuu R, LIE‐NIELSEN E, Oneko O. Recording of maternal deaths in an East African university hospital. Acta Obstet Gynecol Scand. 2010;89(6):789–93.
1728. Richter DL, Sowell RL, Pluto DM. Factors affecting reproductive decisions of African American women living with HIV. Women Health. 2002;36(1):81–96.
1729. Grossman D, Onono M, Newmann SJ, Blat C, Bukusi EA, Shade SB, et al. Integration of family planning services into HIV care and treatment in Kenya: a cluster-randomized trial. Aids. 2013;27:S77–85.
1730. Omollo C. Determinants of contraceptives preference and use among people living with hiv and aids in rural areas: a study of Nyamarambe division, Kisii county, Kenya. 2021.
1731. Bachanas P, Kidder D, Medley A, Pals SL, Carpenter D, Howard A, et al. Delivering prevention interventions to people living with HIV in clinical care settings: results of a cluster randomized trial in Kenya, Namibia, and Tanzania. AIDS Behav. 2016;20:2110–8.
1732. Adilo TM, Wordofa HM. Prevalence of fertility desire and its associated factors among 15-to 49-year-old people living with HIV/AIDS in Addis Ababa, Ethiopia: a cross-sectional study design. HIV/AIDS-Research Palliat Care. 2017;167–76.
1733. Ashimi AO, Amole TG, Abubakar MY, Ugwa EA. Fertility desire and utilization of family planning methods among HIV‑positive women attending a tertiary hospital in a suburban setting in Northern Nigeria. Trop J Obstet Gynaecol. 2017;34(1):54–60.
1734. Singh S, Bankole A, Woog V. Evaluating the need for sex education in developing countries: sexual behaviour, knowledge of preventing sexually transmitted infections/HIV and unplanned pregnancy. Sex Educ. 2005;5(4):307–31.
1735. Kanyangarara M, Sakyi K, Laar A. Availability of integrated family planning services in HIV care and support sites in sub-Saharan Africa: a secondary analysis of national health facility surveys. Reprod Health. 2019;16:1–9.
1736. Juliastuti D, Dean J, Fitzgerald L. Sexual and reproductive health of women living with HIV in Muslim-majority countries: a systematic mixed studies review. BMC Int Health Hum Rights. 2020;20:1–12.
1737. Esplen E. Women and girls living with HIV/AIDS: overview and annotated bibliography. Institute of Development Studies at the University of Sussex Brighton, UK; 2007.
1738. Hagey JM, Akama E, Ayieko J, Bukusi EA, Cohen CR, Patel RC. Barriers and facilitators adolescent females living with HIV face in accessing contraceptive services: a qualitative assessment of providers’ perceptions in western Kenya. African J Reprod Gynaecol Endosc. 2015;18(1).
1739. Adilo TM. Prevalence and determinants of contraceptive utilization among reproductive age women living with HIV/AIDS in Addis Ababa, Ethiopia; a cross sectional study design. EC Gynaecol. 2017;4(3):97–112.
1740. Brandt R. The mental health of people living with HIV/AIDS in Africa: a systematic review. African J AIDS Res. 2009;8(2):123–33.
1741. Juliastuti D, Dean J, Fitzgerald L. Sexual and reproductive health of women living with HIV in Muslim-majority countries: a systematic mixed studies review. BMC Int Health Hum Rights. 2020;20:1–12.
1742. Esplen E. Women and girls living with HIV/AIDS: overview and annotated bibliography. Institute of Development Studies at the University of Sussex Brighton, UK; 2007.
1743. Hagey JM, Akama E, Ayieko J, Bukusi EA, Cohen CR, Patel RC. Barriers and facilitators adolescent females living with HIV face in accessing contraceptive services: a qualitative assessment of providers’ perceptions in western Kenya. African J Reprod Gynaecol Endosc. 2015;18(1).
1744. Adilo TM. Prevalence and determinants of contraceptive utilization among reproductive age women living with HIV/AIDS in Addis Ababa, Ethiopia; a cross sectional study design. EC Gynaecol. 2017;4(3):97–112.
1745. Brandt R. The mental health of people living with HIV/AIDS in Africa: a systematic review. African J AIDS Res. 2009;8(2):123–33.
1746. Mbongueh MC, Nicholas T, Ndum AC, Gisèle EL, Nguedia A, Clement J. Unintended pregnancy and sexually transmissible infections amongst adolescents and young adults in Douala IV municipality, Cameroon: Prevalence, knowledge, and associated factors. 2023;
1747. Khu NH, Vwalika B, Karita E, Kilembe W, Bayingana RA, Sitrin D, et al. Fertility goal-based counseling increases contraceptive implant and IUD use in HIV-discordant couples in Rwanda and Zambia. Contraception. 2013;88(1):74–82.
1748. Haile D, Lagebo B. Magnitude of dual contraceptive method utilization and the associated factors among women on antiretroviral treatment in Wolaita zone, Southern Ethiopia. Heliyon. 2022;8(6).
1749. Credé S, Hoke T, Constant D, Green MS, Moodley J, Harries J. Factors impacting knowledge and use of long acting and permanent contraceptive methods by postpartum HIV positive and negative women in Cape Town, South Africa: a cross-sectional study. BMC Public Health. 2012;12:1–9.
1750. Wasie B, Belyhun Y, Moges B, Amare B. Effect of emergency oral contraceptive use on condom utilization and sexual risk taking behaviours among university students, Northwest Ethiopia: a cross-sectional study. BMC Res Notes. 2012;5:1–9.
1751. Feyissa TR, Melka AS. Demand for modern family planning among married women living with HIV in western Ethiopia. PLoS One. 2014;9(11):e113008.
1752. Young IC, Benhabbour SR. Multipurpose prevention technologies: oral, parenteral, and vaginal dosage forms for prevention of HIV/STIs and unplanned pregnancy. Polymers (Basel). 2021;13(15):2450.
1753. Tirado V, Orsini N, Strömdahl S, Hanson C, Ekström AM. Knowledge gaps related to HIV and condom use for preventing pregnancy: a cross-sectional study among migrants in Sweden. BMC Public Health. 2024;24(1):2334.
1754. Amuyunzu-Nyamongo M, Tendo-Wambua L, Babishangire B, Nyagero J, Yitbarek N, Matasha M, et al. Barriers to behaviour change as a response to STD including HIV/AIDS: the East African experience. In Citeseer; 1999.
1755. Chanda P, JO EK, Ochieng LA. FACTORS AFFECTING UPTAKE OF CONTRACEPTIVES AMONG WOMEN AGED 15-25 IN THE CONTEXT OF EARLY PREGNANCY AND HIV/AIDS PREVENTION IN UGANDA.
1756. Dibaba Y, Fantahun M, Hindin MJ. The association of unwanted pregnancy and social support with depressive symptoms in pregnancy: evidence from rural Southwestern Ethiopia. BMC Pregnancy Childbirth. 2013;13:1–8.
1757. Abebe EC, Ayele TM, Dejenie TA, Muche ZT. Assessment of Modern Contraceptive Utilization and Associated Factors Among Women Living With HIV/AIDS In DTRH In Debre Tabor Town, South Gondar Zone, Ethiopia. 2021;
1758. Othieno C, Babigumira JB, Richardson B. Are women with complications of an incomplete abortion more likely to be HIV infected than women without complications? BMC Womens Health. 2015;15:1–9.
1759. Kwame KA, Bain LE, Manu E, Tarkang EE. Use and awareness of emergency contraceptives among women of reproductive age in sub-Saharan Africa: a scoping review. Contracept Reprod Med. 2022;7(1):1.
1760. Reynolds HW, Janowitz B, Homan R, Johnson L. The value of contraception to prevent perinatal HIV transmission. Sex Transm Dis. 2006;33(6):350–6.
1761. Kisaakye P. Contraceptive histories: A comparative analysis of switching behaviour in five East African countries. 2019;
1762. Mamboleo N. Unwanted pregnancy and induced abortion among female youths: a case study of Temeke district. Muhimbili University of Health and Allied Sciences; 2012.
1763. Bastola K. Unintended pregnancy among currently pregnant maried women in Nepal. 2013.
1764. Shehu AU, Joshua IA, Umar Z. Knowledge of contraception and contraceptive choices among human immunodeficiency virus-positive women attending antiretroviral clinics in Zaria, Nigeria. Sub-Saharan African J Med. 2016;3(2):84–90.
1765. Uwamariya J, Nyandwi J, Mukanyangezi M, Kadima J. Sexual activity and emergency contraception among female students in the University of Rwanda. Int J Trop Dis Heal. 2015;8(4):170–7.
1766. Maloiy L, Wawire V. Status and Dynamics of Gender Mainstreaming in East Africa Community COVID-19 Social and Economic Response Policies, Strategies and Interventions. 2021;
1767. Pretorius L, Gibbs A, Crankshaw T, Willan S. Interventions targeting sexual and reproductive health and rights outcomes of young people living with HIV: a comprehensive review of current interventions from sub-Saharan Africa. Glob Health Action. 2015;8(1):28454.
1768. Haddad L, Wall KM, Vwalika B, Khu NH, Brill I, Kilembe W, et al. Contraceptive discontinuation and switching among couples receiving integrated HIV and family planning services in Lusaka, Zambia. Aids. 2013;27:S93–103.
1769. Warren CE, Mayhew SH, Hopkins J. The current status of research on the integration of sexual and reproductive health and HIV services. Stud Fam Plann. 2017;48(2):91–105.
1770. Warren CE, Mayhew SH, Vassall A, Kimani JK, Church K, Obure CD, et al. Study protocol for the Integra Initiative to assess the benefits and costs of integrating sexual and reproductive health and HIV services in Kenya and Swaziland. BMC Public Health. 2012;12:1–16.
1771. Wilcher R, Cates W. Reproductive choices for women with HIV. Bull World Health Organ. 2009;87(11):833–9.
1772. Abdool Karim S, Baxter C, Frohlich J, Abdool Karim Q. The need for multipurpose prevention technologies in sub‐Saharan Africa. BJOG An Int J Obstet Gynaecol. 2014;121:27–34.
1773. Matthews LT, Crankshaw T, Giddy J, Kaida A, Smit JA, Ware NC, et al. Reproductive decision-making and periconception practices among HIV-positive men and women attending HIV services in Durban, South Africa. AIDS Behav. 2013;17:461–70. Harrington BJ, Pence BW, John M, Melhado CG, Phulusa J, Mthiko B, et al. Prevalence and factors associated with antenatal depressive symptoms among women enrolled in Option B+ antenatal HIV care in Malawi: a cross-sectional analysis. J Ment Heal. 2019;28(2):198–205.
1774. Tesfa A, Bizuneh AD, Tesfaye T, Gebru AA, Ayene YY, Tamene BA. Assessment of knowledge, attitude and practice towards emergency contraceptive methods among female students in Seto Semero high school, Jimma town, south west Ethiopia. Sci J Public Heal. 2015;3(4):478–86.
1775. Hoque ME. Reported risky sexual practices amongst female undergraduate students in KwaZulu-Natal, South Africa. African J Prim Heal care Fam Med. 2011;3(1):1–6.
1776. Maharaj P. Male attitudes to family planning in the era of HIV/AIDS: evidence from KwaZulu-Natal, South Africa. J South Afr Stud. 2001;27(2):245–57.
1777. Greene S, Ion A, Kwaramba G, Smith S, Loutfy MR. “Why are you pregnant? What were you thinking?”: How women navigate experiences of HIV-related stigma in medical settings during pregnancy and birth. Soc Work Health Care. 2016;55(2):161–79.
1778. Tenkorang EY. Intimate partner violence and the sexual and reproductive health outcomes of women in Ghana. Heal Educ Behav. 2019;46(6):969–80.
1779. DeJong J, Jawad R, Mortagy I, Shepard B. The sexual and reproductive health of young people in the Arab countries and Iran. Reprod Health Matters. 2005;13(25):49–59.
1780. Tepper NK, Curtis KM, Jamieson DJ, Marchbanks PA. Update to CDC’s US Medical Eligibility Criteria for Contraceptive Use, 2010: revised recommendations for the use of hormonal contraception among women at high risk for HIV infection or infected with HIV. MMWR Morb Mortal Wkly Rep. 2012;61(24).
1781. Bobrova N, Sergeev O, Grechukhina T, Kapiga S. Social‐cognitive predictors of consistent condom use among young people in Moscow. Perspect Sex Reprod Health. 2005;37(4):174–8.
1782. Organization WH. Making the case for interventions linking sexual and reproductive health and HIV in proposals to the Global Fund to Fight AIDS, Tuberculosis and Malaria. World Health Organization; 2010.
1783. Liamputtong P. Women, motherhood, and living with HIV/AIDS: an introduction. In: Women, Motherhood and Living with HIV/AIDS: A Cross-Cultural Perspective. Springer; 2013. p. 1–24.
1784. Okereke CI. Unmet reproductive health needs and health-seeking behaviour of adolescents in Owerri, Nigeria. Afr J Reprod Health. 2010;14(1).
1785. Mshweshwe-Pakela NT, Matlakala MC, Mbengo F. Attitudes to, and knowledge and use of contraception among female learners attending a high school in Mdantsane. Afr J Nurs Midwifery. 2017;19(1):170–89.
1786. Mullu G, Gizachew A, Amare D, Alebel A, Wagnew F, Tiruneh C, et al. Prevalence of gender based violence and associated factors among female students of Menkorer high school in Debre Markos town, Northwest Ethiopia. Science (80- ). 2015;3(1):67–74.
1787. Ewunetie AA, Alemayehu M, Endalew B, Abiye H, Gedif G, Simieneh MM. Sexual and reproductive health problems and needs of street youths in East Gojjam Zone Administrative Towns, Ethiopia: Exploratory qualitative study. Adolesc Health Med Ther. 2022;55–66.
1788. K Shetty A. Epidemiology of HIV infection in women and children: a global perspective. Curr HIV Res. 2013;11(2):81–92.
1789. Bharat S, Mahendra VS. Meeting the sexual and reproductive health needs of people living with HIV: challenges for health care providers. Reprod Health Matters. 2007;15(sup29):93–112.
1790. Oyieke JBO, Obore S, Kigondu CS. Millennium development goal 5: a review of maternal mortality at the Kenyatta National Hospital, Nairobi. East Afr Med J. 2006;83(1):4–9.
1791. Omoro T, Gray SC, Otieno G, Mbeda C, Phillips-Howard PA, Hayes T, et al. Teen pregnancy in rural western Kenya: a public health issue. Int J Adolesc Youth. 2018;23(4):399–408.
1792. Hale F, Vazquez M. Violence against women living with HIV/AIDS: A background paper. Washingt DC Dev Connect. 2011;
1793. Allen RH. The role of family planning in poverty reduction. Obstet Gynecol. 2007;110(5):999–1002.
1794. Nedjat S, Moazen B, Rezaei F, Hajizadeh S, Majdzadeh R, Setayesh HR, et al. Sexual and reproductive health needs of HIV-positive people in Tehran, Iran: a mixed-method descriptive study. Int J Heal Policy Manag. 2015;4(9):591.
1795. Oindo ML. Contraception and sexuality among the youth in Kisumu, Kenya. Afr Health Sci. 2002;2(1):33–9.
1796. Pellowski JA, Price DM, Harrison AD, Tuthill EL, Myer L, Operario D, et al. A systematic review and meta-analysis of antiretroviral therapy (ART) adherence interventions for women living with HIV. AIDS Behav. 2019;23:1998–2013.
1797. Bankole A, Biddlecom A, Singh S, Guiella G, Zulu E. Sexual behavior, knowledge and information sources of very young adolescents in four sub-Saharan African countries. Afr J Reprod Health. 2007;11(3):28–43.
1798. Matseke G, Rodriguez VJ, Peltzer K, Jones D. Intimate partner violence among HIV positive pregnant women in South Africa. J Psychol Africa. 2016;26(3):259–66.
1799. Chersich MF, Rees H V. Vulnerability of women in southern Africa to infection with HIV: biological determinants and priority health sector interventions. Aids. 2008;22:S27–40.
1800. Juma M, Alaii J, Bartholomew LK, Askew I, Van den Born B. Understanding orphan and non-orphan adolescents’ sexual risks in the context of poverty: a qualitative study in Nyanza Province, Kenya. BMC Int Health Hum Rights. 2013;13:1–8.
1801. Kriel Y, Milford C, Cordero J, Suleman F, Beksinska M, Steyn P, et al. Male partner influence on family planning and contraceptive use: perspectives from community members and healthcare providers in KwaZulu-Natal, South Africa. Reprod Health. 2019;16:1–15.
1802. Moradi F, Balaghi Z, Joulaei H, Zare N, Mohammadi S, Moghadami M. Unmet Need for Prevention of Unwanted Pregnancy in Shiraz. 2014;
1803. Tessema ZT, Teshale AB, Tesema GA, Tamirat KS. Determinants of completing recommended antenatal care utilization in sub-Saharan from 2006 to 2018: evidence from 36 countries using Demographic and Health Surveys. BMC Pregnancy Childbirth. 2021;21:1–12.
1804. Bazant ES, Koenig MA. Women’s satisfaction with delivery care in Nairobi’s informal settlements. Int J Qual Heal Care. 2009;21(2):79–86.
1805. Greene S, Ion A, Elston D, Kwaramba G, Smith S, Carvalhal A, et al. “why aren’t you breastfeeding?”: how mothers living with HIV talk about infant feeding in a “breast is best” world. Health Care Women Int. 2015;36(8):883–901.
1806. Nkosi M. Male circumcision as an HIV prevention strategy and implications for woMen’s sexual and reproductive health rights. Agenda. 2008;22(75):141–54.
1807. Weinrib R, Minnis A, Agot K, Ahmed K, Owino F, Manenzhe K, et al. End-users’ poduct preference across three multipurpose prevention technology delivery forms: baseline results from young women in Kenya and South Africa. AIDS Behav. 2018;22:133–45.
1808. Osinde MO, Kaye DK, Kakaire O. Intimate partner violence among women with HIV infection in rural Uganda: critical implications for policy and practice. BMC Womens Health. 2011;11:1–7.
1809. Adhikari R. Factors affecting awareness of emergency contraception among college students in Kathmandu, Nepal. BMC Womens Health. 2009;9:1–5.
1810. Ward MC. A different disease: HIV/AIDS and health care for women in poverty. Cult Med Psychiatry. 1993;17:413–30.
1811. Maina WK, Kim AA, Rutherford GW, Harper M, K’Oyugi BO, Sharif S, et al. Kenya AIDS Indicator Surveys 2007 and 2012: implications for public health policies for HIV prevention and treatment. JAIDS J Acquir Immune Defic Syndr. 2014;66:S130–7.
1812. Birungi H, Obare F, Mugisha JF, Evelia H, Nyombi J. Preventive service needs of young people perinatally infected with HIV in Uganda. AIDS Care. 2009;21(6):725–31.
1813. Renzaho AMN, Kamara JK, Georgeou N, Kamanga G. Sexual, reproductive health needs, and rights of young people in slum areas of Kampala, Uganda: a cross sectional study. PLoS One. 2017;12(1):e0169721.
1814. Obiyan MO, Olaleye AO, Oyinlola FF, Folayan MO. Factors associated with pregnancy and induced abortion among street-involved female adolescents in two Nigeria urban cities: a mixed-method study. BMC Health Serv Res. 2023;23(1):25.
1815. Joyce C, Keraka ÂM, Njagi J. Assessment of the knowledge on pre conception care among women of reproductive age in Ruiru sub-county, Kiambu county, Kenya. Glob J Heal Sci. 2018;3(1):82–100.
1816. Mutsindikwa T, Ashipala DO, Tomas N, Endjala T. Knowledge, Attitudes and Practices of Contraception among tertiary students at the University Campus in Namibia. Glob J Heal Sci. 2019;11:180.
1817. Musyimi CW, Mutiso VN, Nyamai DN, Ebuenyi I, Ndetei DM. Suicidal behavior risks during adolescent pregnancy in a low-resource setting: A qualitative study. PLoS One. 2020;15(7):e0236269.
1818. Tadesse G, Yakob B. Risky sexual behaviors among female youth in Tiss Abay, a semi-urban area of the Amhara Region, Ethiopia. PLoS One. 2015;10(3):e0119050.
1819. Roudi-Fahimi F. Women’s reproductive health in the Middle East and North Africa. Population Reference Bureau Washington, DC; 2003.
